# Mosunetuzumab with polatuzumab vedotin in relapsed or refractory aggressive large B cell lymphoma: a phase 1b/2 trial

**DOI:** 10.1038/s41591-023-02726-5

**Published:** 2023-12-10

**Authors:** Lihua E. Budde, Adam J. Olszewski, Sarit Assouline, Izidore S. Lossos, Catherine Diefenbach, Manali Kamdar, Nilanjan Ghosh, Dipenkumar Modi, Waleed Sabry, Seema Naik, Amitkumar Mehta, Shazia K. Nakhoda, Stephen D. Smith, Kathleen Dorritie, Ting Jia, Song Pham, Ling-Yuh Huw, Jing Jing, Hao Wu, Wahib S. Ead, Iris To, Connie Lee Batlevi, Michael C. Wei, Julio C. Chavez

**Affiliations:** 1 City of Hope Comprehensive Cancer Center, Duarte, CA USA; 2 Brown University, Providence, RI USA; 3 Jewish General Hospital, McGill University, Montreal, Quebec Canada; 4 Sylvester Comprehensive Cancer Center, University of Miami Health System, Miami, FL USA; 5 Perlmutter Cancer Center at NYU Langone Health, New York, NY USA; 6 University of Colorado, Aurora, CO USA; 7 Hematologic Oncology and Blood Disorders, Atrium Health Levine Cancer Institute, Charlotte, NC USA; 8 Karmanos Cancer Institute/Wayne State University, Detroit, MI USA; 9 Saskatoon Cancer Center, Saskatoon, Saskatchewan Canada; 10 Penn State Cancer Institute, Hershey, PA USA; 11 University of Alabama at Birmingham, Birmingham, AL USA; 12 Fox Chase Cancer Center, Philadelphia, PA USA; 13 Fred Hutchinson Cancer Center, Seattle, WA USA; 14 UPMC Hillman Cancer Center, University of Pittsburgh, Pittsburgh, PA USA; 15 Roche (China) Holding Ltd, Shanghai, China; 16 F. Hoffmann-La Roche Ltd, Mississauga, Ontario Canada; 17 Genentech, Inc., South San Francisco, CA USA; 18 Moffitt Cancer Center, Tampa, FL USA

**Keywords:** Immunotherapy, Phase II trials, B-cell lymphoma, Cancer immunotherapy

## Abstract

Relapsed/refractory aggressive large B cell lymphoma (LBCL) remains an area of unmet need. Here we report the primary analysis of a phase 1b/2 trial of outpatient mosunetuzumab (a CD20xCD3 T-cell-engaging bispecific antibody) plus polatuzumab vedotin (an anti-CD79B antibody–drug conjugate) in relapsed/refractory LBCL. The phase 2 component is a single arm of an ongoing multi-arm trial. The primary endpoint during dose expansion was independent review committee (IRC)-assessed best overall response rate. Secondary endpoints included investigator-assessed overall response rate, complete response, duration of response, progression-free survival and overall survival. At data cutoff, 120 patients were enrolled (22 dose escalation, 98 dose expansion). The primary endpoint was met during dose expansion, with IRC-assessed best overall response rate and complete response rates of 59.2% (58/98; 95% confidence interval (CI): 48.8–69.0) and 45.9% (45/98; 95% CI: 35.8–56.3), respectively (median follow-up, 23.9 months). Median duration of complete was not reached (95% CI: 20.5–not estimable (NE)). Median progression-free survival was 11.4 months (95% CI: 6.2–18.7). Median overall survival was 23.3 months (95% CI: 14.8–NE). Across dose escalation and expansion, the most common grade 3 or higher adverse events were neutropenia (25.0%, 30/120) and fatigue (6.7%, 8/120). Any-grade cytokine release syndrome occurred in 16.7% of patients. These data demonstrate that mosunetuzumab plus polatuzumab vedotin has a favorable safety profile with highly durable responses suitable as second-line therapy in transplant-ineligible relapsed/refractory LBCL. ClinicalTrials.gov identifier: NCT03671018.

## Abstract (web summary)

In an ongoing phase 1/2 trial, mosunetuzumab, a CD20xCD3 bispecific antibody, plus polatuzumab vedotin, an anti-CD79B antibody–drug conjugate, had an overall response rate of 59.2%, supporting the potential of this combination for second-line treatment of patients with transplant-ineligible or CAR-T-cell-ineligible relapsed or refractory large B cell lymphoma.

## Main

Large B cell lymphoma (LBCL), the most common aggressive non-Hodgkin lymphoma (NHL)^1^, is managed in the front line with rituximab-based immunochemotherapy regimens that have curative potential, such as rituximab plus cyclophosphamide, doxorubicin, vincristine and prednisone (R-CHOP); or polatuzumab vedotin in combination with rituximab, cyclophosphamide, doxorubicin and prednisone (Pola-R-CHP)^2,3^. However, approximately 20–40% of patients with LBCL are either refractory to front-line therapy or experience subsequent relapse^3,4^.

Standard of care is evolving in the second-line treatment of LBCL and includes traditional salvage chemotherapy followed by consolidation with autologous stem cell transplant (ASCT) or chimeric antigen receptor (CAR)-T cell therapy in patients who are transplant ineligible or those with early disease relapse after front-line therapy^5–7^ or additional immunochemotherapy. Multiple challenges remain in delivering therapies with durable and curative potential in the relapsed/refractory (R/R) setting. Approximately half of patients with R/R LBCL are unsuitable for ASCT, and, even after ASCT, only 50% attain a durable remission^5–9^. For patients suitable for CAR-T cell therapy, there are multiple barriers, including manufacturing challenges, severe life-threatening toxicities and access to specialist treatment centers, which can increase health disparities^10,11^. Furthermore, approximately 50% of patients with LBCL do not respond or relapse after CAR-T cell therapy^5,6^. Although other treatments, including bispecific antibodies^12,13^, antibody–drug conjugates^14^, monoclonal antibody combinations^15,16^, targeted therapies^17^ and chemoimmunotherapy regimens^18,19^, are available, there remains a need to develop new regimens that balance safety, efficacy and patient access in R/R LBCL, especially for non-tertiary and community practices where many patients receive treatments.

Mosunetuzumab and polatuzumab vedotin have individually shown promising anti-lymphoma activity and manageable toxicity profiles in patients with R/R NHL^20,21^. Mosunetuzumab is an off-the-shelf CD20xCD3 T-cell-engaging bispecific antibody that engages and redirects T cells to eliminate malignant B cells^22^ and was recently approved in R/R follicular lymphoma (FL)^23^. Mosunetuzumab, which is administered in an outpatient setting, is efficacious with a favorable toxicity profile in patients with R/R diffuse large B cell lymphoma (DLBCL; NCT02500407)^24^, including patients who had received prior CAR-T cell therapy with an overall response rate (ORR) of 42.0% and a complete response rate of 23.9%^20^. Polatuzumab vedotin is an antibody–drug conjugate that is composed of an anti-CD79b monoclonal antibody conjugated by a protease-cleavable linker to a potent microtubule inhibitor, monomethyl auristatin E (MMAE)^3^. After binding to CD79b on B cells, polatuzumab vedotin is internalized; the linker is cleaved; and MMAE is released to inhibit intracellular division and induce apoptosis^25^. Polatuzumab vedotin in combination with chemoimmunotherapy is approved for the treatment of previously untreated and R/R DLBCL^26^.

Mosunetuzumab combined with polatuzumab vedotin (mosun-pola) targets distinct components of malignant B cell biology^21,27^, and initial reports demonstrated safety and efficacy, supporting development of this combination therapy as a fixed-duration outpatient regimen in second-line, transplant-ineligible LBCL^3,28,29^. Here we report the primary analysis of an ongoing study of the mosun-pola combination (NCT03671018).

## Results

### Study design

**Extended Data Fig. 1:**
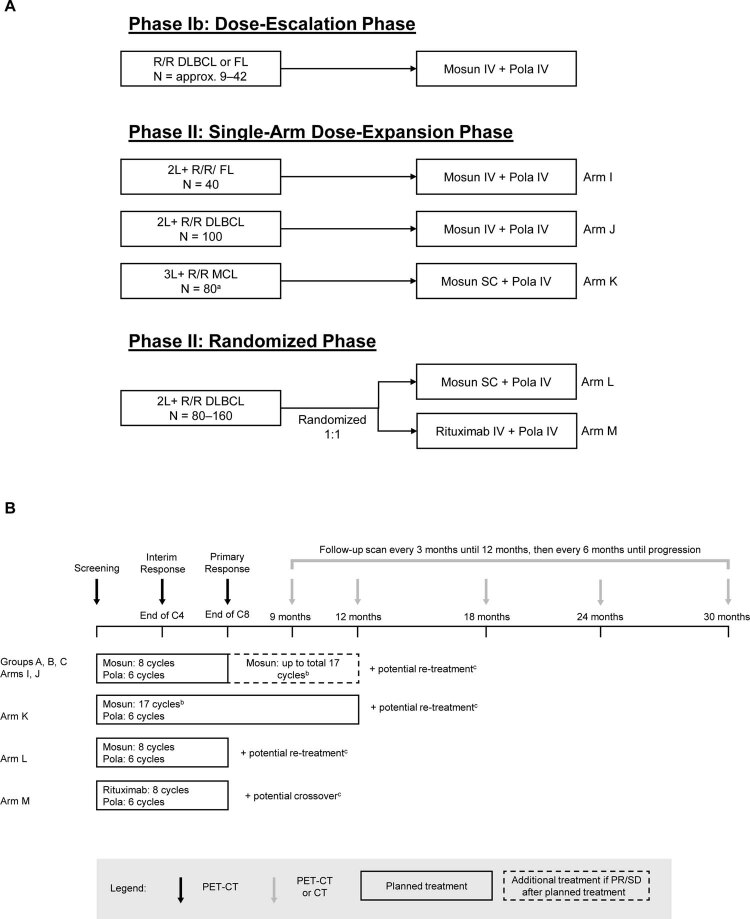
Study schema of: A, overview of study design, and B, overview of response assessments. ^a^Safety data of the first 6 safety-evaluable patients enrolled in Arm K will be reviewed by the IMC prior to enrolling the full expansion cohort; ^b^See protocol section 3.1.5 for mosun treatment beyond 8 cycles and re-treatment; ^c^See protocol section 3.1.5 for mosun re-treatment; ^d^See protocol section 3.1.5 for Arm M-crossover. 2 L, second line; C, cycle; CT, computerised tomography; FL, follicular lymphoma; DLBCL, diffuse large B-cell lymphoma; IMC, internal monitoring committee; IV, intravenous; MCL, mantle cell lymphoma; Mosun, mosunetuzumab; N, number; PET, positron emission tomography; Pola, polatuzumab vedotin; PR, partial response; R/R, relapsed/refractory; SC, subcutaneous; SD, stable disease.

This is an ongoing phase 1b/2 multi-arm clinical trial of mosun-pola in R/R NHL. Here we present the phase 1b dose-escalation cohort in R/R NHL and the single-arm, phase 2 dose-expansion cohort in patients with second-line and later R/R LBCL (Extended Data Fig. 1). The primary efficacy endpoint during dose expansion was independent review committee (IRC)-assessed best ORR. Secondary endpoints included investigator (INV)-assessed best ORR, best complete response rate and complete response rate at the time of the primary response assessment, duration of response (DoR), progression-free survival (PFS) and overall survival. Protocol-defined pharmacokinetic and biomarker endpoints were also assessed. Exploratory endpoints included the proportion of patients who underwent ASCT or allogeneic stem cell transplant (SCT) after achieving a response and the association of response with prognostic subtypes. Safety was evaluated through the incidence and severity of adverse events (AEs).

### Patients

**Fig. 1:**
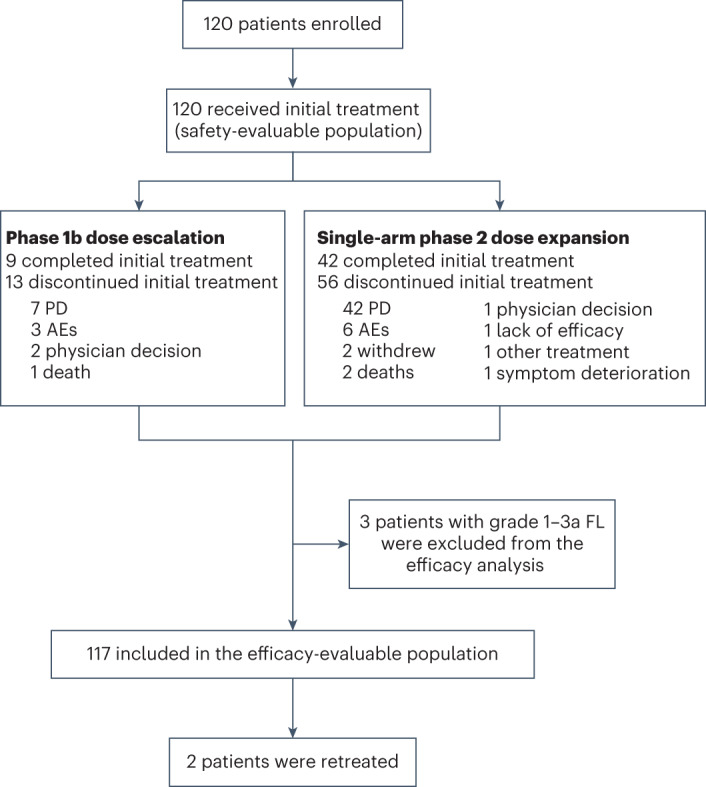
Patient disposition. A total of 22 patients were treated in the phase 1b dose-escalation cohort (*n* = 19 with R/R LBCL and *n* = 3 with R/R FL) and 98 patients in the phase 2 dose-expansion cohort (all with R/R LBCL). PD, progressive disease.

Between 25 September 2018 and 14 February 2022, 120 patients were enrolled from 15 sites across two countries (the USA and Canada), with 22 patients treated in the phase 1b dose-escalation cohort (*n* = 19 with R/R LBCL and *n* = 3 with R/R FL) and 98 patients treated in the phase 2 dose-expansion cohort (*n* = 98 with R/R LBCL) (Fig. 1). The overall safety population (*n* = 120) included all patients with DLBCL, high-grade B cell lymphoma (HGBCL), transformed FL, grade 3b FL or grade 1–3a FL, as described in the Methods. The overall efficacy population (*n* = 117) excluded three patients with histologically confirmed grade 1–3a FL.

Baseline demographics and clinical characteristics of the overall R/R NHL population (*n* = 120) and the phase 2 dose-expansion R/R LBCL cohort treated at the recommended phase 2 dose (RP2D) are described in Table 1. In the overall population, the median age was 68 years (range, 20–88); 85% had advanced-stage disease; and 64.2% had extranodal disease. Overall, 75 patients (62.5%) had DLBCL, 23 (19.2%) had HGBCL, 11 (9.2%) had transformed FL, eight (6.7%) had FL grade 3b and three (2.5%) had FL grade 1–3a. Of 109 patients with LBCL, 22 (20.2%) had double-hit or triple-hit lymphoma (DH/THL) (Table 1). The overall population received a median of two prior lines of therapy (range, 1–10), including CAR-T cell therapy (*n* = 42 [35.0%]) and ASCT (*n* = 15 [12.5%]). Sixty-nine patients (57.5%) were primary refractory, 93 (77.5%) were refractory to their last prior therapy and 100 (83.3%) were refractory to any prior anti-CD20 therapy. Among 42 patients who received prior CAR-T cell therapy, 33 (78.6%) were refractory to prior CAR-T cell therapy (Table 1). Patient demographics and clinical characteristics were comparable between the overall population and the dose-expansion cohort (Table 1). There were five major protocol deviations from the inclusion criteria, all related to missing tumor biopsy samples at screening. One major protocol deviation from the exclusion criteria was due to the patient not having the protocol-required 4-week washout period after prior rituximab treatment. None of these deviations was deemed to have a major impact on the overall efficacy or safety endpoints of this study.

**Table 1:** Baseline characteristics in the overall population (all patients with R/R NHL in the dose-escalation and dose-expansion cohorts; *n* = 120) and the dose-expansion cohort (R/R LBCL; safety-evaluable population; *n* = 98)

*n* (%) of patients unless stated	Overall population, *n* = 120	Dose-expansion cohort, *n* = 98
Median age (range), years	68 (20–88)	68 (20–88)
Male	81 (67.5)	70 (71.4)
ECOG performance status		
0	46 (38.3)	36 (36.7)
1	67 (55.8)	55 (56.1)
2	7 (5.8)	7 (7.1)
Ann Arbor stage at study entry		
I/II	18 (15.0)	13 (13.3)
III/IV	102 (85.0)	85 (86.7)
Extranodal involvement at study entry	77 (64.2)	65 (66.3)
NHL subtype		
DLBCL	75 (62.5)	68 (69.4)
HGBCL	23 (19.2)	18 (18.4)
Transformed FL	11 (9.2)	8 (8.2)
Grade 3b FL	8 (6.7)	4 (4.1)
Grade 1–3a FL	3 (2.5)	0
COO^a^	(*n* = 109)	(*n* = 94)
GCB	60 (55.0)	53 (56.4)
Non-GCB	38 (31.7)	33 (33.7)
Unknown	11 (10.1)	8 (8.5)
Double/triple-hit status	(*n* = 109)	(*n* = 94)
Yes	22 (20.2)	16 (17.0)
Double-expressor (*MYC* and *BCL2)*	18 (15.0)	14 (14.3)
LDH levels higher than local ULN	63 (52.5)	53 (54.1)
Bulky disease at study entry (>10 cm)	7 (5.8)	6 (6.1)
Median lines of prior therapy (range)	2 (1–10)	2 (1–8)
Previous lines of therapy		
1–2	63 (52.5)	56 (57.1)
≥3	57 (47.5)	42 (42.9)
Previous anti-lymphoma therapy		
Anti-CD20 antibody	120 (100.0)	98 (100.0)
Anthracycline	115 (95.8)	93 (94.9)
CAR-T cell therapy	42 (35.0)	35 (35.7)
ASCT	15 (12.5)	11 (11.2)
Relapsed/refractory^b^ status		
Refractory to last prior therapy	93 (77.5)	76 (77.6)
Refractory to first prior therapy	69 (57.5)	56 (57.1)
Refractory to any prior anti-CD20	100 (83.3)	80 (81.6)
Refractory to prior CAR-T cell therapy	33/42 (78.6)	26/35 (74.3)

Clinical cutoff date: 6 July 2023.

^a^Patients with de novo LBCL, HGBCL and trFL (109 patients in the overall population and 94 patients in the dose-expansion cohort) were evaluable for COO assessments. Non-GCB includes non-GCB derived from immunohistochemistry, ABC derived from GEP and unclassified by GEP. GCB included GCB derived from immunohistochemistry and/or GEP.

^b^Defined as not achieving a response (complete or partial) or progressing within ≤6 months of applicable treatment. ABC, activated B cell-like; GEP, gene expression profiling; LDH, lactate dehydrogenase; trFL, transformed follicular lymphoma.

### Phase 1b dose escalation to determine the RP2D

Mosunetuzumab was administered intravenously in 21-day cycles with cycle 1 step-up dosing: 1 mg on cycle 1, day 1 (C1D1); 2 mg on C1D8; escalated to a loading dose (9 mg, 20 mg, 40 mg or 60 mg) on C1D15 and C2D1; and then continued at the target dose (9 mg, 20 mg, 40 mg or 30 mg) from C3 onwards. Patients with a complete response completed mosunetuzumab after C8, whereas those with a partial response or stable disease continued mosunetuzumab for a total of 17 cycles. Polatuzumab vedotin was administered intravenously before mosunetuzumab at the standard dose of 1.8 mg/kg on D1 of C1–C6 (see Methods for additional details).

The maximum tolerated dose (MTD) was not reached with any of the mosunetuzumab dosing schedules investigated: 1/2/9 mg (*n* = 7), 1/2/20 mg (*n* = 3), 1/2/40 mg (*n* = 6) and 1/2/60/30 mg (*n* = 6). One dose-limiting toxicity (DLT; see protocol in the Supplementary Appendix for DLT definitions) was observed in a patient at the 1/2/40 mg dose who developed asymptomatic, new-onset grade 3 atrial fibrillation. The RP2D of mosunetuzumab was determined to be 1/2/60/30 mg in combination with polatuzumab vedotin 1.8 mg/kg, with six patients treated at this dose and schedule during this part of the study.

In the phase 1 cohort, per INV assessment, best overall complete response rate based on positron emission tomography-computed tomography (PET-CT) and/or CT scan was 47.4% (9/19; 95% CI: 24.5–71.1), and best ORR was 63.2% (12/19; 95% CI: 38.4–83.7), with median DoR not reached (95% CI, 6.3–NE) based on a median follow-up of 41.5 months.

### Efficacy outcomes in the phase 2 dose-expansion cohort

Ninety-eight patients with R/R LBCL were treated at the 1/2/60/30 mg mosunetuzumab dose schedule in combination with 1.8 mg/kg polatuzumab vedotin. At the data cutoff date (6 July 2023), the median follow-up was 23.9 months (95% CI: 21.3–26.8). Median treatment durations of mosunetuzumab and polatuzumab vedotin were 4.9 months and 3.5 months, respectively, with patients receiving a median of eight mosunetuzumab cycles and six polatuzumab vedotin cycles. Forty-two patients (42.9%) completed initial treatment, and 56 patients (57.1%) discontinued due to progressive disease (*n* = 42), AEs (*n* = 6), death (*n* = 2), patient withdrawal (*n* = 2), lack of efficacy (*n* = 1), use of another anti-cancer therapy (*n* = 1), physician decision (*n* = 1) or symptomatic deterioration (*n* = 1).

**Fig. 2:**
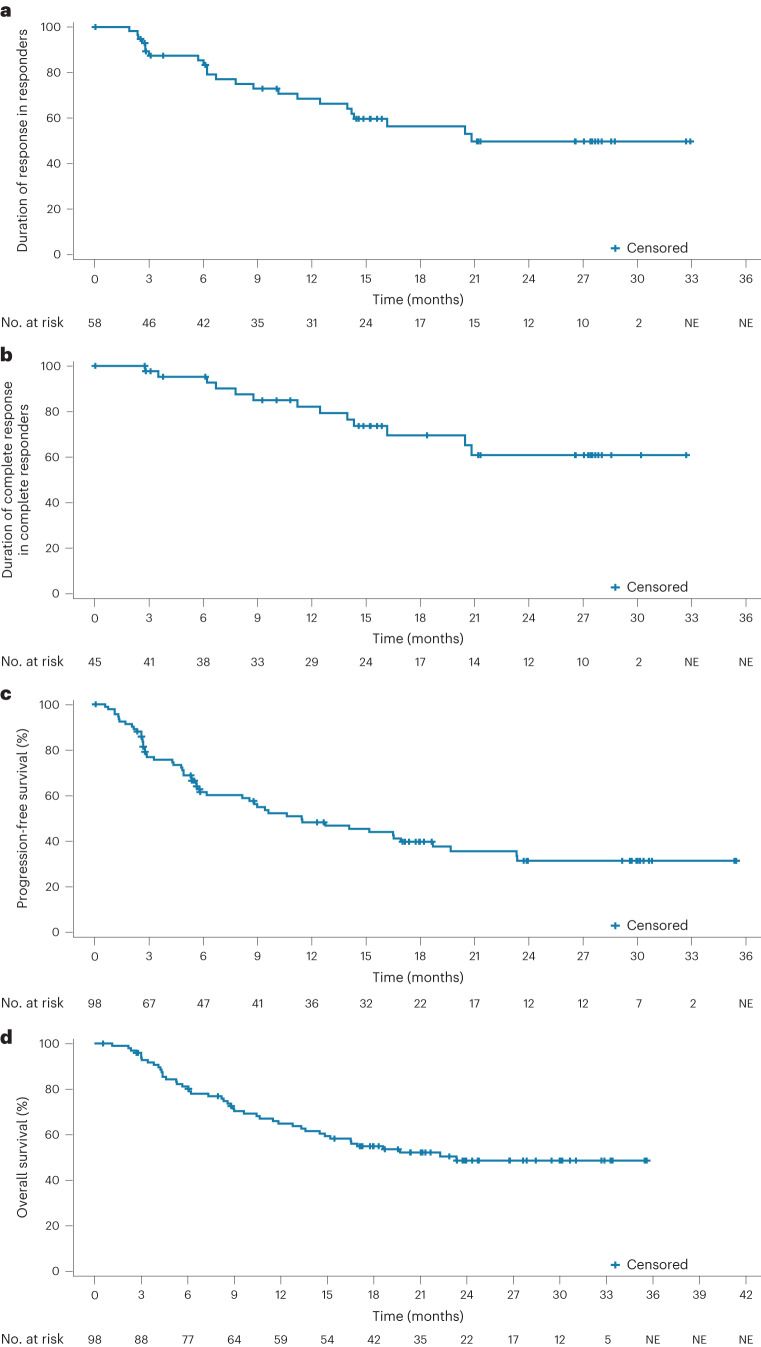
Kaplan–Meier plots by IRC. **a**, DoR in responders (*n* = 58). **b**, DoCR in complete responders (*n* = 45). **c**,**d**, Progression-free survival (**c**) and overall survival (**d**) in the dose-expansion cohort (*n* = 98; efficacy-evaluable population).

Efficacy results are shown in Table 2. The primary efficacy endpoint of best ORR by IRC by Lugano 2014 response criteria^30^ was met. Best ORR by IRC assessment, based on PET-CT and/or CT scan, was 59.2% (95% CI: 48.8–69.0; *P* = 0.0003 at 2.5% one-sided level of significance using an exact binomial test, compared to a historical control rate of 42%)^31^. Best complete response was 45.9% (95% CI: 35.8–56.3; Table 2). Among 58 responders, the Kaplan–Meier-estimated median DoR was 20.8 months (95% CI: 14.2–NE; Fig. 2a and Table 2), and the 24-month event-free rate was 49.7% (95% CI: 34.3–65.2). Among the 45 patients with complete response, median duration of complete response (DoCR) was not reached (95% CI: 20.5–NE; Fig. 2b), and the Kaplan–Meier-estimated 24-month event-free rate was 60.8% (95% CI: 43.2–78.4; Table 2). Median IRC-assessed PFS was 11.4 months (95% CI: 6.2–18.7). Median overall survival was 23.3 months (95% CI: 14.8–NE; Fig. 2d).

**Table 2:** Efficacy summary in the R/R LBCL overall population (that is, all patients with R/R LBCL in the dose-escalation and dose-expansion cohorts; *n* = 117) and the dose-expansion cohort (R/R LBCL; efficacy-evaluable population; *n* = 98)

	Overall population, *n* = 117^a^	Dose-expansion cohort, *n* = 98	
	INV	INV	IRC
Best ORR, *n* (%) [95% CI]	73 (62.4) [53.0–71.2]	62 (63.3) [52.9–72.8]	58 (59.2) [48.8–69.0]
Best complete response rate, *n* (%) [95% CI]	59 (50.4) [41.0–59.8]	50 (51.0) [40.7–61.3]	45 (45.9) [35.8–56.3]
ORR at time of PRA, *n* (%) [95% CI]^b^		46 (46.9) [36.8–57.3]	45 (46.0) [35.8–56.3]
Complete response rate at time of PRA, *n* (%) [95% CI]^b^		42 (42.9) [32.9–53.3]	42 (42.9) [32.9–53.3]
Median time to first response (range), months	2.7 (2.0–6.0)	2.7 (2.0–6.0)	2.6 (1.0–6.0)
Median DoR (95% CI), months	20.8 (14.8–NE)	20.5 (14.0–NE)	20.8 (14.2–NE)
Event-free rate (95% CI), %			
12 months	65.5 (53.9–77.0)	64.1 (51.3–76.8)	68.5 (55.6–81.4)
24 months	49.6 (36.0–63.2)	46.7 (31.5–61.9)	49.7 (34.3–65.2)
Median time to first complete response, months (range)	2.8 (2.0–8.0)	2.8 (2.0–8.0)	2.7 (2.0–6.0)
Median DoCR (95% CI), months	NE (16.2–NE)	NE (16.1–NE)	NE (20.5–NE)
Event-free rate (95% CI), %			
12 months	75.2 (63.4–87.0)	73.4 (60.4–86.4)	82.1 (70.0–94.2)
24 months	57.6 (42.2–73.0)	51.9 (34.5–69.2)	60.8 (43.2–78.4)
Median PFS (95% CI), months	9.4 (5.6–16.9)	9.4 (5.6–16.9)	11.4 (6.2–18.7)
Event-free rate (95% CI), %			
12 months	45.8 (36.4–55.2)	45.2 (35.0–55.4)	48.2 (37.3–59.0)
24 months	31.6 (21.9–41.3)	29.4 (18.8–39.9)	31.3 (20.1–42.6)
Median EFS, months^b^		6.0 (5.4–11.9)	6.9 (5.4–14.0)
Event-free rate (95% CI), %			
12 months		39.3 (29.4–49.2)	42.1 (31.9–52.3)
24 months		28.1 (18.2–37.9)	28.4 (18.4–38.4)
Median overall survival (95% CI), months	27.7 (15.2–NE)	23.3 (14.8–NE)	
Event-free rate (95% CI), %			
12 months	65.7 (56.9–74.6)	64.9 (55.2–74.5)	
24 months	51.3 (41.6–61.0)	48.6 (37.9–59.3)	

Note: In the 62 patients with INV-assessed response, the median DoR and the median DoCR were calculated from 61 patients, as one patient had partial response and progressive disease at the same assessment.

^a^Three patients with histologically confirmed grade 1–3a FL were excluded from the efficacy analysis.

^b^Secondary endpoint for the dose-expansion cohort only.

EFS, event-free survival.

**Extended Data Fig. 2:**
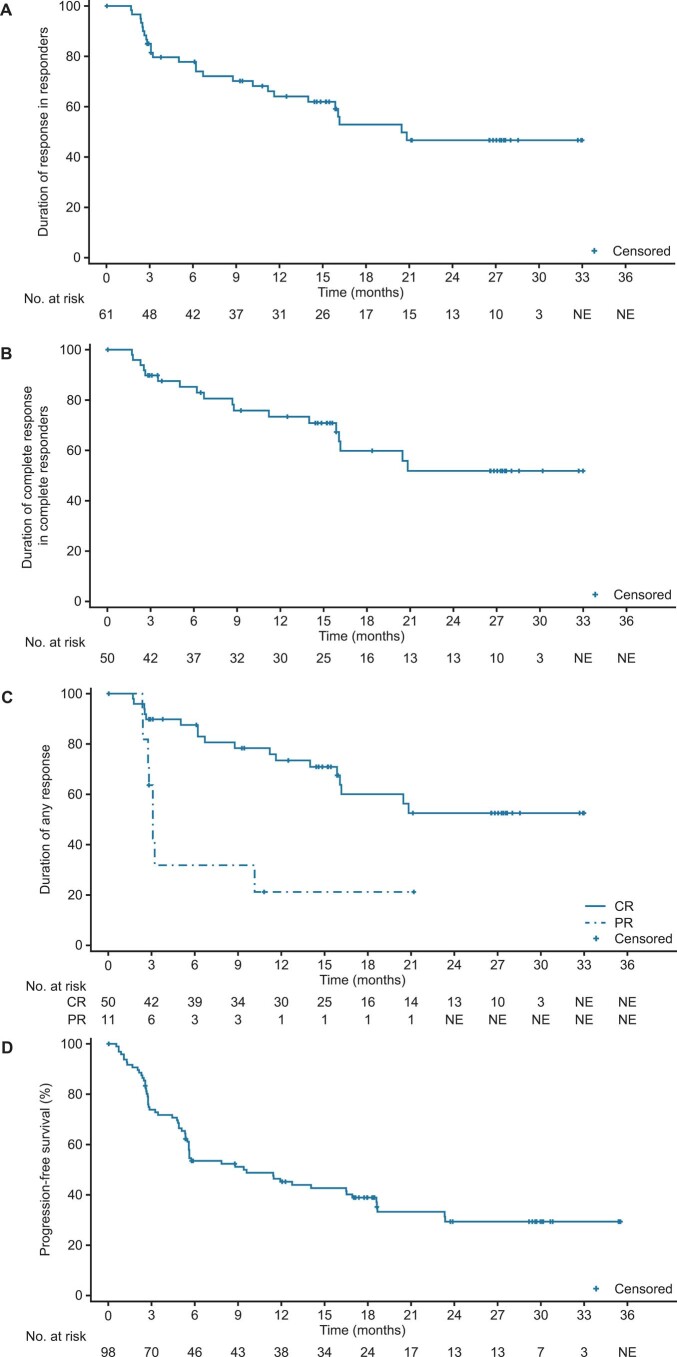
Kaplan–Meier plots by investigator of: A, duration of response in responders (n = 61), B, duration of complete response in complete responders (n = 50), and C, duration of complete and partial responses among responders (n = 61), and D, progression-free survival in the dose-expansion cohort (n = 98; efficacy-evaluable population). Note: in Panel A and C, one patient had PR and PD at the same assessment and was therefore excluded from the duration of response among responders curves. CR, complete response; NE, not estimable; No., number; PD, progressive disease; PR, partial response.

Per INV assessment, best ORR was 63.3% (95% CI: 52.9–72.8), and best complete response was 51.0% (95% CI: 40.7–61.3) (Table 2). DoR and DoCR are shown in Extended Data Fig. 2a,b. Concordance between IRC and INV assessments of DoR was 82%. Six patients who were initially assessed as achieving partial response converted to complete response at subsequent follow-up assessments. Five patients converted from partial response to complete response before completion of C8. One patient with a partial response at the end of C8 converted to complete response after continuing with additional mosunetuzumab until C17. Median DoR was prolonged in patients with complete response versus partial response (not reached (95% CI: 16.1–NE) versus 3.1 months (95% CI: 2.8–10.2)) (Extended Data Fig. 2c). Kaplan–Meier-estimated PFS according to INV is shown in Extended Data Fig. 2d.

Patients with complete response who subsequently progressed after initial treatment were permitted to receive mosun-pola retreatment. Two patients were retreated (one experienced a complete response and one a partial response) with both responses lasting more than 6 months before progression.

### Efficacy in high-risk subgroups

**Fig. 3:**
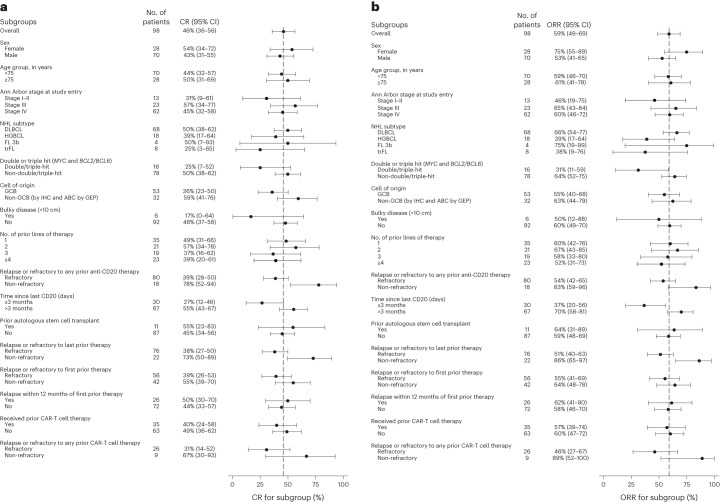
Prespecified subgroup analysis of complete response and ORR in the dose-expansion cohort. a,b, Complete response (CR) rates (**a**) and ORR (**b**) were determined by an IRC. Squares denote the rates, and error bars indicate two-sided exact Clopper–Pearson 95% CIs. The dashed line indicates the response in the overall main analysis cohort (*n* = 98). ABC, activated B cell-like; GEP, gene expression profiling; trFL, transformed follicular lymphoma.

Prespecified subgroup analyses of IRC-assessed best ORR and complete response rates using PET-CT in the phase 2 dose-expansion cohort are shown in Fig. 3. Durable responses were observed with mosun-pola in patients with high-risk pathology or clinical disease course.

Median PFS was 16.5 months (95% CI: 5.6–23.4) in patients who had received one prior line of therapy and 11.4 months (95% CI: 5.7–18.7) in those who had received two or more lines. In patients with DH/THL, median DoR and PFS were 20.5 months (95% CI: 3.0–NE) and 6.2 months (95% CI: 2.6–16.5), respectively. In 35 patients who received prior CAR-T cell therapy in the dose-expansion cohort, the median DoR was NE (95% CI: 8.8–NE), and the median PFS was 9.6 months (95% CI: 4.9–NE). In 26 patients who were refractory to CAR-T cell therapies, the median DoR was 12.5 months (95% CI: 2.8–NE), and the median PFS was 5.7 months (95% CI: 4.3–11.5). In patients with primary refractory disease, median DoR and PFS were 20.5 months (95% CI: 6.7–NE) and 8.5 months (95% CI: 4.9–16.9), respectively.

### Treatments after progression or completion of mosun-pola

Overall, 52 patients received subsequent anti-lymphoma treatment after mosun-pola. Four patients received ASCT as consolidative therapy, including two patients who achieved a complete response and received consolidative ASCT at the end of mosun-pola treatment. Seven patients received allogeneic SCT, including two patients who achieved a complete response with mosun-pola and subsequently received allogeneic SCT as consolidative therapy. Overall, 13 patients received CAR-T cell therapy, one of whom received CAR-T cell therapy while in partial response to mosun-pola. Five patients received polatuzumab vedotin–based therapy in the context of a polatuzumab vedotin–containing regimen as the next line of therapy.

### Safety

Safety of mosun-pola was consistent in the overall safety population and in the phase 2 dose-expansion cohort treated at the RP2D (Table 3 and Extended Data Table 1). The most common (≥20%) AEs of any grade in the overall safety cohort were fatigue (46.7%), neutropenia (35.0%), diarrhea (30.8%), nausea (30.0%), decreased appetite (22.5%), headache (21.7%), pyrexia (20.0%) and dry skin (20.0%) (Table 3).

**Table 3:** AE summary in the overall cohort (R/R NHL in the dose-escalation and dose-expansion cohorts; *n* = 120) and the dose-expansion cohort (R/R LBCL; *n* = 98)

*n* (%)	Overall cohort, *n* = 120	Dose-expansion cohort, *n* = 98
Any AE	119 (99.2)	97 (99.0)
Most common AEs (occurring in ≥20% of patients)		
Fatigue	56 (46.7)	45 (45.9)
Neutropenia^a^	42 (35.0)	29 (29.6)
Diarrhea	37 (30.8)	24 (24.5)
Nausea	36 (30.0)	26 (26.5)
Decreased appetite	27 (22.5)	21 (21.4)
Headache	26 (21.7)	21 (21.4)
Pyrexia	24 (20.0)	22 (22.4)
Dry skin	24 (20.0)	21 (21.4)
CRS^b^	20 (16.7)	18 (18.4)
Any mosun-pola-related AE	108 (90.0)	88 (89.8)
Any grade 3/4 AE	68 (56.7)	54 (55.1)
Most common grade 3/4 AEs (occurring in ≥5% of patients)		
Neutropenia^a^	30 (25.0)	20 (20.4)
Fatigue	8 (6.7)	6 (6.1)
Any mosun-pola-related grade 3/4 AE	46 (38.3)	34 (34.7)
Grade 5 AEs (not including progressive disease)^c^	5 (4.2)	3 (3.1)
Mosun-pola-related grade 5 AE	0	0
AEs of special interest		
CRS		
Grade 3	3 (2.5)	3 (3.1)
Grade 4	0	0
Treatment-related neurologic AEs potentially consistent with ICANS		
Grade 1	4 (3.3)	3 (3.1)
Grade 2	0	0
Grade 3	1 (0.8)	1 (1.0)
Grade 4	1 (0.8)	1 (1.0)
Grade 1 tumor flare	1 (0.8)	1 (1.0)
Febrile neutropenia	0	0
Any AE leading to discontinuation of mosunetuzumab	7 (5.8)	4 (4.1)
Any mosunetuzumab-related AE leading to discontinuation of mosunetuzumab	2 (1.7)	1 (1.0)
Any AE leading to discontinuation of polatuzumab vedotin	11 (9.2)	7 (7.1)
Any polatuzumab vedotin-related AE leading to discontinuation of polatuzumab vedotin	7 (5.8)	4 (4.1)
Any AE leading to mosunetuzumab dose interruption	45 (37.5)	37 (37.8)
Any AE leading to polatuzumab vedotin dose modification/interruption	39 (32.5)	32 (32.7)

^a^Includes the preferred terms neutropenia and decreased neutrophil count.

^b^According to ASTCT 2019 criteria.

^c^Grade 5 AEs in the overall cohort included two patients (1.7%) with COVID-19 pneumonia and one patient (0.8%) each with respiratory failure, sudden cardiac death and pneumonia. Grade 5 AEs in the dose-expansion cohort included two patients (2.0%) with COVID-19 pneumonia and one patient (1.0%) with pneumonia.

Grade 3/4 AEs were reported in 56.7% of patients in the overall safety cohort, and the most common (≥5%) grade 3/4 AEs were neutropenia (25.0%) and fatigue (6.7%) (Table 3). Causality of treatment-related AEs was assessed by the INV. Treatment-related grade 3/4 AEs occurred in 38.3% of patients, and grade 5 AEs (not including progressive disease) occurred in five patients (two patients (1.7%) had COVID-19 pneumonia, and one patient (0.8%) each had respiratory failure, sudden cardiac death and pneumonia). Twelve patients (10.0%) experienced AEs that led to mosunetuzumab and/or polatuzumab vedotin discontinuation, of which eight were considered treatment related: one event each of pneumonitis (grade 3), cellulitis (grade 3) and encephalopathy (grade 4); two events of peripheral neuropathy (grade 1 and 2, respectively); and three events of peripheral sensory neuropathy (two grade 2 and one grade 3). AEs led to mosunetuzumab dose interruption in 45 patients (37.5%) and polatuzumab vedotin dose modification/interruption in 39 patients (32.5%) (Table 3).

**Extended Data Fig. 3:**
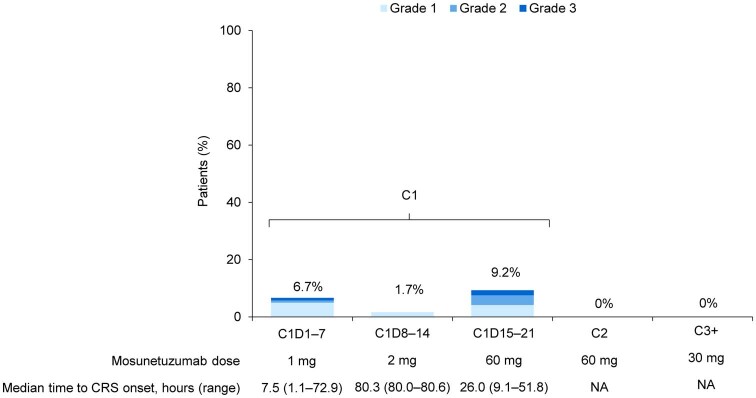
Patients (%) with CRS events by cycle and grade in the overall R/R LBCL population (safety evaluable population). CRS was graded using American Society for Transplantation and Cellular Therapy criteria. C, cycle; CRS, cytokine release syndrome; D, day; LBCL, large B-cell lymphoma; NA, not applicable; R/R, relapsed/refractory.

Cytokine release syndrome (CRS) occurred in 20 of 120 patients (16.7%; Table 3). Twelve patients (10.0%) had grade 1 CRS; five (4.2%) had grade 2 CRS; and three (2.5%) had grade 3 CRS. CRS onset most commonly occurred after C1D1 (eight patients, 6.7%) or C1D15 (11 patients, 9.2%), and two patients (1.7%) had CRS after C1D8. One patient had recurrent CRS with a grade 1 event after C1D1, a further grade 2 event after C1D15 and then no subsequent events (Extended Data Fig. 3). The median time to first CRS onset relative to the most recent dose was 1 day (range, 0–2), and the median duration of CRS was 2 days (range, 1–5). No patients developed CRS events beyond C1. CRS management strategies consisted of corticosteroids in six of 20 patients (30.0%), intravenous fluids in four of 20 patients (20.0%), tocilizumab in three of 20 patients (15.0%), and a single vasopressor and high-flow and low-flow oxygen each in two of 20 patients (10.0%) (Extended Data Table 2). Rates of CRS in the dose-expansion cohort were consistent with those in the overall population (any-grade CRS in 18/98 patients (18.4%), including grade 1 in 10 patients (10.2%), grade 2 in five patients (5.1%) and grade 3 in three patients (3.1%)) (Table 3).

Treatment-related neurologic AEs potentially consistent with immune effector cell–associated neurotoxicity syndrome (ICANS) occurred in six patients (5.0%) in the overall safety population, of whom five (5.1%) were in the dose-expansion cohort. Five patients had grade 1 events of lethargy, attention changes, syncope, confusion and mental status change, respectively. One patient had grade 4 encephalopathy on study D12 in the context of baseline mild dementia made worse from hospitalization and acute congestive heart failure leading to hypoxia. Another patient had grade 3 confusional state and grade 3 dysarthria in the setting of concurrent grade 2 CRS and grade 3 pneumonia (all starting on study D23), and the patient ultimately died from pneumonia.

Peripheral neuropathy occurred in 37 of 120 patients (30.8%), of whom 35 (29.2%) experienced events that were considered related to treatment. Of these, 34 patients (28.3%) had grade 1 or 2 events, and three patients (2.5%) had grade 3 events, which included two events of neuropathy peripheral and one event of sensory peripheral neuropathy. Among 37 patients who experienced peripheral neuropathy events, 11 (29.7%) had recovered by the time of data cutoff. Median time to onset of first peripheral neuropathy events was 39 days (range, 1–223), with a median duration of 55 days (range, 1–353). Five patients (4.2%) experienced peripheral neuropathy events that led to polatuzumab vedotin discontinuation. Similar rates of peripheral neuropathy were observed during dose expansion, in which events occurred in 28 patients (28.6%; all grade 1 or 2) and were considered to be related to treatment in 26 patients (26.5%).

Neutropenia occurred in 42 of 120 patients (35.0%; grade 3 or 4, 25.0%), of whom 38 (31.7%) experienced neutropenia that was considered related to treatment. A total of 33 of 40 patients (82.5%) with recovered/resolved neutropenia received granulocyte colony-stimulating factor. The median time to onset of neutropenia was 43 days (range, 2–168), and the median duration was 8 days (range, 1–809). There were no events of febrile neutropenia. No serious infections with concurrent neutropenia were noted. Rates of neutropenia in the dose-expansion cohort were consistent with those in the overall population (any-grade neutropenia in 29/98 patients (29.6%), grade 3 or 4, 20.4%), and 27 patients (27.6%) experienced neutropenia that was considered related to treatment.

Tumor flare events were reported in three patients (2.5%), all of which occurred before C2. All events were grade 1, non-serious and resolved by the time of data cutoff.

Infections occurred in 47 of 120 patients (39.2%; grade 3 or 4, 8.3%), of whom 17 (14.2%) experienced infections that were considered related to treatment. The most common infection was pneumonia in 11 patients (9.2%). Grade 5 infection occurred in three patients (2.5%), including one event of non-COVID pneumonia and two events of COVID-19 pneumonia. Ten patients (8.3%) had COVID-associated AEs. Among these, five patients (4.2%) were reported to have COVID-19 (including one grade 3 event and one grade 4 event). Another five patients (4.2%) were reported to have COVID-19 pneumonia, of whom two (1.7%) had grade 3 events and two (1.7%) had grade 5 events (previously described). One patient (0.9%) had grade 3 severe acute respiratory syndrome coronavirus 2 (SARS-CoV-2) sepsis, and one patient (0.9%) had grade 2 coronavirus test positive. In these 10 patients with COVID-related events, two patients (1.7%) had serious COVID-19, and four patients (3.3%) had serious COVID-19 pneumonia. Rates of infection in the dose-expansion cohort were consistent with those in the overall population (any-grade infection in 40/98 patients (40.8%); grade 3 or 4, 8.1%), and 13 patients (13.3%) experienced infection that was considered related to treatment.

### Pharmacokinetics

**Extended Data Fig. 4:**
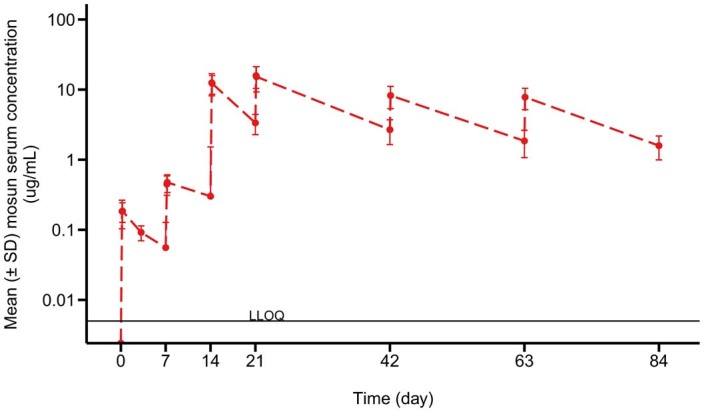
Mosunetuzumab serum concentration over time in the dose-expansion cohort (n = 98). LLOQ, lower limit of quantification; mosun, mosunetuzumab; SD, standard deviation.

The pharmacokinetics of mosunetuzumab administered in combination with polatuzumab vedotin in the phase 2 dose-expansion cohort were comparable to those previously reported with single-agent mosunetuzumab (Extended Data Fig. 4)^15,21^.

### Pharmacodynamics

**Extended Data Fig. 5:**
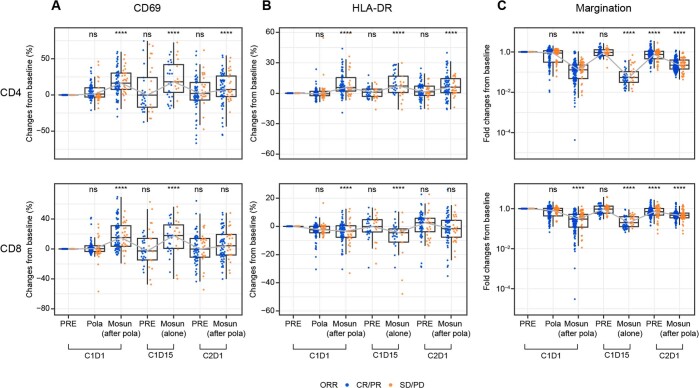
Impact of polatuzumab vedotin on the pharmacodynamics of mosunetuzumab by assessing the CD4+ and CD8+ change from baseline for A, CD69, B, HLA-DR and C, margination. Note: T-cell markers (percentages of CD69+ and PD1+ in CD4 and CD8 T cells) and cell counts of CD4 and CD8, in peripheral blood, were measured at baseline, 30 minutes after polatuzumab vedotin dosing and 2 hours after mosunetuzumab at multiple time points. Blood samples from patients treated with 1/2/60/30 mg of mosun-pola, were evaluated for T cell activation and margination, at C1D1-PRE. (*n* = 88 and 93, respectively), C1D1-Pola (*n* = 79 and 77), C1D1-Mosun (after pola; n = 82 and 79), C1D15-PRE (*n* = 41 and 33), C1D15-Mosun (alone) (*n* = 37 and 27), C2D1-PRE (*n* = 76, and 76) and C2D1-Mosun (after pola; *n* = 77 and 75). Box plots represent changes from baseline at each time point in (**A**) percentages of CD69+CD4+ and CD69 + CD8+ T cells, (**B**) percentages of HLA-DR + CD4+ and HLA-DR + CD8+ T cells and (**C**) CD4 and CD8 cell counts (fold changes). Each patient is represented by a filled circle with the colors depicting the best overall responses. The median change in response at each time point is represented by the line. P values represent pairwise two-sided Wilcox test against baseline (C1D1-PRE) (*, p ≤ 0.05; **, p ≤ 0.01; ***, p ≤ 0.001; ****, p ≤ 0.0001). Comparisons with small effect size (r < 0.3) or P > 0.05 are depicted as ns. Box plots show median and quartiles, and whiskers depict the highest and lowest values that are within 1.5 times of inter-quartile range from the upper and lower quartile values. C, cycle; CR, complete response; D, day; mosun, mosunetuzumab; - ns, not-significant (p > 0.05); ORR, objective response rate; PD, progressive disease; PR, partial response; PRE, pre-treatment; pola, polatuzumab vedotin; SD, stable disease.

T cell activation, using the early activation marker CD69, was not observed after polatuzumab vedotin administration alone. However, percentages of both CD69^+^CD4^+^ and CD69^+^CD8^+^ T cells were elevated 2 h after mosunetuzumab administration at the initial step-up dose (C1D1) and at the target dose on D15 (Extended Data Fig. 5a). Increases in HLA-DR^+^ T cells were observed after mosunetuzumab administration on C1D15 and C2D1 (Extended Data Fig. 5b). Consistent with the pharmacodynamic effects of mosunetuzumab on T cells, margination was not observed with polatuzumab vedotin alone but was seen with subsequent doses of mosunetuzumab. Overall, prior exposure with polatuzumab vedotin did not negatively influence the previously observed pharmacodynamic effects on T cells seen with mosunetuzumab monotherapy (Extended Data Fig. 5c)^32^. There was no clear association of these pharmacodynamic changes with clinical response.

**Extended Data Fig. 6:**
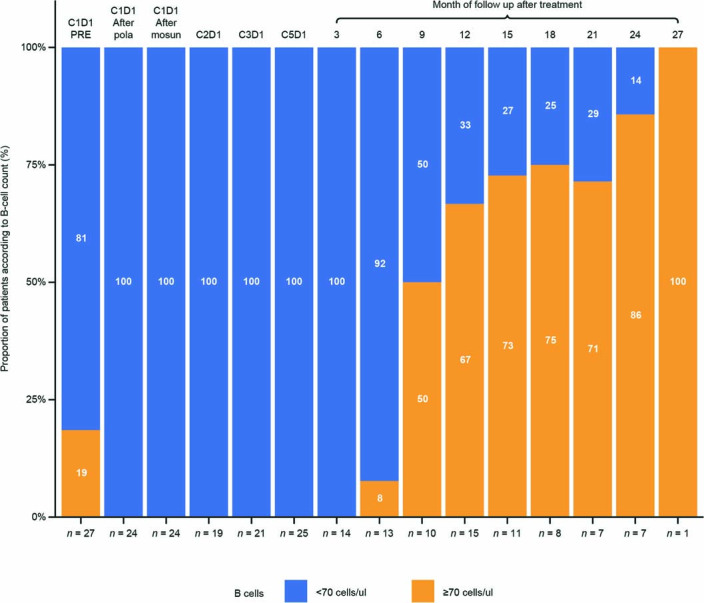
CD19 B-cell recovery over time in patients who achieved a complete response (n = 27) in the dose-escalation and dose-expansion cohorts. Note: The proportion of patients with low (<70 cells/µL) or normal (≥70 cells/µL) CD19+ B cell counts at specified timepoints during treatment and follow-up visits are indicated. C1D1-PRE, C2D1, C3D1 and C5D1 represent timepoints before treatment dosing. C1D1 after polatuzumab vedotin represents the timepoint 30 minutes after polatuzumab vedotin infusion. C1D1 after mosunetuzumab represents the timepoint 2 hours after subsequent mosunetuzumab treatment. The number of patients observed at each time point is depicted at the bottom of each bar. Months at follow-up were calculated from the end of initial treatment ±45 days. The number of B-cell count assessments varied for each patient across the study period.

B cell recovery was assessed given that the mosun-pola regimen targets two distinct B cell lineage markers. B cell counts were evaluated in patients who achieved a complete response. Patients with partial response or stable disease were excluded from the analysis, as circulating tumor B cells and loss of long-term follow-up at time of progression could confound interpretation. Twenty-seven patients who achieved a complete response and had at least one B cell measurement at baseline, during treatment and during the follow-up period were included in this subset analysis. The median follow-up was 25 months (range, 13.5–35.9) for these 27 patients. Median time to B cell recovery, defined as CD19^+^ cells ≥70 cells per microliter (cells/µl), was 12.4 months (95% CI: 11.9–NE) from completion of C8 (13/27 patients) (Extended Data Fig. 6).

## Discussion

Primary analysis results from this phase 1b/2 dose-escalation and dose-expansion study demonstrated that mosun-pola is an effective therapy with durable responses and a manageable safety profile in patients with R/R LBCL. The MTD was not reached, and the RP2D of mosunetuzumab was established as 1/2/60/30 mg in combination with polatuzumab vedotin.

Patients with R/R LBCL in the dose-expansion cohort treated at the RP2D were observed to have responses of 59.2%, including complete response in 45.9% of patients. With a follow-up of almost 2 years, median PFS was 11.4 months, and median DoCR was not reached. Patients who had received prior CAR-T cell therapy and other high-risk subpopulations (DH/THL, HGBCL and primary refractory disease) demonstrated promising efficacy, which is clinically meaningful given the aggressive nature of the disease.

Although comparing across different patient populations and small sample sizes, previous studies showed that single-agent mosunetuzumab achieved a complete response rate of 24%^20^, whereas polatuzumab vedotin achieved a complete response rate of 13%^21^. The complete response rate (45.9%) suggests a synergistic relationship for the mosun-pola combination.

The overall safety profile of mosun-pola was consistent between the overall safety cohort and the dose-expansion cohort treated at the RP2D; was comparable to those of the individual agents in patients with R/R B-NHL^20,21^; and did not identify new safety signals. The observed safety profile supports the outpatient use of this regimen. Treatment-related AEs leading to mosun-pola discontinuation were reported in 10% of the patient population. Multiple features of this regimen, including mosunetuzumab C1 step-up dosing and the combination with polatuzumab vedotin on D1 to hypothetically reduce tumor burden, effectively mitigated CRS. CRS occurred in 16.7% of patients in the overall population and was limited to C1, with only 2.5% grade 3 events and no grade 4 events. The overall CRS incidence was lower than that previously reported with mosunetuzumab monotherapy^20^. All CRS events resolved with low utilization of treatments necessitating intensive medical care. Similar to other bispecific antibodies^12,33^ and in contrast to CAR-T cell therapies^34–36^, the incidence of potential ICANS was low at 5%, with the majority being grade 1 events. Although treatment-related tumor flare events were observed, the incidence was low (2.5%). These safety results are in line with those reported in the mosunetuzumab R/R DLBCL monotherapy trial^20^. Rates of treatment-related neuropathy (29.2%) were comparable with previous reports of mosunetuzumab^37^ and polatuzumab vedotin monotherapies^21^. Although neutropenia was the most common AE (35.0%; grade ≥3, 25.0%), low rates of concurrent serious infection and no febrile neutropenia were observed.

The addition of polatuzumab vedotin to mosunetuzumab had minimal impact on T cell activation by mosunetuzumab, as measured by CD69^+^ T cells or T cell margination. There was no clinical correlation between these pharmacodynamic markers and clinical outcome. The combination therapy also demonstrated a median time to B cell recovery of 12 months after completion of treatment.

Previous studies exploring regimens in the second-line or later settings reported a best ORR of 62% and a median PFS of 5.4 months for polatuzumab plus bendamustine and rituximab^16^ and an ORR of 60% and median PFS of 11.6 months for tafasitamab plus lenalidomide (tafa-len)^15^. Notably, the current study enrolled a higher proportion of primary refractory patients (57.5% versus 19.0%) and post-CAR-T cell therapy patients (35% versus 0%) compared to the tafa-len population in the L-MIND study^15^. A real-world study of tafa-len across 11 US institutions with 178 patients noted an ORR of 31% and a median PFS of 1.9 months^38^. Clinical trials of CAR-T cell therapy in the second-line and third-line settings, across both transplant-eligible and transplant-ineligible patient groups, have reported response rates ranging from 45% to 92%, median PFS ranging from 3 months to 14.7 months and 1-year PFS rates of approximately 30–50%^5–7,9,34,39–42^. However, 78–95% of patients undergoing CAR-T cell therapy experience grade 3 or higher AEs. For example, all-grade CRS events were observed in 38–93% of patients^5,6,39,41^. In studies of CAR-T cell therapies intended for transplant-ineligible patients, prolonged grade 3 or higher cytopenia events were observed in more than 30% of patients^7,9^. Many feasibility challenges to CAR-T cell delivery, including limited manufacturing capacity, referral to specialist centers, gaps in infrastructure and logistic hurdles for physicians and patients, have led to discussions of prioritizing or developing systems to allocate resources to accommodate the medical demand^43–46^. More recently, other bispecific antibody monotherapies have been approved, generating responses of 52–63% in patients with R/R LBCL, median PFS ranging between 4.4 months and 4.9 months and median DoCR of 12 months to not reached with a follow-up of approximately 1 year^12,13^.

In the current study, cell of origin (COO) was assessed by investigators. A numerically greater response was observed in patients with R/R non-germinal center B cell-like (GCB) LBCL compared to those with R/R GCB-LBCL. However, there remains a benefit in patients with GCB-LBCL; the ORR and complete response rates were 55% and 36%, respectively. Comparatively, complete remissions with single-agent mosunetuzumab in third-line and later R/R LBCL were 24% in GCB and 28% in non-GCB^20^. Other regimens in R/R LBCL, such as tafa-len, have also demonstrated numerically higher overall responses in non-GCB than GCB (68.5% versus 42.9%)^47^. Although we recognize the ease and simplicity of COO, other molecular classifiers, including LymphGen, DZsig and the Chapuy classifier, may serve as a more nuanced prognostic and predictive biomarkers in LBCL^48–51^. Biomarker analyses of these molecular subgroups in the POLARIX study may provide further information of predictive strategies other than COO. Additionally, although polatuzumab vedotin may have a heightened sensitivity based on molecular classification of LBCL, mosunetuzumab is an immune-based targeted therapy with a mechanism of action that is likely independent of COO. Our subgroup analysis is based on a small sample size, and additional analysis is needed to determine whether the combination of mosun-pola potentially transcends COO.

The migration of polatuzumab vedotin to the front-line setting identifies a need to understand the ability to retreat with polatuzumab vedotin. Similar to repeated CD20 targeting, polatuzumab vedotin retreatment may be possible if CD79B expression is retained and polatuzumab vedotin–associated toxicities are limited. In this study, two patients were retreated with mosun-pola while still on study, and five patients were retreated with polatuzumab vedotin as a component of next anti-lymphoma therapy. However, a larger cohort of patients is necessary to further guide clinical practice. In patients treated with polatuzumab vedotin–based therapy in the front-line setting, mosun-pola may be suited for patients who are not refractory to polatuzumab vedotin and have no substantial polatuzumab vedotin–specific contraindications or persistent AEs.

The rapidly evolving treatment landscape for R/R LBCL offers patients multiple options. In the second-line transplant-ineligible space, treatment options may include tafa-len, CAR-T cell therapy and traditional chemotherapy, with selection dependent upon potential for cure. Acknowledging differences in study design, patient populations and caveats of cross-trial comparisons, the clinical outcomes and safety profile of mosun-pola are encouraging. The benefits of mosun-pola lie in its relatively high activity, durable responses and ease of administration as a fixed-duration regimen applicable to community oncology practices. Additionally, mosun-pola is distinguished as a bispecific antibody combination with a non-traditional chemotherapy partner. Despite transplant eligibility being an exclusion criterion, some patients were able to receive ASCT, allogeneic SCT and CAR-T cell therapy after mosun-pola, suggesting that disease status and/or disease-associated comorbidities were contributory features for transplant ineligibility at the time of enrollment. In particular, some patients were resistant to salvage therapy yet achieved a response to mosun-pola, which facilitated subsequent eligibility for consolidation with ASCT, allogeneic SCT or CAR-T cell therapy.

Our study is limited by its single-arm design and the potential for selection bias. Furthermore, although the responses in high-risk subgroups are promising, the study is underpowered to assess efficacy in these subgroups. Additional translational studies are needed to fully understand mechanisms of resistance to this combination regimen. CD20 loss has been observed as a mechanism of acquired resistance to treatment regimens targeting CD20, including mosunetuzumab^52–55^. Data assessing CD79B levels are more limited; however, measurements by immunohistochemistry and RNA-based gene expression have demonstrated results consistent with other lineage markers that exhibit generally high, consistent expression patterns in pre-dose biopsy specimens from patients with R/R DLBCL^16^. Limitations of the study also include the lack of patients with prior polatuzumab vedotin exposure and the lack of systematic polatuzumab vedotin retreatment data. Additional data are needed in patients with prior polatuzumab vedotin exposure, particularly in the non-GCB subgroup. Nonetheless, the current results led to the development of the ongoing global, randomized, open-label phase 3 study (SUNMO: NCT05171647), which is evaluating the efficacy and safety of mosun-pola in patients with R/R aggressive B-NHL who are ineligible for ASCT, a study that permits prior treatment with polatuzumab vedotin^56^.

In conclusion, this phase 1b/2 study demonstrated that mosun-pola, a combination regimen targeting two biologically relevant B cell targets using a T-cell-engaging bispecific antibody and an antibody–drug conjugate, induced durable responses in patients with R/R LBCL, including patients with poor clinical and pathologic prognostic features, such as relapse after CAR-T cell therapy. Despite enrolling patients with poor prognostic features, the regimen has a safety profile that is manageable in an outpatient setting. Based on the observed efficacy and safety profile, mosun-pola holds promise for patients with aggressive R/R LBCL ineligible for ASCT or aggressive intense immunochemotherapy.

## Methods

### Study design and participants

NCT03671018 is an ongoing, open-label, multicenter, phase 1b dose-escalation and phase 2 single-arm dose-expansion study of mosunetuzumab combined with polatuzumab vedotin (mosun-pola) in B cell NHL. Here we present results of dose escalation in patients with R/R NHL and single-arm expansion in second-line or later LBCL.

In summary, eligible patients were aged ≥18 years with an Eastern Cooperative Oncology Group (ECOG) performance status of 0–2, life expectancy of at least 12 weeks and histologically confirmed LBCL. Histologically confirmed LBCL was defined as de novo LBCL (that is, DLBCL), HGBCL (that is, fluorescence in situ hybridization–verified *MYC/BCL2*, *MYC/BCL6* or *MYC/BCL2/BCL6* translocation or site-noted HGBCL without translocations), grade 1–3a FL, transformed FL or grade 3b FL for the phase 1b dose-escalation and phase 2 dose-expansion cohorts. The phase 1b dose-escalation cohort also included patients with histologically confirmed grade 1–3a FL, who were included in safety analyses but excluded from efficacy analyses in this manuscript. Patients had relapsed disease or disease that was refractory to at least one previous line of treatment, including an anti-CD20 therapy. Early relapse was defined as relapse within 12 months of first prior therapy. Refractory disease was defined as a lack of response or progression within 6 months of last treatment.

Patients could not have current eligibility for ASCT; eligibility was decided at the physician’s discretion. Although no specific criteria were in place to determine whether a patient was eligible for transplant, criteria for transplant ineligibility were captured, including age, performance status, comorbidities and insufficient response to salvage therapy. Patients needed to meet only one of these criteria to be ineligible for ASCT. Further details of key inclusion and exclusion criteria are provided below.

Key Inclusion criteria:

- Signed informed consent form
- Age ≥18 years at time of signing informed consent form
- Able to comply with the study protocol and procedures in the investigator’s judgment
- ECOG PS of 0, 1 or 2; life expectancy of at least 12 weeks
- Histologically confirmed FL or DLBCL from the 2016 World Health Organization classification diagnoses of lymphoid neoplasms that has either relapsed or become refractory to a prior regimen
- Measurable disease, defined as at least one bi-dimensionally measurable nodal lesion, defined as larger than 1.5 cm in its longest dimension, or at least one bi-dimensionally measurable extranodal lesion, defined as larger than 1.0 cm in its longest dimension
- Pathology report for the initial histopathology diagnosis and the most recent histopathology diagnosis before study entry must be provided
  - Patients with transformed FL must also provide the pathology report at the time of disease transformation.
  - The results of all tests conducted on the tissue at initial diagnosis, including, but not limited to, tests assessing COO, *BCL2* and *MYC* abnormalities, should be provided if done.
- Agreement to provide tumor samples as follows:
  - Undergo biopsy from a safely accessible site per investigator determination
  - Patients who are unable to undergo biopsy procedures may be eligible for study enrollment if archival tumor tissue samples (paraffin blocks or at least 20 unstained slides), in place of a fresh biopsy, can be sent to the sponsor.
  - Bone marrow biopsy and aspirate (if applicable)
  - AEs from prior anti-cancer therapy resolved to grade ≤1

- Laboratory findings as follows:
  - Adequate liver function: aspartate aminotransferase (AST) and alanine transaminase (ALT) ≤2.5× upper limit of normal (ULN) and total bilirubin ≤1.5× ULN. Patients with a documented history of Gilbert syndrome and in whom total bilirubin elevations are accompanied by elevated indirect bilirubin are eligible.
  - Adequate hematologic function: platelet count ≥75,000/mm^3^ without transfusion within 14 days before first dose of study treatment, ANC ≥1,000/mm^3^ and total hemoglobin ≥9 g/dl without transfusion within 21 days before first dose of study treatment
  - Patients with extensive marrow involvement of NHL and/or disease-related cytopenias (for example, immune thrombocytopenia) may be enrolled if: platelet count is ≥50,000/mm^3^ without transfusion within 14 days, absolute neutrophil count ≥500/mm^3^ and any hemoglobin but without transfusion within 7 days.
  - International normalized ratio ≤1.5× ULN in the absence of therapeutic anticoagulation
  - Partial thromboplastin time or activated partial thromboplastin time ≤1.5× ULN in the absence of lupus anticoagulant or therapeutic anticoagulation
  - Estimated creatinine hydrochloride ≥50 ml/min by the Cockroft–Gault method or other institutional standard methods (for example, based on nuclear medicine renal scan)
  - Negative HIV test at screening. Patients with a positive HIV test at screening are also eligible provided they are stable on anti-retroviral therapy, have a CD4 count ≥200 per microliter and have an undetectable viral load.
- Women of childbearing potential must agree to remain abstinent or use contraceptive measures and agree to refrain from donating eggs, as defined below:
  - Women must remain abstinent or use contraceptive methods with a failure rate of less than 1% per year during the treatment period and for 3 months after the final dose of mosunetuzumab and for 9 months after the final dose of polatuzumab vedotin. Women must refrain from donating eggs during this same period.

- For men: agreement to remain abstinent or use a condom and agree to refrain from donating sperm, as defined below:
  - With female partners of childbearing potential or pregnant female partners, men must remain abstinent or use a condom during the treatment period and for 6 months after the final dose of polatuzumab vedotin, to avoid exposing the embryo. Men must refrain from donating sperm during this same period.
  Key exclusion criteria:
- Inability to comply with protocol-mandated hospitalization and activity restrictions
- Pregnant or breastfeeding or intending to become pregnant during the study or within 3 months after the final dose of mosunetuzumab or within 9 months after the final dose of polatuzumab vedotin
- Prior treatment with mosunetuzumab or other CD20-directed bispecific antibodies
- Prior treatment with polatuzumab vedotin
- Current grade >1 peripheral neuropathy
- Prior use of any monoclonal antibody, radioimmunoconjugate or antibody–drug conjugate within 4 weeks before first dose of study treatment
- Treatment with any chemotherapeutic agent or treatment with any other anti-cancer agent (investigational or otherwise) within 4 weeks or five half-lives of the drug, whichever is shorter, before first dose of study treatment
- Treatment with radiotherapy within 2 weeks before first dose of study treatment
  - If patients have received radiotherapy within 4 weeks before the first study treatment administration, patients must have at least one measurable lesion outside of the radiation field. Patients who have only one measurable lesion that was previously irradiated but subsequently progressed are eligible.
- ASCT within 100 days before first study treatment administration
- Prior treatment with CAR-T cell therapy within 30 days before first study treatment administration
- Current eligibility for ASCT in patients with R/R DLBCL, R/R transformed FL or R/R grade 3b FL
- Prior allogeneic SCT
- Prior solid organ transplant
- Known or suspected history of hemophagocytic lymphohistiocytosis
- History of confirmed progressive multifocal leukoencephalopathy
- History of severe allergic or anaphylactic reactions to monoclonal antibody therapy (or recombinant antibody-related fusion proteins)
- History of other malignancy that could affect compliance with the protocol or interpretation of results
  - Patients with a history of curatively treated basal or squamous cell carcinoma of the skin or in situ carcinoma of the cervix are allowed.
  - Patients with a malignancy that has been treated with curative intent will also be allowed if the malignancy has been in remission without treatment for ≥2 years before first study treatment administration.

- Current or past history of central nervous system (CNS) lymphoma
- Current or past history of CNS disease, such as stroke, epilepsy, CNS vasculitis or neurodegenerative disease
  - Patients with a history of stroke who have not experienced a stroke or transient ischemic attack in the past 2 years and have no residual neurologic deficits as judged by the investigator are allowed.
  - Patients with a history of epilepsy who have had no seizures in the past 2 years while not receiving any anti-epileptic medications are allowed in the expansion cohorts only.

- Substantial cardiovascular disease, such as New York Heart Association class III or IV cardiac disease, myocardial infarction within the last 6 months, unstable arrhythmias or unstable angina
- Substantial active pulmonary disease (for example, bronchospasm and/or obstructive pulmonary disease)
- Known active bacterial, viral, fungal, mycobacterial, parasitic or other infection (excluding fungal infections of nail beds) at study enrollment or any major episode of infection requiring treatment with intravenous antibiotics or hospitalization (relating to the completion of the course of antibiotics) within 4 weeks before first study treatment administration
- Known or suspected chronic active Epstein–Barr virus infection
- Recent major surgery within 4 weeks before first study treatment administration
  - Protocol-mandated procedures (for example, tumor biopsies and bone marrow biopsies) are permitted.
- Positive test results for chronic hepatitis B infection
  - Patients with occult or prior hepatitis B infection (defined as positive total hepatitis B core antibody and negative HBsAg) may be included if hepatitis B virus (HBV) DNA is undetectable at the time of screening. These patients must be willing to undergo monthly DNA testing and appropriate antiviral therapy as indicated.
- Acute or chronic hepatitis C virus (HCV) infection
  - Patients who are positive for HCV antibody must be negative for HCV by polymerase chain reaction (PCR) to be eligible for study participation.

- Administration of a live, attenuated vaccine within 4 weeks before first dose of study treatment administration or anticipation that such a live, attenuated vaccine will be required during the study
  - Patients must not receive live, attenuated vaccines while receiving study treatment and after the last dose until B cell recovery to the normal ranges. Killed vaccines or toxoids should be given at least 4 weeks before the first dose of study treatment to allow development of sufficient immunity.
  - Inactivated influenza vaccination should be given during local influenza season only.
  - Investigators should review the vaccination status of potential study patients being considered for this study and follow the US Centers for Disease Control and Prevention guidelines for adult vaccination with any other non-live vaccines intended to prevent infectious diseases before study.
- History of autoimmune disease, including, but not limited to, myasthenia gravis, myositis, autoimmune hepatitis, systemic lupus erythematosus, rheumatoid arthritis, inflammatory bowel disease, vascular thrombosis associated with antiphospholipid syndrome, Wegener granulomatosis, Sjögren syndrome, Guillain–Barré syndrome, multiple sclerosis, vasculitis or glomerulonephritis
- Received systemic immunosuppressive medications (including, but not limited to, cyclophosphamide, azathioprine, methotrexate, thalidomide and anti-tumor necrosis factor agents) with the exception of corticosteroid treatment ≤10 mg per day prednisone or equivalent within 2 weeks before first dose of study treatment
  - The use of inhaled corticosteroids is permitted. The use of mineralocorticoids for management of orthostatic hypotension is permitted.
  - The use of physiologic doses of corticosteroids for management of adrenal insufficiency is permitted.
  - Patients who received acute, low-dose, systemic immunosuppressant medications (for example, single dose of dexamethasone for nausea or B symptoms) may be enrolled.

- Clinically substantial history of liver disease, including viral or other hepatitis, current alcohol abuse or cirrhosis
- Any serious medical condition or abnormality in clinical laboratory tests that, in the INV’s judgment, precludes the patient’s safe participation in and completion of the study or which could affect compliance with the protocol or interpretation of results

The overall safety population included patients with histologically confirmed LBCL (as defined as de novo LBCL, HGBCL, grade 3b FL and transformed FL) or grade 1–3a FL (*n* = 120). The efficacy analyses excluded the three patients with histologically confirmed grade 1–3a FL (*n* = 117).

In the phase 1b dose-escalation (modified 3 + 3 design) cohort, intravenous mosunetuzumab was administered in 21-day cycles with C1 step-up dosing to mitigate for CRS—that is, 1 mg on C1D1, followed by 2 mg on C1D8 and then escalated to a target/loading dose of 9 mg, 20 mg, 40 mg or 60 mg given on C1D15 and D1 of C2+ (eight or 17 cycles depending on response). In the highest dose group (1/2/60/30 mg), mosunetuzumab was administered at 1 mg on C1D1, followed by 2 mg on C1D8, 60 mg on C1D15 and C2D1 and then 30 mg on D1 of C3+, and this was determined to be the RP2D. An intravenous infusion of polatuzumab vedotin 1.8 mg/kg was administered prior, starting on D1 of each 21-day cycle for six cycles. Hospitalization was mandatory for all patients on D1 of C1 and C2 in the dose-escalation cohort.

In the phase 2 dose-expansion cohort, intravenous mosunetuzumab was administered at the RP2D (1/2/60/30 mg) in 21-day cycles. An intravenous infusion of polatuzumab vedotin 1.8 mg/kg was administered on D1 of each 21-day cycle for six cycles. Hospitalization was not mandatory during treatment in the dose-expansion cohort.

In both phase 1b and 2 cohorts, intravenous corticosteroid premedication (dexamethasone 20 mg or methylprednisolone 80 mg) was administered 1 h before each mosunetuzumab dose during C1 and C2 and was optional from C3 onwards, unless the patient experienced a CRS event in the prior cycle. Certain premedication, such as antipyretics and antihistamines, were allowed during the study but were not required as part of the protocol.

Phase 1 study objectives were to evaluate the safety, tolerability and pharmacokinetics of mosun-pola as well as preliminary assessment of the anti-tumor activity of the combination regimen in patients with R/R LBCL. Phase 2 study objectives were to evaluate the efficacy, safety and pharmacokinetics of mosun-pola in patients with R/R LBCL.

All patients provided written informed consent. The study was approved by institutional review boards or ethics committees at each center (WCG Clinical, Inc.; NYU School of Medicine, Office of Science and Research Institutional Review Board; University of Miami, Human Subject Research Office; Wayne State University, IRB Administration Office; Advarra; Lifespan Research Protection, Office of Research; Quebec Integrated Health and Social Services, University Network for West-Central Montreal; University of Saskatchewan Biomedical Research Ethics Board, Royal University Hospital; and Penn State Health Milton S. Hershey Medical Center, Institutional Review Board Human Subjects Protection Office). The trial was conducted in accordance with the Declaration of Helsinki, International Conference on Harmonization Guidelines for Good Clinical Practice and applicable laws and regulations. The study protocol is available as part of the Supplementary Information.

### Recruitment and blinding

This trial was open label with no blinding. Patients were recruited in 15 sites across two countries (the USA and Canada) among patients of the site or among patients referred from other hospitals from September 2018 to February 2022. Sites were at either academic or community hospitals. The phase 1 portion of the clinical trial was enrolled based on slot availability; the study had established sites for the study that would screen patients at those locations to determine eligibility for the trial. The phase 2 portion was an open enrollment to the active participating sites. To participate in this study and before any non-routine baseline or screening evaluation, investigators at each study site ensured that each patient was fully informed of the study and had signed a written informed consent. The patient’s eligibility was evaluated during the screening period before enrollment. No bias emerging from recruitment is expected. Patients were enrolled irrespective of gender, which was self-reported by the patient. Randomization was not performed, as we report results from the single-arm dose-expansion cohort.

### Biomarker assays

Peripheral blood samples were collected for central flow cytometry analysis of selected B cell and T cell markers. Whole blood samples were collected in Becton Dickinson (BD) Vacutainer tubes containing sodium heparin. For cell labeling, samples were labeled with antibodies for 30 min in the dark at ambient temperature. Red blood cells were then lysed, using BioLegend RBC Lysis Buffer for 15 min at ambient temperature. After centrifugation, samples were washed with BD Stain Buffer and resuspended in 1% formalin fixative. Assay panel tubes were stored at 4 °C and acquired within 4 h of preparation.

**Extended Data Fig. 7:**
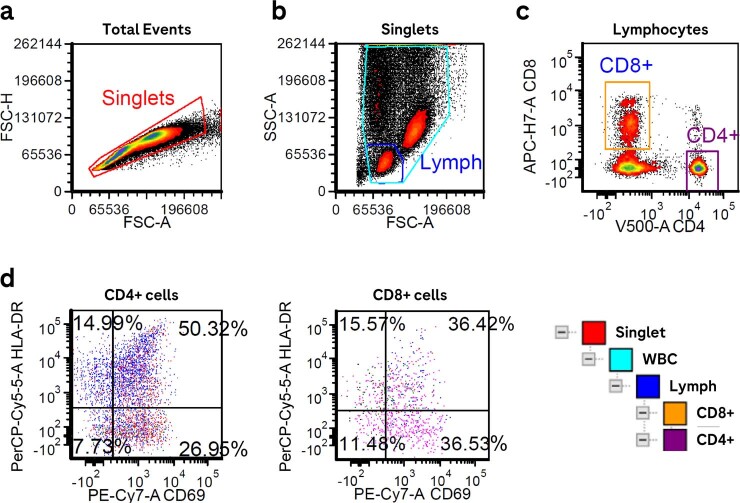
Schematic of the gating strategy for analysis of flow cytometry data. Note: **a**) Doublets were excluded with the Singlets gate based on FSC-H and FSC-A; **b**) set a debris-free white blood cell (WBC) gate based on an FSC-A by SSC-A, also a sub-gate on SSC-Alo and FSC-Amid for Lymphocytes; **c**) On the Lymphocytes, CD4 positive cells and CD8 positive cells were gated; **d**) Examine expression of HLA-DR and CD69 on the gated CD4+ cells or CD8+ cells. Boundaries between positive and negative staining HLA-DR and CD69 were based on backbone only (CD4 and CD8) stained control.

CD4 and CD8 concentrations (cells/µl) were calculated from the absolute leukocyte counts from a CBC blood sample collected at the same time using the following formula: CD4 or CD8 (cells/µl) = white blood cell concentration (cells/µl) × [(CD4 or CD8 event counts)/(white blood cell event counts)]. Extended Data Fig. 7 illustrates a schematic example of the gating strategy (accession ID: BC1692812, C1D1, pre-dose). The cocktail of antibodies consisted of CD4-BV510 (SK3, BD, 562970), HLA-DR PerCP-Cy5.5 (G46-6, BD, 560652), CD69 PE-Cy7 (FN50, BD, 557745) and CD8 APC-H7 (SK-1, BD, 561423).

Samples were acquired on BD FACSCanto II flow cytometers (BD Biosciences, designated at ILS-Dublin Canto D, s/n V33896201828) using FACSDiva software (BD Biosciences, version 6.1.3). CD19^+^ B cell counts were quantified by a standard TBNK (lymphocyte immunotyping) flow cytometry panel at LabCorp. T cell markers were measured with a validated custom panel at ICON (ICON plc). B cell counts were evaluated in patients who achieved a complete response. CD19^+^ cells ≥70 cells/µl was considered the lower level of normal for B cell recovery^57^. A time-to-event analysis was performed to assess time to B cell recovery. T cell activation was measured by flow cytometry to assess the impact of polatuzumab vedotin on the pharmacodynamics of mosunetuzumab.

### Assessments

Interim response assessments were obtained between C4D15 and C4D21 and at primary response assessment (PRA) at the end of C8. Patients with a complete response at PRA completed treatment at C8, whereas those with a partial response or stable disease at PRA continued mosunetuzumab monotherapy for a total of 17 cycles, unless progressive disease or unacceptable toxicity occurred. The number of cycles of polatuzumab vedotin was limited to six in total, irrespective of response. Retreatment with mosunetuzumab monotherapy or mosun-pola was permitted in patients who experienced progressive disease after an initial complete response.

PET and diagnostic-quality CT scans were required at screening, at the interim response assessment and at the PRA visit. During follow-up, CT scans with or without PET scans were used. Before a metabolic complete response was achieved, it was recommended that PET scans should continue in conjunction with diagnostic-quality CT scans. Additionally, if progressive disease or relapse was suspected before the PRA, both PET and diagnostic-quality CT scans should be performed for tumor assessment. Lugano 2014 criteria were used to assess overall response to study treatment^30^.

When determining best response, the PET scan result was used unless it was missing or not evaluable. There were five patients in whom this was the case, so the CT scan result was used instead.

### Study endpoints during the phase 1b dose escalation

The primary objectives in the dose-escalation cohort were to evaluate safety and tolerability and to determine any DLTs, the MTD and the RP2D of mosunetuzumab in combination with polatuzumab vedotin 1.8 mg/kg. The secondary objectives were anti-tumor activity, determined by measuring the complete response rate at the time of PRA based on PET-CT; best ORR (complete response or partial response at any time) on study, based on PET and/or CT scan; and DoR, defined as the time from the first occurrence of a documented ORR to progressive disease or relapse or death from any cause, whichever occurred first. Response was determined by the INV using Lugano 2014 criteria^41^. AEs were reported using National Cancer Institute Common Terminology Criteria for Adverse Events (NCI CTCAE) version 5.0. CRS events were graded according to American Society for Transplantation and Cellular Therapy (ASTCT) criteria^58^.

### Study endpoints during the phase 2 dose expansion

The primary efficacy endpoint in the dose-expansion cohort was best ORR based on PET-CT and/or CT scan by independent review committee (IRC) using Lugano 2014 criteria^30^. Secondary efficacy endpoints were also assessed using Lugano 2014 criteria^30^ and included best ORR on study based on PET-CT and/or CT scan determined by the INV; best complete response rate and complete response rate at PRA based on PET-CT and/or CT scan determined by the INV and the IRC; DoR determined by the INV and the IRC; PFS, defined as the time from first study treatment to the first occurrence of progressive disease or relapse or death from any cause, whichever occurred first, determined by the INV and the IRC; and overall survival, defined as the time from first study treatment to death from any cause. AEs were reported using NCI CTCAE version 5.0. CRS events were graded according to ASTCT criteria^58^.

### Statistical analysis

The sample size of the dose-escalation cohort was based on dose-escalation rules, which was a modified 3 + 3 design. A minimum of three patients were initially enrolled in each cohort to evaluate DLTs. If none of the first three DLT-evaluable patients experienced a DLT, then enrollment of the next cohort could proceed. If a patient experienced a DLT, then the cohort was expanded to six patients to be evaluated for additional DLTs. For the phase 2 dose-expansion cohort, a sample size of 100 patients was calculated to provide 99% power to detect a difference in ORR, with a two-sided significance level of 5%. The primary endpoint of ORR was to be assessed using an exact binomial test at a one-sided 2.5% level of significance, rejecting the null hypothesis of ORR 42%^31^. Complete response rates were estimated along with Clopper–Pearson exact 95% CIs. For DoR and PFS, Kaplan–Meier methods were used to estimate the medians and event-free rates at 12 months and 24 months. The Brookmeyer–Crowley method was used to calculate 95% CIs for the medians, and Greenwood’s formula was used to calculate standard errors and 95% CIs for PFS. FACSDiva software (BD Biosciences, version 6.1.3), FCS Express (DeNovo, version 4, Clinical Edition) and SAS version 9.4 were used for data analysis.

An internal monitoring committee (IMC) gave recommendations for study conduct, based on trial safety data, to ensure enhanced patient safety during study treatment. The IMC consisted of a Medical Monitor chair, who was not associated with the study, and representatives from Clinical Science, Safety Science and Biostatistics, who were all external to the study team.

### Reporting summary

Further information on research design is available in the Nature Portfolio Reporting Summary linked to this article.

## Online content

Any methods, additional references, Nature Portfolio reporting summaries, source data, extended data, supplementary information, acknowledgements, peer review information; details of author contributions and competing interests; and statements of data and code availability are available at 10.1038/s41591-023-02726-5.

### Supplementary information

Supplementary Information Supplementary redacted protocol

Reporting Summary

## Figures and Tables

**Extended Data Table 1:**
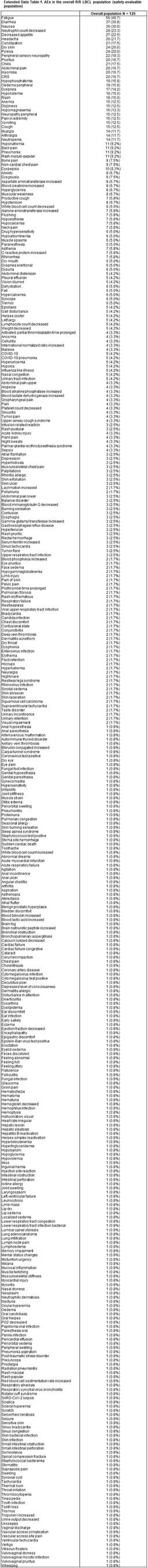
AEs in the overall R/R LBCL population (safety-evaluable population)

**Extended Data Table 2:**
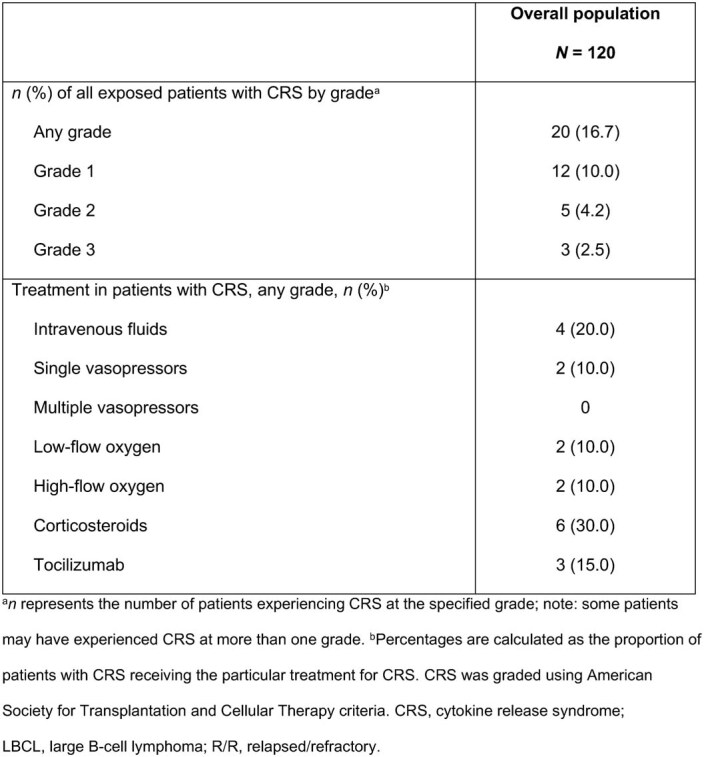
CRS management in the overall R/R LBCL population (safety evaluable population)

^a^*n* represents the number of patients experiencing CRS at the specified grade. Note: some patients may have experienced CRS at more than one grade.

^b^Percentages were calculated as the proportion of patients with CRS receiving the particular treatment for CRS. CRS was graded using ASTCT criteria.

## Data Availability

The sponsor was involved in the design and conduct of the study and in the collection, management, analysis and interpretation of the data. All authors had full access to the data in the study. Qualified researchers may request access to individual patient-level data through the clinical study data request platform (https://vivli.org/). Further details on Roche’s criteria for eligible studies are available at https://vivli.org/members/ourmembers/. For further details on Roche’s Global Policy on the Sharing of Clinical Information and how to request access to related clinical study documents, see https://assets.roche.com/f/176343/x/5590acbc9f/roche-global-policy-on-sharing-of-clinical-study-information.pdf.
