# Supplementary material for: Mosunetuzumab with polatuzumab vedotin in relapsed or refractory aggressive large B cell lymphoma: a phase 1b/2 trial
Source: Nat Med. 2023 Dec 10;30(1):229–39. doi: 10.1038/s41591-023-02726-5 (PMC10803244; doi:10.1038/s41591-023-02726-5)
Supplement: Supplementary file 1 — Supplementary redacted protocol [file 41591_2023_2726_MOESM1_ESM.pdf]

nature medicine

Article

https://doi.org/10.1038/s41591-023-02726-5

Mosunetuzumab with polatuzumab vedotin in relapsed or refractory aggressive large B cell lymphoma: a phase 1b/2 trial

In the format provided by the authors and unedited

PROTOCOL

**TITLE:**	**AN OPEN-LABEL, MULTICENTER, PHASE IB/II TRIAL EVALUATING THE SAFETY, TOLERABILITY, PHALRMACOKINETICS, AND EFFICACY OF MOSUNETUZUMAB (BTCT4465A) IN COMBINATION WITH POLATUZUMAB VEDOTIN IN PATIENTS WITH B-CELL NON-HODGKIN LYMPHOMA**
**PROTOCOL NUMBER:**	GO40516
**VERSION NUMBER:**	8
**EUDRACT NUMBER:**	2018-001141-13
**IND NUMBER:**	120651
**NCT NUMBER**	NCT03671018
**TEST PRODUCTS:**	Mosunetuzumab (RO7030816; BTCT4465A)<br>Polatuzumab vedotin (RO5541077; DCDS4501S)
**MEDICAL MONITOR:**	[redacted], *Pharm.D.*
**SPONSOR:**	F. Hoffmann-La Roche Ltd
**APPROVAL DATE:**	See electronic signature and date stamp on the final page of this document.

CONFIDENTIAL

This clinical study is being sponsored globally by F. Hoffmann-La Roche Ltd of Basel, Switzerland. However, it may be implemented in individual countries by Roche's local affiliates, including Genentech, Inc. in the United States. The information contained in this document, especially any unpublished data, is the property of F. Hoffmann-La Roche Ltd (or under its control) and therefore is provided to you in confidence as an investigator, potential investigator, or consultant, for review by you, your staff, and an applicable Ethics Committee or Institutional Review Board. It is understood that this information will not be disclosed to others without written authorization from Roche except to the extent necessary to obtain informed consent from persons to whom the drug may be administered.

PROTOCOL HISTORY

Protocol		Associated Country-Specific Protocol		
Version	Date Final	Country	Version	Date Final
8	See electronic date stamp on the final page of this document.	—	—	—
7	13 July 2021	—	—	—
6	22 December 2020	—	—	—
5	17 July 2020	United States	5	3 March 2020
4	16 August 2019	Germany	4	23 October 2019
3	1 February 2019	—	—	—
2	23 October 2018	—	—	—
1	23 April 2018	—	—	—

PROTOCOL AMENDMENT, VERSION 8:
RATIONALE

Protocol GO40516 has been amended to add an interim futility analysis for the randomized expansion Phase II portion of the study (Arms L and M), to add the Cumulative Illness Rating Scale-Geriatric (CIRS-G) assessment, and to allow patients who have a positive HIV test at screening to enroll provided they fulfill specific criteria. Substantive changes to the protocol, along with a rationale for each change, are summarized below:

- The treatment for Relapsed or Refractory (R/R) diffuse large B cell lymphoma (DLBCL)/transformed follicular lymphoma (FL) section has been updated to include recent Phase III POLARIX study data (Section 1.1.1).
- The details regarding previously granted accelerated approval for idelalisib (Zydelig®), copanlisib (Aliqopa™), duvelisib (Copiktra®), and umbralisib (Ukoniq®) has been updated as idelalisib, duvelisib, and umbralisib were voluntary withdrawn by the sponsors due to lack of confirmation of clinical benefit and safety concerns (Section 1.1.2, Table 2 footnotes “d”, “e”, and “f”).
- The approval status of mosunetuzumab and polatuzumab vedotin has been updated (Section 1.2, Section 1.3, and Section 5.1).
- The Summary of Clinical Data with Mosunetuzumab section has been updated based on the current Mosunetuzumab Investigator’s Brochure, Version 8 dated April 2022 and current available data from mosunetuzumab Studies GO40516, GO29781, and GO40554 (Section 1.2.2, Tables 4, 5, and 6).
- The CIRS-G assessment has been added to further characterize the elderly population based on comorbidity parameters enrolled in the study (Table 7, Section 4.5.11, Section 6.4.3, Appendix 1 footnote “e”, and Appendix 16).

  Presence of lymphoma or hematologic deficiencies due to lymphoma will not be included in the score for the CIRS-G assessment, since all patients in the study have lymphoma at study entry (Section 4.5.11, Appendix 16).
- Arms L and M have been added to the exploratory biomarker objective to make a preliminary assessment of minimal residual disease (MRD) status following mosunetuzumab treatment in combination with polatuzumab vedotin (Table 7).
- The randomized phase in study schema has been corrected to show the correct histology, sample size, and formulation (Figure 1).
- The “another anti-cancer agent” has been replaced with “next anti-lymphoma treatment” throughout the protocol to improve clarity on specified treatment (Sections 3.1.1, 4.4.3, 5.3.1, 5.6, Appendix 1 footnote “i” and “dd”).
- The responsibilities of the investigator and the role of the Medical Monitor in determining patient management have been clarified (Section 3.1.2.2).
- An interim futility analysis has been added for the randomized expansion phase in order to determine if an increase of sample size is needed to increase the likelihood to detect a difference in response rates between mosunetuzumab plus polatuzumab

vedotin (Arm L) versus rituximab plus polatuzumab vedotin (Arm M). The enrollment in Arms L and M may expand to [redacted] patients total ([redacted] patients in each arm) based on interim futility analysis of randomized Phase II portion of the study (Sections 3.1.3.3, 3.3.7, 4.1 and 6.9.2).

- Additional clarification has been provided regarding the total number of permitted polatuzumab vedotin cycles for patients who receive treatment in Arm M and Arm M-crossover (Section 3.1.5.2).
- Language has been added that patients who have a positive HIV test at screening may be eligible if they meet specific criteria. The language “positive serologic test results for HIV infection” has been removed from the exclusion criteria. This change is to align with the current FDA guidance on cancer clinical trial eligibility criteria for patients with HIV, Hepatitis B Virus, or Hepatitis C Virus infections (Sections 4.1.1, 5.1.2.1 and Appendix 1 footnote “t”).
- Exclusion criteria has been amended to clarify that patients who received acute, low-dose, systemic immunosuppressant medications may be enrolled (Section 4.1.2).
- Language has been clarified regarding the use of commercial tocilizumab obtained locally if permitted by local regulations and if needed for emergency purposes to treat cytokine-release syndrome (Section 4.3.1.4).
- The clarification has been provided for the length of time patients will be observed after receiving mosunetuzumab SC doses (Section 4.3.2.1).
- The language regarding corticosteroid premedication for patients receiving mosunetuzumab SC with other clinical studies in the mosunetuzumab program has been aligned (Section 4.3.2.1).
- The permitted therapy section has been updated to allow intrathecal chemotherapy for CNS prophylaxis while on study (Section 4.4.1).
- SARS-CoV-2 has been replaced with coronavirus disease 2019 (COVID-19) as per definition and guidance on concomitant administration of COVID-19 vaccines with mosunetuzumab (Section 4.4.1.5)
- The clarification has been provided that peripheral blood smear and/or flow cytometry can be used in place of a bone marrow biopsy to confirm the presence of circulating lymphoma cells at screening (Section 4.5.6.2 and Appendix 1 footnote “ee”).
- Transplant eligibility status will not be performed at primary response assessment, end of treatment, and study discontinuation visits, since this is no longer an exploratory endpoint in previous protocol amendment (Section 4.5.7 and Appendix 1 footnote “f”).
- The clarification has been provided that on-treatment biopsy may be omitted if there is no lesion available to biopsy due to response to treatment or the on-treatment biopsy may cause additional safety risk(s) for the patient (Section 4.5.8 and Appendix 1 footnote “q”).

- The email address for withdrawal from the Research Biosample Repository after site closure has been corrected (Section 4.5.13.6).
- Guidance on how to assess a possible tumor flare event has been added (Section 5.1.2.1).
- Guidance for mosunetuzumab, polatuzumab vedotin, and rituximab dose delay in the setting of bilirubin $>$3.0 mg/dL has been updated (Table 16).
- The guidance for repeat step-up dosing has been updated if mosunetuzumab dose is delayed for 6 weeks or longer. For patients treated with mosunetuzumab IV, repeat the mosunetuzumab Cycle 1 step-up dosing, and if there was a $>$7 days dose delay in Cycle 1, repeat the mosunetuzumab at the highest dose. For patients treated with mosunetuzumab SC, repeat the 5 mg dose followed by the next planned dose on Day 8 if there was a dose delay for 6 weeks or longer; if there was a $>$7 days dose delay in Cycle 1, then the 5 mg dose would be repeated prior to resuming the planned Cycle 1 treatment schedule (Section 5.1.7.2 and Appendix 1).
- Emergency Medical Contacts for this study have changed and respective contact information has been updated (title pages, Protocol Amendment Acceptance Form, and Section 5.4.1).
- Language regarding allowing conversion to IV mosunetuzumab administration after developing unacceptable localized injection-site reactions following mosunetuzumab SC injection has been added (Section 5.1.7.7).
- Language regarding reporting of pregnancies has been clarified (Section 5.4.3.2).
- The clarification has been provided that the best objective response rate (ORR) between Arm L over Arm M would demonstrate clinical benefits of mosunetuzumab in combination with polatuzumab vedotin over rituximab in combination with polatuzumab vedotin (Section 6.4.1).
- Arms M and L have been added to the guidance on acceptable dosing windows (Appendix 1 footnote “c”).

Additional minor changes have been made to improve clarity and consistency. Substantive new information appears in italics. This amendment represents cumulative changes to the original protocol.

TABLE OF CONTENTS

PROTOCOL AMENDMENT ACCEPTANCE FORM 17

PROTOCOL SYNOPSIS 18

1. BACKGROUND 38

1.1 Background on B-Cell Lymphomas 38

1.1.1 Treatment for Relapsed or Refractory DLBCL/Transformed FL 39

1.1.2 Treatment for Relapsed or Refractory FL 43

1.1.3 Treatment for Relapsed or Refractory Mantle Cell Lymphoma 47

1.2 Background on Mosunetuzumab 48

1.2.1 Nonclinical Studies with Mosunetuzumab.............................. 49

1.2.2 Summary of Clinical Data with Mosunetuzumab 50

1.2.2.1 Safety 50

1.2.2.2 Efficacy... 54

1.2.2.3 Clinical Pharmacokinetics and Immunogenicity 54

1.3 Background on Polatuzumab Vedotin 55

1.4 Study Rationale and Benefit-Risk Assessment...................... 55

1.4.1 Rationale for Treatment of Mosunetuzumab in Combination with Polatuzumab Vedotin 56

1.4.2 Rationale for Treatment of Rituximab in Combination with Polatuzumab Vedotin (Arm M) 56

1.4.3 Benefit-Risk Assessment of Mosunetuzumab in Combination with Polatuzumab Vedotin 57

1.4.4 COVID-19 Related Risk Assessment 57

2. OBJECTIVES AND ENDPOINTS 58

3. STUDY DESIGN 63

3.1 Description of the Study... 63

3.1.1 Overview of Study 63

3.1.2 Dose-Finding Phase 67

3.1.2.1 Definition of DLT Assessment Period and DLTs 70

3.1.2.2 Dose-Finding Rules and Determination of the Maximum Tolerated Dose 72

3.1.2.3 Group A: Cycle 1 Step-Up Mosunetuzumab Escalation with Concurrent Administration of Polatuzumab Vedotin Starting in Cycle 1 73
3.1.2.4 Group B: Cycle 1 Polatuzumab Vedotin with Delayed Start Cycle 1 Step-Up Mosunetuzumab Escalation 75
3.1.2.5 Group C: Cycle 1 Step-Up Mosunetuzumab Escalation with Concurrent Administration of Polatuzumab Vedotin Starting in Cycle 2 76
3.1.2.6 Rules for Continued Dosing Beyond the DLT Assessment Period 81
3.1.3 Expansion Phase 82
3.1.3.1 Single-Arm Expansion 82
3.1.3.2 Arm K: Safety Run-In and Expansion 82
3.1.3.3 Randomized Phase II for R/R DLBCL 83
3.1.4 Treatment of Non-Hodgkin Lymphoma after Disease Progression 84
3.1.5 Mosunetuzumab Treatment Duration, Re-Treatment Following Disease Progression, and Crossover 85
3.1.5.1 Mosunetuzumab Treatment Duration and Re-Treatment 85
3.1.5.2 Crossover for Patients from Arm M (Rituximab plus Polatuzumab vedotin) to Arm M-crossover (Mosunetuzumab SC plus polatuzumab vedotin) 89
3.1.6 Internal Monitoring Committee 90
3.1.7 Independent Review Committee 90
3.2 End of Study and Length of Study 91
3.3 Rationale for Study Design 91
3.3.1 Rationale for Mosunetuzumab Dose and Schedule 91
3.3.1.1 Rationale for Intravenous Step-Up Dosing of Mosunetuzumab (Groups A, B, and C; Arms I and J) 92
3.3.1.2 Rationale for Subcutaneous Step-Up Dosing of Mosunetuzumab (Arms K and L) 92
3.3.2 Rationale for Polatuzumab Vedotin Dose and Schedule 94
3.3.3 Rationale for Dose-Finding Schedules (Groups A, B, and C) 94

3.3.4 Rationale for Dose-Finding Rules 95
3.3.5 Rationale for Mosunetuzumab DLT Assessment Period 95
3.3.6 Rationale for Continued Dosing beyond the DLT Assessment Period and Study Re-Treatment 96
3.3.7 Rationale for Patient Population 96
3.3.8 Rationale for the Single-Arm Expansion DLBCL, FL and MCL Cohorts 97
3.3.9 Rationale for the Randomized Phase II R/R DLBCL Control Arm 98
3.3.10 Rationale for Crossover 98
3.3.11 Rationale for PET-Defined Complete Response 98
3.3.12 Rationale for Use of an Independent Review Committee 99
3.3.13 Rationale for Mandatory Hospitalization with Mosunetuzumab Administration (Dose-Finding Groups A, B, and C; Arm K Safety Run-in) 99
3.3.14 Rationale for Using ASTCT Consensus Grading for CRS 100
3.3.15 Rationale for the Treatment of CRS Using Tocilizumab 100
3.3.16 Rationale for Pharmacokinetic and Anti-Drug Antibody Sampling Schedule 101
3.3.17 Rationale for Biomarker Assessments 102
3.3.18 Rationale for Patient-Reported Outcomes 103
4. MATERIALS AND METHODS 103
4.1 Patients 103
4.1.1 Inclusion Criteria 103
4.1.2 Exclusion Criteria 107
4.2 Method of Treatment Assignment 110
4.3 Study Treatment and Other Treatments Relevant to the Study Design 110
4.3.1 Study Treatment Formulation, Packaging, and Handling 111
4.3.1.1 Mosunetuzumab 111
4.3.1.2 Polatuzumab Vedotin 111

4.3.1.3 Rituximab ... 111
4.3.1.4 Tocilizumab ... 111
4.3.2 Study Treatment Dosage, Administration, and Compliance ... 111
4.3.2.1 Mosunetuzumab ... 112
4.3.2.2 Polatuzumab Vedotin ... 113
4.3.2.3 Rituximab ... 114
4.3.2.4 Tocilizumab ... 116
4.3.3 Study Treatment Regimens ... 117
4.3.3.1 Mosunetuzumab IV plus Polatuzumab Vedotin (Groups A, B, and C; Arms I, and J) ... 117
4.3.3.2 Mosunetuzumab SC plus Polatuzumab Vedotin (Arm K and L) ... 118
4.3.3.3 Rituximab plus Polatuzumab Vedotin (Arm M) ... 119
4.3.3.4 Cross Over Treatment to Mosunetuzumab plus Polatuzumab Vedotin (Arm M-crossover) ... 120
4.3.4 Investigational Medicinal Product Accountability ... 120
4.3.5 Continued Access to Mosunetuzumab, Polatuzumab Vedotin, Rituximab and Tocilizumab ... 120
4.4 Concomitant Therapy ... 121
4.4.1 Permitted Therapy ... 121
4.4.1.1 Treatment and Prophylaxis of Neutropenia ... 122
4.4.1.2 Prophylaxis for Tumor Lysis Syndrome ... 122
4.4.1.3 Prophylaxis for Infections ... 122
4.4.1.4 Pre-Planned Radiotherapy ... 122
4.4.1.5 COVID-19 Vaccinations ... 122
4.4.1.6 Other Concomitant Medications ... 123
4.4.2 Cautionary Therapy ... 123
4.4.2.1 Medications Given with Precaution Due to Effects Related to Cytochrome P450 Enzymes ... 123
4.4.2.2 Herbal Therapies ... 125
4.4.3 Prohibited Therapy ... 125
4.4.4 Additional Restrictions ... 125
4.4.4.1 Immunizations ... 125
4.5 Study Assessments ... 126

4.5.1 Informed Consent Forms and Screening Log 126
4.5.2 Medical History, Concomitant Medication, and Demographic Data 126
4.5.3 Physical Examinations 127
4.5.4 Prognostic Indices 127
4.5.5 Vital Signs 128
4.5.6 Tumor and Response Evaluations 128
4.5.6.1 Radiographic Assessments 128
4.5.6.2 Bone Marrow Assessments 129
4.5.7 Assessment of Transplant Eligibility Status 130
4.5.8 Laboratory, Biomarker, and Other Biological Samples 130
4.5.9 Electrocardiograms 133
4.5.10 Patient-Reported Outcomes 134
4.5.10.1 EQ-5D-5L 135
4.5.11 *Cumulative Illness Rating Scale-Geriatrics (CIRS-G)* 135
4.5.12 Optional Tumor Biopsies 136
4.5.13 Optional Samples for Research Biosample Repository 136
4.5.13.1 Overview of the Research Biosample Repository 136
4.5.13.2 Approval by the Institutional Review Board or Ethics Committee 136
4.5.13.3 Sample Collection 137
4.5.13.4 Confidentiality 137
4.5.13.5 Consent to Participate in the Research Biosample Repository 138
4.5.13.6 Withdrawal from the Research Biosample Repository 138
4.5.13.7 Monitoring and Oversight 139
4.6 Treatment, Patient, Study, and Site Discontinuation 139
4.6.1 Study Treatment Discontinuation 139
4.6.2 Patient Discontinuation from Study 140
4.6.3 Study Discontinuation 140

4.6.4 Site Discontinuation 140

5. ASSESSMENT OF SAFETY 141

5.1 Safety Plan 141

5.1.1 Mosunetuzumab Administration and Hospitalization 142

5.1.2 Risks Associated with Mosunetuzumab 143

5.1.2.1 Known Risks Associated with Mosunetuzumab 143

5.1.2.2 Potential Risks Associated with Mosunetuzumab 147

5.1.3 Risks Associated with Polatuzumab Vedotin 150

5.1.3.1 Known Risks Associated with Polatuzumab Vedotin 151

5.1.3.2 Potential Risks Associated with Polatuzumab Vedotin 152

5.1.4 Risks of Overlapping Toxicities with Mosunetuzumab and Polatuzumab Vedotin 154

5.1.5 Risks Associated with Rituximab 154

5.1.6 Risks of Overlapping Toxicities with Rituximab and Polatuzumab Vedotin 154

5.1.7 Management of Patients Who Experience Adverse Events 155

5.1.7.1 Dose Delays and Dose Modifications 155

5.1.7.2 Treatment Interruption 161

5.1.7.3 Schedule Modifications 162

5.1.7.4 Infusion-Related Reactions and Anaphylaxis 163

5.1.7.5 Cytokine Release Syndrome 164

5.1.7.6 Hemophagocytic Lymphohistiocytosis 171

5.1.7.7 Injection-Site Reactions after SC Administration of Mosunetuzumab 172

5.1.7.8 Tumor Lysis Syndrome 173

5.1.7.9 Neurological Adverse Events 174

5.1.7.10 Elevated Liver Enzymes and Hepatotoxicity 177

5.1.7.11 Neutropenia and Thrombocytopenia in Patients Receiving Mosunetuzumab 178

5.1.8 Internal Monitoring Committee 178

5.2 Safety Parameters and Definitions 179

5.2.1 Adverse Events 179
5.2.2 Serious Adverse Events (Immediately Reportable to the Sponsor) 180
5.2.3 Adverse Events of Special Interest (Immediately Reportable to the Sponsor) 181
5.2.4 Dose-Limiting Toxicities (Immediately Reportable to the Sponsor) 182
5.3 Methods and Timing for Capturing and Assessing Safety Parameters 182
5.3.1 Adverse Event Reporting Period 182
5.3.2 Eliciting Adverse Event Information 183
5.3.3 Assessment of Severity of Adverse Events 183
5.3.4 Assessment of Causality of Adverse Events 184
5.3.5 Procedures for Recording Adverse Events 185
5.3.5.1 Infusion-Related Reactions/Cytokine Release Syndrome and Anaphylaxis Attributed to Mosunetuzumab 185
5.3.5.2 Injection-Site Reactions 186
5.3.5.3 Infusion-Related Reactions Attributed to Polatuzumab Vedotin *or Rituximab* 186
5.3.5.4 Diagnosis versus Signs and Symptoms 186
5.3.5.5 Adverse Events That Are Secondary to Other Events 187
5.3.5.6 Persistent or Recurrent Adverse Events 187
5.3.5.7 Abnormal Laboratory Values 188
5.3.5.8 Abnormal Vital Sign Values 188
5.3.5.9 Abnormal Liver Function Tests 189
5.3.5.10 Deaths 189
5.3.5.11 Preexisting Medical Conditions 190
5.3.5.12 Lack of Efficacy or Worsening of DLBCL, FL, or MCL 190
5.3.5.13 Hospitalization or Prolonged Hospitalization 190
5.3.5.14 Patient-Reported Outcome Data 191
5.4 Immediate Reporting Requirements from Investigator to Sponsor 191
5.4.1 Emergency Medical Contacts 192

5.4.2 Reporting Requirements for Serious Adverse Events and Adverse Events of Special Interest 192
5.4.2.1 Events That Occur prior to Study Drug Initiation 192
5.4.2.2 Events That Occur after Study Drug Initiation 192
5.4.3 Reporting Requirements for Pregnancies 193
5.4.3.1 Pregnancies in Female Patients 193
5.4.3.2 Pregnancies in Female Partners of Male Patients 193
5.4.3.3 Abortions 194
5.4.3.4 Congenital Anomalies/Birth Defects 194
5.4.4 Reporting Requirements for Cases of Accidental Overdose or Medication Error 194
5.5 Follow-Up of Patients after Adverse Events 195
5.5.1 Investigator Follow-Up 195
5.5.2 Sponsor Follow-Up 196
5.6 Adverse Events That Occur after the Adverse Event Reporting Period 196
5.7 Expedited Reporting to Health Authorities, Investigators, Institutional Review Boards, and Ethics Committees 196
6. STATISTICAL CONSIDERATIONS AND ANALYSIS PLAN 197
6.1 Determination of Sample Size 197
6.2 Summaries of Conduct of Study 199
6.3 Summaries of Treatment Group Comparability 199
6.4 Efficacy Analyses 199
6.4.1 Primary Efficacy Endpoint 199
6.4.2 Secondary Efficacy Endpoints 200
6.4.3 Exploratory Efficacy Endpoints 201
6.5 Safety Analyses 201
6.6 Pharmacokinetic Analyses 202
6.7 Immunogenicity Analyses 202
6.8 Biomarker Analyses 202
6.9 Interim Analyses 203
6.9.1 Planned Interim Safety Analyses 203

6.9.2 Interim Futility Analysis 203
6.9.3 Optional Interim Analyses 204

7. DATA COLLECTION AND MANAGEMENT 204
7.1 Data Quality Assurance 204
7.2 Electronic Case Report Forms 204
7.3 Patient-Reported Outcome Data 205
7.4 Source Data Documentation 205
7.5 Use of Computerized Systems 206
7.6 Retention of Records 206

8. ETHICAL CONSIDERATIONS 206
8.1 Compliance with Laws and Regulations 206
8.2 Informed Consent 206
8.3 Institutional Review Board or Ethics Committee 208
8.4 Confidentiality 208
8.5 Financial Disclosure 209

9. STUDY DOCUMENTATION, MONITORING, AND ADMINISTRATION 209
9.1 Study Documentation 209
9.2 Protocol Deviations 209
9.3 Site Inspections 209
9.4 Administrative Structure 209
9.5 Publication of Data and Protection of Trade Secrets 210
9.6 Protocol Amendments 211

10. REFERENCES 212

LIST OF TABLES

Table 1 Summary of Clinical Trial Data in Patients with Relapsed or Refractory DLBCL/Transformed FL 40
Table 2 Summary of Clinical Trial Data in Patients with R/R FL 45
Table 3 Summary of Retrospective Outcome Analysis in Patients with Relapsed or Refractory MCL after Treatment with BTK Inhibitor 48

Table 4 Overview of Adverse Events in Studies GO29781, GO40516 and GO40554 ... 52
Table 5 CRS Events in Safety Evaluable Patients with R/R NHL Treated with Mosunetuzumab Either Monotherapy or Combination With Polatuzumab Vedotin ... 53
Table 6 Neutropenia and Febrile Neutropenia Events in Safety Evaluable Patients with R/R NHL Treated with Mosunetuzumab Either Monotherapy or Combination With Polatuzumab Vedotin ... 53
Table 7 Objectives and Corresponding Endpoints ... 59
Table 8 Examples of Dose Escalation and De-Escalation for Groups A, B, and C ... 80
Table 9 Administration of First and Subsequent Infusions of Rituximab ... 116
Table 10 Mosunetuzumab Plus Polatuzumab Vedotin Regimen (Group A) ... 118
Table 11 Mosunetuzumab Plus Polatuzumab Vedotin Regimen (Group B) ... 118
Table 12 Mosunetuzumab Plus Polatuzumab Vedotin Regimen(Group C) ... 118
Table 13 Mosunetuzumab SC Plus Polatuzumab Vedotin Regimen (Arm K and L) ... 119
Table 14 Rituximab Plus Polatuzumab Vedotin Regimen (Arm M) ... 119
Table 15 Steps of Dose Reduction for Polatuzumab Vedotin ... 156
Table 16 Guidelines for Dose Delay, Modification or Discontinuation of Mosunetuzumab [b], Polatuzumab Vedotin and Rituximab ... 157
Table 17 Management of Infusion-Related Symptoms for Polatuzumab Vedotin or Rituximab ... 164
Table 18 Management of Cytokine Release Syndrome for Patients Receiving Mosunetuzumab (IV and SC) ... 165
Table 19 Management Guidelines for Suspected and Confirmed Hemophagocytic Lymphohistiocytosis ... 172
Table 20 Management Guidelines for Injection-Site Reactions ... 172
Table 21 Management Guidelines for Neurological Adverse Events for Patients Receiving Mosunetuzumab ... 175
Table 22 Management Guidelines for Liver Function Test Abnormalities and Hepatic Events for Patients Receiving Mosunetuzumab ... 177
Table 23 Adverse Event Severity Grading Scale for Events Not Specifically Listed in NCI CTCAE ... 183
Table 24 Causal Attribution Guidance ... 185
Table 25 Clopper-Pearson Exact 95% Confidence Intervals for Assumed Observed ORR based on Sample Size of [redacted], and [redacted] Patients ... 198
Table 26 Probability of Safety-Signal Detection based on Sample Size of [redacted], and [redacted] Patients ... 198

LIST OF FIGURES

Figure 1 Study Schema 65
Figure 2 Study Schema: Dose-Finding Phase (Groups A, B, and C) 69
Figure 3 DLT Assessment Periods for Groups A, B, and C 78
Figure 4 Mosunetuzumab plus Polatuzumab Vedotin Treatment/Re-Treatment Schema (Dose Escalation Phase, Arms I, J, *and K*) 89
Figure 5 Mosunetuzumab plus Polatuzumab Vedotin Treatment/Re-Treatment/Crossover Schema (Arms L and M) 90
Figure 6 POPPK Model Simulated Mosunetuzumab Concentration-Time Profiles Following IV 1/2/60/30 mg and SC 5/45/45 mg Q3W Dose Regimens 93

LIST OF APPENDICES

Appendix 1 Schedule of Activities 225
Appendix 2 Schedule for Post-Treatment Follow-Up 241
Appendix 3 Schedule of Pharmacokinetic, Immunogenicity, and Biomarker Blood Samples: Groups A, B, and C (Dose-Finding Phase) and Arms I and J (Combination Expansion Phase) 243
Appendix 4 Schedule of Pharmacokinetic, Immunogenicity, and Biomarker Blood Samples: Arms K and L (Mosunetuzumab SC Combination Expansion Phase) 249
Appendix 5 Schedule of Pharmacokinetic, Immunogenicity, and Biomarker Blood Samples: Arm M (Rituximab-Polatuzumab Vedotin Control; Expansion Phase) 252
Appendix 6 Schedule for Tocilizumab Treatment of Cytokine Release Syndrome 254
Appendix 7 Lugano Response Criteria for Malignant Lymphoma (Cheson et al. 2014) 256
Appendix 8 ASTCT Cytokine Release Syndrome Consensus Grading 261
Appendix 9 Examples of Sensitive In Vivo CYP Substrates and CYP Substrates with Narrow Therapeutic Range 262
Appendix 10 Recommended Anaphylaxis Management 264
Appendix 11 Follicular Lymphoma International Prognostic Index, International Prognostic Index, and Mantle Cell Lymphoma International Prognostic Index 265
Appendix 12 EuroQol 5-Dimension, 5-Level (EQ-5D-5L) Questionnaire 270
Appendix 13 Neurologic Adverse Events that May Affect Driving 273
Appendix 14 Recommendations for the Use of White Blood Cell Growth Factors 274
Appendix 15 Definitions of Laboratory and Clinical Tumor Lysis Syndrome 276
Appendix 16 Cumulative Illness Rating Scale-Geriatric (CIRS-G) 277

PROTOCOL AMENDMENT ACCEPTANCE FORM

**TITLE:**	**AN OPEN-LABEL, MULTICENTER, PHASE Ib/II TRIAL EVALUATING THE SAFETY, TOLERABILITY, PHARMACOKINETICS, AND EFFICACY OF MOSUNETUZUMAB (BTCT4465A) IN COMBINATION WITH POLATUZUMAB VEDOTIN IN PATIENTS WITH B-CELL NON-HODGKIN LYMPHOMA**
**PROTOCOL NUMBER:**	GO40516
**VERSION NUMBER:**	8
**EUDRACT NUMBER:**	2018-001141-13
**IND NUMBER:**	120651
**NCT NUMBER**	NCT03671018
**TEST PRODUCTS:**	Mosunetuzumab (RO7030816; BTCT4465A)<br>Polatuzumab vedotin (RO5541077; DCDS4501S)
**MEDICAL MONITOR:**	[redacted], *Pharm.D.*
**SPONSOR:**	F. Hoffmann-La Roche Ltd

I agree to conduct the study in accordance with the current protocol.

____
Principal Investigator's Name (print)

____ ____________________
Principal Investigator's Signature Date

Please retain the signed original of this form for your study files. Please return a copy of the signed form as instructed by the CRO.

PROTOCOL SYNOPSIS

**TITLE:**	**AN OPEN-LABEL, MULTICENTER, PHASE Ib/II TRIAL EVALUATING THE SAFETY, TOLERABILITY, PHARMACOKINETICS, AND EFFICACY OF MOSUNETUZUMAB (BTCT4465A) IN COMBINATION WITH POLATUZUMAB VEDOTIN IN PATIENTS WITH B-CELL NON-HODGKIN LYMPHOMA**
**PROTOCOL NUMBER:**	GO40516
**VERSION NUMBER:**	8
**EUDRACT NUMBER:**	2018-001141-13
**IND NUMBER:**	120651
**NCT NUMBER**	NCT03671018
**TEST PRODUCTS:**	Mosunetuzumab (RO7030816; BTCT4465A)<br>Polatuzumab vedotin (RO5541077; DCDS4501S)
**PHASE:**	Phase Ib/II
**INDICATION:**	B-cell non-Hodgkin lymphoma
**SPONSOR:**	F. Hoffmann-La Roche Ltd

Objectives and Endpoints

The study *will evaluate the safety, tolerability, pharmacokinetics, and efficacy of* IV or SC mosunetuzumab plus polatuzumab vedotin in patients with relapsed or refractory (R/R) B-cell non-Hodgkin lymphoma (NHL). The randomized Phase II portion of the study will evaluate the safety and efficacy of mosunetuzumab SC plus polatuzumab vedotin compared to rituximab plus polatuzumab vedotin in patients with R/R diffuse large B cell lymphoma (DLBCL). Specific objectives and corresponding endpoints for the study are outlined below.

Phase Ib-Specific Objectives:	
**Safety Objective**	**Corresponding Endpoints**
• To evaluate the safety and tolerability of mosunetuzumab plus polatuzumab vedotin in patients with R/R DLBCL or FL, including estimation of the MTD, determination of the RP2D, and characterization of DLTs	• Occurrence and severity of adverse events, including DLTs, with severity determined according to NCI CTCAE v5.0; for CRS, severity determined according to the ASTCT CRS Consensus Grading criteria<br>• Change from baseline in targeted vital signs<br>• Change from baseline in targeted clinical laboratory test results
**Efficacy Objective**	**Corresponding Endpoints**
• To make a preliminary assessment of the anti-tumor activity of mosunetuzumab plus polatuzumab vedotin	• CR rate at the time of PRA based on PET-CT, as determined by the investigator using Lugano 2014 criteria

Phase Ib-Specific Objectives (cont.):	
**Efficacy Objective (cont.)**	**Corresponding Endpoints (cont.)**
	• Best ORR (CR or PR at any time) in the study based on PET and/or CT scan, as determined by the investigator using Lugano 2014 criteria<br>• DOR, defined as the time from the first occurrence of a documented objective response to disease progression or relapse, as determined by the investigator using Lugano 2014 criteria, or death from any cause, whichever occurs first
**Phase II-Specific Objectives:**	
**Primary Efficacy Objectives**	**Corresponding Endpoint**
• To evaluate the efficacy of mosunetuzumab IV plus polatuzumab vedotin in patients with R/R FL (Arm I)<br>• To evaluate the efficacy of mosunetuzumab IV plus polatuzumab vedotin (Arm J) in patients with R/R DLBCL<br>• To evaluate the efficacy of mosunetuzumab SC plus polatuzumab vedotin (Arm K) in patients with R/R MCL<br>• To evaluate the efficacy of mosunetuzumab SC plus polatuzumab vedotin (Arm L) compared to rituximab plus polatuzumab vedotin (Arm M) in patients with R/R DLBCL	• Best ORR (CR or PR at any time) in the study based on PET-CT and/or CT scan, as determined by the IRC using Lugano 2014 criteria
**Secondary Efficacy Objectives**	**Corresponding Endpoints**
• To evaluate the efficacy of mosunetuzumab IV plus polatuzumab vedotin (Arm I) in patients with R/R FL<br>• To evaluate the efficacy of mosunetuzumab IV plus polatuzumab vedotin (Arm J) in patients with R/R DLBCL<br>• To evaluate the efficacy of mosunetuzumab SC plus polatuzumab vedotin (Arm K) in patients with R/R MCL<br>• To evaluate the efficacy of mosunetuzumab SC plus polatuzumab vedotin (Arm L) compared to rituximab plus polatuzumab vedotin (Arm M) in patients with R/R DLBCL	• Best ORR (CR or PR at any time) on study based on PET-CT and/or CT scan, as determined by the investigator using Lugano 2014 criteria<br>• Best CR rate on study based on PET-CT and/or CT scan, as determined by the investigator and IRC using Lugano 2014 criteria<br>• CR rate at the time of PRA based on PET-CT, as determined by the investigator and IRC using Lugano 2014 criteria<br>• ORR, defined as CR or PR, at PRA based on PET-CT, as determined by the investigator and IRC using Lugano 2014 criteria

Phase II-Specific Objectives (cont.):	
**Secondary Efficacy Objectives**	**Corresponding Endpoints**
	• DOR, defined as the time from the first occurrence of a documented objective response to disease progression or relapse as determined by the investigator and IRC using Lugano 2014 criteria or death from any cause, whichever occurs first<br>• PFS, defined as the time from first study treatment to the first occurrence of disease progression or relapse, as determined by the investigator and IRC using Lugano 2014 criteria, or death from any cause, whichever occurs first<br>• EFS, defined as the time from first study treatment to the first occurrence of disease progression or relapse, as determined by the investigator and IRC using Lugano 2014 criteria, initiation of NALT, or death from any cause, whichever occurs first.<br>• OS, defined as the time from first study treatment to death from any cause
**Exploratory Efficacy Objectives**	**Corresponding Endpoints**
• To evaluate the efficacy of mosunetuzumab IV plus polatuzumab vedotin (Arm I) in patients with R/R FL<br>• To evaluate the efficacy of mosunetuzumab IV plus polatuzumab vedotin (Arm J) in patients with R/R DLBCL<br>• To evaluate the efficacy of mosunetuzumab SC plus polatuzumab vedotin (Arm K) in patients with R/R MCL<br>To evaluate the efficacy of mosunetuzumab SC plus polatuzumab vedotin (Arm L) compared to rituximab polatuzumab vedotin (Arm M) in patients with R/R DLBCL	• Proportion of patients who undergo SCT after achieving a response while in the study<br>• Proportion of patients who undergo allogeneic SCT after achieving a response while in the study<br>• *CIRS-G prognostic impact on PFS and OS for patients ≥65 years old (Arm J, Arm L, and Arm M only)*
**Exploratory Health Status Utility Objective**	**Corresponding Endpoint**
• To assess health status of patients (Arm K only)	• Health status (EQ-5D-5L)

Phase II-Specific Objectives (cont.):	
**Safety Objectives**	**Corresponding Endpoints**
• To evaluate the safety of mosunetuzumab IV plus polatuzumab vedotin (Arm I) in patients with R/R FL<br>• To evaluate the safety of mosunetuzumab IV plus polatuzumab vedotin (Arm J) in patients with R/R DLBCL<br>• To evaluate the safety of mosunetuzumab SC plus polatuzumab vedotin (Arm K) in patients with R/R MCL<br>• To evaluate the safety of mosunetuzumab SC plus polatuzumab vedotin (Arm L) compared to rituximab polatuzumab vedotin (Arm M) in patients with R/R DLBCL	• Occurrence and severity of adverse events, with severity determined according to NCI CTCAE v5.0. For CRS, severity determined according to the ASTCT CRS Consensus Grading criteria.<br>• Change from baseline in targeted vital signs<br>• Change from baseline in targeted clinical laboratory test results
**Objectives for Both Phase Ib and Phase II:**	
**Pharmacokinetic Objective**	**Corresponding Endpoints**
• To characterize the pharmacokinetics of mosunetuzumab (SC and IV) when administered in combination with polatuzumab vedotin (Groups A, B, and C; Arms I, J, K, and L)	• For mosunetuzumab pharmacokinetics in combination with polatuzumab vedotin:<br>• C_{max}<br>• C_{min}<br>• Total exposure (AUC), CL, and volume of distribution, as estimated by population PK modeling, as appropriate, and supported by data
**Exploratory Pharmacokinetic Objectives**	**Corresponding Endpoints**
• To characterize the pharmacokinetics of polatuzumab vedotin when administered in combination with mosunetuzumab (Groups A, B, and C; Arms I, J, K, and L) or rituximab (Arm M)	• For polatuzumab vedotin pharmacokinetics in combination with mosunetuzumab:<br>• C_{EOI}<br>• C_{trough}<br>• Total exposure (AUC), CL, and volume of distribution, as estimated by population PK modeling, as appropriate, and supported by data
• To characterize the relationship between pharmacokinetics and safety, biomarkers, or efficacy	• Relationship between pharmacokinetics and safety, biomarkers, or efficacy endpoints, as appropriate
• To assess potential PK interactions between mosunetuzumab and polatuzumab vedotin (Groups A, B, and C; Arms I, J, K, and L)	• Concentrations of mosunetuzumab when administered in combination with polatuzumab vedotin compared with mosunetuzumab given as a single agent based on historical data<br>• Concentrations of polatuzumab vedotin analytes when administered in combination with mosunetuzumab compared with polatuzumab vedotin as a single agent based on historical data

Immunogenicity Objectives	Corresponding Endpoint
• To assess the incidence of ADAs to mosunetuzumab (Groups A, B, and C; Arms I, J, K, and L)<br>• To assess the incidence of ADAs to polatuzumab vedotin (Groups A, B, and C; Arms I, J, K, and L)	• Relationship between ADA status and efficacy, safety, pharmacokinetics, and biomarkers
**Exploratory Biomarker Objectives**	**Corresponding Endpoints**
• To identify biomarkers that are predictive of response to mosunetuzumab plus polatuzumab vedotin (i.e., predictive biomarkers), are associated with progression to a more severe disease state (i.e., prognostic biomarkers), are associated with acquired resistance to mosunetuzumab plus polatuzumab vedotin, are associated with susceptibility to developing adverse events, can provide evidence of mosunetuzumab plus polatuzumab vedotin activity, or can increase the knowledge and understanding of disease biology<br>• To make a preliminary assessment of response to mosunetuzumab plus polatuzumab vedotin in different clinical and biologic prognostic subgroups of NHL<br>• To make a preliminary assessment of MRD status following mosunetuzumab treatment in combination with polatuzumab vedotin (Groups A, B, and C; Arms I, J, K*, L and M*)	• Association between prognostic subtypes, exploratory biomarkers, and PET-CT CR rate, ORR, DOR, PFS, and EFS endpoints<br>• Relationship over time between ctDNA and tumor burden as measured by imaging

ADA = anti-drug antibody; ASTCT = American Society for Transplantation and Cellular Therapy; AUC = area under the concentration–time curve; C_{EOI} = concentration at end of infusion; *CIRS-G = Cumulative Illness Rating Scale-Geriatric;* CL = clearance; C_{max} = maximum serum concentration; Cmin = minimum serum concentration; CR = complete response; CRS = cytokine release syndrome; CT = computed tomography (scan); C_{trough} = trough concentration; ctDNA = circulating tumor DNA; DLBCL = diffuse large B-cell lymphoma; DLT = dose-limiting toxicity; DOR = duration of response; EFS = event-free survival; EQ-5D-5L = EuroQol 5-Dimension, 5-Level (questionnaire); FL = follicular lymphoma; IRC = Independent Review Committee; MCL = mantle cell lymphoma; MRD = minimal residual disease; MTD = maximum tolerated dose; NALT = new anti-lymphoma treatment; NCI CTCAE v5.0 = National Cancer Institute Common Terminology Criteria for Adverse Events, Version 5.0; NHL = non-Hodgkin lymphoma; ORR = objective response rate; OS = overall survival; PET = positron emission tomography (scan); PET-CR = complete response based on positron emission tomography scan; PET-CT = positron emission tomography-computed tomography (scan); PFS = progression-free survival; PK = pharmacokinetic; PR = partial response; PRA = primary response assessment; RP2D = recommended Phase II dose; R/R = relapsed or refractory; SCT = stem-cell transplantation.

Study Design

Description of Study

Overview of Study Design

This Phase Ib/II open-label, multicenter study will evaluate the safety, tolerability, pharmacokinetics, and efficacy of IV or SC mosunetuzumab in combination with polatuzumab vedotin in patients with DLBC, FL*, and mantle cell lymphoma (MCL)*. The study will include an initial dose-finding phase followed by a single-arm expansion phase for second line or later (2L+) patients with R/R DLBCL and 2L+ R/R FL. In addition, mosunetuzumab SC dosing in combination with polatuzumab vedotin will be evaluated in patients with at least 2 prior lines of systemic therapy (3L+) for the treatment of R/R MCL (Arm K), and in patients with (2L+) R/R DLBCL in the randomized Phase II portion of the study (Arm L and M).

All patients will be closely monitored for adverse events throughout the study and for at least 90 days after the last dose of study treatment or until the initiation of *next anti-lymphoma treatment*, whichever is earlier. Adverse events will be graded according to the National Cancer Institute Common Terminology Criteria for Adverse Events, Version 5.0 (NCI CTCAE v5.0), with the exception of cytokine release syndrome (CRS) events, which will be graded according to the ASTCT CRS Consensus Grading criteria. An IMC will be established to monitor patient safety throughout the study.

Blood samples will be taken at various timepoints before and during study treatment administration for biomarker analyses and to characterize the PK properties of mosunetuzumab and polatuzumab vedotin, as well as the immunogenicity of mosunetuzumab and polatuzumab vedotin when given in combination.

Response in the Phase II expansion portion of the study (Arms I, J, K, L, and M) will be determined by an Independent Review Committee (IRC) and investigators using the Lugano Response Criteria for Malignant Lymphoma, hereinafter referred to as the "Lugano 2014 criteria," at the following timepoints:

- Interim response assessment at will occur between D15 of C4 and D21 of C4, prior to C5
- Primary response assessment (PRA)

  For all cohorts (Groups A and C; Arms I, J, K, L, and M, if following Group A or C dosing schedule): at the end of Cycle 8 (C8D21 ± 1 week) (or end of C9 [C9D21 ± 1 week] for Group B; Arms I, J, K, L, and M, if following the Group B dosing schedule)

Patients will continue to be evaluated every 3 months (± 2 weeks) by computed tomography (CT) scan and/or positron emission tomography (PET)-CT for the first year after C1D1, and then every 6 months (± 2 weeks) until disease progression, death, withdrawal of consent, or initiation of *next anti-lymphoma treatment*. Tumor assessments should also be performed to confirm clinical suspicion of relapse or disease progression.

Study treatments will be administered every 21 days.

- Mosunetuzumab will be administered for 8–17 cycles for Groups A, B, and C, and Arms I and J.
- Mosunetuzumab will be administered for 17 cycles for Arm K only.
- *Mosunetuzumab will be administered for 8 cycles for Arm L and Arm M-crossover.*
- *Rituximab (375 mg/m²) will be administered for 8 cycles for Arm M.*
- Polatuzumab vedotin (1.8 mg/kg) will be administered for 6 cycles *for Groups A, B, and C, and Arms I, J, K, L and M.*
- *Polatuzumab vedotin (1.8 mg/kg) will be administered for 6 cycles or less for Arm M-*crossover, depending on the number of cycles of polatuzumab vedotin administered in Arm M.

Dose-Finding Phase

The purpose of the dose-finding phase is to determine the recommended Phase II dose (RP2D) and schedule for mosunetuzumab when given in combination with fixed doses of polatuzumab vedotin (1.8 mg/kg) in patients with R/R DLBCL or FL. Approximately 9-42 patients with either R/R DLBCL or FL may be enrolled in up to three dose-escalation treatment groups:

- **Group A:** C1 step-up mosunetuzumab escalation with concurrent administration of polatuzumab vedotin starting in C1, both administered by IV infusion
- **Group B:** C1 polatuzumab vedotin with delayed start C1 step-up mosunetuzumab escalation, both administered by IV infusion
- **Group C:** C1 step-up mosunetuzumab escalation with concurrent administration of polatuzumab vedotin starting in C2, both administered by IV infusion

Dose escalation Groups A, B, and C may be run sequentially or in parallel, at the discretion of the Sponsor. Dose escalation will be performed based on a modified 3+3 design. Dose-escalation cohorts (within each group) will consist of at least 3 patients, unless dose-limiting toxicity (DLTs) are observed in the first 2 patients prior to enrollment of a third patient. Approximately 6-12 patients will be treated at the RP2D and schedule of mosunetuzumab in combination with polatuzumab vedotin prior to the expansion phase.

For each dose-escalation cohort in Groups A, B, and C, treatment will be staggered such that the second patient enrolled in the cohort will receive the first dose of study treatment at least 72 hours after the first enrolled patient receives the first dose of study treatment, to assess for any severe and unexpected acute drug or infusion-related toxicities. Dosing in subsequent patients in each cohort will be staggered by at least 24 hours from the end of the prior patient's administration. In each scenario, the Sponsor must receive documentation of the status of the prior patient before the next patient receives the first dose of study treatment.

Patients will be closely monitored for adverse events during a DLT assessment window. Adverse events meeting the criteria for DLT, as defined below, will be reported to the Sponsor within 24 hours. Staggered patient enrollment will not be required for enrollment of additional patients to acquire additional safety and pharmacodynamics (PD) data at a dose level that has been shown to not exceed the maximum tolerated dose (MTD).

Patients exhibiting acceptable safety and evidence of clinical benefit may continue to receive study treatment every 21 days for 8 cycles (or 17 cycles if partial response [PR] or stable disease [SD] after 8 cycles) for mosunetuzumab, and for 6 cycles for polatuzumab vedotin, until confirmed objective disease progression or unacceptable toxicity, whichever occurs first. Re-treatment with mosunetuzumab combined with polatuzumab vedotin-based on clinical responses to initial treatment are detailed in the protocol.

Dose-finding Groups A, B, or C may be prioritized or suspended by the Sponsor based on the overall safety profile, in consultation with the IMC. The IMC will review cumulative safety data and make recommendations regarding dose escalation and overall study conduct on the basis of trial safety data to ensure patient safety while receiving study treatment. These include recommendations to open or suspend patient enrollment in a given dose-escalation cohort based on the overall benefit-risk profile of mosunetuzumab in combination with polatuzumab vedotin during dose finding.

Mosunetuzumab dose levels will be independent of patient weight (flat-dosing). The starting dose level of step-up mosunetuzumab is 1 mg (DL_1, fixed for all schedules), 2 mg (DL_2, fixed for all schedules, given 7 days after DL_1), and 9 mg (DL_3, initial mosunetuzumab test dose, given 7 days after DL_2), for each initial cohort in Groups A, B, and C based on preliminary data from Study GO29781; for additional details, see the rationale for mosunetuzumab starting dose and schedule.

During dose finding in Groups A, B, and C, only the DL_3 test dose may be escalated or de-escalated according to the rules below.

See the protocol for information on the definition of the DLT assessment period and DLTs; dose-finding rules and determination of the MTD for Groups A, B, and C; and rules for continued dosing beyond the DLT assessment period.

Expansion Phase

The expansion phase is designed to further assess the safety and efficacy of mosunetuzumab and polatuzumab vedotin at the RP2D and schedule determined in the dose-finding phase. Mosunetuzumab dose and schedule in combination with polatuzumab vedotin to be assessed in the expansion phase will be determined based on IMC recommendation and in consultation with the investigators following a review of cumulative safety data in dose finding. Patients with R/R DLBCL, R/R FL, or R/R MCL will be enrolled during the expansion phase and treated as described below.

Single-Arm Expansion

Approximately [redacted] patients will be treated with mosunetuzumab plus polatuzumab vedotin in the single-arm expansion phase. The patient cohorts will be assigned to the following arms:

- **Arm I:** R/R FL (Grade 1–3a); [redacted] patients (mosunetuzumab IV plus polatuzumab vedotin)
- **Arm J:** R/R DLBCL, transformed FL, or Grade 3b FL; 100 patients (mosunetuzumab IV plus polatuzumab vedotin)
- **Arm K:** R/R MCL; [redacted] patients (mosunetuzumab SC plus polatuzumab vedotin)

Dose expansion Arms I, J, and K may be run sequentially or in parallel and may also be prioritized or suspended, at the discretion of the Sponsor.

See the protocol for information on treatment of NHL after disease progression, and mosunetuzumab treatment duration and re-treatment following disease progression.

Randomized Phase II for R/R DLBCL

Approximately [redacted] patients with R/R DLBCL will be randomized at 1:1 ratio to the treatment with mosunetuzumab SC plus polatuzumab vedotin (Arm L) and rituximab plus polatuzumab vedotin (Arm M):

Arm L: R/R DLBCL; [redacted] patients (mosunetuzumab SC plus polatuzumab vedotin)

Arm M: R/R DLBCL; [redacted] patients (rituximab IV plus polatuzumab vedotin)

Patients will be randomized 1:1 with the use of stratified permuted blocks. Randomization will be stratified by the number of prior treatment regimens (one prior line of therapy versus ≥2 prior lines of therapy).

Number of Patients

Up to [redacted] patients are expected to be enrolled in this study at approximately 40 investigative sites globally.

Target Population

Inclusion Criteria

Patients must meet the following criteria for study entry:

- Signed Informed Consent Form
- Age ≥ 18 years at time of signing Informed Consent Form
- Able to comply with the study protocol and procedures in the investigator's judgment
- ECOG PS of 0, 1, or 2
- Life expectancy of at least 12 weeks
- Patients must have histologically confirmed FL, DLBCL or MCL from the following diagnoses by 2016 WHO classification of lymphoid neoplasms, that have either relapsed or have become refractory to a prior regimen as defined below.

  R/R FL (Groups A, B, C and Arm I)

  – Histology

    FL Grade 1, 2, 3a

    In situ follicular neoplasia

    Duodenal-type FL

    Pediatric-type FL

- Relapsed to prior regimen(s) after having a documented history of response (complete response [CR], CR unconfirmed [CRu], or PR) of ≥6 months in duration from completion of regimen(s) or
- Refractory to any prior regimen, defined as no response to the prior therapy, or progression within 6 months of completion of the last dose of therapy
- Must have received at least one prior systemic treatment regimen containing an anti-CD20-directed therapy.

R/R DLBCL (Groups A, B, C and Arm *J, L and M*)

- Histology

  DLBCL, not otherwise specified (NOS) (including germinal center B cell type and activated B-cell type)

  T-cell/histiocyte-rich large B-cell lymphoma

  High-grade B-cell lymphoma with MYC and BCL-2 and/or BCL-6 rearrangements

  EBV+DLBCL, NOS

  HHV8+DLBCL, NOS

  High grade B-cell lymphoma, NOS

  Anaplastic lymphoma kinase (ALK)+large B-cell lymphoma

  FL Grade 3b
- Relapsed to prior regimen(s) after having a documented history of response (CR, CRu, or PR) of ≥ 6 months in duration from completion of regimen(s) or
- Refractory to any prior regimen, defined as no response to the prior therapy, or progression within 6 months of completion of the last dose of therapy
- Must have received at least one prior systemic treatment regimen containing an anti-CD20-directed therapy.
- Transformed FL is an eligible diagnosis for enrollment in the DLBCL cohort but must be R/R to standard therapies for transformed FL.

  Patients with Richter's transformation are not eligible for enrollment into the study.

  The Sponsor may retain the option to limit the number of patients with transformed FL enrolled in the study.
- Grade 3b FL is an eligible diagnosis for enrollment in the DLBCL cohort but must be R/R to standard therapies for aggressive NHL.

  The Sponsor may retain the option to limit the number of patients with Grade 3b FL enrolled in the study.
- High grade B-cell lymphoma with MYC and BCL2 and/or BCL6 rearrangements and high-grade B-cell lymphoma NOS are eligible diagnoses for enrollment in the DLBCL cohort but must be R/R to standard therapies for aggressive NHL.

The Sponsor may retain the option to limit the number of patients enrolled in the R/R DLBCL cohort based on the prior systemic treatment.

R/R MCL (Arm K)

- Histology

  MCL
- Relapsed to prior regimen(s) after having a documented history of response (CR, CRu, or PR) of ≥ 6 months in duration from completion of regimen(s)
- Refractory to any prior regimen, defined as no response to the prior therapy, or progression within 6 months of completion of the final dose of therapy
- Must have received at least two prior systemic treatment regimens, which include agents from all three classes below:

  Anti-CD20-directed therapy

BTK inhibitor

Anthracycline or bendamustine

- Measurable disease, defined as at least one bi-dimensionally measurable nodal lesion, defined as > 1.5 cm in its longest dimension, or at least one bi-dimensionally measurable extranodal lesion, defined as > 1.0 cm in its longest dimension
- Pathology report for the initial histopathology diagnosis and the most recent histopathology diagnosis prior to study entry must be provided.
  - Patients with transformed FL must also provide the pathology report at the time of disease transformation.
  - The results of all tests conducted on the tissue at initial diagnosis, including, but not limited to, tests assessing cell of origin, *BCL2* and *MYC* abnormalities, should be provided if done.
  - For patients with MCL, results of all tests conducted on the tissue at initial diagnosis and/or relapse, including, but not limited to, the MCL subtype (nodular and blastoid), Ki-67 proliferation index, and TP53 mutation status, should be provided if done.
- Agreement to provide tumor samples as follows:

  Agreement to undergo biopsy from a safely accessible site per investigator determination.

  Patients who are unable to undergo biopsy procedures may be eligible for study enrollment if archival tumor tissue samples (paraffin blocks or at least 20 unstained slides) in place of a fresh biopsy, can be sent to the Sponsor.

  Bone marrow biopsy and aspirate (if applicable)
- Adverse events from prior anti-cancer therapy resolved to Grade $\leq$1
- Laboratory values as follows:
  - Hepatic Function

    AST and ALT $\leq$2.5 $\times$ ULN

    Total bilirubin $\leq$1.5 $\times$ ULN

    Patients with a documented history of Gilbert syndrome and in whom total bilirubin elevations are accompanied by elevated indirect bilirubin are eligible.
  - Hematologic Function

    Platelet count $\geq$75,000/mm3 without transfusion within 14 days prior to first dose of study treatment

    ANC $\geq$ 1000/mm3

    Total hemoglobin $\geq$9 g/dL without transfusion within 21 days prior to first dose of study treatment
  - Patients with extensive marrow involvement of NHL and/or disease-related cytopenias (e.g., immune thrombocytopenia) may be enrolled if below is met.

    Platelet count $\geq$50,000/mm^3 without transfusion within 14 days

    ANC $\geq$500/mm^3

    Any hemoglobin but without transfusion within 7 days
  - INR $\leq$ 1.5 $\times$ ULN in the absence of therapeutic anticoagulation
  - PTT or aPTT $\leq$1.5 $\times$ ULN in the absence of lupus anticoagulant or therapeutic anticoagulation
- Estimated creatinine CL $\geq$ 50 mL/min by Cockroft-Gault method or other institutional standard methods, e.g. based on nuclear medicine renal scan
- *Patients who have a negative HIV test at screening.*

  Patients with a positive HIV test at screening are also eligible provided they are stable on anti-retroviral therapy, have a CD4 count ≥ 200/µL, and have an undetectable viral load.
- For women of childbearing potential: agreement to remain abstinent (refrain from heterosexual intercourse) or use contraceptive measures, and agreement to refrain from donating eggs, as defined below:

- Women must remain abstinent or use contraceptive methods with a failure rate of $<$ 1% per year during the treatment period and for 3 months after the final dose of mosunetuzumab, 9 months after the final dose of polatuzumab vedotin, 12 months after the final dose of rituximab, and 3 months after the final dose of tocilizumab, as applicable. Women must refrain from donating eggs during this same period.
- A woman is considered to be of childbearing potential if she is postmenarcheal, has not reached a postmenopausal state ($\geq$ 12 continuous months of amenorrhea with no identified cause other than menopause), and has not undergone surgical sterilization (removal of ovaries and/or uterus).
- Examples of contraceptive methods with a failure rate of $<$ 1% per year include bilateral tubal ligation, male sterilization, established proper use of hormonal contraceptives that inhibit ovulation, hormone-releasing intrauterine devices, and copper intrauterine devices.
- The reliability of sexual abstinence should be evaluated in relation to the duration of the clinical trial and the preferred and usual lifestyle of the patient. Periodic abstinence (e.g., calendar, ovulation, symptothermal, or postovulation methods) and withdrawal are not acceptable methods of contraception.

- For men: agreement to remain abstinent (refrain from heterosexual intercourse) or use a condom, and agreement to refrain from donating sperm, as defined below:
  - With female partners of childbearing potential or pregnant female partners, men must remain abstinent or use a condom during the treatment period and for, 6 months after the final dose of polatuzumab vedotin, 3 months after the final dose of rituximab, and 60 days after the final dose of tocilizumab, as applicable, to avoid exposing the embryo. Men must refrain from donating sperm during this same period.
  - The reliability of sexual abstinence should be evaluated in relation to the duration of the clinical trial and the preferred and usual lifestyle of the patient. Periodic abstinence (e.g., calendar, ovulation, symptothermal, or postovulation methods) and withdrawal are not acceptable methods of contraception.

Exclusion Criteria

Patients who meet any of the following criteria will be excluded from study entry:

- Inability to comply with protocol-mandated hospitalization and activity restrictions
- Pregnant or breastfeeding, or intending to become pregnant during the study or within 3 months after the final dose of mosunetuzumab, 9 months after the final dose of polatuzumab vedotin, 12 months after the final dose of rituximab, and 3 months after the final dose of tocilizumab, as applicable.
  - Women of childbearing potential must have a negative serum pregnancy test result within 7 days prior to initiation of study treatment.
- Prior treatment with mosunetuzumab or other CD20-directed bispecific antibodies
- Prior treatment with polatuzumab vedotin
- Current > Grade 1 peripheral neuropathy
- Prior use of any monoclonal antibod*ies*, radioimmunoconjugate*s* or ADC*s for anti-lymphoma treatment* within 4 weeks before first dose of study treatment
- Treatment with any chemotherapeutic agent, or treatment with any other anti-*lymphoma* agent (investigational or otherwise) within 4 weeks or 5 half-lives of the drug, whichever is shorter, prior to first dose of study treatment
- Treatment with radiotherapy within 2 weeks prior to the first dose of study treatment
  - If patients have received radiotherapy within 4 weeks prior to the first study treatment administration, patients must have at least one measurable lesion outside of the radiation field. Patients who have only one measurable lesion that was previously irradiated but subsequently progressed are eligible.
- Autologous SCT within 100 days prior to first study treatment administration

- Prior treatment with CAR-T therapy within 30 days before first study treatment administration
- Current eligibility for autologous stem-cell transplantation (SCT) in patients with R/R DLBCL, R/R transformed FL, or R/R Grade 3b FL
- Prior allogeneic SCT
- Prior solid organ transplantation
- Patients with known or suspected history of hemophagocytic lymphohistiocytosis (HLH)
- Patients with history of confirmed progressive multifocal leukoencephalopathy (PML)
- History of severe allergic or anaphylactic reactions to monoclonal antibody therapy (or recombinant antibody-related fusion proteins)
- History of other malignancy that could affect compliance with the protocol or interpretation of results
    - Patients with a history of curatively treated basal or squamous cell carcinoma of the skin or in situ carcinoma of the cervix are allowed.
    - Patients with a malignancy that has been treated with curative intent will also be allowed if the malignancy has been in remission without treatment for ≥ 2 years prior to first study treatment administration.
- Current or past history of CNS lymphoma
- Current or past history of CNS disease, such as stroke, epilepsy, CNS vasculitis, or neurodegenerative disease
    - Patients with a history of stroke who have not experienced a stroke or transient ischemic attack in the past 2 years and have no residual neurologic deficits as judged by the investigator are allowed.
    - Patients with a history of epilepsy who have had no seizures in the past 2 years while not receiving any anti-epileptic medications are allowed in the expansion cohorts only.
- Significant cardiovascular disease such as New York Heart Association Class III or IV cardiac disease, myocardial infarction within the last 6 months, unstable arrhythmias, or unstable angina
- Significant active pulmonary disease (e.g., bronchospasm and/or obstructive pulmonary disease)
- Known active bacterial, viral, fungal, mycobacterial, parasitic, or other infection (excluding fungal infections of nail beds) at study enrollment, or any major episode of infection requiring treatment with IV antibiotics or hospitalization (relating to the completion of the course of antibiotics) within 4 weeks prior to first study treatment administration
- Known or suspected chronic active EBV infection
- Recent major surgery within 4 weeks prior to first study treatment administration
    - Protocol-mandated procedures (e.g., tumor biopsies and bone marrow biopsies) are permitted.
- Positive test results for chronic hepatitis B infection (defined as positive hepatitis B surface antigen [HBsAg] serology)
    - Patients with occult or prior hepatitis B infection (defined as positive total hepatitis B core antibody and negative HBsAg) may be included if HBV DNA is undetectable at the time of screening. These patients must be willing to undergo monthly DNA testing and appropriate antiviral therapy as indicated.
- Acute or chronic HCV infection
    - Patients who are positive for HCV antibody must be negative for HCV by PCR to be eligible for study participation.
- Administration of a live, attenuated vaccine within 4 weeks before first dose of study treatment administration or anticipation that such a live, attenuated vaccine will be required during the study

  - Patients must not receive live, attenuated vaccines (e.g., FluMist®) while receiving study treatment and after the last dose until B-cell recovery to the normal ranges. Killed vaccines or toxoids should be given at least 4 weeks prior to the first dose of study treatment to allow development of sufficient immunity.
  - Inactivated influenza vaccination should be given during local influenza season only.
  - Investigators should review the vaccination status of potential study patients being considered for this study and follow the U.S. Centers for Disease Control and Prevention guidelines for adult vaccination with any other non-live vaccines intended to prevent infectious diseases prior to study.
- History of autoimmune disease, including, but not limited to, myasthenia gravis, myositis, autoimmune hepatitis, systemic lupus erythematosus, rheumatoid arthritis, inflammatory bowel disease, vascular thrombosis associated with antiphospholipid syndrome, Wegener granulomatosis, Sjögren syndrome, Guillain-Barré syndrome, multiple sclerosis, vasculitis, or glomerulonephritis
  - Patients with a history of autoimmune-related hypothyroidism on a stable dose of thyroid replacement hormone may be eligible.
  - Patients with controlled Type 1 diabetes mellitus who are on an insulin regimen are eligible for the study.
  - Patients with a history of disease-related immune thrombocytopenic purpura, autoimmune hemolytic anemia, or other stable autoimmune diseases may be eligible.
- Received systemic immunosuppressive medications (including, but not limited to, cyclophosphamide, azathioprine, methotrexate, thalidomide, and anti-tumor necrosis factor agents) with the exception of corticosteroid treatment ≤10 mg/day prednisone or equivalent within 2 weeks prior to first dose of study treatment
  - The use of inhaled corticosteroids is permitted.
  - The use of mineralocorticoids for management of orthostatic hypotension is permitted.
  - The use of physiologic doses of corticosteroids for management of adrenal insufficiency is permitted.
  - *Patients who received acute, low-dose, systemic immunosuppressant medications (e.g., single dose of dexamethasone for nausea or B symptoms) may be enrolled.*
- Clinically significant history of liver disease, including viral or other hepatitis, current alcohol abuse, or cirrhosis
- Any serious medical condition or abnormality in clinical laboratory tests that, in the investigator's judgment, precludes the patient's safe participation in and completion of the study, or which could affect compliance with the protocol or interpretation of results

End of Study

The end of this study is defined as the date when the last patient, last visit occurs, or the date at which the last data point required for statistical analysis or safety follow-up is received from the last patient, whichever occurs later. The end of the study is expected to occur approximately 30 months after the last patient is enrolled.

Length of Study

The total length of the study, from screening of the first patient to the end of the study, is expected to be approximately 80 months.

<u>Investigational Medicinal Products</u>

The investigational medicinal products (IMPs) for this study are mosunetuzumab, polatuzumab vedotin, rituximab, and tocilizumab.

Mosunetuzumab will be administered either by IV infusion (Groups A, B, C; Arms I and J) or SC injection (Arms K and L) using standard medical syringes, syringe pumps or IV bags where applicable, in combination with polatuzumab vedotin. Flat dosing independent of body weight will be used for mosunetuzumab. The dose of mosunetuzumab for each patient will depend on their dose level assignment as detailed in the protocol.

Polatuzumab vedotin will be administered by IV infusion in combination with mosunetuzumab *or rituximab*. The dose of polatuzumab vedotin for each patient will be 1.8 mg/kg.

Rituximab (375 mg/m²) will be administered by IV infusion in combination with polatuzumab vedotin (Arm M). No dose modifications of rituximab are allowed.

Tocilizumab should be administered when necessary, as described in the protocol.

See the protocol for additional information on study treatment dosage, administration, and compliance, as well details regarding study treatment regimens.

Statistical Methods

Efficacy Analysis

For Arms I, J and K, *L and M* the population for the efficacy analyses will consist of all treated patients, with patients grouped according to their *received treatment*.

The efficacy analyses in the Phase Ib dose-finding portion of the study are exploratory. Descriptive summary statistics will be provided for all efficacy endpoints.

Primary Efficacy Endpoint

The primary efficacy endpoint for Arms I, J, K, L, and M is best objective response rate (ORR), defined as the percentage of patients with CR or PR at any time based on PET-CT and/or CT scan and as determined by the IRC using the Lugano 2014 criteria. Patients with missing or no response assessments will be classified as non-responders. The best ORRs between Arms L and M will be compared approximately 6 months after the last patient is enrolled in the randomized Phase II portion. *A best ORR delta of ≥10% between Arm L over Arm M would demonstrate clinical benefits of mosunetuzumab in combination with polatuzumab vedotin over rituximab in combination with polatuzumab vedotin.*

Comparison with respect to ORR between the treated patient population and historical controls will be tested for Arm J (R/R DLBCL/trFL/FL3b) and Arm K (R/R MCL). The control ORR is assumed to be 42% for Arm J and 30% for Arm K. The following hypothesis will be tested at a one-sided 0.025 level of significance using an exact binomial test in *Arm* J cohort:

Ho: ORR =42% versus Ha: ORR =42%.

The following hypothesis will be tested at a one-sided 0.025 level of significance using an exact binomial test in Arm K cohort:

Ho: ORR = 30% versus Ha: ORR > 30%

The ORR will be estimated and the corresponding Clopper-Pearson exact 95% CI will be constructed for each treatment arm.

Determination of Sample Size

The sample size for the Phase Ib dose-finding portion of the study is based on the dose-escalation rules described in the protocol. The planned enrollment for the dose-finding phase is approximately 9–42 patients. After the RP2D has been determined for mosunetuzumab combined with polatuzumab vedotin, a total of approximately [redacted] patients will be enrolled in the Phase II single-arm expansion portion of the study (100 patients in the R/R DLBCL cohort, [redacted] patients in the R/R FL cohort, and [redacted] patients in the R/R MCL cohort). Approximately [redacted] patients with R/R DLBCL will also be enrolled in the Phase II randomized portion of the study.

The primary efficacy endpoint for the Phase II expansion is best ORR as determined by PET-CT and/or CT scan as assessed by the IRC (Arm I, J, K, L and M). The primary analysis will be estimation of best ORR in patients treated with mosunetuzumab in combination with polatuzumab vedotin in the R/R DLBCL cohort (Arm J and L), in patients treated with mosunetuzumab in combination with polatuzumab vedotin in the R/R FL cohort (Arm I), in patients treated with mosunetuzumab in combination with polatuzumab vedotin in the R/R MCL cohort (Arm K), and in patients treated with rituximab in combination with polatuzumab vedotin in the R/R DLBCL cohort (Arm M).

With [redacted] patients in a treatment arm, the 95% exact Clopper-Pearson CIs for estimation of the true ORR rate would have a margin of error not exceeding ±16.7%, ±11.6%, or 10.3% respectively. The Clopper-Pearson exact 95% CIs corresponding to observed ORR ranging from 30% to 80% based on sample sizes of 40, 80, and 100.

For the R/R DLBCL/trFL/FL3b expansion cohort of Arm J, with observed ORR of 65%, a sample size of 100 patients will result in 95% CI of (55%, 74%; i.e., a true ORR of 42% is ruled out). Additionally, the planned sample size of 100 patients will provide more extensive safety data and 99% power to detect a difference in ORR, with a two-sided significance level of 5%.

For the R/R DLBCL randomized Phase II portion Arms L and M, the margin of error for 95% exact Clopper-Pearson CIs for estimation of the true ORR would not exceed ±16.7% *and ±11.6%*, with [redacted] patients in each arm*, respectively*.

With respect to assessment of safety, point estimates will be presented. The protocol provides probabilities of seeing at least one adverse event among [redacted] patients for true adverse event frequencies ranging from 1%–20%. For example, with [redacted] patients in a treatment arm, there is at least an 87% chance of observing at least one adverse event with true incidence of ≥ 5%.

Interim Analyses

Planned Interim Safety Analyses

The IMC will review all cumulative safety data by cohort and by treatment arm on a periodic basis during the Phase II expansion portion of the study, occurring when approximately [redacted] and [redacted] total patients in the Phase II single arm expansion cohorts have each received at least 2 cycles of treatment, or more frequent as indicated or requested by the Medical Monitor. The IMC may make recommendations regarding study conduct, including but not limited to, the following: performing additional safety analyses, amending the study protocol, holding patient enrollment pending further safety evaluations, holding/discontinuing study treatment, making decisions to modify or discontinue the requirement for hospitalization with study treatment, or terminating the study. Any outcomes of these safety reviews that affect study conduct will be communicated in a timely manner to the investigators for notification of the IRB/EC. A detailed plan will be included in the IMC Charter.

Interim Futility Analysis

An interim futility analysis will be conducted in Arm K. If the interim analysis, which will be conducted at least 3 months after approximately [redacted] patients have been dosed *with at least one dose of study treatment*, demonstrates a posterior probability of ≤ 30% that the true investigator-assessed ORR is > 30% (e.g., observing no more than 4 [20%] responders in [redacted] patients), enrollment in Arm K may be stopped.

An interim futility analysis will be conducted in Arms L and M. If the interim analysis, which will be conducted at least 3 months after approximately [redacted] patients in Arm M have been dosed with at least one dose of study treatment, demonstrates a posterior probability of > 85% that the true investigator-assessed ORR in Arm M is > 42% (e.g., observing at least [redacted] responders in [redacted] patients), enrollment in Arms L and M may expand to [redacted] patients total ([redacted] patients in each arm). Otherwise, if the interim analysis demonstrates a posterior probability of ≤ 85% that the true investigator-assessed ORR in Arm M is > 42% (e.g., observing no more than [redacted]esponders in [redacted] patients), enrollment in Arms L and M may be stopped at [redacted] patients total ([redacted] patients in each arm).

LIST OF ABBREVIATIONS AND DEFINITIONS OF TERMS

Abbreviation	Definition
2L+	second line or later
acMMAE	antibody-conjugated mono-methyl auristatin E
ADA	anti-drug antibody
ADC	antibody-drug conjugate
ALK	anaplastic lymphoma kinase
ASTCT	American Society for Transplantation and Cellular Therapy
AUC	area under the concentration–time curve
BR	Bendamustine *plus rituximab*
BSA	body surface area
BTK	Bruton's tyrosine kinase
C	Cycle
CAR	chimeric antigen receptor
CCOD	*clinical cut-off date*
CIRS-G	*Cumulative Illness Rating Scale-Geriatric*
CHOP	cyclophosphamide, doxorubicin, vincristine, and prednisone
CHP	cyclophosphamide, doxorubicin, and prednisone
CL	clearance
CLL	chronic lymphocytic leukemia
C_{max}	maximum serum concentration
C_{min}	minimum serum concentration
CMV	cytomegalovirus
COVID-19	*coronavirus disease 2019*
CR	complete response
CRO	contract research organization
CRS	cytokine release syndrome
Cru	complete response, unconfirmed
CT	computed tomography (scan)
CTCAE	Common Terminology Criteria for Adverse Events

Abbreviation	Definition
CVP	cyclophosphamide, vincristine, and prednisone
D	Day
DI-CCNAE	driving-impacting cognition or consciousness neurologic events
DL	dose level
DLBCL	diffuse large B-cell lymphoma
DLT	dose-limiting toxicity
DOR	duration of response
EBV	Epstein-Barr virus
EC	Ethics Committee
ECOG PS	Eastern Cooperative Oncology Group Performance Status
eCRF	electronic Case Report Form
EDC	electronic data capture
EFS	event-free survival
EOI	end of infusion
EQ-5D-5L	EuroQol 5-Dimension, 5-Level (questionnaire)
E.U.	*European Union*
EZH2	enhancer of Zeste 2 Polycomb Repressive Complex 2 Subunit
Fc	fragment crystallizable
FDA	(U.S.) Food and Drug Administration
FIL	*Fondazione Italiana Linfomi*
FL	follicular lymphoma
FLIPI	Follicular Lymphoma International Prognostic Index
G-CSF	granulocyte colony stimulating factor
GELF	Groupe d'Etude des Lymphomes Folliculaires (criteria)
GHS	good health status
GMR	geometric mean ratio
HBcAb	hepatitis B core antibody
HBsAg	hepatitis B surface antigen
HBV	hepatitis B virus
HCV	hepatitis C virus
HIPAA	Health Insurance Portability and Accountability Act
HLH	hemophagocytic lymphohistiocytosis
HR	*hazard ratio*
HRQoL	health-related quality of life
ICH	International Council for Harmonisation

Abbreviation	Definition
IFN-γ	interferon-γ
IHC	immunohistochemistry
IL	interleukin
IMC	Internal Monitoring Committee
IMP	investigational medicinal product
IND	Investigational New Drug (Application)
IPI	International Prognostic Index
IRB	Institutional Review Board
IRC	Independent Review Committee
IRR	infusion-related reaction
IxRS	interactive voice or web-based response system
Len	lenalidomide
Len+R	lenalidomide plus rituximab
LDH	lactate dehydrogenase
LFT	liver function test
MAS	macrophage activation syndrome
MCL	mantle cell lymphoma
MIPI	mantle cell lymphoma international prognostic index
MMAE	mono-methyl auristatin E
MRI	magnetic resonance imaging
MTD	maximum tolerated dose
NALT	new anti-lymphoma treatment
NCCN	National Comprehensive Cancer Network
NCI	National Cancer Institute
NCI CTCAE v5.0	National Cancer Institute Common Terminology Criteria for Adverse Events, Version 5.0
NGS	next-generation sequencing
NHL	non-Hodgkin lymphoma
NK	natural killer (cell)
NOS	not otherwise specified
ORR	objective response rate
OS	overall survival
PBMC	peripheral blood mononuclear cell
PCR	polymerase chain reaction
PD	pharmacodynamic
PE	polyethylene
PET	positron emission tomography (scan)

Abbreviation	Definition
PET-CR	complete response based on positron emission tomography scan
PET-CT	positron emission tomography–computed tomography (scan)
PFS	progression-free survival
PK	pharmacokinetic
PML	progressive multifocal leukoencephalopathy
popPK	population pharmacokinetics
PP	polypropylene
PR	partial response
PRA	primary response assessment
PRO	patient-reported outcome
PUR	polyurethane
PVC	polyvinyl chloride
Q3W	every 3 weeks
QoL	quality of life
QTcF	QT interval corrected through use of Fridericia’s formula
RBR	Research Biosample Repository
R-CHOP	rituximab plus cyclophosphamide, doxorubicin, vincristine, and prednisone
R-CHP	rituximab plus cyclophosphamide, doxorubicin, and prednisone
R-CVP	rituximab plus cyclophosphamide, vincristine, and prednisone
R-DHAP	rituximab plus dexamethasone, cytosine arabinoside, and cisplatin
R-ICE	rituximab plus ifosfamide, carboplatin, and etoposide
RP2D	recommended Phase II dose
R/R	relapsed or refractory
SARS-CoV-2	*severe acute respiratory syndrome coronavirus 2*
SCT	stem-cell transplantation
SD	stable disease
sGA	*simplified geriatric assessment*
SLL	*small lymphocytic lymphoma*
TDB	T-cell–dependent bispecific (antibody)
TK	toxicokinetic
TLS	tumor lysis syndrome
TNF-α	tumor necrosis factor-α

Abbreviation	Definition
ULN	upper limit of normal
U.S.	*United States*
USPI	U.S. Package Insert
V_{ss}	volume of distribution at steady state
WES	whole exome sequencing
WGS	whole genome sequencing
XPO1	exportin 1

1. BACKGROUND

1.1 BACKGROUND ON B-CELL LYMPHOMAS

Non-Hodgkin lymphoma (NHL) is the most common hematologic malignancy in adults. The majority of NHLs are of B-cell origin, with multiple different histologic subtypes that confer different clinical outcomes. Generally, NHL can be divided into aggressive and indolent lymphomas, which reflects their clinical course. Diffuse large B-cell lymphoma (DLBCL), the most common histologic subtype of NHL, accounts for approximately 31% of NHL cases and is classified as an aggressive NHL. Follicular lymphoma (FL) is the second most common histologic subtype of NHL, accounts for approximately 22% of NHL cases, and is classified as an indolent lymphoma (Armitage and Weisenburger 1998).

Regardless of the biologic and clinical heterogeneity of B-cell lymphomas, patients with advanced stage B-cell malignancies are typically initially treated with intensive cytotoxic chemotherapy combined with monoclonal antibodies, such as the anti-CD20 monoclonal antibody, rituximab (Rituxan®, MabThera®). Although durable responses can be achieved in some patients, the majority of patients will ultimately experience progressive or relapsed disease. Indolent B-cell malignancies, including FL, as well as approximately half of all aggressive lymphomas, remain incurable despite advances in immunochemotherapy that have resulted in longer progression-free survival (PFS) (Coiffier et al. 2002; Feugier et al. 2005; Hiddemann et al. 2005; Vidal et al. 2012). Moreover, as NHL is frequently diagnosed in older patients, the ability to tolerate cytotoxic chemotherapy is a major barrier to treatment success. Consequently, there remains a need for novel treatments that can significantly extend disease-free survival and overall survival (OS), while providing at least acceptable, if not superior safety and tolerability.

Recent developments have supported the effectiveness of therapies that utilize T cells in the treatment of B-cell malignancies. One approach involves the ex vivo manipulation of autologous or allogeneic T cells to express chimeric antigen receptors (CARs) that target lineage-specific surface molecules such as CD19. Anti-CD19 CAR-expressing T cells produced deep and durable responses in patients with relapsed or refractory (R/R) leukemias (Kochenderfer et al. 2012; Grupp et al. 2013). However, toxicities related to severe cytokine release syndrome (CRS), optimization of dose and schedule, and scalability of production to the broader cancer population constitute significant barriers to their clinical development. A second approach of T-cell–directed therapy involves the use of bispecific molecules that directly engage endogenous T cells with tumor cells. In hematologic malignancies, this approach has been demonstrated by the bispecific T-cell engager molecule, blinatumomab (Bargou et al. 2008), a 55-kDa fusion protein derived from two single-chain peptides recognizing CD19 and CD3. The mechanism of action of blinatumomab is the redirected lysis of $CD19^+$ B cells by T cells. Evidence of potent clinical activity of blinatumomab in B-cell malignancies has been reported in patients with R/R NHL and leukemia (Bargou et al. 2008; Viardot et al. 2010, 2011).

However, due to its size and structure, blinatumomab has a half-life of approximately 2 hours in humans, necessitating its administration by continuous infusion over a 4–8 week period (Nagorsen et al. 2012).

1.1.1 Treatment for Relapsed or Refractory DLBCL/Transformed FL

Diffuse large B-cell lymphoma is the most common aggressive form of NHL (Armitage and Weisenburger 1998). Additionally, each year around 3% of FLs transform into higher-grade NHL, most commonly DLBCL (Lossos and Gascoyne 2011), leading to almost a third of histologic transformation in 10 years. These patients with DLBCL, transformed from a previous FL histology, are treated with the same standard therapies as high-grade lymphomas.

The use of immunochemotherapy, most commonly rituximab plus cyclophosphamide, doxorubicin, vincristine, and prednisone (R-CHOP) for newly diagnosed DLBCL, led to a significant improvement in survival in patients in all age groups. In older patients ($>$60 years of age), R-CHOP was associated with a 2-year event-free survival (EFS) rate of 57% and a 10-year survival rate of 43.5% (Coiffier et al. 2010). In younger patients (18–60 years of age) with favorable prognostic features, R-CHOP demonstrated a 3-year EFS rate of 79% and a survival rate at 3 and 6 years of 93% and 74.3%, respectively (Pfreundschuh et al. 2011). However, nearly 40% of patients with DLBCL will eventually die of relapsed disease or disease that is refractory to first-line treatment. Patients with a high-risk International Prognostic Index (IPI) have a 5-year PFS rate of only 40% following treatment with R-CHOP (Zhou et al. 2014).

Specific molecular subsets of DLBCL are associated with an inferior outcome following R-CHOP therapy. Germinal center B-cell–like DLBCL had a better prognosis than activated (non-germinal) B-cell–like DLBCL, with a 3-year survival rate of 84% versus 56%, respectively ($p < 0.001$) (Lenz et al. 2008). Several genetic abnormalities predictive of poor outcome have been identified in DLBCL, including MYC rearrangement, BCL2 and BCL6 overexpression, and TP53 mutations. Rearrangement in MYC (MYC-positive DLBCL) has been reported in 9%–17% of DLBCL cases and often correlates with the germinal center B-cell–like DLBCL phenotype (Savage et al. 2009; Barrans et al. 2010). DLBCL treated with R-CHOP has a markedly worse 5-year survival rate in patients with MYC-positive DLBCL compared with MYC-negative DLBCL (33% vs. 72%) (Savage et al. 2009). Concurrent MYC and IGH-BCL2 rearrangement ("double-hit" DLBCL), observed in 2%–11% of patients with DLBCL, represents a DLBCL subset with an inferior outcome (5-year PFS of 18%; 5-year survival of 27%) (Savage et al. 2009; Dunleavy et al. 2014). Mutations in TP53 have been described in approximately 20% of patients with DLBCL and are strong predictors of poor OS (Young et al. 2008; Xu-Monette et al. 2012).

Second-line treatments of DLBCL/transformed FL consist of high-dose chemotherapy regimens such as rituximab plus ifosfamide, carboplatin, and etoposide (R-ICE)

or rituximab plus dexamethasone, cytosine arabinoside, and cisplatin (R-DHAP) followed by autologous stem-cell transplantation (SCT). More than half of the patients do not achieve a complete remission after treatment with these regimens (Gisselbrecht et al. 2010). Moreover, elderly patients or patients with comorbidities are often deemed ineligible for this aggressive therapy.

Furthermore, since the introduction of the monoclonal anti-CD20 antibody rituximab, it becomes more challenging to find effective therapies for patients with R/R DLBCL and prior exposure to rituximab. In these patients, the disease prognosis remains dismal and the complete remission rates achieved with currently approved agents or investigational agents remain low (see Table 1).

Table 1 Summary of Clinical Trial Data in Patients with Relapsed or Refractory DLBCL/Transformed FL

Therapy Regimen (n = patient number for efficacy assessment)	ORR (%)	CR (%)	Median PFS, months	Median OS, months	NHL Response Criteria
Rituximab plus gemcitabine and oxaliplatin (n = 48) [a]	55%	CR/CRu = 44%	5.0	11.0	International Working Group Criteria (Cheson et al. 1999)
Pixantrone (n = 64) [b]	41%	CR/CRu = 23%	5.3	10.2	International Working Group Criteria (Cheson et al. 1999)
Rituximab plus bendamustine (n = 137) or rituximab plus gemcitabine (n = 35) [c]	44%	CR/CRu = 16%	3.5	9.5	Cheson et al. 2007
Rituximab plus inotuzumab ozogamicin (n = 166) [c]	41%	CR/CRu = 16%	3.7	9.5	Cheson et al. 2007
Blinatumomab (n = 21) [d]	43%	CR = 19%	3.7	5.0	Cheson et al. 2007
Blinatumomab (n = 11) [e]	55%	CR/CRu = 36%	–	–	International Working Group Criteria (Cheson et al. 1999)
Axicabtagene ciloleucel (n = 101) [f]	72%	CR = 51%	5.8	Not reached	Cheson et al. 2007
Polatuzumab vedotin plus bendamustine and rituximab (n = 40) [g]	Best ORR 70%	Best CR = 58%	9.5	12.4	Cheson et al. 2014, modified

Table 1 Summary of Clinical Trial Data in Patients with Relapsed or Refractory DLBCL/Transformed FL (cont.)

Therapy Regimen (n = patient number for efficacy assessment)	ORR (%)	CR (%)	Median PFS, months	Median OS, months	NHL Response Criteria
Tisagenlecleucel (n = 93) [h]	52%	40%	DOR not reached	12.0	Cheson et al. 2014
Tafasitamab and lenalidomide (n = 80) [i]	60%	43%	21.7	Not reached	Cheson et al. 2014
Selinexor (n = 127) [j]	29%	13%	2.6	9.1	Cheson et al. 2014
Lisocabtagene maraleucel (n = 256) [k]	73%	53%	6.8	21.1	Cheson et al. 2014
Loncastuximab tesirine (n = 145) [l]	48%	24%	4.9	9.9	Cheson et al. 2014

CR = complete response as the best response; CRu = unconfirmed complete response as the best response; DLBCL = diffuse large B-cell lymphoma; DOR = duration of response; FL = follicular lymphoma; NHL = non-Hodgkin lymphoma; ORR = objective response rate; USPI = U.S. Package Insert.

[a] Mounier et al. 2013.

[b] Pettengell et al. 2012. Includes 53 patients with DLBCL, 10 patients with transformed indolent lymphoma, and 1 patient with Grade 3 FL.

[c] Dang et al. 2017.

[d] Viardot et al. 2016.

[e] Goebeler et al. 2016.

[f] Yescarta USPI; Neelapu et al. 2016.

[g] Sehn et al. 2020.

[h] Schuster et al. 2019.

[i] Salles et al. 2020.

[j] Kalakonda et al. 2020.

[k] Abramson et al. 2020.

[l] Caimi et al. 2021.

Several novel therapeutic agents have been approved for the management of R/R DLBCL in some countries.

Polatuzumab vedotin, an antibody-drug conjugate (ADC) targeting CD79b: In a randomized, Phase II portion of Study GO29365, polatuzumab vedotin in combination with bendamustine (BR) (n = 40) showed better efficacy than BR alone (n = 40) in transplant-ineligible participants with R/R DLBCL marked by a CRR at the end of the treatment (40.0% vs. 17.5%; p = 0.026), PFS (median 9.5 months vs. 3.7 months; p < 0.001) both assessed by independent review comitte (IRC), and OS (median 12.4 months vs. 4.7 months; p = 0.002; Sehn et al. 2019). *In the Phase III study POLARIX (NCT03274492), polatuzumab vedotin combined with rituximab,*

cyclophosphamide, doxorubicin, and prednisone (pola-R-CHP) showed superior PFS compared to R-CHOP (hazard ratio [HR] 0.73; 95% CI: 0.57–0.95; p < 0.02). The 2-year PFS rate was 76.7% (95% CI: 72.7–80.8) with pola-R-CHP vs. 70.2% (95% CI: 65.8–74.6) with R-CHOP (Tilly et al. 2021). Polatuzumab vedotin in combination with R-CHP has been approved by the European Commission for the treatment of adult patients with previously untreated DLBCL.

Tafasitamab, a humanized anti-CD19 monoclonal antibody: tafasitamab in combination with lenalidomide was evaluated in 81 patients with R/R DLBCL after at least one line of therapy. The best response rate was 60%, including a best CRR of 43%, with a median duration of response (DOR) of 21.7 months (Salles et al. 2020). While the response rates and the duration of therapy were clinically significant, the study excluded or limited enrollment of a very high-risk population such as participants with primary refractory disease and double-hit lymphoma.

Selinexor, an exportin 1 (XPO1) inhibitor: Selinexor was evaluated in 127 patients with R/R DLBCL (Kalakonda et al. 2020). Grade 3/4 thrombocytopenia was observed in 46% of participants. Objective response rate was 29%, including a CRR of 13%. Although this is an oral regimen with significant convenience, the untrivial myelosuppression and the low response rates may limit the actual use of this regimen in this setting.

Loncastuximab tesirine, an ADC targeting CD19: loncastuximab tesirine was evaluated in 145 patients with R/R DLBCL after at least one line of therapy (Caimi *et al.* 2021). The best response rate was 48.3%, including a best CRR of 24.1%, with a median DOR of 10.3 months.

In addition, CD19-directed chimeric antigen receptor (CAR) T-cell therapies have become available in some countries for use in the third-line or later setting (axicabtagene ciloleucel, tisagenlecleucel, and lisocabtagene maraleucel). Although these therapies have shown efficacy with durable complete responses (CRs), their use may be limited for the general population with R/R DLBCL due to the toxicity profile which requires carefully selected participants and treatment in centers with specially trained staff. In addition, the waiting period associated with CAR T-manufacture may be prohibitive in participants with rapidly progressing disease.

Despite the above-mentioned improvement in the therapeutic approaches in R/R DLBCL, the clinical outcome of patients with this condition, especially those who are not eligible for transplant, is still poor. Of note, tafasitamab, loncastuximab tesirine and CAR-T products target the same antigen, CD19, raising the concern for potential cross resistance. Taken together, there remains a significant unmet medical need for patients with transplant-ineligible R/R DLBCL.

1.1.2 Treatment for Relapsed or Refractory FL

Follicular lymphoma remains an incurable disease with currently available therapies. The addition of rituximab, an anti-CD20 monoclonal antibody, to induction chemotherapy, including cyclophosphamide, doxorubicin, vincristine, and prednisone (CHOP); cyclophosphamide, vincristine, and prednisone (CVP); fludarabine; or bendamustine, followed by rituximab maintenance therapy, are common first-line therapies (NCCN 2018). More recently, a comparison of rituximab-based chemoimmunotherapy with obinutuzumab-based chemoimmunotherapy demonstrated improved PFS with obinutuzumab, suggesting that more potent targeting of CD20 may result in more durable responses in first-line treatment (Marcus et al. 2017).

Despite significant therapeutic progress with the use of immunochemotherapy as first-line treatment, most patients will eventually relapse. Relapses are characterized by increasing refractoriness and decreasing DOR to subsequent lines of therapy. Treatment options for R/R FL include lenalidomide with rituximab, as well as immunochemotherapy as first-line therapy with rituximab or obinutuzumab (NCCN 2018; also see Table 2). In patients with rituximab-refractory indolent NHL, obinutuzumab plus bendamustine followed by obinutuzumab maintenance demonstrated improved PFS and OS compared with bendamustine alone (Sehn et al. 2016; Cheson et al. 2018).

The Food and Drug Administration (FDA) *previously* granted accelerated approval to idelalisib (Zydelig®), copanlisib (Aliqopa™), duvelisib (Copiktra®), and umbralisib (Ukoniq®) as single agents for the treatment of patients with relapsed FL who have received at least two prior systemic therapies. These agents are inhibitors of phosphoinositide 3'-kinase targeting various isoforms. These agents result in partial, but not complete, responses in a majority of patients with R/R FL, and both are associated with a median PFS of less than 1 year in single-arm studies (see Table 2). Furthermore, these agents have been associated with significant toxicities in some patients. *In Quarter 4 2021, duvelisib (Copiktra®) was voluntarily withdrawn from the U.S. market for the FL indication due to lack of confirmation of clinical benefit, as the required confirmatory trial was never initiated by the Sponsor for business reasons. Similarly, in Quarter 1 2022, idelalisib (Zydelig®) was voluntarily withdrawn by the Sponsor in the United States for FL and small lymphocytic lymphoma (SLL) indications due to lack of confirmation of clinical benefit in patients with RR FL or SLL, citing challenges with recruitment to the accelerated approval post marketing requirement study. In parallel, umbralisib (Ukoniq®) accelerated approval was withdrawn by the Sponsor as recommended by the FDA due to safety concerns. Additional considerations related to these PI3K withdrawals from the U.S. market (including other PI3K inhibitors not mentioned within this context may still be on the market such as copanlisib [Aliqopa®]) are highlighted in the PI3K inhibitor class advisory committee meeting of non-FDA experts in April 2022, concerning trends in OS in multiple randomized controlled trials, toxicities of the PI3K inhibitor class, inadequate dose optimization, and trial*

design considerations regarding the limitations of single-arm trials (FDA Briefing Document, ODAC 2022).

Tazemetostat is a unique enhancer of zeste 2 polycomb repressive complex 2 subunit (EZH2) inhibitor, which received accelerated approval by U.S. FDA for patients with R/R FL with EZH2 mutation after at least two prior systemic therapies, and for patients with R/R FL with no satisfactory alternative treatment options. In patients with R/R FL with EZH2 mutation, objective response was observed in 69% including CR in 13%. Response was also observed in patients with FL without EZH2 mutation (Table 2). The treatment was relatively well tolerated.

Axicabtagene ciloleucel (Yescarta) recently received an accelerated approval for R/R FL. While the response rates are high and duration of response seems to be long (see Table 2), the use may be limited for the general population with R/R FL due to the toxicity profile which requires carefully selected participants and treatment in centers with specially trained staff.

In summary, multiply relapsed FL thus remains a disease with high unmet medical need for which improved therapies are needed.

Table 2 Summary of Clinical Trial Data in Patients with R/R FL

Therapy Regimen (n = patient number for efficacy assessment)	ORR (%)	CR (%)	Median DOR	Median PFS	Fatal and Serious Treatment-Emergent Adverse Events [a, b, i]
**Patients with indolent NHL treated with one or more prior lines of systemic therapy**					
Obinutuzumab plus bendamustine induction and obinutuzumab maintenance (n = 194) vs. bendamustine (n = 202) [i]	69%	17%	Median not estimated	25.8 months vs. 14.1 months, hazard ratio 0.57	Febrile neutropenia, 5.4%<br>Infusion-related reactions, 3.4%<br>Pneumonia, 3.4%
R-CHOP + observation (n = 91) [j]	85%	30%	Median not reported	27.0 months	–
R-CHOP + R-maintenance (n = 98) [j]				57.0 months	Grade 3–4 infection during R-maintenance, 9%
Lenalidomide + rituximab (n = 46) [k]	76%	39%	Median not reported	Median not reported	–
**Patients treated with two or more prior lines of systemic therapy**					
Idelalisib (n = 72) [c, d, g]	54%	8%	Median not evaluable	11.0 months	Hepatotoxicity, 11%–18%<br>Diarrhea/colitis, 14%–19%<br>Pneumonitis, 4%<br>Infections, 21%–36%<br>Intestinal perforation
Copanlisib (n = 104) [c, h]	59%	14%	12.2 months	11.2 months	Infections, 19%<br>Hyperglycemia, 41%<br>Hypertension, 26%<br>Pneumonitis, 5%<br>Neutropenia, 24%
Duvelisib (n =83) [e, l]	42.2%	1.2%	10 months (all iNHL)	9.8 months (all iNHL)	Grade ≥3 neutropenia 24.8%<br>Grade ≥3 diarrhea 14.7%<br>Grade ≥3 pneumonitis 7.80%<br>Grade ≥3 colitis 4.7%

Table 2 Summary of Clinical Trial Data in Patients with R/R FL (cont.)

Therapy Regimen (n = patient number for efficacy assessment)	ORR (%)	CR (%)	Median DOR	Median PFS	Fatal and Serious Treatment-Emergent Adverse Events [a, b, i]
Umbralisib (n = 117) [m, f]	45%	3%	Not reached	10.6 months	Grade ≥3 neutropenia 11.5%<br>Grade ≥3 diarrhea 10.1%<br>Grade ≥3 AST/ALT 7.2%<br>Grade ≥3 pneumonitis 1.0%<br>Grade ≥3 colitis 0.5%
Tazemetostat<br>EZH2 mut n = 45<br>EZH2 wt n = 54 [n]	69%<br>35%	13%<br>4%	10.9 months<br>13.0 months	13.8 months<br>11.1 months	Grade ≥3 thrombocytopenia 3%<br>Grade ≥3 neutropenia 3%<br>Grade ≥3 anemia 2%
Axi-cel (Yescarta) n = 84 [o]	94%	80%	Not reached	Not reached	Grade ≥3 CRS 6%<br>Grade ≥3 neurotoxicity 15%

CR = complete response as the best response; CRS = cytokine release syndrome; DOR = median of duration of response; EZH2 = enhancer of zeste 2 polycomb repressive complex 2 subunit; FL = follicular lymphoma; iNHL = indolent non-Hodgkin lymphoma; mut = mutation; NHL = non-Hodgkin lymphoma; ORR = objective response rate; PFS = progression-free survival; R = rituximab; R-CHOP rituximab plus cyclophosphamide, doxorubicin, vincristine, and prednisone; R/R = relapsed or refractory; USPI = U.S. Package Insert.

[a] Zydelig (idelalisib) USPI.

[b] Aliqopa (copanlisib) USPI.

[c] Accelerated approval was granted for this indication based on overall response rate. Continued approval for this indication may be contingent upon verification and description of clinical benefit in a confirmatory trial.

[d] *In Q1 2022, idelalisib was voluntarily withdrawn by the Sponsor in the United States for FL and SLL indications due to lack of confirmation of clinical benefit, See Section 1.1.2 for further details.*

[e] *In Q4 2021, duvelisib was voluntarily withdrawn from the U.S. market for the FL indication due to lack of confirmation of clinical benefit, See Section 1.1.2 for further details.*

[f] *Accelerated approval was withdrawn by the Sponsor due to safety concerns.*

[g] Gopal et al. 2014.

[h] Dreyling et al. 2017.

[i] Sehn et al. 2016; Cheson et al. 2018.

[j] van Oers et al. 2006; van Oers et al. 2010.

[k] Leonard et al. 2015.

[l] Flinn et al. 2019.

[m] Fowler et al. 2021.

[n] Morschhauser et al. 2020.

[o] Jacobson et al. 2020.

1.1.3 Treatment for Relapsed or Refractory Mantle Cell Lymphoma

Mantle cell lymphoma (MCL) is generally considered an incurable disease, and the majority of patients require systemic therapy. The most common front-line therapy is chemoimmunotherapy including an anti-CD20 antibody with or without consolidative autologous SCT. For RR MCL, non-chemotherapeutic agents have been evaluated and approved.

Bortezomib was initially indicated for use in patients with MCL who have received at least one previous therapy. Lenalidomide is approved for use in patients with MCL whose disease has relapsed or progressed after two previous therapies, one of which includes bortezomib. In addition, temsirolimus received a label in the treatment of adult patients with relapsed or refractory MCL by the EMA.

More recently, ibrutinib was granted accelerated approval by the FDA (2013) and a market authorization in the E.U. (2014) for the treatment of patients with MCL who have received at least one previous therapy based on response rate with DOR data obtained from a single-arm trial. Additional Bruton's tyrosine kinase (BTK) inhibitors (acalabrutinib and zanubrutinib) also received accelerated approval from the FDA based on response rate with DOR data obtained from single-arm trials. Given the favorable safety profile and efficacy in this disease, BTK inhibitors are now the most preferred choice in R/R MCL. This treatment generally is to be continued without a pre-specified duration *of* treatment, and options for patients with MCL whose disease progress after a treatment with BTK inhibitors are limited.

Immune engaging therapy targeting B-cell specific antigens have recently been investigated, such as the administration of T cells engineered ex vivo to recognize tumor cells (also known as CAR-T cells). Brexucabtagene autoleucel received accelerated approval in the U.S. in 2020, and a Positive Opinion has been adopted by *the European Commission* for patients with R/R MCL after BTK inhibitor therapy. The studied population was patients with relapsed or refractory MCL after BTK inhibitor therapy (Wang et al 2020). Although the data on CAR-T therapy is promising, it is associated with toxicities that constitute significant clinical risk, including CRS and neurologic toxicities, and ensuring the consistency, quality, and dose of autologous cell-based therapies remains non-trivial. Additionally, this potentially limits patients with progressive disease who may not have time to wait for CAR-T cell manufacturing.

Several studies evaluating the therapeutic effect of different regimens in patients with MCL after a BTK inhibitor are summarized in Table 3. With the exception of intensive chemotherapy and the above-mentioned CAR-T therapy which are suitable only for selected patients, the efficacy of subsequent treatment is limited, with complete response rates of 19% or less, and the prognosis is poor. Therefore, novel treatments with improved efficacy and tolerability are needed, particularly in patients with diseases that are resistant or refractory to BTK inhibitors.

Table 3 Summary of Retrospective Outcome Analysis in Patients with Relapsed or Refractory MCL after Treatment with BTK Inhibitor

Treatment	Number of Patients Median Prior Lines of Therapy (Range)	Efficacy	Notes
Various [a]	n = 31<br>Median 2<br>(range 1–8)	CR 19%<br>ORR 32%<br>Median DOR 6 months<br>Median OS 8.4 months	Single institution. Reason for discontinuation of ibrutinib included disease progression, toxicity, or elective SCT.
Various [b]	n = 73<br>Median 4<br>(range 1–13)	CR 7%<br>ORR 26%<br>Median DOR 4.6 months<br>PFS 1.9 months<br>Median OS 5.8 months	Across 15 institutions. Disease progressed while on ibrutinib.
Lenalidomide-containing regimen [c]	n = 58<br>Median 4<br>(range 1–13)	CR 14%<br>ORR 29%<br>Median DOR 5 months	In Len *monotherapy* or Len + R patients (n = 24), CR rate 4%, ORR 21%.
R-BAC [d]	n =36<br>Median 2<br>(range 1–6)	CR 60%<br>ORR 83%<br>Median PFS 10.1 months<br>Median OS 12.5 months	Selected patient population for intensive chemotherapeutic regimen with toxicity. Many bridged to SCT after this regimen.
Brexucabtagene autoleucel [e]	n = 60<br>Median 3<br>(range 1–5)	CR 67%<br>ORR 93%<br>12-months PFS 61%<br>12-months OS 83%	74 enrolled, 71 had CAR-T product, 68 received treatment. 91% CRS any grade, 15% Grade ≥ 3, 63% neurotoxicity any grade, 35% Grade ≥ 3

BTK = Bruton's tyrosine kinase; CAR-T =chimeric antigen receptor-modified T-cell therapy; CR = complete response; DOR = duration of response; Len = lenalidomide; Len+R = lenalidomide plus rituximab; MCL; ORR = objective response rate; OS = overall survival; PFS = progression-free survival; SCT = stem-cell transplantation.

[a] Cheah et al. 2015.

[b] Martin et al. 2015.

[c] Wang et al. 2017.

[d] McCulloch et al. 2020.

[e] Wang et al. 2020.

1.2 BACKGROUND ON MOSUNETUZUMAB

Mosunetuzumab (RO7030816; also known as BTCT4465A) is a full length, humanized anti-CD20/CD3 T-cell–dependent bispecific (TDB) antibody of an IgG1 isotype that is

produced using the knobs-into-holes technology (Atwell et al 1997; Spiess et al. 2013). One Fab region of the antibody is directed against the extracellular domain of the CD3ε subunit of the T-cell receptor complex and the other Fab region is directed against the extracellular domain of CD20 (Atwell et al 1997; Spiess et al. 2013). Mosunetuzumab contains the N297G amino acid substitution in the fragment crystallizable (Fc) region according to Eu numbering (Edelman et al. 1969; Kabat et al. 1991). This substitution results in a non-glycosylated heavy chain that has minimal binding to Fcγ receptors and, consequently, prevents Fc effector functions. Mosunetuzumab is derived from Chinese hamster ovary cells.

As a T-cell–recruiting bispecific antibody targeting CD20 expressing B cells, mosunetuzumab is a conditional agonist; target B-cell killing is observed only upon simultaneous binding to CD20 on B cells and CD3 on T cells. Engagement of both arms of mosunetuzumab results in the formation of an immunologic synapse between a target B cell and a cytotoxic T cell resulting in T-cell activation in a target- and dose-dependent manner. T-cell activation is manifested by the expression of activation-related surface markers (e.g., CD69 and CD25), transient release of cytokines (e.g., interferon-γ [IFN-γ], tumor necrosis factor-α [TNF-α], interleukins [IL]-2, -6, and -10), and robust T-cell proliferation. Subsequent directed release of perforin and a cocktail of granzymes from T cells through the immunologic synapse result in B-cell lysis.

Mosunetuzumab (Lunsumio®) as monotherapy has been conditionally approved by the European Commission for the treatment of adult patients with relapsed or refractory FL who have received at least two prior systemic therapies. Clinical development is ongoing for additional indications including aggressive NHL. Refer to local prescribing information for further details of approved use.

1.2.1 Nonclinical Studies with Mosunetuzumab

Comprehensive pharmacologic, pharmacokinetic/toxicokinetic (PK/TK), pharmacodynamic (PD), and toxicology studies were conducted to support the entry of mosunetuzumab into clinical trials and continued clinical development. Studies were conducted using mosunetuzumab or functionally related antibody clones. In vitro studies with human peripheral blood mononuclear cells (PBMCs) and B-cell lymphoma cell lines and in vivo studies in cynomolgus monkeys support the mechanism of action of mosunetuzumab-induced T-cell activation, cytokine release, and proliferation in the presence of CD20-positive target B-cells with subsequent killing of target cells. Single-dose and repeat-dose (up to 26-week) toxicity, PK/TK, and PD studies with mosunetuzumab following IV and/or SC administration in cynomolgus monkeys have been completed. The nonclinical PK behavior observed for mosunetuzumab is consistent with that expected for a humanized IgG1 monoclonal antibody with a component of target-mediated clearance (CL). The acute toxicities associated with mosunetuzumab treatment are largely driven by stimulation of T cells, as evidenced by PD changes in cytokine levels and activated T-cell numbers. In repeat-dose toxicity

studies in cynomolgus monkeys, the increase of cytokine levels, T-cell activation, and acute postdose observations were primarily limited to the first dose and were reduced or negligible following subsequent doses. Therefore, clinical strategies to control the extent of T-cell stimulation through alternative dose schedules or other prophylactic measures (such as pretreatment with corticosteroids) may be necessary to optimize the benefit-risk profile of mosunetuzumab when administered as a single-agent and in combination with other anti-lymphoma agents where the combinatorial safety profile is currently not known.

Additionally, subcutaneous administration of mosunetuzumab in cynomolgus monkeys was as effective as IV dosing at the same dose level in inducing B-cell depletion, effectively reducing cytokine release, eliminating acute toxicities, and reducing the incidence of vascular/perivascular findings in a repeat-dose study. This suggests that SC administration may be a viable approach to mitigate mosunetuzumab-related toxicities without compromising efficacy.

Refer to the Mosunetuzumab Investigator's Brochure for details on nonclinical studies.

1.2.2 Summary of Clinical Data with Mosunetuzumab

Evaluation of mosunetuzumab *monotherapy* was initiated in Study GO29781, an open-label, multicenter, Phase I/Ib trial evaluating the safety and pharmacokinetics of escalating doses of mosunetuzumab as a single agent and combined with atezolizumab in patients with R/R B-cell NHL and chronic lymphocytic leukemia (CLL). *The safety and efficacy of mosunetuzumab (IV or SC) in combination with polatuzumab vedotin was further evaluated in Studies GO40516 and GO40554.*

A summary of the clinical safety, efficacy, as well as clinical pharmacokinetics and immunogenicity data from GO29781, GO40516, and GO40554 is provided in the sections below.

1.2.2.1 Safety

A summary of the safety data from Studies GO29781, GO40516*, and GO40554 are* provided below. *The safety profile summary will focus on Group B11 and Group F of Study GO29781, Arm J of Study GO40516, and Cohort C of Study GO40554. Details of each treatment group:*

- *Group B11 of Study GO29781 is to evaluate the safety of mosunetuzumab IV using the recommended Phase II dose (RP2D) with a C1 step-up dose schedule at 1/2/60/30mg dose.*
- *Group F of Study GO29781 is to evaluate the safety of mosunetuzumab SC using two step-up dosing schedules, 5/15/45 mg (Group F1, 5 mg on C1D1; 15 mg on C1D8; 45 mg on C1D15) and 5/45/45 mg (Group F2, 5 mg on C1D1; 45 mg on C1D8; 45 mg on C1D15), to further reduce the rate and severity of CRS events. The starting dose of 5 mg was selected as the projected C_{max} is similar to the recommended initial dose of the IV step-up dosing regimen. The target dose of*

45 mg SC was selected as it is projected to provide a lower C_{max} but higher area under the concentration–time curve (AUC) at steady state than the 30 mg IV maintenance dose. The 5/45/45 mg dosing regimen showed comparable safety profiles to the 5/15/45 mg dosing regimen but with a shortened window of CRS associated mostly with only the first two doses of mosunetuzumab during Cycle 1. Further, the 5/45/45 mg dosing regimen also allows faster achievement of target dose. Therefore, this dose is considered the preferred dose for mosunetuzumab SC monotherapy.

- *Arm J of Study GO40516 is to evaluate the safety profile of mosunetuzumab IV in combination with polatuzumab vedotin in patients with R/R NHL. Mosunetuzumab used the RP2D dose of 1/2/60/30 mg Q3W and polatuzumab vedotin at 1.8 mg/kg up to 6 cycles.*
- *Cohort C of Study GO40554 is to evaluate safety of mosunetuzumab SC in combination with polatuzumab vedotin in patients with DLBCL following first-line immunochemotherapy and as monotherapy or in combination with polatuzumab vedotin in elderly/unfit patients with previously untreated DLBCL. The mosunetuzumab SC doses evaluated in this cohort are 5/15/45 mg and 5/45/45 mg up to 8 cycles Q3W and polatuzumab vedotin at 1.8 mg/kg up to 6 cycles.*

An overview of adverse events, including CRS, neutropenia and febrile neutropenia are summarized in Table 4, Table 5, Table 6, respectively. Most treatment emergent adverse events occurred during the first treatment cycle. The most common treatment-related adverse events are CRS and neutropenia for both IV and SC administration of mosunetuzumab. For further information on all treatment groups to date, refer to the current version of the Mosunetuzumab Investigator's Brochure.

Table 4 Overview of Adverse Events in Studies GO29781, GO40516 and GO40554

	GO29781Mono IV (1/2/60/30mg) Group B11 N=219	GO29781 Mono SC (5/15/45mg) or (5/45/45mg) Group F, N=119	GO40516 M (IV)+Pola Combo (1/2/60/30mg) Arm J, N=[redacted]	GO40554 M (SC)+Pola Combo (5/15/45mg) or (5/45/45mg) Cohort C, N=[redacted]
Number of Patients with ≥ 1 Adverse Event	215 (98.2%)	111 (93.3%)	[redacted] ([redacted]%)	[redacted] ([redacted]%)
Adverse events of Grade 3-5 (not including PD)	146 (66.7%)	55 (46.2%)	[redacted]	[redacted]
Adverse event leading to dose modification / interruption of mosunetuzumab	74 (33.8%)	25 (21.0%)	[redacted] ([redacted]%)	[redacted] ([redacted]%)
Adverse event leading to withdrawal of mosunetuzumab	10 (4.6%)	2 (1.7%)	[redacted]([redacted]%)	[redacted] ([redacted]%)
Mosunetuzumab-related adverse event	189 (86.3%)	99 (83.2%)	[redacted] ([redacted]%)	[redacted] ([redacted]%)
Mosunetuzumab-related adverse event leading to withdrawal of treatment	4 (1.8%)	0	[redacted] ([redacted]%)	[redacted]
Serious adverse event	115 (52.5%)	45 (37.8%)	[redacted]([redacted]%)	[redacted] ([redacted]%)
Mosunetuzumab-related serious adverse event	75 (34.2%)	26 (21.8%)	[redacted] ([redacted]%)	[redacted] ([redacted]%)
Mosunetuzumab related serious adverse event leading to withdrawal of treatment	4 (1.8%)	0	[redacted]	[redacted]

PD = progressive disease.

Table 5 CRS Events in Safety Evaluable Patients with R/R NHL Treated with Mosunetuzumab Either Monotherapy or Combination With Polatuzumab Vedotin

	Grade	GO29781 Mono IV (1/2/60/30mg) Group B11 N=219	GO29781 Mono SC (5/15/45mg) or (5/45/45mg) Group F, N=119	GO40516 M (IV)+Pola Combo (1/2/60/30mg) Arm J, N=[redacted]	GO40554 M (SC)+Pola Combo (5/15/45mg) or (5/45/45mg) Cohort C, N=[redacted]
ASTCT grade	Any	86 (39.3%)	29 (24.4%)	[redacted] ([redacted]%)	[redacted] ([redacted]%)
	1	49 (22.4%)	20 (16.8%)	[redacted] ([redacted]%)	[redacted] ([redacted]%)
	2	31 (14.2%)	8 (6.7%)	[redacted] ([redacted]%)	[redacted] ([redacted]%)
	3	5 (2.3%)	1 (0.8%)	[redacted] ([redacted]%)	[redacted] ([redacted]%)
	4	1 (0.5%)	0	[redacted]	[redacted]

ASTCT = American Society for Transplantation and Cellular Therapy; R/R/ NHL = relapsed or refractory non-Hodgkin lymphoma.

Table 6 Neutropenia and Febrile Neutropenia Events in Safety Evaluable Patients with R/R NHL Treated with Mosunetuzumab Either Monotherapy or Combination With Polatuzumab Vedotin

	Grade	GO29781 Mono IV (1/2/60/30mg) Group B11 N=219	GO29781 Mono SC (5/15/45mg) or (5/45/45mg) Group F, N=119	GO40516 M (IV)+Pola Combo (1/2/60/30mg) Arm J, N [redacted]	GO40554 M (SC)+Pola Combo (5/15/45mg) or (5/45/45mg) Cohort C, N [redacted]
Neutropenia	Any grade	60 (27.4%)	21 (17.6%)	[redacted] ([redacted]%)	[redacted] ([redacted]%)
	1	4 (1.8%)	1 (0.8%)	[redacted] ([redacted]%)	[redacted]
	2	3 (1.4%)	2 (1.7%)	[redacted] ([redacted]%)	[redacted] ([redacted]%)
	3	24 (11.0%)	9 (7.6%)	[redacted] ([redacted]%)	[redacted] ([redacted]%)
	4	29 (13.2%)	9 (7.6%)	[redacted] ([redacted]%)	[redacted]
Febrile neutropenia	Any grade	5 (2.3%)	2 (1.7%)	[redacted] ([redacted]%)	[redacted]
	3	5 (2.3%)	2 (1.7%)	[redacted] ([redacted]%)	[redacted]
	4	0	0	[redacted]	[redacted]

R/R/ NHL = relapsed or refractory non-Hodgkin lymphoma.

1.2.2.2 Efficacy

In Study GO29781, [redacted] patients with R/R B-cell NHL across histolog*ies* were treated with mosunetuzumab monotherapy *IV and* [redacted] *patients were treated with mosunetuzumab monotherapy SC*. In *response-*evaluable patients treated with mosunetuzumab IV (n = [redacted]), the objective response rate was [redacted]% including CR rate *of* [redacted]%. *In response-evaluable patients treated with mosunetuzumab SC (n =* [redacted]*, ongoing as of clinical cut-off date [CCOD] of* [redacted]*), the objective response rate was* [redacted] *% including CR rate of* [redacted] *%.*

In Study GO40516 in Arm J (mosunetuzumab IV 1/2/60/30 mg plus polatuzumab vedotin 1.8 mg/kg) for patients with R/R DLBCL (n = [redacted], ongoing as of *CCOD of* [redacted]), objective response was observed in [redacted]% *of patients* including CR in [redacted]% *of patients*.

Refer to the Mosunetuzumab Investigator's Brochure for more detailed information.

1.2.2.3 Clinical Pharmacokinetics and Immunogenicity

Clinical PK data from Group A (0.05 to 2.8 mg IV fixed dose, Q3W dosing), Group B (0.4/1/2.8 to 1/2/60 mg IV C1 step-up doses on D1/8/15, followed by Q3W dosing) and Group D (1.6 to 20 mg SC fixed dose, Q3W dosing) were analyzed in the ongoing Phase I/Ib (Study GO29781) study.

Following IV dosing, mosunetuzumab serum drug concentrations reach maximum serum concentration (C_{max}) at the end of infusion (approximately 4 hours) and decline in a *bi–*exponential fashion, with *an estimated apparent* half-life ($t_{1/2}$) of approximately *3* to *10* days. *The terminal half-life estimate was 16.1 days at steady state based on population pharmacokinetics (popPK) model simulations.* The PK exposure increased in an approximately dose proportional manner over the dose ranges tested. The popPK following IV administrations of mosunetuzumab was well described by a 2-compartment PK model with time-dependent clearance.

Following SC dosing, mosunetuzumab demonstrated a favorable SC PK profile with *a relatively* high bioavailability (*87%, estimated by the preliminary population PK model*) and a *median* time to maximum concentration [T_{max}] *of* approximately 3–8 days). The SC dose is *associated with* reduced C_{max} (approximately *60–80*% *reduction*), but similar exposure (AUC), when compared *to that following equivalent IV doses based on cross-group comparisons.* Additional information on mosunetuzumab clinical PK characteristics can be found in the Investigator's Brochure

In Study GO29781, ADAs to mosunetuzumab were detected in [redacted] patient out of [redacted] patients as of the ADA data cutoff date of [redacted].

Refer to the Mosunetuzumab Investigator's Brochure for details on clinical study results.

1.3 BACKGROUND ON POLATUZUMAB VEDOTIN

CD79b is a cell-surface antigen whose expression is restricted to all mature B cells except plasma cells. It is expressed in a majority of B-cell–derived malignancies, including nearly all NHLs and CLLs (Dornan et al. 2009). Relating specifically to DLBCL, CD79b is expressed by essentially all tumor cells (Olejniczak et al. 2006; Pfeifer et al. 2015), enabling its use as a target in all subtypes of DLBCL independent of dominant signaling pathways. Antibodies bound to CD79b are rapidly internalized, which makes CD79b ideally suited for targeted delivery of cytotoxic agents (Polson et al. 2007, 2009).

Polatuzumab vedotin (DCDS4501S) is an ADC that contains a humanized IgG1 anti-human CD79b monoclonal antibody (MCDS4409A) and a potent anti-mitotic agent, monomethyl auristatin E (MMAE), linked through a protease-labile linker, maleimidocaproyl-valine-citrulline-p-aminobenzyloxycarbonyl.

MMAE has a mode of action that is similar to that of all vinca-alkaloids, including vincristine, which is a component of standard chemotherapy (e.g., R-CHOP used for treatment of lymphoma). Following binding at the cell-surface epitope and internalization of the ADC by the targeted cell, MMAE is released following cleavage of the linker by lysosomal enzymes. MMAE then binds to tubulin and disrupts the microtubule network, resulting in inhibition of cell division and cell growth (Doronina et al. 2003). This therapeutic approach takes advantage of the specific targeting capability of the antibody, the cytotoxic activity of MMAE, and the increased potency of MMAE compared with vincristine. It is hypothesized that polatuzumab vedotin in combination with other novel agents will provide enhanced efficacy and safety to patients with NHL.

In an early study of polatuzumab vedotin (DCS4968g), [redacted] patients with R/R MCL were treated with polatuzumab vedotin (1.8mg/kg or 2.4mg/kg) either as a single agent or in combination with rituximab. [redacted] patients experienced a partial response (PR).

In some countries, polatuzumab vedotin has been approved for use in combination with bendamustine and rituximab for the treatment of adult patients with R/R DLBCL. *In addition, polatuzumab vedotin in combination with R-CHP has been approved for use by the European Commission for the treatment of adult patients with previously untreated DLBCL.* Refer to local prescribing information for further details of approved use.

Refer to the Polatuzumab Vedotin Investigator's Brochure for details on nonclinical and clinical studies.

1.4 STUDY RATIONALE AND BENEFIT-RISK ASSESSMENT

Progress has been made in the treatment of FL, DLBCL, and MCL; however, a significant number of patients will not be cured of the disease. Instead, they will experience relapse or death from progression or treatment-related toxicity. There is

a need for the continued development of safe and effective therapies for patients with disease that relapses or for those who develop refractory disease during or after first-line therapy.

The goals of this Phase Ib/II study are to assess the safety, tolerability, pharmacokinetics, pharmacodynamics, and efficacy of escalating doses of mosunetuzumab in combination with polatuzumab vedotin, in patients with R/R FL, DLBCL, and MCL expected to express CD20; to determine a RP2D and schedule of mosunetuzumab in combination with polatuzumab vedotin; and to evaluate the efficacy of mosunetuzumab in combination with polatuzumab vedotin. Given the relatively poor prognosis of patients with R/R FL, DLBCL, and MCL who have failed standard therapies, the promising anti-tumor activity observed with mosunetuzumab and polatuzumab vedotin individually, the potentially tolerable safety profile of this combination, and the safety-mitigation plan outlined in this protocol, the anticipated benefit-risk balance of this clinical study is acceptable. In addition, the randomized Phase II portion of the study is designed to allow assessment of the individual contribution of mosunetuzumab when given in combination with polatuzumab vedotin in R/R DLBCL (Section 3.3.8).

1.4.1 Rationale for Treatment of Mosunetuzumab in Combination with Polatuzumab Vedotin

There exists a strong rationale for testing the combination of the T-cell recruiting bispecific antibody mosunetuzumab with the ADC polatuzumab vedotin. Each agent targets a specific mechanistic node in the cancer immunity cycle (Chen and Mellman 2013): The T-cell recruiting bispecific facilitates forced recognition of tumor cells by T cells, while the ADC will induce tumor-cell killing leading to release of tumor-specific neo-antigens that may elicit additional anti-tumor adaptive immune responses. Each agent targets a different cell surface antigen (CD20, CD79b), which may mitigate against antigen-loss escape mechanisms of resistance to a single agent. Finally, each agent has shown promising single-agent anti-tumor activity in patients with R/R FL, DLBCL, and MCL (Palanca-Wessels et al. 2015; Mosunetuzumab Investigator's Brochure).

1.4.2 Rationale for Treatment of Rituximab in Combination with Polatuzumab Vedotin (Arm M)

The addition of anti-CD20 antibody to polatuzumab vedotin (Study GO27834) showed no new safety signals compared to polatuzumab vedotin monotherapy (Study DCS4968g), and demonstrated better CR rate (18% and 21% at polatuzumab vedotin 1.8 mg/kg and 2.4 mg/kg, respectively) compared to polatuzumab vedotin monotherapy (0% and 15%, respectively). This regimen has been utilized in clinical practice and is listed in National Comprehensive Cancer Network (NCCN) guidelines (NCCN Guideline Version 4.2021 for DLBCL) as one of the preferred regimens for the treatment of transplant ineligible patients with R/R DLBCL.

1.4.3 Benefit-Risk Assessment of Mosunetuzumab in Combination with Polatuzumab Vedotin

The Phase Ib part of this study was the first clinical evaluation of the combination of mosunetuzumab with polatuzumab vedotin (Group A). At the time of study initiation, no direct benefits were known, but the available nonclinical and clinical data provided a strong rationale for investigating the potential benefit of this combination in patients with R/R B-cell lymphoma. Consequently, the mosunetuzumab IV in combination with polatuzumab vedotin was generally well tolerated, and provided acceptable toxicity profile (Section 1.2.2.1) and promising efficacy (Section 1.2.2.2). Based on this, benefit-risk balance of this combination therapy is still considered favorable, supporting the expansion (Arm I, J, K, and L).

Specific areas of potential overlapping toxicities (Section 5.1.4), based on the potential or identified risks of each agent, include neutropenia, infections, infusion-related reactions (IRRs), and tumor lysis syndrome (TLS).

To minimize potential exacerbation of overlapping toxicities, the sequencing of mosunetuzumab and polatuzumab vedotin administration will be tested in up to three different schedules during the dose-finding phase (see Section 3.1.2). The safety monitoring and mitigation plan, which has been adapted specifically for overlapping toxicities for combination treatment, is described in detail in Section 5.1. In addition to the monitoring and mitigation plan for known overlapping risks of mosunetuzumab and polatuzumab vedotin, patients will also be closely followed for toxicities associated with the combination not previously identified with single-agent treatment and/or for more severe or more frequent toxicities than that observed with the individual agents.

1.4.4 COVID-19 Related Risk Assessment

In the setting of the *coronavirus disease 2019* (COVID-19) pandemic, patients with comorbidities including those with cancer, may be a more vulnerable patient population, with the potential for more severe clinical outcomes from COVID-19. Infection with severe acute respiratory syndrome coronavirus 2 (SARS-CoV-2) has been associated with higher morbidity and mortality in patients with cancer in some retrospective analyses. It is unclear whether or how cancer therapies such as chemotherapy, targeted therapy, or immunotherapy impact the incidence or severity of COVID-19. It is not known if mosunetuzumab will increase the risk of infection with SARS-CoV-2.

Severe COVID-19 is associated with dysregulated immune response involving the inflammatory cytokines interleukin (IL)-6, IL-10, IL-2, and IFN-γ (Merad and Martin 2020). Mosunetuzumab is a CD20-targeted therapy and has the identified risk of CRS although it is not known if there may be potential for an increased risk of an enhanced inflammatory response if a participant develops a SARS-CoV-2 infection while receiving mosunetuzumab. Additionally, there may be overlapping clinical features of CRS and severe COVID-19 driven by hyperinflammatory response while receiving mosunetuzumab. Investigators should use their clinical judgment when evaluating and

managing patients with suspected signs and symptoms. Patients with known active infection will be excluded from the study (Section 4.1.2), and guidance on the risk of infection is provided in Section 5.1.2.2 and Section 5.1.3.1. Guidance on the risk of CRS is provided in Section 5.1.2.1.

With reference to *COVID-19* vaccines, an assessment was conducted to determine whether there is an impact of the *COVID-19* vaccines on the benefit-risk assessment of this study protocol. There is limited data concerning the possible interactions between cancer immunotherapy treatment and *COVID-19* vaccination. Given that the mechanism of action of CD20–targeted therapies leads to B cell depletion, it is expected that the efficacy of *COVID-19* vaccines may be diminished. Refer to Section 4.4.1.5 for additional guidance on *COVID-19* vaccine administration and timing.

2. OBJECTIVES AND ENDPOINTS

The study will evaluate the safety, tolerability, pharmacokinetics, and efficacy of IV or SC mosunetuzumab plus polatuzumab vedotin in patients with R/R B-cell NHL. The randomized Phase II portion of the study will evaluate the safety and efficacy of mosunetuzumab SC plus polatuzumab vedotin compared to rituximab plus polatuzumab vedotin in patients with R/R DLBCL. Specific objectives and corresponding endpoints for the study are outlined in Table 7.

Table 7 Objectives and Corresponding Endpoints

Phase Ib-Specific Objectives:	
**Safety Objective**	**Corresponding Endpoints**
• To evaluate the safety and tolerability of mosunetuzumab plus polatuzumab vedotin in patients with R/R DLBCL or FL, including estimation of the MTD, determination of the RP2D, and characterization of DLTs	• Occurrence and severity of adverse events, including DLTs, with severity determined according to NCI CTCAE v5.0; for CRS, severity determined according to the ASTCT CRS Consensus Grading criteria (Appendix 8)<br>• Change from baseline in targeted vital signs<br>• Change from baseline in targeted clinical laboratory test results
**Efficacy Objective**	**Corresponding Endpoints**
• To make a preliminary assessment of the anti-tumor activity of mosunetuzumab plus polatuzumab vedotin	• CR rate at the time of PRA (Section 6.4.1) based on PET-CT, as determined by the investigator using Lugano 2014 criteria (Appendix 7)<br>• Best ORR (CR or PR at any time) in the study based on PET and/or CT scan, as determined by the investigator using Lugano 2014 criteria (Appendix 7)<br>• DOR, defined as the time from the first occurrence of a documented objective response to disease progression or relapse, as determined by the investigator using Lugano 2014 criteria (Appendix 7), or death from any cause, whichever occurs first
**Phase II-Specific Objectives:**	
**Primary Efficacy Objectives**	**Corresponding Endpoint**
• To evaluate the efficacy of mosunetuzumab IV plus polatuzumab vedotin in patients with R/R FL (Arm I)<br>• To evaluate the efficacy of mosunetuzumab IV plus polatuzumab vedotin (Arm J) in patients with R/R DLBCL	• Best ORR (CR or PR at any time) in the study based on PET-CT and/or CT scan, as determined by the IRC using Lugano 2014 criteria (Appendix 7)

Table 7 Objectives and Corresponding Endpoints (cont.)

Phase II-Specific Objectives (cont.):	
**Primary Efficacy Objectives**	**Corresponding Endpoint**
• To evaluate the efficacy of mosunetuzumab SC plus polatuzumab vedotin (Arm K) in patients with R/R MCL<br>• To evaluate the efficacy of mosunetuzumab SC plus polatuzumab vedotin (Arm L) compared to rituximab plus polatuzumab vedotin (Arm M) in patients with R/R DLBCL	
**Secondary Efficacy Objectives**	**Corresponding Endpoints**
• To evaluate the efficacy of mosunetuzumab IV plus polatuzumab vedotin (Arm I) in patients with R/R FL<br>• To evaluate the efficacy of mosunetuzumab IV plus polatuzumab vedotin (Arm J) in patients with R/R DLBCL<br>• To evaluate the efficacy of mosunetuzumab SC plus polatuzumab vedotin (Arm K) in patients with R/R MCL<br>• To evaluate the efficacy of mosunetuzumab SC plus polatuzumab vedotin (Arm L) compared to rituximab plus polatuzumab vedotin (Arm M) in patients with R/R DLBCL	• Best ORR (CR or PR at any time) on study based on PET-CT and/or CT scan, as determined by the investigator using Lugano 2014 criteria (Appendix 7)<br>• Best CR rate on study based on PET-CT and/or CT scan, as determined by the investigator and IRC using Lugano 2014 criteria (Appendix 7))<br>• CR rate at the time of PRA (Section 6.4.1) based on PET-CT, as determined by the investigator and IRC using Lugano 2014 criteria (Appendix 7)<br>• ORR, defined as CR or PR, at PRA based on PET-CT, as determined by the investigator and IRC using Lugano 2014 criteria (Appendix 7)<br>• DOR, defined as the time from the first occurrence of a documented objective response to disease progression or relapse as determined by the investigator and IRC using Lugano 2014 criteria (Appendix 7), or death from any cause, whichever occurs first<br>• PFS, defined as the time from first study treatment to the first occurrence of disease progression or relapse, as determined by the investigator and IRC using Lugano 2014 criteria (Appendix 7), or death from any cause, whichever occurs first<br>• EFS, defined as the time from first study treatment to the first occurrence of disease progression or relapse, as determined by the investigator and IRC using Lugano 2014 criteria (Appendix 7), initiation of NALT, or death from any cause, whichever occurs first.

Table 7 Objectives and Corresponding Endpoints (cont.)

Phase II-Specific Objectives (cont.):	
**Secondary Efficacy Objectives**	**Corresponding Endpoints**
	• OS, defined as the time from first study treatment to death from any cause
**Exploratory Efficacy Objectives**	**Corresponding Endpoints**
• To evaluate the efficacy of mosunetuzumab IV plus polatuzumab vedotin (Arm I) in patients with R/R FL<br>• To evaluate the efficacy of mosunetuzumab IV plus polatuzumab vedotin (Arm J) in patients with R/R DLBCL<br>• To evaluate the efficacy of mosunetuzumab SC plus polatuzumab vedotin (Arm K) in patients with R/R MCL<br>• To evaluate the efficacy of mosunetuzumab SC plus polatuzumab vedotin (Arm L) compared to rituximab polatuzumab vedotin (Arm M) in patients with R/R DLBCL	• Proportion of patients who undergo SCT after achieving a response while in the study<br>• Proportion of patients who undergo allogeneic SCT after achieving a response while in the study<br>• *CIRS-G prognostic impact on PFS and OS for patients ≥65 years old (Arm J, Arm L, and Arm M only)*
**Exploratory Health Status Utility Objective**	**Corresponding Endpoint**
• To assess health status of patients (Arm K only)	• Health status (EQ-5D-5L)
**Safety Objectives**	**Corresponding Endpoints**
• To evaluate the safety of mosunetuzumab IV plus polatuzumab vedotin (Arm I) in patients with R/R FL<br>• To evaluate the safety of mosunetuzumab IV plus polatuzumab vedotin (Arm J) in patients with R/R DLBCL<br>• To evaluate the safety of mosunetuzumab SC plus polatuzumab vedotin (Arm K) in patients with R/R MCL<br>• To evaluate the safety of mosunetuzumab SC plus polatuzumab vedotin (Arm L) compared to rituximab polatuzumab vedotin (Arm M) in patients with R/R DLBCL	• Occurrence and severity of adverse events, with severity determined according to NCI CTCAE v5.0. For CRS, severity determined according to the ASTCT CRS Consensus Grading criteria (Appendix 8).<br>• Change from baseline in targeted vital signs<br>• Change from baseline in targeted clinical laboratory test results
**Objectives for Both Phase Ib and Phase II:**	
**Pharmacokinetic Objective**	**Corresponding Endpoints**
• To characterize the pharmacokinetics of mosunetuzumab (SC and IV) when administered in combination with polatuzumab vedotin (Groups A, B, and C; Arms I, J, K, and L)	• For mosunetuzumab pharmacokinetics in combination with polatuzumab vedotin:<br>• C_{max}<br>• C_{min}

Table 7 Objectives and Corresponding Endpoints (cont.)

Objectives for Both Phase Ib and Phase II (cont.):	
**Pharmacokinetic Objective**	**Corresponding Endpoints**
	• Total exposure (AUC), CL, and volume of distribution, as estimated by population PK modeling, as appropriate, and supported by data
**Exploratory Pharmacokinetic Objectives**	**Corresponding Endpoints**
• To characterize the pharmacokinetics of polatuzumab vedotin when administered in combination with mosunetuzumab (Groups A, B, and C; Arms I, J, K, and L) or rituximab (Arm M)	• For polatuzumab vedotin pharmacokinetics in combination with mosunetuzumab or rituximab:<br>• C_{EOI}<br>• C_{trough}<br>• Total exposure (AUC), CL, and volume of distribution, as estimated by population PK modeling, as appropriate, and supported by data
• To characterize the relationship between pharmacokinetics and safety, biomarkers, or efficacy	• Relationship between pharmacokinetics and safety, biomarkers, or efficacy endpoints, as appropriate
• To assess potential PK interactions between mosunetuzumab and polatuzumab vedotin (Groups A, B, and C; Arms I, J, K, and L)	• Concentrations of mosunetuzumab when administered in combination with polatuzumab vedotin compared with mosunetuzumab given as a single agent based on historical data<br>• Concentrations of polatuzumab vedotin analytes when administered in combination with mosunetuzumab compared with polatuzumab vedotin as a single agent based on historical data
**Immunogenicity Objectives**	**Corresponding Endpoint**
• To assess the incidence of ADAs to mosunetuzumab (Groups A, B, and C; Arms I, J, K, and L)<br>• To assess the incidence of ADAs to polatuzumab vedotin (Groups A, B, and C; Arms I, J, K, M, and L)	• Relationship between ADA status and efficacy, safety, pharmacokinetics, and biomarkers

Table 7 Objectives and Corresponding Endpoints (cont.)

Exploratory Biomarker Objectives	Corresponding Endpoints
• To identify biomarkers that are predictive of response to mosunetuzumab plus polatuzumab vedotin (i.e., predictive biomarkers), are associated with progression to a more severe disease state (i.e., prognostic biomarkers), are associated with acquired resistance to mosunetuzumab plus polatuzumab vedotin, are associated with susceptibility to developing adverse events, can provide evidence of mosunetuzumab plus polatuzumab vedotin activity, or can increase the knowledge and understanding of disease biology<br>• To make a preliminary assessment of response to mosunetuzumab plus polatuzumab vedotin in different clinical and biologic prognostic subgroups of NHL<br>• To make a preliminary assessment of MRD status following mosunetuzumab treatment in combination with polatuzumab vedotin (Groups A, B, and C; Arms I, J, K, *L and M*)	• Association between prognostic subtypes, exploratory biomarkers, and PET-CT CR rate, ORR, DOR, PFS, and EFS endpoints<br>• Relationship over time between ctDNA and tumor burden as measured by imaging

ADA = anti-drug antibody; ASTCT = American Society for Transplantation and Cellular Therapy; AUC = area under the concentration–time curve; C_{EOI} *= concentration at end of infusion; CIRS-G = Cumulative Illness Rating Scale-Geriatric;* CL = clearance; Cmax = maximum serum concentration; Cmin = minimum serum concentration; CR = complete response; CRS = cytokine release syndrome; CT = computed tomography (scan); C_{trough} *= trough concentration;* ctDNA = circulating tumor DNA; DLBCL = diffuse large B-cell lymphoma; DLT = dose-limiting toxicity; DOR = duration of response; EFS = event-free survival; EQ-5D-5L = EuroQol 5-Dimension, 5-Level (questionnaire); FL = follicular lymphoma; IRC = Independent Review Committee; MCL = mantle cell lymphoma; MRD = minimal residual disease; MTD = maximum tolerated dose; NALT = new anti-lymphoma treatment; NCI CTCAE v5.0 = National Cancer Institute Common Terminology Criteria for Adverse Events, Version 5.0; NHL = non-Hodgkin lymphoma; ORR = objective response rate; OS = overall survival; PET = positron emission tomography (scan); PET-CR = complete response based on positron emission tomography scan; PET-CT = positron emission tomography-computed tomography (scan); PFS = progression-free survival; PK = pharmacokinetic; PR = partial response; PRA = primary response assessment; RP2D = recommended Phase II dose; R/R = relapsed or refractory; SCT = stem-cell transplantation.

3. STUDY DESIGN

3.1 DESCRIPTION OF THE STUDY

3.1.1 Overview of Study

This Phase Ib/II open-label, multicenter study will evaluate the safety, tolerability, pharmacokinetics, and efficacy of *IV* or *SC* mosunetuzumab in combination with polatuzumab vedotin in patients with DLBCL*,* FL*, and MCL*. The study will include an

initial dose-finding phase (see Section 3.1.2) followed by a single-arm expansion phase for second line or later (2L+) patients with R/R DLBCL and 2L+R/R FL (see Section 3.1.3). In addition, mosunetuzumab SC dosing in combination with polatuzumab vedotin will be evaluated in patients with at least 2 prior lines of systemic therapy (3L+) for the treatment of R/R MCL (Arm K), and in patients with (2L+) R/R DLBCL in the randomized Phase II portion of the study (Arm L and M). Figure 1 presents an overview of the study design.

Up to [redacted] patients are expected to be enrolled in this study at approximately 40 investigative sites globally.

All patients will be closely monitored for adverse events throughout the study and for at least 90 days after the last dose of study treatment or until the initiation of *next anti-lymphoma treatment*, whichever is earlier (see Section 5.3.1). Adverse events will be graded according to the National Cancer Institute Common Terminology Criteria for Adverse Events, Version 5.0 (NCI CTCAE v5.0), with the exception of CRS events, which will be graded according to the ASTCT CRS Consensus Grading criteria (Appendix 8). An IMC will be established to monitor patient safety throughout the study (see Section 5.1.8).

Blood samples will be taken at various timepoints before and during study treatment administration (see Appendix 3) for biomarker analyses and to characterize the PK properties of mosunetuzumab and polatuzumab vedotin, as well as the immunogenicity of mosunetuzumab and polatuzumab vedotin when given in combination.

Response in the Phase II expansion portion of the study (Arms I, J, K, L, and M) will be determined by an IRC (Section 3.1.7) and investigators using the Lugano Response Criteria for Malignant Lymphoma (Cheson et al. 2014; see Appendix 7), hereinafter referred to as the "Lugano 2014 criteria," at the following timepoints:

- Interim response assessment will occur between D15 of C4 and D21 of C4, prior to C5
- Primary response assessment (PRA)
  - For all cohorts (Groups A and C; Arms I, J, K, L and M, if following Group A or C dosing schedule): at the end of Cycle 8 (C8D21 ± 1 week) (or end of C9 [C9D21 ± 1 week] for Group B; Arms I, J, K, L and M, if following the Group B dosing schedule)

Patients will continue to be evaluated every 3 months (±2 weeks) by computed tomography (CT) scan and/or positron emission tomography (PET)-CT for the first year after C1D1, and then every 6 months (±2 weeks) until disease progression, death, withdrawal of consent, or initiation of *next anti-lymphoma treatment* (see Figure 1B and Section 4.5.6). Tumor assessments should also be performed to confirm clinical suspicion of relapse or disease progression.

The schedules of activities are provided in Appendix 1–Appendix 4.

Figure 1 Study Schema

A. Overview of Study Design

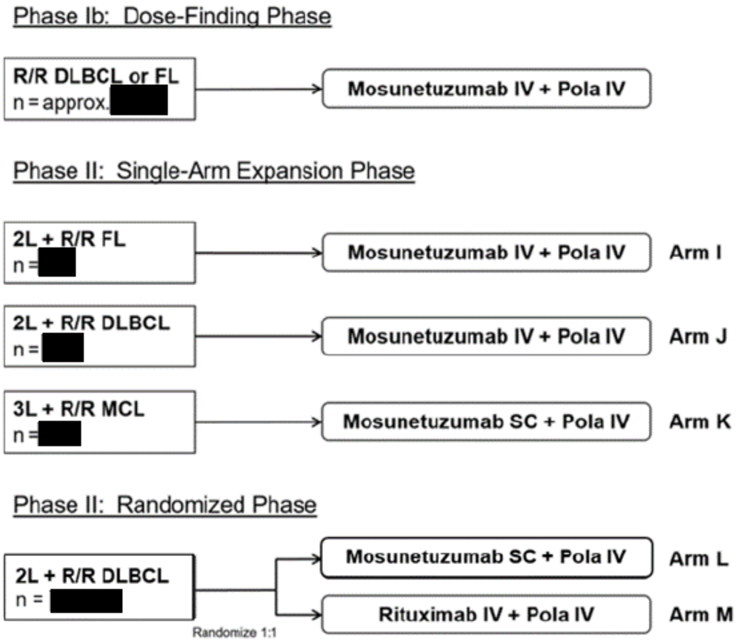

2L + = second line or later; 3L + = third line or later; approx. = approximately; DLBCL = diffuse large B-cell lymphoma; FL = follicular lymphoma; IMC = Internal Monitoring Committee; MCL = mantle cell lymphoma; Pola = polatuzumab vedotin; R/R = relapsed or refractory.

[a] Safety data of the first 6 safety-evaluable patients enrolled in Arm K will be reviewed by the IMC prior to enrolling the full expansion cohort (Section 3.1.3.2).

Figure 1 Study Schema (cont.)

B. Overview of Response Assessments

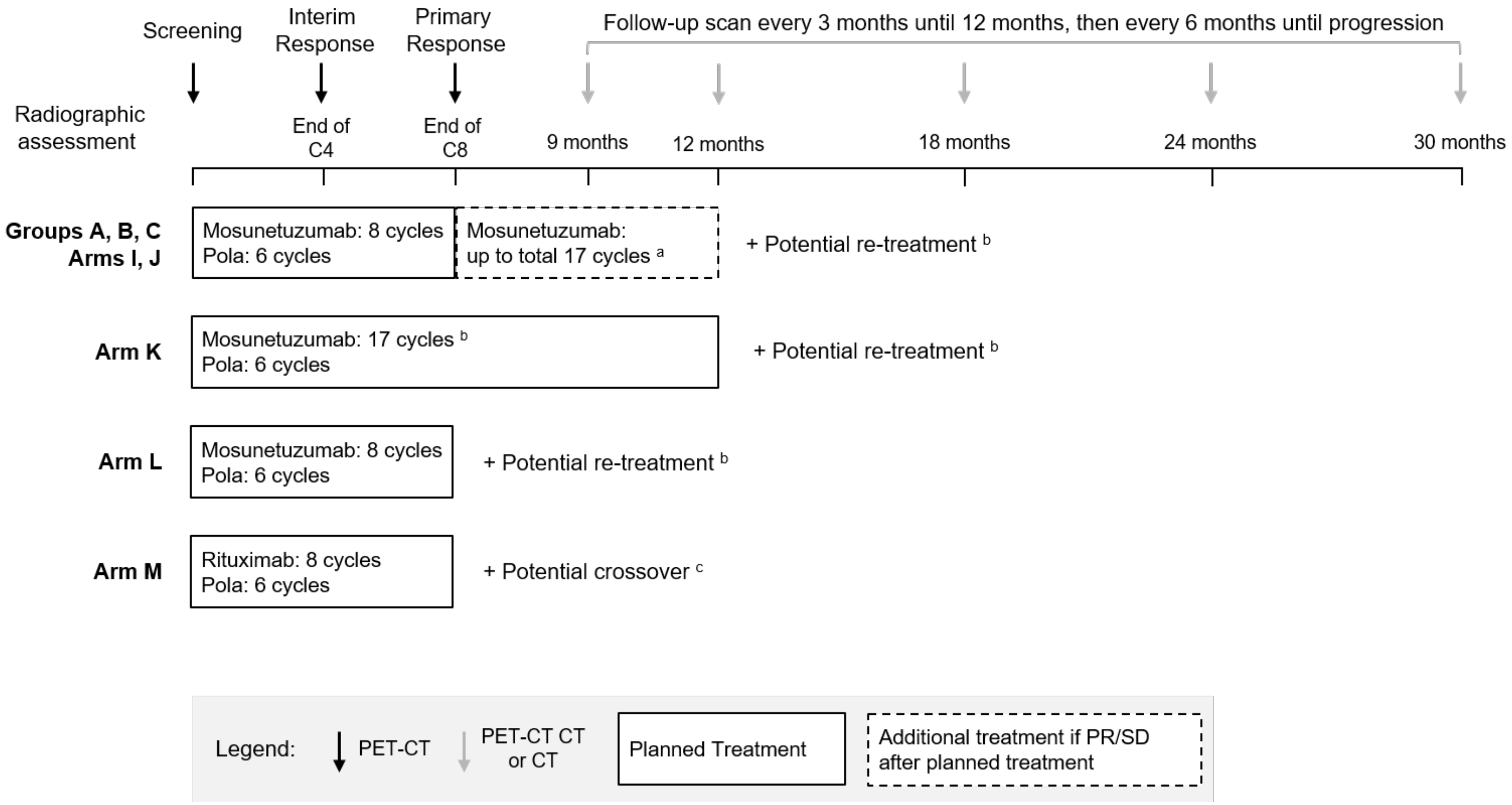

CT = computed tomography (scan); PET-CT = positron emission tomography–computed tomography (scan); Pola = polatuzumab vedotin.

[a] See Section 3.1.5 for mosunetuzumab treatment beyond 8 cycles and re-treatment

[b] See Section 3.1.5 for mosunetuzumab re-treatment

[c] See Section 3.1.5 for Arm M-crossover

Study treatments will be administered every 21 days.

- Mosunetuzumab will be administered for 8 or 17 cycles for Groups A, B, and C, and Arms I and J (see Section 3.1.5 for mosunetuzumab treatment duration).
- Mosunetuzumab will be administered for 17 cycles for Arm K only.
- Mosunetuzumab will be administered for 8 cycles for Arm L and Arm M-crossover (See Section 3.1.5.2).
- Rituximab (375 mg/m^2) will be administered for 8 cycles for Arm M.
- Polatuzumab vedotin (1.8 mg/kg) will be administered for 6 cycles for Groups A, B, and C, and Arms I, J, K, L and M.
- Polatuzumab vedotin (1.8 mg/kg) will be administered for 6 cycles or less for Arm M-crossover, depending on the number of cycles of polatuzumab vedotin administered in Arm M (See Section 3.1.5.2)

3.1.2 Dose-Finding Phase

The purpose of the dose-finding phase is to determine the RP2D and schedule for mosunetuzumab when given in combination with fixed doses of polatuzumab vedotin (1.8 mg/kg) in patients with R/R DLBCL or FL. Approximately 9–42 patients with either R/R DLBCL or FL may be enrolled in up to three dose-escalation treatment groups (Figure 2):

- **Group A:** C1 step-up mosunetuzumab escalation with concurrent administration of polatuzumab vedotin starting in C1, both administered by IV infusion
- **Group B:** C1 polatuzumab vedotin with delayed start C1 step-up mosunetuzumab escalation, both administered by IV infusion
- **Group C:** C1 step-up mosunetuzumab escalation with concurrent administration of polatuzumab vedotin starting in C2, both administered by IV infusion

Dose escalation Groups A, B, and C may be run sequentially or in parallel, at the discretion of the Sponsor. Dose escalation will be performed based on a modified 3+3 design. Dose-escalation cohorts (within each group) will consist of at least 3 patients, unless DLTs are observed in the first 2 patients prior to enrollment of a third patient. Approximately 6–12 patients will be treated at the RP2D and schedule of mosunetuzumab in combination with polatuzumab vedotin prior to the expansion phase.

For each dose-escalation cohort in Groups A, B, and C, treatment will be staggered such that the second patient enrolled in the cohort will receive the first dose of study treatment at least 72 hours after the first enrolled patient receives the first dose of study treatment, to assess for any severe and unexpected acute drug or infusion-related toxicities. Dosing in subsequent patients in each cohort will be staggered by at least 24 hours from the end of the prior patient's administration. In each scenario, the Sponsor must receive documentation of the status of the prior patient before the next patient receives the first dose of study treatment.

Patients will be closely monitored for adverse events during a DLT assessment window. Adverse events meeting the criteria for DLT, as defined below (see Section 3.1.2.1), will be reported to the Sponsor within 24 hours (see Section 5.4.2). Staggered patient enrollment will not be required for enrollment of additional patients to acquire additional safety and PD data at a dose level that has been shown to not exceed the MTD.

Patients exhibiting acceptable safety and evidence of clinical benefit (as defined in Section 3.1.2.6) may continue to receive study treatment every 21 days for 8 cycles (or 17 cycles if PR or stable disease [SD] after 8 cycles) for mosunetuzumab, and for 6 cycles for polatuzumab vedotin, until confirmed objective disease progression or unacceptable toxicity, whichever occurs first. Re-treatment with mosunetuzumab combined with polatuzumab vedotin based on clinical responses to initial treatment are detailed in Section 3.1.5.

Dose-finding Groups A, B, or C may be prioritized or suspended by the Sponsor based on the overall safety profile, in consultation with the IMC (Section 5.1.8). As described in Section 5.1.8, the IMC will review cumulative safety data and make recommendations regarding dose escalation and overall study conduct on the basis of trial safety data to ensure patient safety while receiving study treatment. These include recommendations to open or suspend patient enrollment in a given dose-escalation cohort based on the overall benefit–risk profile of mosunetuzumab in combination with polatuzumab vedotin during dose finding.

Mosunetuzumab dose levels will be independent of patient weight (flat-dosing).
The starting dose level of step-up mosunetuzumab is 1 mg (DL_1, fixed for all schedules), 2 mg (DL_2, fixed for all schedules, given 7 days after DL_1), and 9 mg (DL_3, initial mosunetuzumab test dose, given 7 days after DL_2), for each initial cohort in Groups A, B, and C based on preliminary data from Study GO29781; for additional details, see the rationale for mosunetuzumab starting dose and schedule (Section 3.3.1).

During dose finding in Groups A, B, and C, only the DL_3 test dose may be escalated or de-escalated according to the rules below.

Figure 2 shows an overview of the dose-finding phase.

Figure 2 Study Schema: Dose-Finding Phase (Groups A, B, and C)

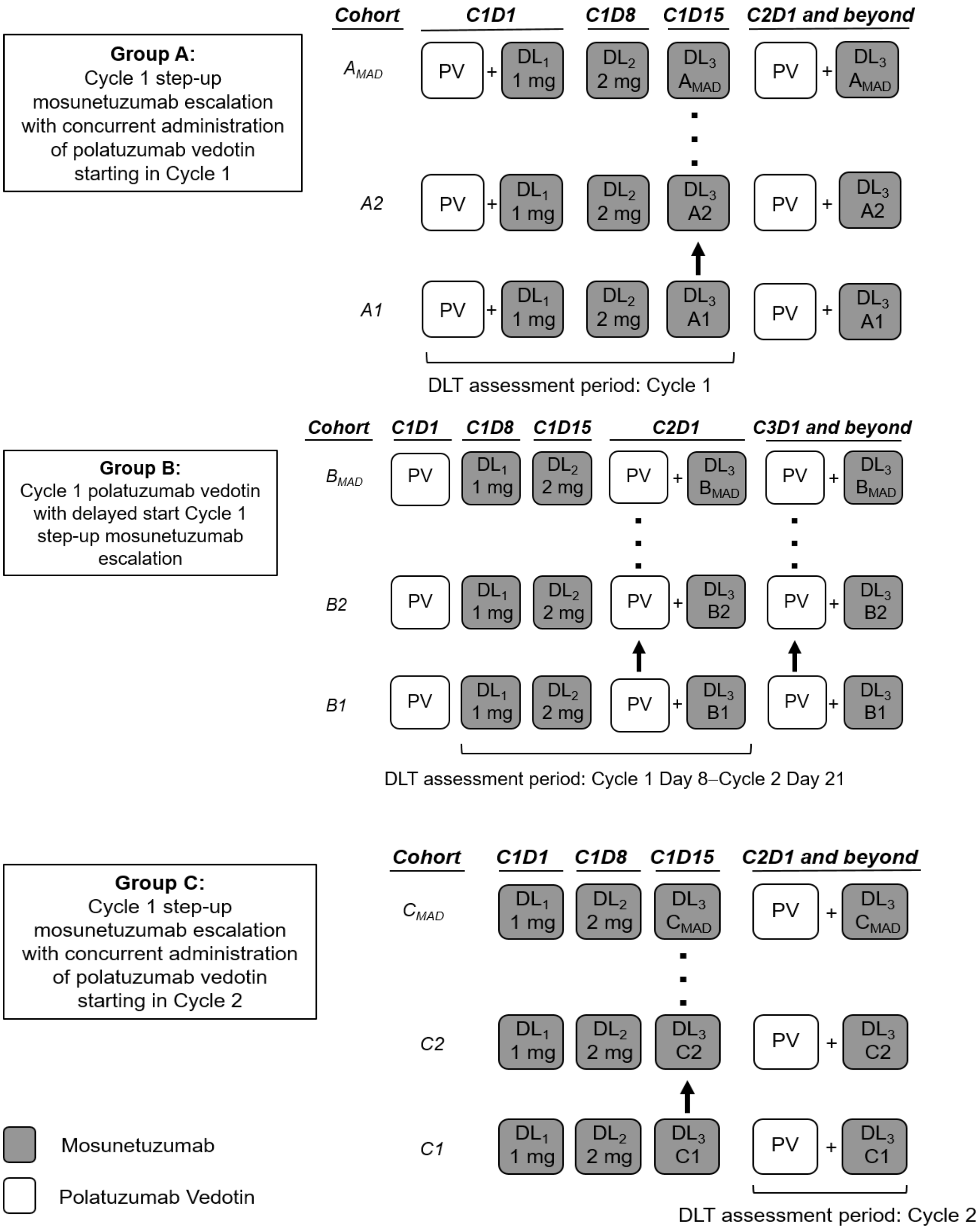

C = cycle (except in reference to group/cohort “C”); D = day; DL = dose level; DLT = dose-limiting toxicity; PV = polatuzumab vedotin; MAD = maximal assessed dose.

Note: $DL_{1–3}$ indicates mosunetuzumab step-up dose levels 1–3.

3.1.2.1 Definition of DLT Assessment Period and DLTs

All adverse events, including DLTs, will be reported according to instructions in Section 5.2 and graded according to NCI CTCAE v5.0 unless otherwise indicated. Although CRS will be graded according to the ASTCT CRS consensus grading criteria (Appendix 8), for dose-escalation decisions, DLTs related to CRS will be defined based on individual signs and symptoms and laboratory data (Section 5.3.5.1) according to NCI CTCAE v5.0. DLTs will be treated according to clinical practice and will be monitored through their resolution. All adverse events should be considered related to study treatment unless such events are clearly attributed by the investigator to another clearly identifiable cause (e.g., documented disease progression, concomitant medication, or pre-existing medical condition). Decreases in B cells, lymphopenia, and/or leukopenia due to decreases in B cells will not be considered DLTs as they are expected PD outcomes of mosunetuzumab and polatuzumab vedotin treatment.

3.1.2.1.1 Definition of DLT Assessment Period

For dose-escalation purposes, the DLT assessment period is defined by the following time periods depending on the assigned dose-escalation group, based on the timing of first administration of mosunetuzumab in the presence of polatuzumab vedotin (Figure 3):

- **Group A:** C1D1 through C1D21
- **Group B:** C1D8 through C2D21
- **Group C:** C2D1 through C2D21

For individual patients who have dose delays for a non-DLT adverse event of 7 days or fewer following the first DL_1, DL_2, or DL_3 dose, the DLT assessment period will be extended as follows:

- **Group A**: The DLT window will extend until the start of the actual C2D1 infusion.
- **Group B**: The DLT window will extend until the start of the actual C3D1 infusion.
- **Group C**: The DLT window will extend until the start of the actual C3D1 infusion.

For patients who have dose delays for a non-DLT adverse event exceeding 7 days following the first DL_1, DL_2, or DL_3 dose, the patient may be DLT unevaluable for the purposes of clearing the cohort (see Section 3.1.2.2).

3.1.2.1.2 Definition of DLT

In the dose-finding phase of this study (Group A, B, or C), a DLT is defined as any <u>one</u> of the following events occurring during the DLT assessment period:

- Any Grade ≥3 hematologic adverse event in the absence of another clearly identifiable cause, with the following exceptions:
  - Grade 3 or 4 neutropenia that is not accompanied by temperature elevation (as a single oral temperature of ≥ 38.3°C [101°F] or an oral temperature of ≥38.0°C [100.4°F] sustained for ≥1 hour) and improves to Grade ≤2 (or to ≥80% of the baseline value, whichever is lower) without a delay of the next scheduled cycle of study treatment exceeding 7 days
  - Grade 3 or 4 lymphopenia, which is an expected outcome of therapy
  - Grade 3 or 4 leukopenia, which is an expected outcome of therapy
  - Grade 3 or 4 thrombocytopenia that improves to Grade ≤2 (or to ≥80% of the baseline value, whichever is lower) before D1 of the next scheduled cycle of study treatment without platelet transfusion and is not associated with bleeding that is considered clinically significant by the investigator
  - Grade 3 or 4 anemia that does not require an emergent transfusion
- Any Grade ≥3 non-hematologic adverse event not considered by the investigator to be attributable to another clearly identifiable cause, with the following exceptions:
  - Grade 3 diarrhea that responds to standard-of-care therapy within 72 hours
  - Grade 3 nausea or vomiting in the absence of premedication or that can be managed with resulting resolution to Grade ≤2 with oral or IV anti-emetics within 72 hours
  - Grade 3 nausea or vomiting that requires total parenteral nutrition or hospitalization are not excluded and should be considered a DLT.
  - Grade 3 laboratory abnormality that is asymptomatic and deemed by the investigator not to be clinically significant
  - Grade 3 fatigue lasting ≤3 days
  - Grade 3 (NCI CTCAE v5.0) individual signs and symptoms of CRS after mosunetuzumab infusion (Section 5.3.5.1) that occurs in the context of Grade ≤ 2 CRS (as defined by the ASTCT CRS Consensus Grading criteria; (Appendix 8) and lasts <3 days will not be considered a DLT
  - Grade 3 elevation in ALT or AST, provided the following criteria are met:

    Any Grade 3 AST or ALT elevation that lasts <3 days

    ALT or AST level is ≤8 × the upper limit of normal (ULN)

    ALT or AST elevation resolves to Grade <2 (< 5 × ULN) within 7 days

    Total and direct bilirubin and other laboratory parameters of liver synthetic function (e.g., prothrombin time) are normal

    There are no clinical signs or symptoms of hepatic injury
- Any case involving an increase in hepatic transaminase >3× baseline in combination with either an increase in direct bilirubin >2× ULN or clinical jaundice, without any findings of cholestasis or signs of hepatic dysfunction and in the absence of other contributory factors (e.g., worsening of metastatic disease or

concomitant exposure to known hepatotoxic agent or of a documented infectious etiology) is suggestive of potential drug-induced liver injury (according to Hy's Law) and will be considered a DLT unless the following criteria are met:

  - Any AST or ALT $> 3 \times$ the ULN and total bilirubin $> 2 \times$ ULN where no individual laboratory value exceeds Grade 3 and lasts < 3 days will not be considered a DLT.

3.1.2.2 Dose-Finding Rules and Determination of the Maximum Tolerated Dose

Specific rules for Groups A, B, and C dose finding are detailed in Sections 3.1.2.3, 3.1.2.4, and 3.1.2.5. Initiation of individual group dose escalations will be at the Sponsor's discretion. All dose-escalation decisions will be made based on recommendations of the IMC. Relevant demographic, adverse event, laboratory, dose administration, and available PK and PD data (e.g., plasma cytokines and markers of T-cell activation) will be reviewed prior to each dose-escalation decision.

- Determination of whether a patient is evaluable for DLT assessment will be made in accordance with the following rules:
  - Patients who receive study treatment and remain on study through the DLT assessment window will be considered DLT-evaluable.
  - Patients who discontinue from treatment with mosunetuzumab or polatuzumab vedotin prior to completing the DLT assessment window for reasons other than a DLT will be considered non-evaluable for dose-escalation decisions and MTD determination and will be replaced by an additional patient at that same dose level.
- For patients enrolled into Group A only: Patients who have dose delays of mosunetuzumab exceeding 7 days (Section 3.1.2.3) following the scheduled C1D1, C1D8, or C1D15 dose of mosunetuzumab for a non-DLT adverse event may be DLT-unevaluable and may be replaced at the discretion of the Medical Monitor.
- For patients enrolled into Group B only: Patients who have dose delays exceeding 7 days (Section 3.1.2.4) following the scheduled C1D8, C1D15, or C2D1 dose of mosunetuzumab for a non-DLT adverse event may be DLT-unevaluable and may be replaced at the discretion of the Medical Monitor.
- For patients in Group C only: If an enrolled patient experiences any treatment-emergent toxicity that does not completely resolve to baseline level prior to initiation of combination treatment in C2, that patient would be considered unevaluable for dose-escalation decisions and MTD determination and will be replaced by an additional patient at that same dose level and schedule.
- Patients who receive supportive care during the DLT assessment window that confounds the evaluation of DLTs (not including supportive care described in Section 3.1.2.1 as part of the DLT definition) may be replaced at the discretion of the Medical Monitor.
- If a patient experiences a DLT as described above, the patient will be observed for resolution of the toxicity. If the DLT resolves to Grade ≤ 2 (or $\geq 80\%$ of the

baseline value) and it is determined to be in the patient's best interest to continue study treatment, the patient may continue to receive study treatment *at the investigator's discretion following consultation with the Medical Monitor*.

On the basis of a review of real-time safety data and available preliminary PK data, dose escalation may be halted or modified by the Sponsor as deemed appropriate. During the dose-finding phase, DL_3 dose escalation will be based on IMC review of the totality of safety data in dose escalation and expansion. Based on IMC recommendations, the DL_3 dose may be escalated up to 100%, or up to, but not exceeding, a dose corresponding to one dose level below the maximum assessed dose for mosunetuzumab using the step-up schedule as a single agent from Study GO29781.

To acquire additional safety and PD data to better inform the RP2D, additional patients may be enrolled at a dose level that has been shown to not exceed the MTD based on the dose-escalation criteria described above, and at which there is evidence of anti-tumor activity and/or PD biomarker modulation. Up to approximately 3 additional patients per dose level may be enrolled. For the purposes of dose-escalation decisions, these patients will not be included as part of the DLT-evaluable population.

Mosunetuzumab Starting Dose for Groups A, B, and C

The starting dose level of step-up mosunetuzumab is 1 mg (DL_1, fixed for all schedules), 2 mg (DL_2, fixed for all schedules), and 9 mg (DL_3, initial mosunetuzumab test dose) for each initial cohort in Groups A, B, and C. This starting dose level is one dose level below the maximum assessed dose for mosunetuzumab with the step-up schedule as a single agent from Study GO29781 (for additional details, see Sections Section 1.2.2.1 and 3.3.1, and the Mosunetuzumab Investigator's Brochure). During dose finding in Groups A, B, and C, only the DL_3 test dose may be escalated or de-escalated according to the rules below.

3.1.2.3 Group A: Cycle 1 Step-Up Mosunetuzumab Escalation with Concurrent Administration of Polatuzumab Vedotin Starting in Cycle 1

Group A will evaluate mosunetuzumab and polatuzumab vedotin given concurrently starting on C1D1.

Dosing Schedule

Patients enrolled in dose-finding Group A will receive mosunetuzumab- 1 mg (DL_1) on C1D1, 2 mg (DL_2) on C1D8, and the first DL_3 test dose on C1D15 by IV infusion. In C2 and beyond (up to 8–17 cycles, see Section 3.1.5), the mosunetuzumab DL_3 dose will be given on D1 of each 21-day cycle, with D1 of C2 being 7 days after the C1D15 dose.

Patients will receive polatuzumab vedotin 1.8 mg/kg by IV infusion on D1 of each 21-day cycle for up to a maximum of 6 cycles, starting on C1D1.

Mosunetuzumab and polatuzumab vedotin may be given up to ±1 day from the scheduled date for C2 (i.e., with a minimum of 6 days after C1D15 dosing), and ±2 days from the scheduled date for C3 and beyond (i.e., with a minimum of 19 days between doses) for logistic/scheduling reasons. See Sections 4.3.2 and 4.3.3 for additional details on study treatment administration.

Dose-Finding Rules

Dose escalation in Group A will use a modified 3+3 design. The DLT assessment period for Group A is C1D1 through C1D21 (see Figure 3). Dose escalation of mosunetuzumab DL_3 alone will be based on recommendations by the IMC for each successive cohort if the escalation rules outlined below are met:

- A minimum of 3 patients will initially be enrolled in each cohort unless the first 2 enrolled patients experience a protocol-defined DLT (see Section 3.1.2.1), in which case enrollment into the cohort will be terminated.
- If none of the first 3 DLT-evaluable patients experiences a DLT, enrollment of the next cohort at the next highest dose level may proceed.
- If 1 of the first 3 DLT-evaluable patients experiences a DLT, the cohort will be expanded to 6 patients. All patients will be evaluated for DLTs before any dose-escalation decision.
  - If no additional patient experiences a DLT in the first 6 DLT-evaluable patients, enrollment of the next cohort at the next highest dose level may proceed.
- If the cohort has expanded to 6 patients, the cumulative MTD of a cohort may be exceeded under two scenarios (see below), depending on the timing of DLTs relative to the administration of the first DL_3 test dose (on C1D15 in Group A). Because of the step-up dosing schedule of mosunetuzumab during the DLT assessment period, DLTs occurring prior to the administration of the first DL_3 test dose should be ascribed to the administration of DL_1 or DL_2. DLTs occurring after the administration of the first DL_3 test dose should be ascribed to the administration of DL_1, DL_2, or DL_3.
  - Scenario 1:

    Because DL_1 and DL_2 are fixed for each cohort in each group and are evaluated with each cohort during dose escalation, the assessment of MTD associated with these two doses will review all DLTs occurring prior to the administration of the first DL_3 test dose across all cohorts within a group.

    The MTD will have been exceeded if the number of DLTs prior to the administration of the first DL_3 test dose across all applicable cohorts has ≥80% chance that the true DLT rate ≥20%, by the posterior probability approach (Thall and Simon 1994). For example, there is an ≥80% chance that true DLT rate ≥20% if DLTs observed in 2/4, 2/5, 2/6, 3/7, 3/8, 3/9, 3/10, 4/11, 4/12, 4/13, 4/14, or 5/15 patients.

    If the MTD has been exceeded based on DLTs occurring prior to the administration of the first DL3 test dose, there are no dose reductions allowed for DL_1 or DL_2. Instead an alternate schedule in other groups should be tested.

– Scenario 2:

  If 2 or more out of 6 DLT-evaluable patients experience a DLT after the administration of the first DL_3 test dose, the MTD will have been exceeded and dose escalation will stop. An additional 3 patients will be evaluated for DLTs at the preceding dose level, unless 6 patients have already been evaluated at that level. However, if the dose level at which the MTD is exceeded is $>$25% higher than the preceding dose level, 6 patients may be evaluated at an intermediate dose level.

- If the MTD is exceeded at any dose level, the highest dose where fewer than 2 out of 6 DLT-evaluable patients (i.e., $<$33%) experience a DLT after the administration of the first DL_3 test dose will be declared the MTD.
- If the MTD is not exceeded at any dose level, the highest dose administered in this group will be declared the maximum assessed dose.
- Dose de-escalation
  – In the event that the initial mosunetuzumab DL_3 test dose in combination with polatuzumab vedotin is above the MTD (i.e., $\geq$33% out of 6 DLT-evaluable patients experience a DLT after the administration of the first DL_3 test dose), a reduced DL_3 dose level that is at least 25% lower may be evaluated in an additional cohort of 3 to 6 patients. If this dose level is again above the MTD, further DL_3 dose reductions of $>$25% of the preceding DL_3 dose may be assessed in subsequent cohorts of 3–6 patients.

  Mosunetuzumab dose levels may be rounded if the difference before and after the rounding is within 15% (e.g., 13.5 may be rounded to 14 mg, and 27 mg may be rounded to 30 mg).

  An example of dose-escalation and de-escalation is shown in Table 8; doses listed in this table are for illustrative purposes only.

3.1.2.4 Group B: Cycle 1 Polatuzumab Vedotin with Delayed Start Cycle 1 Step-Up Mosunetuzumab Escalation

Group B will evaluate an alternate schedule of polatuzumab vedotin and mosunetuzumab, with polatuzumab starting on C1D1 and mosunetuzumab step-up doses starting on C1D8. Group B dose escalation may proceed in parallel with Groups A and C dose escalation.

Dosing Schedule

Patients enrolled in dose-escalation Group B will receive polatuzumab vedotin 1.8 mg/kg by IV infusion on D1 of each 21-day cycle for up to a maximum of 6 cycles, starting on C1D1.

Patients will receive mosunetuzumab 1 mg (DL_1) on C1D8, 2 mg (DL_2) on C1D15, and the first DL_3 test dose on C2D1 by IV infusion. In C3 and beyond (up to 9–17 cycles, see Section 3.1.5) the mosunetuzumab DL_3 dose will be given on D1 of each cycle.

Mosunetuzumab and polatuzumab vedotin may be given up to ±2 days from the scheduled date for C3 and beyond (i.e., with a minimum of 19 days between doses) for logistic/scheduling reasons.

Dose-Finding Rules

Dose escalation in Group B will use the same modified 3+3 design and dose-escalation and de-escalation rules as Group A (Section 3.1.2.3). The DLT assessment period for Group B is C1D8 through C2D21 (see Figure 3). The main difference between Group A and Group B dose-escalation rules is the timing of the administration of the first DL_3 test dose, which occurs on C2D1 for Group B (see Figure 3). An example of dose-escalation and de-escalation is illustrated in Table 8; doses listed in this table are for illustrative purposes only.

3.1.2.5 Group C: Cycle 1 Step-Up Mosunetuzumab Escalation with Concurrent Administration of Polatuzumab Vedotin Starting in Cycle 2

Group C will evaluate an alternate schedule of polatuzumab vedotin and mosunetuzumab, with mosunetuzumab given on a step-up schedule starting on C1D1, and polatuzumab vedotin given starting on C2D1. Group C dose escalation may proceed in parallel with Groups A and B dose escalation.

Dosing Schedule

Patients enrolled in Group C will receive mosunetuzumab 1 mg (DL_1) on C1D1, 2 mg (DL_2) on C1D8, and DL_3 test dose on C1D15 by IV infusion. In C2 and beyond (up to 8–17 cycles, see Section 3.1.5) the mosunetuzumab DL_3 dose will be given on D1 of each 21-day cycle.

Patients will receive polatuzumab vedotin 1.8 mg/kg by IV infusion on D1 of each cycle up to a maximum of 6 cycles, starting on C2D1.

For logistic/scheduling reasons, C2D1 administration of mosunetuzumab and polatuzumab vedotin may be given up to ±1 day from the scheduled date for C2 (i.e., with a minimum of 6 days after C1D15 dosing, and ±2 days from the scheduled date for C3 and beyond [with a minimum of 19 days between doses]). C2D1 dosing delays are detailed in Section 3.1.2.2.

Dose-Finding Rules

The DLT assessment window in Group C is from C2D1 through C2D21 (see Figure 3), because polatuzumab vedotin is given in combination with mosunetuzumab starting on C2D1. Between C1D1 and C1D21, if a patient experiences a treatment-emergent toxicity that does not completely resolve to baseline level by C2D1, the patient may be considered unevaluable for dose-escalation decisions and MTD determination and be replaced by an additional patient at that same dose level and schedule.

Dose escalation in Group C will use a 3 + 3 design. Dose escalation of mosunetuzumab DL_3 alone will be based on recommendations by the IMC for each successive cohort if the escalation rules outlined below are met:

- A minimum of 3 patients will initially be enrolled in each cohort unless the first 2 enrolled patients experience a protocol-defined DLT (see Section 3.1.2.1) in which case enrollment into the cohort will be terminated.
- If none of the first 3 DLT-evaluable patients experiences a DLT, enrollment of the next cohort at the next highest dose level may proceed.
- If 1 of the first 3 DLT-evaluable patients experiences a DLT, the cohort will be expanded to 6 patients. All patients will be evaluated for DLTs before any dose-escalation decision. If there are no additional patients experiencing a DLT in the first 6 DLT-evaluable patients, enrollment of the next cohort at the next highest dose level may proceed.
  - If 2 or more out of 6 DLT-evaluable patients in a cohort experience a DLT, the MTD will have been exceeded and dose escalation will stop. An additional 3 patients will be evaluated for DLTs at the preceding dose level, unless 6 patients have already been evaluated at that level. However, if the dose level at which the MTD is exceeded is ≥ 25% higher than the preceding tested dose level, 6 patients may be evaluated at an intermediate dose level.
- If the MTD is not exceeded at any dose level, the highest dose at which fewer than 2 out of 6 DLT-evaluable patients (i.e., < 33%) experience a DLT will be declared the MTD.
- If the MTD is not exceeded at any dose level, the highest dose administered in this group will be declared the maximum assessed dose.
- Dose de-escalation
  - In the event that the initial DL_3 test dose in combination with polatuzumab vedotin is above the MTD (i.e., ≥ 33% out of 6 DLT-evaluable patients experience a DLT), a reduced DL_3 dose level that is at least 25% lower may be evaluated in an additional cohort of 3 to 6 patients. If this dose level is again above the MTD, further DL_3 dose reductions of > 25% of the preceding DL_3 dose may be assessed in subsequent cohorts of 3–6 patients.
  - The highest dose level where fewer than 2 out of 6 DLT-evaluable patients (i.e., < 33%) experience DLTs will be declared the MTD.
- Mosunetuzumab dose levels may be rounded if the difference before and after the rounding is within 15% (e.g., 13.5 may be rounded to 14 mg, and 27 mg may be rounded to 30 mg).
- An example of dose-escalation and de-escalation is shown in Table 8; doses listed in this table are for illustrative purposes only.

Figure 3 DLT Assessment Periods for Groups A, B, and C

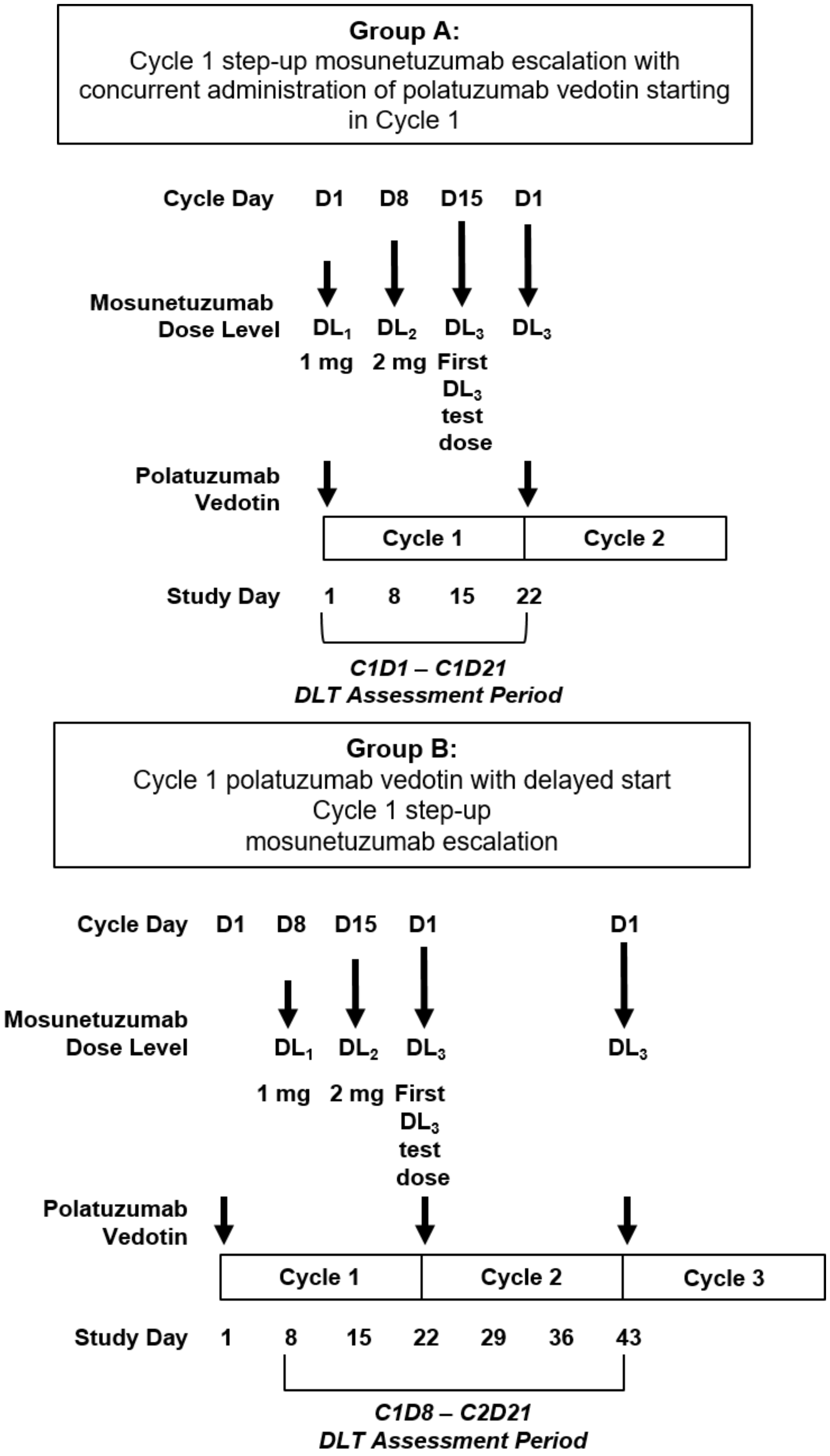

Figure 3 DLT Assessment Periods for Groups A, B, and C (cont.)

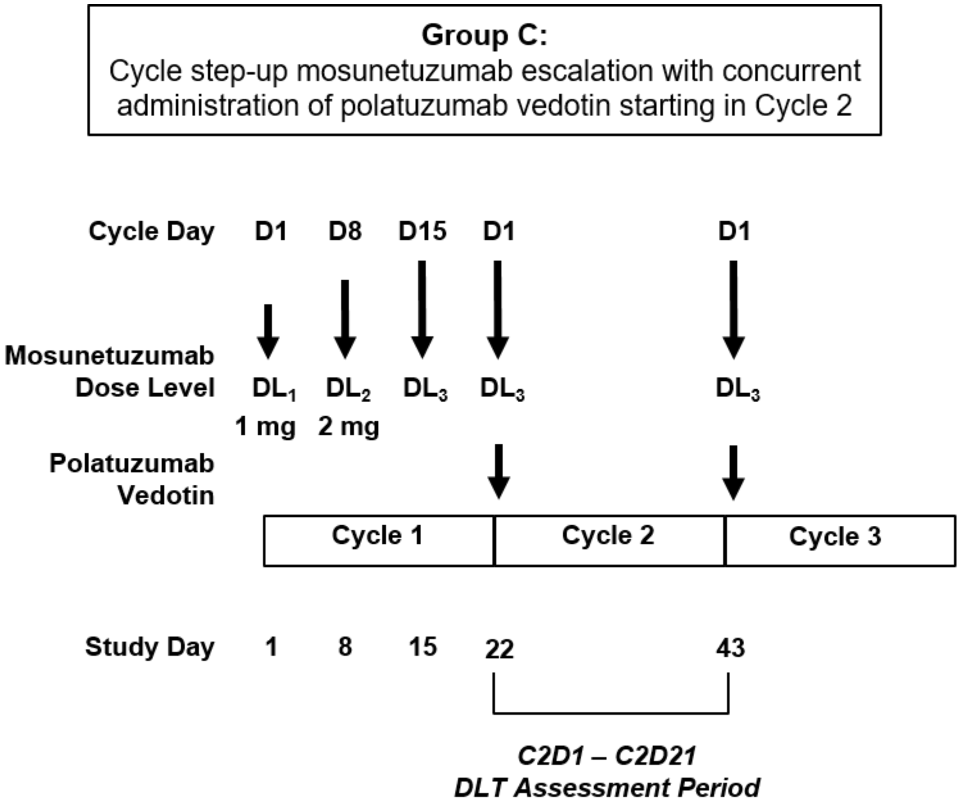

C = cycle; D = day; DL = dose level; DLT = dose-limiting toxicity.

Table 8 Examples of Dose Escalation and De-Escalation for Groups A, B, and C

Group	Cohort	DL_1 (mg)	DL_2 (mg)	DL_3 (mg)
A	Cohort A3 (escalation)	1.0	2.0	40.0
	Cohort A2 (escalation)	1.0	2.0	20.0
	Cohort A1 (initial cohort)	1.0	2.0	9.0
	Cohort A0 (de-escalation)	1.0 No DL_1 de-escalation allowed; assess alternate schedule.	2.0 No DL_2 de-escalation allowed; assess alternate schedule.	6.0
B	Cohort B3 (escalation)	1.0	2.0	40.0
	Cohort B2 (escalation)	1.0	2.0	20.0
	Cohort B1 (initial cohort)	1.0	2.0	9.0
	Cohort B0 (de-escalation)	1.0 No DL_1 de-escalation allowed; assess alternate schedule.	2.0 No DL_2 de-escalation allowed; assess alternate schedule.	6.0
C	Cohort C3 (escalation)	1.0	2.0	40.0
	Cohort C2 (escalation)	1.0	2.0	20.0
	Cohort C1 (initial cohort)	1.0	2.0	9.0
	Cohort C0 (de-escalation)	1.0 No DL_1 de-escalation allowed.	2.0 No DL_2 de-escalation allowed.	6.0

DL = dose level.

3.1.2.6 Rules for Continued Dosing Beyond the DLT Assessment Period

Patients who meet the below criteria will be eligible to receive additional cycles of study treatment with mosunetuzumab given in combination with polatuzumab vedotin every 21 days (the day of infusion being D1 of each cycle) beyond the DLT assessment period.

- Ongoing clinical benefit: Patients must have no clinical signs or symptoms of progressive disease; radiographic tumor assessments at the end of the DLT window are not required in order to minimize unnecessary radiation exposure. Patients will be clinically assessed for disease progression on D1 of each cycle.
  - For patients with NHL, disease progression should be confirmed by radiographic imaging as defined by Lugano 2014 criteria (Appendix 7). Patients with NHL who have radiographic disease progression prior to the completion of the study treatment period will be generally ineligible to receive further mosunetuzumab treatment. However, in limited cases, treatment after apparent radiographic disease progression may be allowed as outlined in Section 3.1.4.
- Acceptable toxicity: Patients who experience Grade 4 non-hematologic adverse events with the possible exception of Grade 4 TLS should discontinue study treatment and may not be re-treated. Patients who experience Grade 4 TLS may be considered for continued study treatment provided they meet the criteria specified in Section 5.1.7. All other study treatment related adverse events from prior study treatment administration must have decreased to Grade ≤ 1 or baseline grade by the next administration. Exceptions on the basis of ongoing overall clinical benefit may be allowed after a careful assessment and discussion of benefit–risk with the patient by the study investigator and the Medical Monitor. Any treatment delay for toxicities not attributed to study treatment may not require study treatment discontinuation but must be discussed with the Medical Monitor.

Patients exhibiting acceptable safety and evidence of clinical benefit as described above may continue to receive study treatment as described in Section 3.1.5. Within each treatment group, a lower dose level on D1 of C3 or subsequent cycles may be administered to assess whether a lower dose than the mosunetuzumab DL_3 administered in the first two cycles is sufficient to maintain clinical efficacy during later cycles.

Once an RP2D is declared, the IMC may permit patients receiving mosunetuzumab at doses below the RP2D to be dose escalated to the RP2D. A patient may be dose escalated to the RP2D provided that no prior DLTs or dose reductions have occurred, and provided that the treating physician views such dose escalation is in the patient's best interest.

Patients who complete study treatment without disease progression will continue to be monitored, including regularly scheduled tumor assessments as outlined in Appendix 2 until discontinuation from the post-treatment follow-up (e.g., due to progression).

Patients may have the option for re-treatment for development of recurrent disease as described in Section 3.1.5.

3.1.3 Expansion Phase

The expansion phase is designed to further assess the safety and efficacy of mosunetuzumab and polatuzumab vedotin at the RP2D and schedule determined in the dose-finding phase. Mosunetuzumab dose and schedule in combination with polatuzumab vedotin to be assessed in the expansion phase will be determined based on IMC recommendation and in consultation with the investigators following a review of cumulative safety data in dose finding. Patients with R/R DLBCL, R/R FL, or R/R MCL will be enrolled during the expansion phase and treated as described below.

3.1.3.1 Single-Arm Expansion

Approximately [redacted] patients will be treated with mosunetuzumab plus polatuzumab vedotin in the single-arm expansion phase. The patient cohorts will be assigned to the following arms:

- **Arm I**: R/R FL (Grade 1–3a); [redacted] patients (mosunetuzumab IV plus polatuzumab vedotin)
- **Arm J**: R/R DLBCL, transformed FL, or Grade 3b FL; 100 patients (mosunetuzumab IV plus polatuzumab vedotin)
- **Arm K**: R/R MCL; [redacted] patients (mosunetuzumab SC plus polatuzumab vedotin)

Dose expansion Arms I, J, and K may be run sequentially or in parallel and may also be prioritized or suspended, at the discretion of the Sponsor.

3.1.3.2 Arm K: Safety Run-In and Expansion

Arm K may begin enrolling patients after the RP2D is declared in Study GO40516 and after an optimal mosunetuzumab SC step-up dose has been identified in Study GO29781 (Group F). The mosunetuzumab dosing schedule in combination with polatuzumab vedotin (concurrent or staggered dosing) in Arm K will follow the same dosing schedule as the RP2D in study GO40516 (e.g., Group A, in which both drugs are administered starting on C1 D1), and the mosunetuzumab SC step-up doses in Arm K may not exceed the highest subcutaneous step-up doses assessed in Study GO29781 (see Section 4.3.3.2 for details of the dose and dosing schedules).

The first 6 safety-evaluable patients in Arm K will be considered as a safety run-in group for the R/R MCL cohort. After these patients have completed at least one cycle of mosunetuzumab in combination with polatuzumab vedotin, the IMC will review cumulative safety data in these patients to recommend either allowing the enrollment of additional patients or halting the rest of the Arm K expansion enrollment. The IMC may also recommend modifications to the dose and/or dosing schedule for Arm K, and a second safety cohort of approximately 6 patients may be enrolled, if needed. An additional IMC review will take place after these patients in the second safety run-in

group have completed at least one cycle of mosunetuzumab in combination with polatuzumab vedotin.

For patients in the safety run-in group, treatment will be staggered such that the second patient enrolled in the cohort will receive the first dose of study treatment at least 72 hours after the first enrolled patient receives the first dose of study treatment, to assess for any severe and unexpected acute drug or infusion-related toxicities. Dosing in subsequent patients in each cohort will be staggered by at least 24 hours from the end of the prior patient's administration. In each scenario, the Sponsor must receive documentation of the status of the prior patient before the next patient receives the first dose of study treatment.

On the basis of review of real-time safety data and preliminary PK data, the dose may be halted or modified by the Sponsor, as deemed appropriate. Should an individual patient at any time develop unacceptable and/or intolerable localized injection-site reaction toxicity (Table 20) following SC administration of mosunetuzumab, conversion to mosunetuzumab IV administration may be considered following discussion with and approval by the Medical Monitor. In these cases, the mosunetuzumab IV dosing regimen will follow the dosing schedule of the Study GO40516 RP2D, and the dose may not exceed the highest cleared dose in Study GO40516.

3.1.3.3 Randomized Phase II for R/R DLBCL

Approximately [redacted] patients with R/R DLBCL will be randomized at 1:1 ratio to the treatment with mosunetuzumab SC plus polatuzumab vedotin (Arm L) and rituximab plus polatuzumab vedotin (Arm M):

- **Arm L:** R/R DLBCL; [redacted] patients (mosunetuzumab SC plus polatuzumab vedotin)
- **Arm M:** R/R DLBCL; [redacted] patients (rituximab IV plus polatuzumab vedotin)

Patients will be randomized 1:1 with the use of stratified permuted blocks. Randomization will be stratified by the number of prior treatment regimens (one prior line of therapy versus ≥ 2 prior lines of therapy). See Section 3.3.9 for the rationale for the control arm.

The IMC will review the cumulative safety data after the first [redacted] patients have been randomized into Arm L and have completed at least one cycle of mosunetuzumab SC. Enrollment into both Arms L and M will not pause at the time of IMC review and will continue after these patients have been randomized into Arm L. The IMC may recommend either allowing the enrollment of additional patients or halting the rest of Arms L and M. The IMC may also recommend modifications to the dose and/or dosing schedule for Arm L. If needed, an IMC review will take place after an additional approximately [redacted] patients have completed at least one cycle of mosunetuzumab SC in combination with polatuzumab vedotin.

The randomized Phase II portion of the study will also conduct an interim futility analysis at least [redacted] *months after approximately* [redacted] *patients in Arm M have received at least one dose of study treatment. At this futility analysis, if Arm M demonstrates a posterior probability of >85% that the true investigator-assessed ORR in Arm M is >42% (e.g., observing at least* [redacted] *responders in* [redacted] *patients), enrollment in Arms L and M may expand to* [redacted] *patients total (*[redacted] *patients in each arm). Otherwise, enrollment in Arms L and M may be stopped at* [redacted] *patients total (*[redacted] *patients in each arm). For further details, refer to Section 6.9.2.*

Patients assigned to Arm M are allowed to receive mosunetuzumab SC plus polatuzumab vedotin if they meet the criteria for the crossover before or at the end of 8 cycles of rituximab plus polatuzumab vedotin therapy (Arm M-crossover). See Section 3.1.5.2 and Figure 5 for the criteria and crossover procedure.

3.1.4 Treatment of Non-Hodgkin Lymphoma after Disease Progression

Experience with cancer immunotherapy for solid tumors has demonstrated that responding tumors may initially increase in size due to the influx of immune cells, a phenomenon known as "pseudoprogression" (Wolchok et al. 2009). Pseudoprogression has also been described in the context of lymphoma (Cheson et al. 2016; Salles et al. 2016), and similarly it is possible that mosunetuzumab and/or polatuzumab vedotin therapy may initially increase tumor size and metabolic activity by inducing the influx of T cells into the tumor. Given this, a repeat tumor biopsy, if clinical disease progression is observed, is strongly encouraged. Additionally, if the study investigator believes that a patient with NHL receiving mosunetuzumab (Groups A, B, C; Arms I, J, K, and L) is deriving clinical benefit despite radiographic evidence of progressive disease as defined by the Lugano 2014 criteria (Appendix 7), that patient may continue study treatment provided the following criteria are met:

- There is an absence of symptoms and signs (including worsening of laboratory values) indicating unequivocal progression of disease.
- There is no decline in Eastern Cooperative Oncology Group Performance Status (ECOG PS).
- There is an absence of tumor progression at critical anatomical sites including the central airway, the great vessels, and other organs or tissues where compromised function secondary to tumor progression would be expected to result acutely in severe and/or irreversible disability or death.

Patients must provide written consent to acknowledge discussion with the treating investigator of the benefit-risk balance of continuing study treatment beyond radiographic progression. Patients continuing study treatment despite apparent radiographic progression will be strongly encouraged to undergo a repeat tumor biopsy to assess whether increases in tumor volume are due to immune cell infiltration or neoplastic proliferation, provided that such a biopsy can be performed safely on

a non-target lesion. If true progression is suspected based on the investigator's judgment, clinical factors, or biopsy findings that are consistent with neoplastic proliferation, or if radiographic disease progression is confirmed at a subsequent tumor assessment, the patient will be ineligible to receive further study treatment.

3.1.5 Mosunetuzumab Treatment Duration, Re-Treatment Following Disease Progression, and Crossover

3.1.5.1 Mosunetuzumab Treatment Duration and Re-Treatment

Patients who initially respond or have SD to mosunetuzumab combined with polatuzumab vedotin may benefit from additional cycles beyond the initial 8 cycles of mosunetuzumab treatment, depending on anti-tumor responses to initial treatment.

To test this hypothesis, patients will be eligible for mosunetuzumab re-treatment (for all patients) or continued study treatment beyond the initial 8 cycles (for patients in Groups A, B, and C, and Arms I and J), either as single-agent or combined with polatuzumab vedotin, as described below. The study re-treatment dose and schedule will be one that has been previously demonstrated in dose escalation to be safe, provided the following criteria are met:

- Pertinent eligibility criteria (Section 4.1.1 and Section 4.1.2) are met at the time that mosunetuzumab treatment (with or without polatuzumab vedotin) is re-initiated, with the following exceptions:
  - Prior therapy with mosunetuzumab is allowed.
  - Prior therapy with polatuzumab vedotin is allowed.
  - Serology tests to demonstrate HIV, hepatitis C virus (HCV), and hepatitis B virus (HBV) status do not need to be repeated unless clinically indicated. Epstein-Barr virus (EBV) and cytomegalovirus (CMV) by polymerase chain reaction (PCR) must be repeated.
  - Manageable and reversible immune-related adverse events with initial study treatment are allowed and do not constitute an exclusionary history of autoimmune disease.
  - Patients must not have experienced Grade 4 non-hematologic adverse events that were not considered by the investigator to be attributable to another clearly identifiable cause during initial study treatment, with the possible exception of TLS (Section 5.1.7).
  - Patients who experienced Grade 2 or 3 adverse events that were not considered by the investigator to be attributable to another clearly identifiable cause during initial treatment must have resolved these toxicities to Grade ≤ 1.
  - Patients may require hospitalization following the first re-treatment administration (Section 5.1.1). The need for hospitalization following the first re-treatment administration will be made based on the recommendation of the IMC (Sections 3.1.5.2 and 5.1.8) and in consultation with study investigators.

- No intervening systemic anti-cancer therapy was administered between the completion of initial study treatment and re-initiation of study treatment.
- Written informed consent is provided to acknowledge deferring any standard treatment options that may exist in favor of reinitiating study treatment and to undergo a biopsy of recurrent or progressing tumor if clinically feasible.

Patients proceeding to re-treatment following disease progression will need to complete screening assessments (Appendix 1) to re-confirm eligibility, including undergoing a repeat tumor biopsy from a safely accessible site to assess: 1) CD20 expression status and 2) changes/status of the tumor and immune microenvironment. Patients who provide written informed consent but have no lesion amenable for biopsy at disease progression may still be considered for study drug re-treatment.

The dose and schedule of study treatment to be administered for patients receiving re-treatment will be a previously tested dose and schedule that has cleared the DLT assessment period. Patients receiving re-treatment should follow the schedule of assessments as outlined in Appendix 1–Appendix 6.

The schema of the duration of initial study treatment and options for re-treatment (for all patients) or continued study treatment beyond the initial 8 cycles of study treatment (for Groups A, B, C, Arms I and J) are described in Figure 4. The dose and schedule of administration of mosunetuzumab with or without polatuzumab vedotin based on the nature and timing of study treatment is described below.

Polatuzumab vedotin will be given for 6 cycles during re-treatment, unless progressive disease or unacceptable toxicity is observed prior to completion of the 6 cycles. Mosunetuzumab will be given as described below:

- Eight cycles of mosunetuzumab (or 17 cycles in Arm K) will be given unless progressive disease or unacceptable toxicity is observed prior to completion of the 8 cycles (or 17 cycles in Arm K).
  - If progressive disease is observed, then patients will discontinue study treatment.
- Patients in Groups A, B, and C, and Arms I and J who achieve CR at PRA *as the best overall response* after 8 cycles of mosunetuzumab treatment will not receive any additional cycles of mosunetuzumab and be monitored according to the schedule in Appendix 2.
  - If progressive disease following completion of combination treatment is observed, the following apply:

    If the patient has Grade $\leq$1 peripheral neuropathy, mosunetuzumab combined with polatuzumab vedotin re-treatment may be initiated.

    If the patient has continued Grade $>$1 peripheral neuropathy or otherwise by physician choice, single-agent mosunetuzumab re-treatment may be initiated.

- Patients in Groups A, B, and C, and Arms I and J who achieve a PR or maintain SD at PRA *as the best overall response* after receiving 8 cycles of mosunetuzumab treatment may continue single-agent mosunetuzumab for up to a total of 17 cycles unless progressive disease or unacceptable toxicity is observed.
  - PRA should be conducted at the end of C8 (C8D21 ± 1 week) prior to the C9D1 treatment for Groups A and C (and Arms I and J, if following the Group A or C dosing schedule) or at the end of C9 (C9D21 ± 1 week) prior to C10D1 treatment for Group B (and Arms I and J, if following the Group B dosing schedule), in order to inform the duration of study treatment.

    If CR, PR, or SD is achieved by the completion of 17 total cycles, patients will continue to be monitored according to the schedule in Appendix 2.

    If progressive disease is observed on additional cycles of mosunetuzumab treatment, then patients will discontinue study treatment.
- Patients in Arm K who complete 17 cycles of mosunetuzumab treatment will not receive any additional cycles of mosunetuzumab and be monitored according to the schedule in Appendix 2.
  - If progressive disease following completion of combination treatment is observed, the following will apply:

    If the patient has Grade ≤1 peripheral neuropathy, mosunetuzumab combined with polatuzumab vedotin re-treatment may be initiated.

    If the patient has continued Grade >1 peripheral neuropathy or otherwise by physician choice, single-agent mosunetuzumab re-treatment may be initiated.
- Patients in Arm L or Arm M-crossover (see Section 3.1.5.2) who complete 8 cycles of mosunetuzumab treatment will not receive any additional cycles of mosunetuzumab and be monitored according to the schedule in Appendix 2.
  - If progressive disease following completion of combination treatment is observed, the following will apply:

    If the patient has Grade ≤1 peripheral neuropathy, mosunetuzumab combined with polatuzumab vedotin re-treatment may be initiated.

    If the patient has continued Grade >1 peripheral neuropathy or otherwise by physician choice, single-agent mosunetuzumab re-treatment may be initiated.

Additional rounds of re-treatment with mosunetuzumab with or without polatuzumab vedotin are permitted, following the re-treatment rules described.

Re-Treatment Dose and Schedule of Administration

If the time between the last dose of initial treatment and first dose of re-treatment including step dose is ≥6 weeks, administer mosunetuzumab at previous schedule including C1 step-up.

If the time between the last dose of initial treatment and first dose of re-treatment including step dose is $<$6 weeks, administer mosunetuzumab at previous schedule C2 dose every 21 days; no mosunetuzumab step-up is needed.

The formulation of mosunetuzumab for re-treatment must be the same as the original formulation administered during initial treatment for that individual patient (e.g., if SC was administered in the initial treatment phase for a patient, then SC must also be administered for re-treatment).

Figure 4 Mosunetuzumab plus Polatuzumab Vedotin Treatment/Re-Treatment Schema (Dose Escalation Phase, Arms I, J, *and K*)

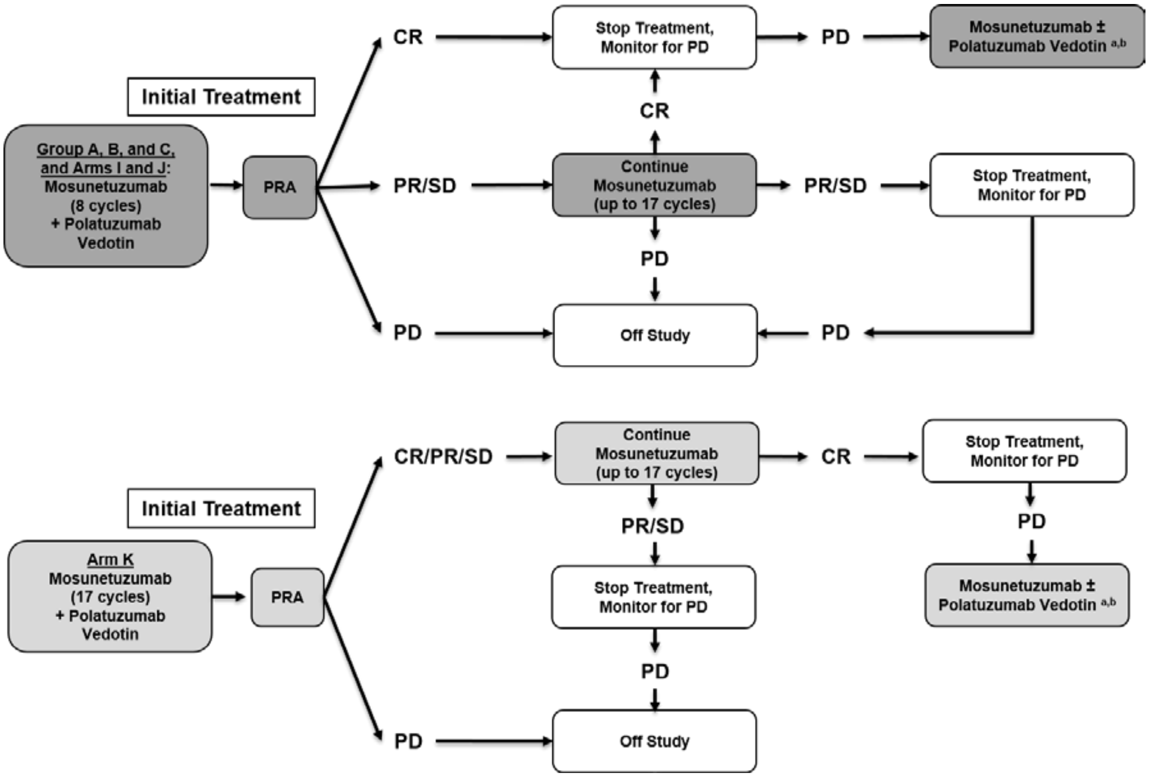

CR = complete response; PD = progressive disease; PR = partial response; SD = stable disease; PRA = primary response assessment.

[a] Additional rounds of re-treatment are permitted; follow treatment flow for initial treatment

[b] If the patient has continued Grade > 1 peripheral neuropathy or otherwise by physician choice, single-agent mosunetuzumab re-treatment may be initiated.

3.1.5.2 Crossover for Patients from Arm M (Rituximab plus Polatuzumab Vedotin) to Arm M-crossover (Mosunetuzumab SC plus Polatuzumab Vedotin)

- Patients in Arm M have the option to receive treatment with mosunetuzumab SC plus polatuzumab vedotin (Arm M-crossover) within 6 weeks if either of the following criteria is met:
  - The patient has disease progression before or at the end of 8 cycles of Arm M treatment
  - The disease assessment is stable disease at the end of 8 cycles of Arm M treatment
- Treatment cycles of polatuzumab vedotin in Arm M-crossover will be reduced so that the total number of treatment cycles of polatuzumab vedotin in Arm M and Arm M-crossover do not exceed 8 cycles. *For example, if the patient received 6 cycles in Arm M and moves to crossover, they should receive no more than 2 more cycles in the Arm M-crossover. If the patient received 4 cycles in Arm M and moves to*

crossover, they should receive no more than 4 more cycles in the Arm M-crossover. Patients in Arm M-crossover will receive a total of 8 cycles of mosunetuzumab SC as in Arm L, regardless of the number of rituximab treatment cycles given in Arm M.

- Patients whose disease assessment is either CR or PR upon completion of 8 cycles of Arm M treatment will not receive any additional treatment and be monitored according to the schedule in Appendix 2.

Patients proceeding to crossover following disease progression will need to complete screening assessments (Appendix 1), except for the screening tumor biopsy requirement. To be eligible for the crossover, the patient must fulfill the inclusion criteria (Section 4.1.1) except for the screening tumor biopsy requirement, and must not meet any of the exclusion criteria (Section 4.1.2) except for those regarding prior treatment.

Figure 5 Mosunetuzumab plus Polatuzumab Vedotin Treatment/Re-Treatment/Crossover Schema (Arms L and M)

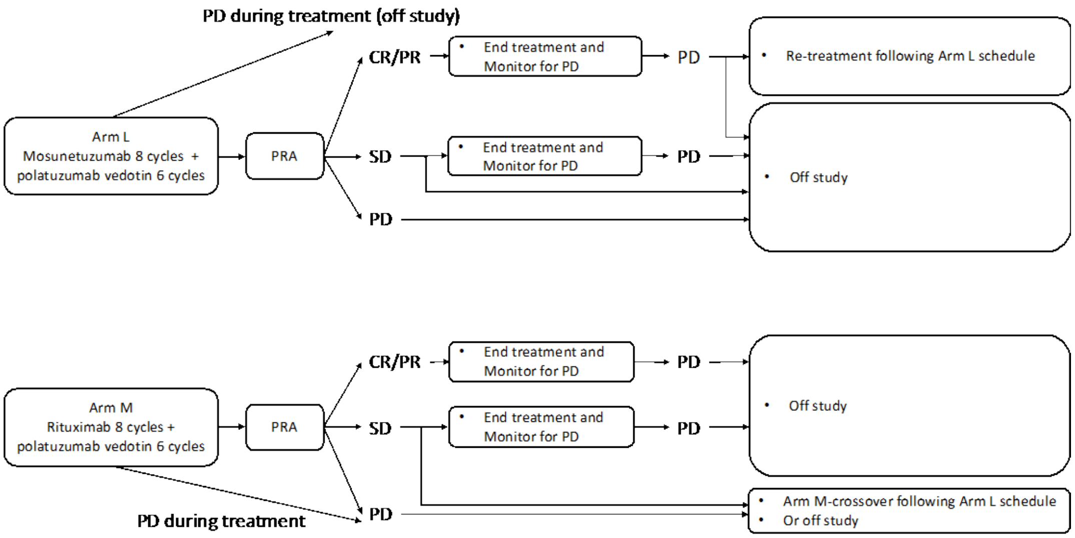

CR =complete response, PD= progressive disease; PR =partial response PRA =primary response assessment.

3.1.6 Internal Monitoring Committee

Because this is the first trial to combine mosunetuzumab with polatuzumab vedotin, an IMC will be utilized during the study to make recommendations regarding study conduct on the basis of trial safety data to ensure enhanced patient safety while receiving study treatment. See Section 5.1.8 for details.

3.1.7 Independent Review Committee

An IRC composed of board-certified radiologists, nuclear medicine specialists, and an oncologist with experience in malignant lymphoma will assess all patients for response (see Appendix 7) on the basis of imaging results and bone marrow biopsy results

(if applicable) for all patients in the expansion cohorts (Arms I, J, K, L, and M). Decisions will be guided by a Charter specific to the independent review.

3.2 END OF STUDY AND LENGTH OF STUDY

The end of this study is defined as the date when the last patient, last visit occurs, or the date at which the last data point required for statistical analysis or safety follow-up is received from the last patient, whichever occurs later. The end of the study is expected to occur approximately 30 months after the last patient is enrolled.

The total length of the study, from screening of the first patient to the end of the study, is expected to be approximately 80 months.

In addition, the Sponsor may decide to terminate the study at any time.

3.3 RATIONALE FOR STUDY DESIGN

3.3.1 Rationale for Mosunetuzumab Dose and Schedule

Mosunetuzumab dosing for this study was based on the experience from the ongoing Phase I Study GO29781 with singe-agent mosunetuzumab in patients with R/R B-cell NHL. The Study GO29781 Group B dosing schedule is the basis for the mosunetuzumab IV dosing in this study, and the Study GO29781 Group F dosing schedule is the basis for the mosunetuzumab SC dosing in this study.

Through its currently known mechanism of action, mosunetuzumab binds to CD3 on T cells and CD20$+B$ cells simultaneously, leading to the activation of T cells and subsequent T-cell mediated killing of B cells. In the GO29781 study, a step-up dose schedule of single-agent mosunetuzumab during C1 was tested to minimize treatment-emergent toxicity associated with T-cell activation and associated cytokine release. Similar step-up dosing regimens have been utilized with other T-cell bispecific molecules such as blinatumomab (Viardot et al. 2010, 2014, 2016) and REGN1979 (Varghese et al. 2014; Brownstein et al. 2015; Smith et al. 2015a). Using the C1 step-up dose schedule, the overall safety profile of mosunetuzumab has been manageable.

In Study GO29781, the length of each single-agent mosunetuzumab treatment cycle was 21 days, which will be maintained in Study GO40516. In Study GO29781, mosunetuzumab monotherapy was given for 8 cycles, and patients who only achieved SD or PR after 8 cycles continued to receive additional therapy up to a total of 17 cycles of mosunetuzumab treatment. Of note, in Study GO29781, patients with R/R FL or R/R DLBCL who achieved CR within the initial 8 cycles of mosunetuzumab monotherapy have shown durable responses without additional treatment. Similarly, in Study GO40516, the initial planned number of cycles of mosunetuzumab for Arms I and J will be 8 cycles, with allowance for a total of 17 cycles if the response after 8 cycles is PR or SD. Patients in Arm L will receive a fixed total of 8 cycles of mosunetuzumab treatment in order to allow comparability with Arm M, in which rituximab treatment is 8 cycles.

A different consideration is given to patients with R/R MCL in Arm K. As of 21 January 2020, in Study GO29781, the 9 CRs observed within the initial 8 cycles of mosunetuzumab monotherapy in 32 patients with R/R MCL had a median duration of CR of only 5.9 months (95% CI: 2.2 months to upper limit not reached). Based on this data, and with the hypothesis that prolonged treatment after CR is considered potentially beneficial for patients with R/R MCL, all patients in Arm K in Study GO40516 will receive a total of 17 cycles in the absence of progression or unacceptable toxicity.

Refer to the Mosunetuzumab Investigator's Brochure for additional details on nonclinical and clinical studies.

3.3.1.1 Rationale for Intravenous Step-Up Dosing of Mosunetuzumab (Groups A, B, and C; Arms I and J)

In Study GO29781, evidence of single-agent mosunetuzumab anti-tumor activity was observed with the following step-up dose schedules, with the first number indicating the dose on C1D1, the second number indicating the dose on C1D8, and the third number indicating the dose on C1D15 and D1 of each subsequent cycle: 0.4/1.0/2.8 mg, 0.8/2.0/4.2 mg, 0.8/2.0/6.0 mg, 1.0/2.0/6.0 mg, and 1.0/2.0/9.0 mg. In Study GO40516, to mitigate against unexpected combination toxicity with polatuzumab vedotin, the starting mosunetuzumab step-up dose will be at least one dose level below the maximum assessed dose for mosunetuzumab with the step-up schedule as a single agent from Study GO29781 (see Section 1.2.2.1).

3.3.1.2 Rationale for Subcutaneous Step-Up Dosing of Mosunetuzumab (Arms K and L)

The rationale for assessing the SC dosing schedule for mosunetuzumab is similar to that of assessing a C1 IV step-up dosing schedule, with the primary goal of minimizing cytokine-driven toxicities upon initial mosunetuzumab dosing.

Nonclinical testing of mosunetuzumab in cynomolgus monkeys showed that SC administration of mosunetuzumab was as efficacious as IV dosing at the same dose level in inducing B-cell depletion, and it effectively reduced cytokine release, eliminated acute toxicities, and reduced the incidence of vascular/perivascular findings in a repeat-dose study. This suggested that SC may provide a viable route of administration to IV with further CRS mitigation and improved patient convenience.

Study GO29781 evaluated subcutaneous mosunetuzumab as single-agent in fixed-dosing (Group D) and step-up dosing (Group F) (see Section 1.2.2 and Mosunetuzumab Investigator’s Brochure for detailed safety information). Mosunetuzumab SC at 5/45/45 mg is considered as preferred dose as a single-agent, given overall comparable safety of CRS and neutropenia to mosunetuzumab IV and SC dosing regimens (fixed-dose and 5/15/45 mg) evaluated and potential clinical benefit of shortened window of CRS occurrence to the first 2 weeks (Section 1.2). Notably, Grade $\geq$ 3 neutropenia rate appears comparable between mosunetuzumab SC as

fixed-doses (Group D) and IV as step-up doses (Group B). Exposure-response analysis based on 391 patients treated with mosunetuzumab IV and 54 patients treated with mosunetuzumab SC suggested lack of dose-reponse or exposure-response relationship with neutropenia incidence rate. Lower rate of Grade ≥3 neutropenia was observed in patients treated with mosunetuzmab SC step-up doses (Group F), likely due to shorter duration of follow up.

In patients with R/R MCL, in Study GO29781, mosunetuzumab single-agent at the dose selected for expansion of 1/2/60/30 mg IV Q3W provided acceptable safety profile. Preliminary PK data suggested similar PK parameters (C_{max} and AUC) between R/R MCL and other R/R NHL histologies. Therefore, it is considered reasonable to utilize the same dose and schedule of mosunetuzumab in combination with polatuzumab in patients with R/R MCL as in other.

Mosunetuzumab IV was well-tolerated at the highest tested dose of 1/2/60/30 mg IV Q3W in combination with polatuzumab vedotin IV in patients with R/R NHL in the dose escalation cohort and Arm J of Study GO40516. The selected SC mosunetuzumab dose at 5/45/45 mg is projected to achieve comparable AUC to that of 1/2/60/30 mg IV, while C_{max} is consistently lower than that of IV throughout treatment duration (Figure 6). Together, mosunetuzumab SC 5/45/45 mg is considered an appropriate dose in combination with polatuzumab vedotin in Arm K and Arm L (Section 4.3.3.2). To ensure the safety of the regimen, interim safety analysis is planned for both Arm K and Arm L as described in Section 3.1.3.1 and Section 3.1.3.2, respectively.

Figure 6 POPPK Model Simulated Mosunetuzumab Concentration-Time Profiles Following IV 1/2/60/30 mg and SC 5/45/45 mg Q3W Dose Regimens

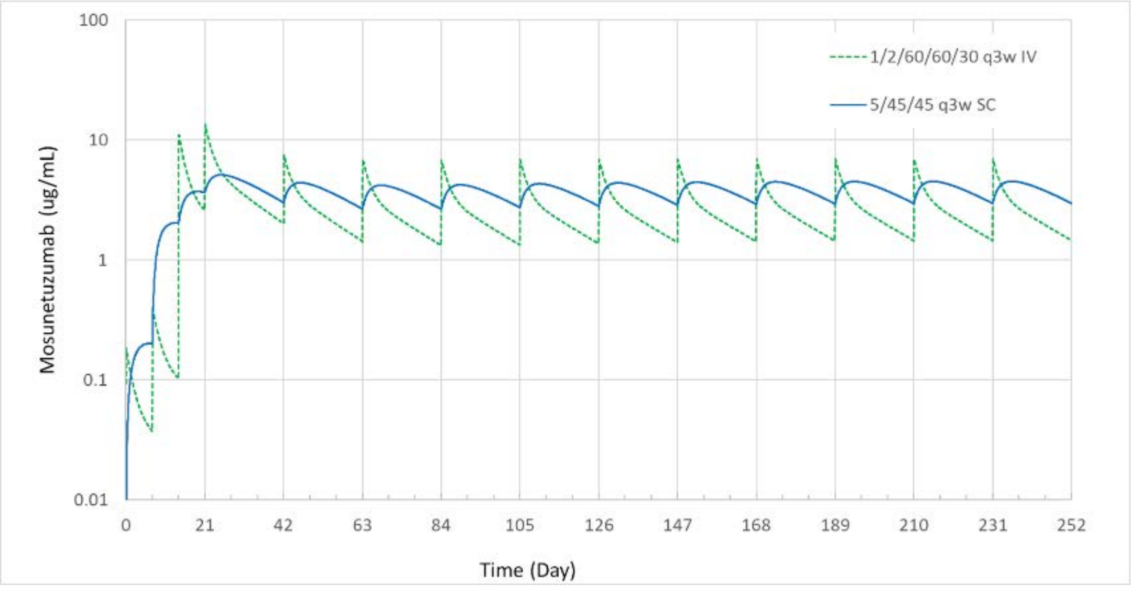

3.3.2 Rationale for Polatuzumab Vedotin Dose and Schedule

At the initiation of this study, over 500 patients had been enrolled in eight trials administering polatuzumab vedotin in B-cell malignancies. At the dose of 1.8 mg/kg every 21 days, polatuzumab vedotin has been well tolerated as monotherapy and in combination with an anti-CD20 monoclonal antibody in patients with R/R B-cell NHL, with expected toxicities including cytopenias and peripheral neuropathy.

In Study GO29365, 39 patients were treated with polatuzumab vedotin at 1.8 mg/kg IV given on D1, in combination with rituximab administered at standard dose of 375 mg/m^2 on D1 and bendamustine 90 mg/m^2 administered on Days 2 and 3 for C1 and on Days 1 and 2 for subsequent cycles on a 21-day cycle. Polatuzumab vedotin plus bendamustine and rituximab was given for up to 6 cycles, which was associated with durable responses and acceptable safety profile (Sehn et al. 2017).

To compare the efficacy of mosunetuzumab plus polatuzumab vedotin with the polatuzumab vedotin plus bendamustine and rituximab regimen from Study GO29365, in this study polatuzumab vedotin will be given at 1.8 mg/kg every 21 days for a total of up to 6 cycles.

Polatuzumab vedotin has been given for up to 17 cycles in prior studies (Palanca-Wessels et al. 2015). For consideration for re-treatment with polatuzumab vedotin beyond the 6 cycles in the initial treatment (see Section 3.1.5), patients will be required to demonstrate resolution of polatuzumab vedotin-related toxicities to Grade ≤1, such as peripheral neuropathy prior to initiation of re-treatment.

See Section 1.3 and the Polatuzumab Investigator's Brochure for additional details on clinical studies.

3.3.3 Rationale for Dose-Finding Schedules (Groups A, B, and C)

During the Phase Ib dose-finding phase, Groups A, B, and C will test alternate schedules of mosunetuzumab and polatuzumab vedotin. Group A will test the safety and tolerability of both agents administered on C1D1. The main potential overlapping toxicity expected with concurrent administration of these agents is neutropenia. Groups B and C will test the safety and tolerability of alternative order and sequence of administration of mosunetuzumab and polatuzumab vedotin, to potentially mitigate against overlapping toxicities, if necessary. In Group B, polatuzumab vedotin will be given on C1D1, and mosunetuzumab step-up will begin on C1D8. The rationale for the Group B schedule is that administration of polatuzumab vedotin prior to mosunetuzumab may de-bulk tumor and decrease the frequency and/or severity of mosunetuzumab-related CRS. In Group C, mosunetuzumab step-up begins on C1D1, and the first dose of polatuzumab vedotin is given on C2D1. The rationale for the Group C schedule is that the delayed start of polatuzumab vedotin would not overlap mosunetuzumab-related CRS or potential associated neutropenia that is typically observed early in C1 with single-agent mosunetuzumab.

3.3.4 Rationale for Dose-Finding Rules

The rules for dose escalation are designed to ensure patient safety while providing an opportunity to identify the optimal mosunetuzumab dose and schedule in combination with polatuzumab vedotin to maximize the benefit-risk profile of the combination.
Key elements of dose escalation are the following. First, the first two dose levels of mosunetuzumab step-up will be fixed at the same dose levels determined for single-agent mosunetuzumab (see the Mosunetuzumab Investigator's Brochure), 1.0 mg for the first dose level (DL_1) and 2.0 mg for the second dose level (DL_2), and only the third dose level (DL_3) will be adjusted during the dose-finding phase. Rules based on the nature and timing of observed safety events have been implemented.

In Groups A and B, the declaration of MTD is based on the timing of DLT events relative to the time of step-dose administration of DL_3. Adverse events attributed to mosunetuzumab can therefore generally be assessed as follows:

- Adverse events occurring prior to the first administration of DL_3 may be attributable to the DL_1 and DL_2 mosunetuzumab doses but not to the DL_3 mosunetuzumab dose.
- Adverse events occurring after the first administration of DL_3 may be attributable to the DL_1, DL_2, and DL_3 mosunetuzumab doses.

To avoid declaring the Group A and B MTD prematurely based on DLTs that occur prior to the DL_3 dose, the rules for determining the overall MTD will take into account:
a) the timing of the DLT relative to the first administration of DL_3 and b) adverse events at the same DL_1 and DL_2 dose across all applicable cohorts, if DLT occurs prior to the first administration of DL_3. These rules are described in Sections 3.1.2.3 and 3.1.2.4.

In Group C, decisions on dose escalation of mosunetuzumab when combined with polatuzumab vedotin will be largely based on safety events observed in C2, which is the first cycle in which mosunetuzumab and polatuzumab vedotin are given concurrently. Analysis based on timing of the adverse events is not required since only one dose level of mosunetuzumab is given in combination with polatuzumab vedotin in Group C.

3.3.5 Rationale for Mosunetuzumab DLT Assessment Period

The DLT assessment periods for Groups A, B, and C, defined in Section 3.1.2.1.1.
In Group A, the DLT assessment period for dose escalation is the 21-day period following the first dose of mosunetuzumab for dose-escalation Group A, to cover the entire step-up period of mosunetuzumab in combination with polatuzumab vedotin.

In Group B, mosunetuzumab is given on C1D8, 1 week after polatuzumab vedotin is given on C1D1. The third dose of the mosunetuzumab step-up is given on C2D1. Therefore, to cover the entire step-up period of mosunetuzumab in combination with polatuzumab vedotin, the DLT assessment period is extended from C1D8 through C2D21.

In Group C, during C1, mosunetuzumab is given at a dose and schedule previously evaluated as safe and tolerable as tested as a single agent. Mosunetuzumab is administered for the first time in combination with polatuzumab vedotin on C2D1. Therefore, the DLT assessment window for this group begins on C2D1 through C2D21.

3.3.6 Rationale for Continued Dosing beyond the DLT Assessment Period and Study Re-Treatment

Nonclinical toxicology and clinical data support single-agent mosunetuzumab treatment every 21 days. The ethical conduct of a clinical study of cancer requires that patients have the opportunity to continue study treatment provided that the treatment is active and tolerable and patients comply with protocol requirements. Therefore, dosing beyond C1 for patients will be allowed in the absence of unacceptable toxicity or objective evidence of disease progression as assessed by the treating study investigator and following a careful assessment and discussion of the potential risks and benefits with the patient, as outlined in Section 3.1.2.6.

The duration of treatment with mosunetuzumab combined with polatuzumab vedotin and single-agent mosunetuzumab is described in Section 3.1.5. In the absence of unacceptable toxicity or disease progression, patients will initially receive 8 cycles of mosunetuzumab, with clinical outcomes from initial treatment determining options for follow-up, continued treatment, or re-treatment. The rationale for basing the duration of study treatment on anti-tumor activity is three-fold. First, chronic, and/or cumulative toxicity potentially associated with prolonged treatment duration can be minimized and provides an opportunity to assess the DOR once study treatment is discontinued. Second, limiting study treatment to a fixed number of treatment cycles provides an opportunity to explore clinical outcomes following re-treatment with mosunetuzumab combined with polatuzumab vedotin or single-agent mosunetuzumab. Third, provision for a biopsy of progressive/relapsed disease prior to study treatment serves to confirm CD20 expression in the tumor and to evaluate the tumor microenvironment to assess for potential mechanisms of resistance to study treatment.

3.3.7 Rationale for Patient Population

This study will enroll patients with a history of a hematologic malignancy that is expected to express the CD20 antigen, specifically FL, DLBCL, transformed FL, and MCL. Confirmation of CD20 expression will not be required during eligibility screening prior to enrollment, but it will be evaluated retrospectively, based on the following rationale:

- Nonclinical studies have demonstrated that mosunetuzumab is broadly active in killing multiple human B lymphoma cell lines with a broad range of CD20 expression levels, including cell lines with very low levels of CD20 expression that are not efficiently killed by rituximab or obinutuzumab, suggesting that even very low levels of CD20 expression may be sufficient for clinical activity.

- CD20 is a cell-surface antigen whose expression is restricted to pre-B cells and all mature B cells except plasma cells. It is expressed in a majority of the B-cell–derived malignancies, including NHL.
- Although a few lymphomas may express relatively low levels of CD20, current information regarding R/R FL, DLBCL and MCL to be included in this study suggest that truly negative tumors (i.e., expressing no detectable CD20) are infrequent (Miyoshi et al. 2012).

Tumor samples obtained from all patients (see Section 4.5.8, Section 4.5.12, Appendix 1, and Appendix 2) will be retrospectively analyzed for CD20 expression, and data collected from this analysis will be used to inform how best to utilize CD20 expression screening in subsequent studies.

After the RP2D has been determined for mosunetuzumab IV or SC combined with polatuzumab vedotin, three indication-specific Phase II, single-arm expansion cohorts and the randomized Phase II portion of the study will be initiated including up to approximately 100 patients with R/R DLBCL, transformed FL, or Grade 3b FL, up to approximately [redacted] patients with R/R FL (Grade 1–3a), up to approximately [redacted] patients with R/R MCL, and up to [redacted] patients in R/R DLBCL (randomized Phase II portion). With current standards of care including salvage immunochemotherapy followed by autologous SCT, survival outcomes remain poor (Gisselbrecht et al. 2010); for patients who are not eligible to receive autologous SCT, survival outcomes are even worse with essentially no chance of prolonged disease control (Friedberg 2011). Similarly, most patients with FL will eventually experience disease relapse after receiving chemoimmunotherapy as first-line treatment. Response rate and DOR of these patients are further decreased in subsequent lines of therapy.

Given the activity of single-agent mosunetuzumab and polatuzumab vedotin, the Phase II expansion cohorts will assess the clinical benefit for mosunetuzumab combined with polatuzumab vedotin in the area of critical unmet medical need of relapsed R/R DLBCL/transformed FL, R/R FL, and R/R MCL. Data from these expansion cohorts will not only provide an assessment of mosunetuzumab and polatuzumab vedotin clinical activity but also inform future clinical studies comparing these regimens to existing standard therapies.

3.3.8 Rationale for the Single-Arm Expansion DLBCL, FL and MCL Cohorts

In the Phase II single-arm expansion phase, the R/R DLBCL, R/R FL, and R/R MCL cohorts test the combination of mosunetuzumab plus polatuzumab vedotin.
The rationale for the single-arm expansion phase is to further assess preliminary efficacy and safety of the mosunetuzumab plus polatuzumab vedotin combination following completion of dose escalation.

3.3.9 Rationale for the Randomized Phase II R/R DLBCL Control Arm

As of [redacted], responses were observed in [redacted] of [redacted] patients with R/R DLBCL in Arm J (ORR [redacted]%), or in [redacted] of [redacted] total patients with R/R DLBCL in Group A and Arm J combined (ORR [redacted]%). The observed response rate is numerically higher than the ORR 42% observed in [redacted] patients with R/R DLBCL treated with polatuzumab vedotin at 1.8mg/kg as monotherapy or in combination with anti-CD20 antibody (Studies DCS4968g and GO27834).

During the randomized Phase II portion enrolling patients with R/R DLBCL, the experimental arm tests the combination of mosunetuzumab SC plus polatuzumab vedotin against a control arm, rituximab plus polatuzumab vedotin. The rationale for this control arm is to further assess the individual contribution of mosunetuzumab when given in combination with polatuzumab vedotin in patients with R/R DLBCL.
The rationale for rituximab in combination with polatuzumab vedotin as the control arm is based on the current clinical practice (NCCN Guideline B-cell lymphoma, Version 4, 5 May 2021, Page BCEL-C), supported by combination data from Study GO27834 (Phillips et al. 2016; Morschhauser et al. 2019) suggesting that rituximab plus polatuzumab vedotin combination is at least as effective as polatuzumab vedotin monotherapy.

3.3.10 Rationale for Crossover

If a patient in Arm M experiences a disease progression before or at the end of therapy, or has stable disease at the end of therapy, the patient will have an option to receive the immediate next treatment with mosunetuzumab plus polatuzumab vedotin (Arm M-crossover, see Section 3.1.5.2). Crossover provides an opportunity for patients not benefiting from the treatment with polatuzumab vedotin in combination with rituximab to receive a mechanistically distinct combination of polatuzumab vedotin and mosunetuzumab. The crossover procedure is described in Section 3.1.5.2.

3.3.11 Rationale for PET-Defined Complete Response

PET scanning has been shown in multiple settings to be a more accurate tool for assessing activity of lymphoma than CT imaging. In aggressive lymphomas such as DLBCL, PET-defined CR is a better predictor of PFS than response as defined by CT. The correlation of PET-defined response and PFS has not been as extensively studied in indolent lymphomas. However, a recent evaluation of PET-defined response in patients with FL treated with standard induction therapy using rituximab plus chemotherapy demonstrated that although PFS was not different between patients achieving PR versus CR, as defined by CT scan, when response criteria incorporating PET were used, patients who achieved PET-negative CR had a significantly longer PFS than did patients who achieved only a PR as defined by PET (Trotman et al. 2011). These findings indicate that response, as defined by PET, correlates with longer-term outcomes in patients with FL, similar to aggressive lymphomas.

3.3.12 Rationale for Use of an Independent Review Committee

An IRC will assess all patients enrolled in the Phase II expansion cohorts (Arms I, J, K, L, and M) of the study for response by PET-CT and/or CT scans through use of the Lugano 2014 criteria (Appendix 7) guided by a Charter specific to the independent review. The IRC functions to evaluate the response assessments in a standardized manner. IRC-assessed endpoints include the primary efficacy endpoint of best ORR and secondary endpoints of CRR, DOR, and PFS (see Section 6.4.1) in a standardized manner and will include board-certified radiologists, nuclear medicine specialists, and board-certified oncologists by blinded independent central review.

3.3.13 Rationale for Mandatory Hospitalization with Mosunetuzumab Administration (Dose-Finding Groups A, B, and C; Arm K Safety Run-in)

Treatment-emergent toxicities, notably CRS, have been observed with single-agent mosunetuzumab. These toxicities generally occur upon first exposure to mosunetuzumab. While the mechanisms of action of these toxicities are not completely understood, it is believed that they are the result of immune cell activation resulting in inflammatory cytokine release. Temporally, laboratory and clinical manifestations of cytokine release occur within several days of treatment and decrease in frequency and severity over time.

Prior clinical experience with step-up single-dose mosunetuzumab indicates that hospitalization is not required with the first dose of mosunetuzumab at 1.0 mg. In this study, because mosunetuzumab is given together with polatuzumab vedotin, hospitalization will be required on the first day of mosunetuzumab administration with polatuzumab vedotin. Hospitalization will also be required when the highest dose of mosunetuzumab (DL_3) is given for the first time with polatuzumab vedotin. For each group, the following hospitalizations will be required:

- Group A: C1D1 and C2D1
- Group B: C1D8 and C2D1
- Group C: C2D1

Since the safety and tolerability of the mosunetuzumab IV combination with polatuzumab vedotin will have already been assessed to identify the RP2D prior to enrollment of Arm K, hospitalization will only be required on the first day (i.e., on C1D1) of mosunetuzumab SC administration with polatuzumab vedotin for patients in the safety run-in group. Based on the safety profiles in the safety run-in part of Arm K, the IMC may determine if mandatory hospitalization must be continued or can be removed for the remaining part of Arm K.

Hospitalization requirements are further described in Section 5.1.1. Decisions to modify or discontinue the requirement for hospitalization in expansion arms will be made based

on the recommendation of the IMC (Sections 3.1.5.2 and 5.1.8) and in consultation with study investigators.

3.3.14 Rationale for Using ASTCT Consensus Grading for CRS

In this study, grading and treatment of the adverse event of CRS arising from mosunetuzumab treatment are described in Appendix 8, which is based on published ASTCT Consensus grading for CRS (Lee et al., 2019). The ASTCT consensus grading system (Lee et al. 2019) was proposed to provide a uniform consensus grading system for CRS; it is intended to be objective, easy to apply, and more accurately categorize the severity of CRS. Incorporation of the ASTCT CRS Consensus Grading criteria allows for alignment across clinical trial sites for the detection, reporting, and timely management of CRS. In addition to diagnostic criteria, recommendations on management of CRS based on its severity, including early intervention with corticosteroids and/or anti-cytokine therapy, are provided and referenced in Section 5.3.5.1.

3.3.15 Rationale for the Treatment of CRS Using Tocilizumab

CRS is caused by the excessive release of cytokines by immune effector or target cells during an exaggerated and sustained immune response. CRS can be triggered by a variety of factors, including infection with virulent pathogens, or by medications that activate or enhance the immune response, resulting in a pronounced and sustained immune response.

Regardless of the inciting agent, severe or life-threatening CRS is a medical emergency. If recognition or management is delayed, it can result in significant disability or fatal outcome. Current clinical management focuses on providing supportive care, treating the individual signs and symptoms, and attempting to dampen down the inflammatory response using high dose corticosteroids. However, this approach is not always successful, especially in the case of late intervention.

CRS is associated with elevations in a wide array of cytokines, including marked elevations in IFN-γ, IL-6, and TNF-α levels. Emerging evidence implicates IL-6 as a central mediator in CRS. IL-6 is a pro-inflammatory multi-functional cytokine produced by a variety of cell types, which has been shown to be involved in a diverse array of physiological processes including T-cell activation. Regardless of the inciting agent, CRS is associated with high IL-6 levels (Panelli et al. 2004; Lee et al. 2014; Doessegger and Banholzer 2015), and IL-6 correlates with the severity of CRS with patients who experience severe or life-threatening CRS (CTC Grades 3 or higher) having much higher IL-6 levels compared with their counterparts who do not experience CRS or experience milder CRS reactions (CTC Grades 0–2) (Chen et al. 2016).

Tocilizumab (Actemra®/RoActemra®) is a recombinant, humanized, anti-human monoclonal antibody directed against soluble and membrane-bound IL-6R, which inhibits IL-6 mediated signaling. Blocking the inflammatory action of IL-6 using tocilizumab could therefore represent an effective approach for the treatment of CRS.

Refer to the local prescribing information for tocilizumab and the Tocilizumab Investigator's Brochure for additional nonclinical and clinical information regarding tocilizumab.

CRS is observed with T-cell recruiting therapies including CAR-T cell therapy and bispecific molecules such as blinatumomab. There have been multiple reports in the literature of tocilizumab being used off-label to successfully treat severe or life-threatening CRS (Teachey et al. 2013; Lee et al. 2014, 2019; National Institutes of Health 2015), and tocilizumab is now approved in the United States, European Union and certain other countries for the treatment of CAR T-cell–induced severe or life-threatening CRS in adults and pediatric patients 2 years of age and older.

Taken together, these findings indicate that patients treated with mosunetuzumab who develop CRS may benefit from tocilizumab therapy. Section 5.1.7.5 describes recommendations regarding the management of CRS.

3.3.16 Rationale for Pharmacokinetic and Anti-Drug Antibody Sampling Schedule

The PK sampling schedule that follows the mosunetuzumab administration is designed to capture data at a sufficient number of timepoints to inform the concentration-time curve and enable the characterization of the key PK parameters (including but not limited to, C_{max}, C_{min}, and AUC) for mosunetuzumab as a single agent and in combination with polatuzumab vedotin. The PK sampling schedule for polatuzumab vedotin, evaluated as acMMAE and unconjugated MMAE, is designed to characterize key PK parameters of polatuzumab vedotin in combination with mosunetuzumab. Additionally, PK samples for polatuzumab vedotin total antibody will be collected based on a sparse sampling concurrent with ADA sampling for polatuzumab vedotin in order to inform the interpretation of any potential immune response towards polatuzumab vedotin. Predose serum rituximab or obinutuzumab PK samples are required in patients who have received prior treatment with rituximab or obinutuzumab in order to characterize any potential interactions between rituximab or obinutuzumab PK and the clinical effects of mosunetuzumab. These data will be used to understand the relationship of PK exposure to dose and support characterization of dose/exposure-response relationships in the combination setting. In addition, these data will be used to explore and characterize the potential PK interactions between polatuzumab vedotin and mosunetuzumab.

Since mosunetuzumab and polatuzumab vedotin are both B-cell depleting molecules and have demonstrated low immunogenicity rates in their respective Phase I studies, the frequency of ADA sampling times for mosunetuzumab will be reduced in this study compared with Study GO29781. The same ADA schedule of assessments will be used for polatuzumab vedotin.

3.3.17 Rationale for Biomarker Assessments

Predictive and prognostic biomarkers, including biomarkers associated with drug target, mechanism of action, and disease biology, may correlate with outcome in patients with R/R FL and DLBCL and may form the underlying rationale for their assessment in patients treated in this study.

Changes in immune-related biomarkers in blood may provide evidence for biologic activity of the combination of mosunetuzumab and polatuzumab vedotin treatment. An exploratory objective of this study is to assess potential PD biomarkers (including, but not limited to, cytokines, T-cell activation and proliferation, natural killer (NK) cells, B cells, and other exploratory biomarkers) in blood samples. In addition, potential correlations of these biomarkers with the dose, safety, anti-tumor activity, or resistance to the combination therapies of mosunetuzumab and polatuzumab vedotin will be explored.

In addition to peripheral blood sampling, tumor biopsies will be obtained from patients with safely accessible tumors as detailed in Section 4.5.8. Evaluating changes to the tumor immune microenvironment is important in understanding potential mechanisms of mosunetuzumab and polatuzumab vedotin resistance. Therefore, samples may be tested for quantitative and functional changes in the immune cell infiltrate as well as changes to tumor intrinsic factors using both immunohistochemistry (IHC) and gene expression assays. Additionally, if sufficient material is available (DLBCL and MCL cohorts only), tumor tissue may be analyzed for alterations that are associated with drug target, prognostic subtype or outcome including, but not limited to, BCL-2, MYC, CD79B, MYD88, CARD11, and TNFAIP3.

Patients experiencing disease progression or disease relapse after treatment with mosunetuzumab/polatuzumab vedotin may be eligible for re-treatment. Given that loss of CD20 expression after mosunetuzumab treatment is a potential mechanism of resistance to anti-CD20 therapy, potentially similar to antigen-loss escape resistance mechanisms observed with other T-cell bispecific therapies (Topp et al. 2011), a repeat biopsy from a safely accessible site should be obtained which may be used to confirm CD20 expression and assess tumor immune status prior to re-treatment.

In addition to the exploratory objectives listed above, specimens (if consent is given) stored in the Research Biosample Repository (RBR) will also be used for the following:

- To evaluate the association of biomarkers with efficacy and/or adverse events associated with mosunetuzumab and polatuzumab vedotin treatment
- To increase the knowledge and understanding of disease biology and/or to develop biomarker or diagnostic assays and to establish the performance characteristics of these assays

3.3.18 Rationale for Patient-Reported Outcomes

Health-related quality of life (HRQoL) is an important outcome in the care of patients with B-cell malignancies and can be multifaceted. Depending on the specific diagnosis and treatment administered, patients may face limitations on daily functioning (e.g., physical activities, emotional functioning) (Jerkeman et al. 2001). As the goal of measuring HRQoL is to quantify benefit of treatment from the patient perspective, the inclusion of patient-reported outcomes (PROs) will assess the impact of treatment on concepts important to patients. To assess the health status of patients with R/R MCL (Arm K), this will be measured by the EuroQol 5-Dimension, 5-Level (EQ-5D-5L) questionnaire. Additional details about this measure is provided in Section 4.5.10.

4. MATERIALS AND METHODS

4.1 PATIENTS

Approximately up to [redacted] patients will be enrolled in this study (approximately 9-42 patients with R/R DLBCL or FL in the dose-finding phase, and approximately 100 patients with R/R DLBCL, [redacted] patients with R/R FL, [redacted] patients with R/R MCL in the single-arm expansion phase, and [redacted] patients with R/R DLBCL in the randomized Phase II portion).

4.1.1 Inclusion Criteria

Patients must meet the following criteria for study entry:

- Signed Informed Consent Form
- Age ≥18 years at time of signing Informed Consent Form
- Able to comply with the study protocol and procedures in the investigator's judgment
- ECOG PS of 0, 1, or 2
- Life expectancy of at least 12 weeks
- Patients must have histologically confirmed FL, DLBCL or MCL from the following diagnoses by 2016 WHO classification of lymphoid neoplasms, that have either relapsed or have become refractory to a prior regimen as defined below.

  R/R FL (Groups A, B, C and Arm I)

  – Histology

    FL Grade 1, 2, 3a

    In situ follicular neoplasia

    Duodenal-type FL

    Pediatric-type FL

  – Relapsed to prior regimen(s) after having a documented history of response (CR, CR unconfirmed [CRu], or PR) of ≥6 months in duration from completion of regimen(s) or

- Refractory to any prior regimen, defined as no response to the prior therapy, or progression within 6 months of completion of the last dose of therapy
- Must have received at least one prior systemic treatment regimen containing an anti-CD20–directed therapy.

R/R DLBCL (Groups A, B, C Arms J, L and M)

- Histology

  DLBCL, not otherwise specified (NOS) (including germinal center B-cell type and activated B-cell type)

  T-cell/histiocyte-rich large B-cell lymphoma

  High-grade B-cell lymphoma with *MYC* and *BCL-2* and/or *BCL-6* rearrangement

  EBV + DLBCL, NOS

  HHV8 + DLBCL, NOS

  High grade B-cell lymphoma, NOS

  Anaplastic lymphoma kinase (ALK) + large B-cell lymphoma

  FL Grade 3b
- Relapsed to prior regimen(s) after having a documented history of response (CR, CRu, or PR) of ≥ 6 months in duration from completion of regimen(s) or
- Refractory to any prior regimen, defined as no response to the prior therapy, or progression within 6 months of completion of the last dose of therapy
- Must have received at least one prior systemic treatment regimen containing an anti-CD20 directed therapy.
- Transformed FL is an eligible diagnosis for enrollment in the DLBCL cohort but must be R/R to standard therapies for transformed FL.

  Patients with Richter's transformation are not eligible for enrollment into the study.

  The Sponsor may retain the option to limit the number of patients with transformed FL enrolled in the study.
- Grade 3b FL is an eligible diagnosis for enrollment in the DLBCL cohort but must be R/R to standard therapies for aggressive NHL.

  The Sponsor may retain the option to limit the number of patients with Grade 3b FL enrolled in the study.
- High grade B-cell lymphoma with MYC and BCL2 and/or BCL6 rearrangements and *high-grade* B-cell lymphoma NOS are eligible diagnoses for enrollment in the DLBCL cohort but must be R/R to standard therapies for aggressive NHL.

The Sponsor may retain the option to limit the number of patients enrolled in the R/R DLBCL cohort based on the prior systemic treatment.

R/R MCL (Arm K)

- Histology

  MCL

- Relapsed to prior regimen(s) after having a documented history of response (CR, CRu, or PR) of ≥6 months in duration from completion of regimen(s) or
- Refractory to any prior regimen, defined as no response to the prior therapy, or progression within 6 months of completion of the final dose of therapy
- Must have received at least two prior systemic treatment regimens, which include agents from all three classes below:

  Anti-CD20-directed therapy

  BTK inhibitor

  Anthracycline or bendamustine

- Measurable disease, defined as at least one bi-dimensionally measurable nodal lesion, defined as >1.5 cm in its longest dimension, or at least one bi-dimensionally measurable extranodal lesion, defined as >1.0 cm in its longest dimension.
- Pathology report for the initial histopathology diagnosis and the most recent histopathology diagnosis prior to study entry must be provided.
  - Patients with transformed FL must also provide the pathology report at the time of disease transformation.
  - The results of all tests conducted on the tissue at initial diagnosis, including, but not limited to, tests assessing cell of origin, *BCL2* and *MYC* abnormalities, should be provided if done.
  - For patients with MCL, results of all tests conducted on the tissue at initial diagnosis and/or relapse, including, but not limited to, the MCL subtype (nodular and blastoid), Ki-67 proliferation index, and TP53 mutation status, should be provided if done.
- Agreement to provide tumor samples as follows:
  - Agreement to undergo biopsy from a safely accessible site per investigator determination.
  - Patients who are unable to undergo biopsy procedures may be eligible for study enrollment if archival tumor tissue samples (paraffin blocks or at least 20 unstained slides), in place of a fresh biopsy, can be sent to the Sponsor.
  - Bone marrow biopsy and aspirate (if applicable)
- – Adverse events from prior anti-cancer therapy resolved to Grade ≤1
- Laboratory values as follows:
  - Hepatic Function

    AST and ALT ≤2.5×ULN

    Total bilirubin ≤1.5×ULN

Patients with a documented history of Gilbert syndrome and in whom total bilirubin elevations are accompanied by elevated indirect bilirubin are eligible.

- Hematologic Function

  Platelet count $\geq$ 75,000/mm^3 without transfusion within 14 days prior to first dose of study treatment

  ANC $\geq$ 1000/mm^3

  Total hemoglobin $\geq$ 9 g/dL without transfusion within 21 days prior to first dose of study treatment

- Patients with extensive marrow involvement of NHL and/or disease-related cytopenias (e.g., immune thrombocytopenia) may be enrolled if below is met.

  Platelet count $\geq$50,000/mm^3 without transfusion within 14 days

  ANC $\geq$500/mm^3

  Any hemoglobin but without transfusion within 7 days

- INR $\leq$ 1.5 $\times$ ULN in the absence of therapeutic anticoagulation
- PTT or aPTT $\leq$ 1.5 $\times$ ULN in the absence of lupus anticoagulant or therapeutic anticoagulation

• Estimated creatinine CL $\geq$ 50 mL/min by Cockroft-Gault method or other institutional standard methods (e.g., based on nuclear medicine renal scan)

• *Patients who have a negative HIV test at screening.*

  Patients with a positive HIV test at screening are also eligible provided they are stable on anti-retroviral therapy, have a CD4 count $\geq$200/μL, and have an undetectable viral load.

• For women of childbearing potential: agreement to remain abstinent (refrain from heterosexual intercourse) or use contraceptive measures, and agreement to refrain from donating eggs, as defined below:

- Women must remain abstinent or use contraceptive methods with a failure rate of $<$ 1% per year during the treatment period and for 3 months after the final dose of mosunetuzumab, 9 months after the final dose of polatuzumab vedotin, 12 months after the final dose of rituximab, and 3 months after the final dose of tocilizumab, as applicable. Women must refrain from donating eggs during this same period.
- A woman is considered to be of childbearing potential if she is postmenarcheal, has not reached a postmenopausal state ($\geq$ 12 continuous months of amenorrhea with no identified cause other than menopause), and has not undergone surgical sterilization (removal of ovaries and/or uterus).
- Examples of contraceptive methods with a failure rate of $<$ 1% per year include bilateral tubal ligation, male sterilization, established proper use of hormonal contraceptives that inhibit ovulation, hormone-releasing intrauterine devices, and copper intrauterine devices.

– The reliability of sexual abstinence should be evaluated in relation to the duration of the clinical trial and the preferred and usual lifestyle of the patient. Periodic abstinence (e.g., calendar, ovulation, symptothermal, or postovulation methods) and withdrawal are not acceptable methods of contraception.

- For men: agreement to remain abstinent (refrain from heterosexual intercourse) or use a condom, and agreement to refrain from donating sperm, as defined below:
  – With female partners of childbearing potential or pregnant female partners, men must remain abstinent or use a condom during the treatment period and, 6 months after the final dose of polatuzumab vedotin, 3 months after the final dose of rituximab, and 60 days after the final dose of tocilizumab, as applicable, to avoid exposing the embryo. Men must refrain from donating sperm during this same period.
  – The reliability of sexual abstinence should be evaluated in relation to the duration of the clinical trial and the preferred and usual lifestyle of the patient. Periodic abstinence (e.g., calendar, ovulation, symptothermal, or postovulation methods) and withdrawal are not acceptable methods of contraception.

4.1.2 Exclusion Criteria

Patients who meet any of the following criteria will be excluded from study entry:

- Inability to comply with protocol-mandated hospitalization and activity restrictions
- Pregnant or breastfeeding, or intending to become pregnant during the study or within 3 months after the final dose of mosunetuzumab, 9 months after the final dose of polatuzumab vedotin, 12 months after the final dose of rituximab, and 3 months after the final dose of tocilizumab, as applicable.
  – Women of childbearing potential must have a negative serum pregnancy test result within 7 days prior to initiation of study treatment.
- Prior treatment with mosunetuzumab or other CD20-directed bispecific antibodies
- Prior treatment with polatuzumab vedotin
- Current Grade $>$1 peripheral neuropathy
- Prior use of any monoclonal antibod*ies*, radioimmunoconjugate*s* or ADC*s for anti-lymphoma treatment* within 4 weeks before first dose of study treatment
- Treatment with any chemotherapeutic agent, or treatment with any other anti-*lymphoma* agent (investigational or otherwise) within 4 weeks or 5 half-lives of the drug, whichever is shorter, prior to first dose of study treatment
- Treatment with radiotherapy within 2 weeks prior to the first dose of study treatment
  – If patients have received radiotherapy within 4 weeks prior to the first study treatment administration, patients must have at least one measurable lesion outside of the radiation field. Patients who have only one measurable lesion that was previously irradiated but subsequently progressed are eligible.
- Autologous SCT within 100 days prior to first study treatment administration

- Prior treatment with CAR-T therapy within 30 days before first study treatment administration
- Current eligibility for autologous SCT in patients with R/R DLBCL, R/R transformed FL, or R/R Grade 3b FL
- Prior allogeneic SCT
- Prior solid organ transplantation
- Patients with known or suspected history of hemophagocytic lymphohistiocytosis (HLH)
- Patients with history of confirmed progressive multifocal leukoencephalopathy (PML)
- History of severe allergic or anaphylactic reactions to monoclonal antibody therapy (or recombinant antibody-related fusion proteins)
- History of other malignancy that could affect compliance with the protocol or interpretation of results
    - Patients with a history of curatively treated basal or squamous cell carcinoma of the skin or in situ carcinoma of the cervix are allowed.
    - Patients with a malignancy that has been treated with curative intent will also be allowed if the malignancy has been in remission without treatment for ≥ 2 years prior to first study treatment administration.
- Current or past history of CNS lymphoma
- Current or past history of CNS disease, such as stroke, epilepsy, CNS vasculitis, or neurodegenerative disease
    - Patients with a history of stroke who have not experienced a stroke or transient ischemic attack in the past 2 years and have no residual neurologic deficits as judged by the investigator are allowed.
    - Patients with a history of epilepsy who have had no seizures in the past 2 years while not receiving any anti-epileptic medications are allowed in the expansion cohorts only.
- Significant cardiovascular disease such as New York Heart Association Class III or IV cardiac disease, myocardial infarction within the last 6 months, unstable arrhythmias, or unstable angina
- Significant active pulmonary disease (e.g., bronchospasm and/or obstructive pulmonary disease)
- Known active bacterial, viral, fungal, mycobacterial, parasitic, or other infection (excluding fungal infections of nail beds) at study enrollment, or any major episode of infection requiring treatment with IV antibiotics or hospitalization (relating to the completion of the course of antibiotics) within 4 weeks prior to first study treatment administration
- Known or suspected chronic active EBV infection
- Recent major surgery within 4 weeks prior to first study treatment administration

- Protocol-mandated procedures (e.g., tumor biopsies and bone marrow biopsies) are permitted.

- Positive test results for chronic hepatitis B infection (defined as positive hepatitis B surface antigen [HBsAg] serology)
  - Patients with occult or prior hepatitis B infection (defined as positive total hepatitis B core antibody and negative HBsAg) may be included if HBV DNA is undetectable at the time of screening. These patients must be willing to undergo monthly DNA testing and appropriate antiviral therapy as indicated.
- Acute or chronic HCV infection
  - Patients who are positive for HCV antibody must be negative for HCV by PCR to be eligible for study participation.
- Administration of a live, attenuated vaccine within 4 weeks before first dose of study treatment administration or anticipation that such a live, attenuated vaccine will be required during the study
  - Patients must not receive live, attenuated vaccines (e.g., FluMist®) while receiving study treatment and after the last dose until B-cell recovery to the normal ranges. Killed vaccines or toxoids should be given at least 4 weeks prior to the first dose of study treatment to allow development of sufficient immunity.
  - Inactivated influenza vaccination should be given during local influenza season only.
  - Investigators should review the vaccination status of potential study patients being considered for this study and follow the U.S. Centers for Disease Control and Prevention guidelines for adult vaccination with any other non-live vaccines intended to prevent infectious diseases prior to study.
- History of autoimmune disease, including, but not limited to, myasthenia gravis, myositis, autoimmune hepatitis, systemic lupus erythematosus, rheumatoid arthritis, inflammatory bowel disease, vascular thrombosis associated with antiphospholipid syndrome, Wegener granulomatosis, Sjögren syndrome, Guillain-Barré syndrome, multiple sclerosis, vasculitis, or glomerulonephritis
  - Patients with a history of autoimmune-related hypothyroidism on a stable dose of thyroid replacement hormone may be eligible.
  - Patients with controlled Type 1 diabetes mellitus who are on an insulin regimen are eligible for the study.
  - Patients with a history of disease-related immune thrombocytopenic purpura, autoimmune hemolytic anemia, or other stable autoimmune diseases may be eligible.
- Received systemic immunosuppressive medications (including, but not limited to, cyclophosphamide, azathioprine, methotrexate, thalidomide, and anti-tumor necrosis factor agents) with the exception of corticosteroid treatment ≤10 mg/day prednisone or equivalent within 2 weeks prior to first dose of study treatment

– The use of inhaled corticosteroids is permitted.
– The use of mineralocorticoids for management of orthostatic hypotension is permitted.
– The use of physiologic doses of corticosteroids for management of adrenal insufficiency is permitted.
– *Patients who received acute, low-dose, systemic immunosuppressant medications (e.g., single dose of dexamethasone for nausea or B symptoms) may be enrolled.*

- Clinically significant history of liver disease, including viral or other hepatitis, current alcohol abuse, or cirrhosis
- Any serious medical condition or abnormality in clinical laboratory tests that, in the investigator'sjudgment, precludes the patient's safe participation in and completion of the study, or which could affect compliance with the protocol or interpretation of results

4.2 METHOD OF TREATMENT ASSIGNMENT

This is an open-label study. In the Phase Ib dose-finding portion of the study, the Sponsor will allocate patients based on cohort availability and safety and DLT data, with input from the IMC if necessary. In the Phase II single-arm expansion portion of the study, patients will be allocated to either the R/R DLBCL, R/R FL, or R/R MCL arms based on their confirmed NHL subtype. In the randomized Phase II portion for R/R DLBCL, patients will be allocated to each of the treatment arms through the use of a stratified permuted-block randomization to ensure within-stratum balance of patient characteristics between treatment arms. Patients will be randomized to Arms L and M in a ratio of 1:1, stratified by the number of prior treatment regimens (one prior line of therapy vs. ≥2 prior lines of therapy). Randomization will be performed by an interactive voice or web-based response system (IxRS) using stratified permuted blocks for the Phase II randomized expansion cohort.

After written informed consent has been obtained and preliminary eligibility has been established, the study site will submit documentation supporting eligibility to the Sponsor if applicable. The Sponsor will provide the dose group assignment and the patient number will be assigned by the IxRS.

4.3 STUDY TREATMENT AND OTHER TREATMENTS RELEVANT TO THE STUDY DESIGN

The investigational medicinal products (IMPs) for this study are mosunetuzumab, polatuzumab vedotin, rituximab, and tocilizumab.

4.3.1 Study Treatment Formulation, Packaging, and Handling

4.3.1.1 Mosunetuzumab

Mosunetuzumab will be supplied by the Sponsor. For information on the formulation, packaging and handling of mosunetuzumab, refer to the pharmacy manual and the Mosunetuzumab Investigator's Brochure.

4.3.1.2 Polatuzumab Vedotin

Polatuzumab vedotin will be supplied by the Sponsor. For information on the formulation, packaging, and handling of polatuzumab vedotin, see the pharmacy manual and the Polatuzumab Vedotin Investigator's Brochure.

4.3.1.3 Rituximab

Rituximab will be supplied by the Sponsor. For information on the formulation, packaging, and handling of rituximab, see the pharmacy manual and the Rituximab Investigator Brochure.

4.3.1.4 Tocilizumab

Tocilizumab will be supplied by the Sponsor *as an IMP*. *Due to the need to manage CRS urgently and potential accessibility limitations at the site, commercial tocilizumab can be obtained locally by the study site for emergency use purposes. It will be prepared, handled, and managed according to standard institutional practices.*

*All tocilizumab used in the study will be tracked and accounted for as required by ICH GCP. Tocilizumab supplied by the Sponsor will include a clinical study drug/IMP label. Commercial tocilizumab obtained locally by the study site will have the marketed product label. Refer to the local prescribing information for further instructions regarding recommended storage conditions and packaging configuration, as well as the P*harmacy *M*anual and the Tocilizumab Investigator's Brochure.

4.3.2 Study Treatment Dosage, Administration, and Compliance

Information on single-agent mosunetuzumab, polatuzumab vedotin, rituximab, and tocilizumab are presented in this section. The use of these agents as part of a multi-drug regimen is described in Section 4.3.3.

Any dose modification should be noted on the Study Drug Administration electronic Case Report Form (eCRF). Cases of accidental overdose or medication error, along with any associated adverse events, should be reported as described in Section 5.4.4.

Guidelines for dosage modification and treatment interruption or discontinuation for patients who experience adverse events are provided in Section 5.1.7.

4.3.2.1 Mosunetuzumab

Mosunetuzumab will be administered either by IV infusion (Groups A, B, C; Arms I and J) or SC injection (Arms K and L) using standard medical syringes, syringe pumps or IV bags where applicable, in combination with polatuzumab vedotin (Section 4.3.3). Flat dosing independent of body weight will be used for mosunetuzumab. The dose of mosunetuzumab for each patient will depend on their dose level assignment as detailed in Sections 3.1.2 and 4.3.3.

Compatibility testing has shown that mosunetuzumab is stable in infusion sets and IV bags. When administered intravenously, the drug product will be delivered by syringe pump via an IV infusion set or an IV bag with a final mosunetuzumab volume determined by the dose, and an in-line filter should not be used. When administered subcutaneously, mosunetuzumab will be delivered by medical syringe with a final mosunetuzumab volume not to exceed 2.0 mL.

Mosunetuzumab will be administered in a setting with immediate access to trained critical care personnel and facilities equipped to respond to and manage medical emergencies. Hospitalization requirements for patients receiving study treatment are described in Section 5.1.1.

Mosunetuzumab will be administered to well-hydrated patients.

For patients receiving mosunetuzumab IV (Groups A, B, C and Arms I, J), corticosteroid premedication consisting of dexamethasone 20 mg IV or methylprednisolone 80 mg IV should be administered 1 hour prior to the administration of each mosunetuzumab dose. This administration of corticosteroid premedication may be optional for C3 and beyond for patients in Group A and Group C (and Arms I and J if following Group A or C dosing schedule), or C4 and beyond for patients in Group B (and Arms I and J if following Group B dosing schedule) based on investigator's assessment. However, if the patient experiences CRS, premedication with steroids must be administered for the subsequent doses until no additional CRS events are observed.

For patients receiving mosunetuzumab SC (Arms K and L), corticosteroid premedication consisting of 20 mg of dexamethasone *(preferred)* or 80 mg of methylprednisolone*, either IV or orally, should* be administered prior to the administration of each mosunetuzumab dose. In addition, premedication with oral acetaminophen or paracetamol (e.g., 500–1000 mg) and/or 50–100 mg diphenhydramine may be administered per standard institutional practice prior to administration of mosunetuzumab. The administration of corticosteroid premedication is optional for C2 and beyond in patients who received mosunetuzumab SC and did not experience CRS in the previous cycle.

For mosunetuzumab administration, the recommended management of IRRs, which may be indistinguishable from CRS, is detailed in Table 18.

For mosunetuzumab IV administration, mosunetuzumab will initially be infused over 4 hours (±15 minutes). The infusion may be slowed or interrupted for patients experiencing infusion-associated symptoms (see Section 5.1.2.1).

In the absence of infusion-related adverse events, the infusion time of mosunetuzumab in C2 (Group A) or C3 (Groups B and C) and beyond may be reduced to 2 hours (±15 minutes).

For SC administration (Arms K and L), mosunetuzumab will be administered by qualified staff over 30 seconds to 2 minutes. Refer to the pharmacy manual for more details including syringe size and preferred injection site. The recommended management of injection-site reactions is detailed in Section 5.1.7.7. All patients must have IV access in place prior to mosunetuzumab SC administration for at least all doses in C1. Placement of IV access may be optional in C2 and beyond but should be considered for patients who continue to experience, or remain at risk, for CRS.

Following each mosunetuzumab *IV* dose, patients will be observed at least 90 minutes for fever, chills, rigors, hypotension, nausea, or other signs and symptoms of CRS. *For each mosunetuzumab SC dose, patients will be observed for at least 30 minutes during Cycles 1 and 2, and for at least 15 minutes for Cycle 3 and beyond.* Vital signs should be monitored according to the Schedule of Activities (Appendix 1).

Guidelines for mosunetuzumab schedule modification and treatment interruption or discontinuation are provided in Section 5.1.7.

Any overdose or incorrect administration of mosunetuzumab should be noted on the Study Drug Administration eCRF. Adverse events associated with an overdose or incorrect administration of study drug should be recorded on the Adverse Event eCRF.

4.3.2.2 Polatuzumab Vedotin

Polatuzumab vedotin will be administered by IV infusion in combination with mosunetuzumab *(Sections 4.3.3.1 and 4.3.3.2) or rituximab (Section 4.3.3.3)*

The dose of polatuzumab vedotin for each patient will be 1.8 mg/kg. The total dose of polatuzumab vedotin for each patient will depend on the patient's weight on C1D1 (or within 96 hours before C1D1). If the patient's weight within 96 hours prior to D1 of a given treatment cycle increases or decreases > 10% from the weight obtained for C1D1, the new weight should be used to calculate the dose. The weight that triggered a dose adjustment will be taken as the new reference weight for future dose adjustments. All subsequent doses should be modified accordingly.

After reconstitution with Sterile Water for Injection and dilution into IV bags that contain isotonic sodium chloride solution (0.9% NaCl), polatuzumab vedotin will be administered by IV infusion using a dedicated standard administration set with 0.2-μm or 0.22-μm

in-line filters at a final polatuzumab vedotin concentration determined by the patient-specific dose. Compatibility of polatuzumab vedotin with IV bags, infusion lines, filters, and other infusion aids has been established with items made of specific materials of construction. Consult the pharmacy manual and the Polatuzumab Vedotin Investigator's Brochure for a list of compatible materials and specific dose preparation instructions.

The initial dose will be administered over 90 (±10) minutes to patients who are well hydrated. Premedication (e.g., 500-1000 mg of oral acetaminophen or paracetamol and 50–100 mg diphenhydramine as per institutional standard practice) may be administered to an individual patient before administration of polatuzumab vedotin. Administration of corticosteroids is permitted at the discretion of the treating physician. If IRRs are observed with the first infusion in the absence of premedication, premedication must be administered before subsequent doses.

The polatuzumab vedotin infusion may be slowed or interrupted for patients experiencing infusion-associated symptoms. Following the initial dose, patients will be observed for 90 minutes for fever, chills, rigors, hypotension, nausea, or other infusion associated symptoms. If prior infusions have been well tolerated, subsequent doses of polatuzumab vedotin may be administered over 30 (±10) minutes, followed by a 30 minute observation period after the infusion. The time interval between the end of infusion of polatuzumab vedotin and the start of mosunetuzumab infusion should be at least 60 minutes.

Guidelines for dosage modification and treatment interruption or discontinuation are provided in Section 5.1.7.

Any overdose or incorrect administration of study drug should be noted on the Polatuzumab Vedotin Administration eCRF. Adverse events associated with an overdose or the incorrect administration of polatuzumab vedotin should be recorded on the Adverse Event eCRF.

4.3.2.3 Rituximab

Rituximab will be administered by IV infusion in combination with polatuzumab vedotin (Arm M, Section 4.3.3.3). Rituximab 375 mg/m^2 will be administered by IV infusion. No dose modifications of rituximab are allowed. The patient's body surface area (BSA) calculated at screening (within 96 hours before C1D1) should be used to calculate the dose of rituximab throughout the study unless the patient's weight increases or decreases by >10% from screening, in which case BSA should be recalculated and used for subsequent dosing. In obese patients, there is no BSA cap and actual body weight, not adjusted weight, is recommended. Empiric dose adjustment for obese patients (obesity defined as body mass index ≥30, as measured in kilograms divided by meters squared) may be implemented per institutional guidelines.

The rituximab administration should be completed at least 30 minutes before administration of polatuzumab vedotin. The infusion of rituximab may be split over 2 days if the patient is at increased risk for an IRR (high tumor burden, high peripheral lymphocyte count). Administration of rituximab may be continued on the following day, if needed, for patients who experience an adverse event during the rituximab infusion. If a dose of rituximab is split over 2 days, both infusions must occur with appropriate premedication and at the first infusion rate (see Table 9).

All rituximab infusions should be administered to patients after premedication with oral acetaminophen (e.g., 650–1000 mg) and an antihistamine such as diphenhydramine hydrochloride (50–100 mg) 30–60 minutes before starting each infusion (unless contraindicated). An additional glucocorticoid (e.g., 100 mg IV prednisone or prednisolone or equivalent) is allowed at the investigator's discretion. For patients who did not experience infusion-related symptoms with their previous infusion, premedication at subsequent infusions may be omitted at the investigator's discretion.

Rituximab infusions will be administered according to the instructions in Table 9. If a patient tolerates the first cycle of study treatment without significant IRRs, rituximab may be administered as rapid infusion in accordance with local institutional guidelines. During the treatment period, rituximab must be administered to patients in a setting where full emergency resuscitation facilities are immediately available. Patients should be under close supervision of the investigator at all times. For the management of IRRs and anaphylaxis, see Section 5.1.7.3.

Rituximab should be administered as a slow IV infusion through a dedicated line. IV infusion pumps (such as the Braun Infusomat Space) should be used to control the infusion rate of rituximab. Administration sets with polyvinyl chloride (PVC), polyurethane (PUR), or polyethylene (PE) as a product contact surface and IV bags with polyolefin, polypropylene (PP), PVC, or PE as a product contact surface are compatible and can be used. Additional in-line filters should not be used because of potential adsorption. The in-line filter used for the administration of polatuzumab vedotin should not be used for the administration of rituximab.

After the end of the first infusion, the IV line or central venous catheter should remain in place for ≥2 hours in order to administer IV drugs if necessary. If no adverse events occur after 2 hours, the IV line may be removed or the central venous catheter may be de-accessed. For subsequent infusions, the IV line or central venous catheter should remain in place for at least 1 hour after the end of the infusion. If no adverse events occur after 1 hour, the IV line may be removed or the central venous catheter may be de-accessed.

Table 9 Administration of First and Subsequent Infusions of Rituximab

First Infusion (Cycle 1 Day 1)	Subsequent Infusions
Begin infusion at an initial rate of 50 mg/hr.<br>If no infusion-related or hypersensitivity reaction occurs, increase the infusion rate in 50-mg/hr increments every 30 minutes, to a maximum of 400 mg/hr.<br>If a reaction develops, stop or slow the infusion. Administer medications and supportive care in accordance with institutional guidelines. If the reaction resolves, resume the infusion at a 50% reduction in rate (i.e., 50% of rate used at the time the reaction occurred).	If the patient experienced an infusion-related or hypersensitivity reaction during the prior infusion, begin infusion at an initial rate of 50 mg/hr and follow instructions for first infusion.<br>If the patient tolerated the prior infusion well (defined by absence of Grade 2 reactions during a final infusion rate of ≥ 100 mg/hr), begin infusion at a rate of 100 mg/hr.<br>If no reaction occurs, increase the infusion rate in 100-mg/hr increments every 30 minutes, to a maximum of 400 mg/hr.<br>If a reaction develops, stop or slow the infusion. Administer medications and supportive care in accordance with institutional guidelines. If the reaction resolves, resume the infusion at a 50% reduction in rate (i.e., 50% of rate used at the time the reaction occurred).

Guidelines for treatment interruption or discontinuation are provided in Section 5.1.8.

Any overdose or incorrect administration of rituximab should be noted on the Rituximab Administration eCRF. Adverse events associated with an overdose or incorrect administration of rituximab should be recorded on the Adverse Event eCRF.

4.3.2.4 Tocilizumab

Tocilizumab will not be administered to all patients but only to those patients who experience a CRS event for which tocilizumab is indicated (a rescue IMP) as described in Section 5.1.7.5 and Table 18. Tocilizumab will be supplied by the Sponsor *as an IMP*. For information on the formulation, packaging and handling of tocilizumab, refer to the pharmacy manual and the Tocilizumab Investigator's Brochure. Tocilizumab may be obtained locally by the study sites for emergency purposes, if permitted by local regulations, and will be prepared and handled accordingly to standard practice. Refer to the local prescribing information for further instructions regarding recommended storage conditions and packaging configurations. Note: If tocilizumab is administered, refer to Appendix 6 for the schedule of activities for tocilizumab treatment of CRS.

Any overdose or incorrect administration of tocilizumab should be noted on the Study Drug Administration eCRF. Adverse events associated with an overdose or incorrect administration of study drug should be recorded on the Adverse Event eCRF.

4.3.3 Study Treatment Regimens

4.3.3.1 Mosunetuzumab IV plus Polatuzumab Vedotin (Groups A, B, and C; Arms I, and J)

The dose level of polatuzumab vedotin is 1.8 mg/kg administered by IV infusion on the days outlined in the schedules for Groups A, B, and C. For Groups A, B, and C the schedule and dose level of mosunetuzumab will be as described in Section 3.1.2. For Arms I and J, the schedule and dose level of mosunetuzumab will follow the RP2D and schedule selected from Groups A, B, *or* C, and similarly the schedule for polatuzumab vedotin will follow the schedule selected from Groups A, B, or C (see Table 10, Table 11, and Table 12, respectively).

On days when mosunetuzumab is given in combination with polatuzumab vedotin, polatuzumab vedotin will be administered first, followed by mosunetuzumab. Polatuzumab vedotin should be administered as described in Section 4.3.2.2, and mosunetuzumab should be administered as described in Section 4.3.2.1. The time interval between the end of infusion of polatuzumab vedotin and the start of mosunetuzumab infusion/injection should be at least 90 minutes for Cycles 1 and 2, which corresponds to the observation period described in Section 4.3.2.2. For C3 and beyond, if prior polatuzumab vedotin infusions have been well tolerated, the time interval between the end of infusion of polatuzumab vedotin and the start of mosunetuzumab infusion should be at least 60 minutes.

Patients will receive a total of 6 cycles of polatuzumab vedotin. Patients will receive a total of 8 cycles of mosunetuzumab IV in Groups A and C. Because of the delayed start of mosunetuzumab in C1 in Group B, there are 9 cycles of mosunetuzumab IV, but patients in Group B will receive the same total dose of mosunetuzumab as in Groups A and C. Patients in Groups A, B, and C, and Arms I and J may be treated with mosunetuzumab beyond 8 cycles for a total of 17 cycles according to Section 3.1.5. A cycle is 21 days.

Guidelines for dosage modification and treatment interruption or discontinuation are provided in Section 5.1.7.

Table 10 Mosunetuzumab Plus Polatuzumab Vedotin Regimen (Group A)

Agent	Dose	Route	Cycle 1			Cycles 2–6	Cycles 7–8	Cycles 9–17 [a]
			Day 1	Day 8	Day 15	Day 1	Day 1	Day 1
Polatuzumab vedotin	1.8 mg/kg	IV	x	–	–	x	–	–
	DL_1	IV	x	–	–	–	–	–
Mosunetuzumab	DL_2	IV	–	x	–	–	–	–
	DL_3	IV	–	–	x	x	x	x

DL = dose level.

[a] For mosunetuzumab treatment duration beyond Cycle 8, see Section 3.1.5.

Table 11 Mosunetuzumab Plus Polatuzumab Vedotin Regimen (Group B)

Agent	Dose	Route	Cycle 1			Cycles 2–6	Cycles 7–9	Cycles 10–17 [a]
			Day 1	Day 8	Day 15	Day 1	Day 1	Day 1
Polatuzumab vedotin	1.8 mg/kg	IV	x	–	–	x	–	–
	DL_1	IV	–	x	–	–	–	–
Mosunetuzumab	DL_2	IV	–	–	x	–	–	–
	DL_3	IV	–	–	–	x	x	x

DL = dose level.

[a] For mosunetuzumab treatment duration beyond Cycle 9, see Section 3.1.5.

Table 12 Mosunetuzumab Plus Polatuzumab Vedotin Regimen (Group C)

Agent	Dose	Route	Cycle 1			Cycles 2–7	Cycle 8	Cycles 9–17 [a]
			Day 1	Day 8	Day 15	Day 1	Day 1	Day 1
Polatuzumab vedotin	1.8 mg/kg	IV	–	–	–	x	–	–
	DL_1	IV	x	–	–	–	–	–
Mosunetuzumab	DL_2	IV	–	x	–	–	–	–
	DL_3	IV	–	–	x	x	x	x

DL = dose level.

[a] For mosunetuzumab treatment duration beyond Cycle 8, see Section 3.1.5.

4.3.3.2 Mosunetuzumab SC plus Polatuzumab Vedotin (Arm K and L)

For Arm K and L, the schedule of mosunetuzumab SC in combination with polatuzumab vedotin will follow the below dose and schedule (Table 13). A cycle is 21 days.

The treatment in Arm K is for a total of 17 cycles (Section 3.1.5). The treatment in Arm L is for a total of 8 cycles only, regardless of the response status at PRA.

Table 13 Mosunetuzumab SC Plus Polatuzumab Vedotin Regimen (Arm K and L)

			Cycle 1			Cycles 2–6	Cycles 7–8	Cycles 9–17 (Arm K only)
Agent	Dose	Route	Day 1	Day 8	Day 15	Day 1	Day 1	Day 1
Polatuzumab vedotin	1.8 mg/kg	IV	x	–	–	x	–	–
Mosunetuzumab	DL_1	SC	x	–	–	–	–	–
	DL_2	SC	–	x	–	–	–	–
	DL_3	SC	–	–	x	x	x	x

DL =dose level; *IMC=Internal Monitoring Committee*.

Note: The starting dose based on the optimal dose determined in Study GO29781 is the following: DL_1 =5mg, DL_2 =45mg, and DL_3 =45mg.

Upon IMC review of pre-specified number of patients in each cohort, the dose may be reduced.

4.3.3.3 Rituximab plus Polatuzumab Vedotin (Arm M)

Patients will receive a total of 6 cycles of polatuzumab vedotin. Patients will receive a total of 8 cycles of rituximab. A cycle is 21 days.

Treatments will be administered sequentially in the order specified below.

- Schedule for C1–6, D1
  - Rituximab 375 mg/m^2 IV infusion according to instructions in Section 4.3.2.3, Table 14, followed by at least 30 minutes' observation
  - Polatuzumab vedotin 1.8 mg/kg IV infusion over 90 ($\pm$10) minutes as described in Section 4.3.2.2, followed by 90 minutes' observation for infusion-associated symptoms
- Schedule for C7–8, D1
  - Rituximab 375 mg/m^2 IV infusion according to instructions in Section 4.3.2.3, Table 14, followed by at least 30 minutes' observation

Table 14 Rituximab Plus Polatuzumab Vedotin Regimen (Arm M)

			Cycles 1–6	Cycles 7–8
Agent	Dose	Route	Day 1	Day 1
Rituximab	375 mg/m^2	IV	x	x
Polatuzumab vedotin	1.8 mg/kg	IV	x	–

4.3.3.4 Cross Over Treatment to Mosunetuzumab plus Polatuzumab Vedotin (Arm M-crossover)

If a patient in Arm M experiences a disease progression before or at the end of therapy, or has stable disease at the end of therapy, the patient will have an option to receive the immediate next treatment with mosunetuzumab plus polatuzumab vedotin (Arm M-crossover, see Section 3.1.5.2). Arm M-crossover treatment will be identical to the treatment for Arm L. For detailed procedure of crossover and treatment schedule, see Section 3.1.5.2).

4.3.4 Investigational Medicinal Product Accountability

All IMPs required for completion of this study (mosunetuzumab, polatuzumab vedotin, rituximab, and tocilizumab) will be provided by the Sponsor where required by local health authority regulations. The study site will acknowledge receipt of IMPs supplied by the Sponsor, using the IxRS, to confirm the shipment condition and content. Any damaged shipments will be replaced.

IMPs will either be disposed of at the study site according to the study site's institutional standard operating procedure or be returned to the Sponsor (if supplied by the Sponsor) with the appropriate documentation. The site's method of destroying Sponsor-supplied IMPs must be agreed to by the Sponsor. The site must obtain written authorization from the Sponsor before any Sponsor-supplied IMP is destroyed, and IMP destruction must be documented on the appropriate form.

Accurate records of all IMPs received at, dispensed from, returned to, and disposed of by the study site should be recorded on the Drug Inventory Log.

4.3.5 Continued Access to Mosunetuzumab, Polatuzumab Vedotin, Rituximab and Tocilizumab

The Sponsor will offer continued access to Roche IMPs (mosunetuzumab, polatuzumab vedotin, rituximab, and tocilizumab) free of charge to eligible patients in accordance with the Roche Global Policy on Continued Access to Investigational Medicinal Product, as outlined below.

A patient will be eligible to receive Roche IMPs (mosunetuzumab, polatuzumab vedotin, rituximab, and tocilizumab) after completing the study if all of the following conditions are met:

- The patient has a life-threatening or severe medical condition and requires continued Roche IMP treatment for his or her well-being
- There are no appropriate alternative treatments available to the patient
- The patient and his or her doctor comply with and satisfy any legal or regulatory requirements that apply to them

A patient will not be eligible to receive Roche IMPs (mosunetuzumab, polatuzumab vedotin, rituximab, and tocilizumab) after completing the study if any of the following conditions are met:

- The Roche IMP is commercially marketed in the patient's country and is reasonably accessible to the patient (e.g., is covered by the patient's insurance or wouldn't otherwise create a financial hardship for the patient)
- The Sponsor has discontinued development of the IMP or data suggest that the IMP is not effective for B-cell non-Hodgkin lymphomas
- The Sponsor has reasonable safety concerns regarding the IMP as treatment for B-cell non-Hodgkin lymphomas
- Provision of the Roche IMP is not permitted under the laws and regulations of the patient's country

The Roche Global Policy on Continued Access to Investigational Medicinal Product is available at the following web site:

http://www.roche.com/policy_continued_access_to_investigational_medicines.pdf

4.4 CONCOMITANT THERAPY

Concomitant therapy consists of any medication (e.g., prescription drugs, over-the-counter drugs, vaccines, herbal or homeopathic remedies, nutritional supplements) used by a patient in addition to protocol-mandated treatment from 7 days prior to initiation of study drug until 90 days after the last dose of study treatment or start of new anti-lymphoma therapy, whichever is earlier. After this period, concomitant medications should be collected if they were used to manage serious adverse events that are believed to be related to study treatment. All such medications should be reported to the investigator and recorded on the Concomitant Medications eCRF.

4.4.1 Permitted Therapy

In general, investigators should manage a patient’s care with supportive therapies as clinically indicated, per local standard practice.

Patients are permitted to use oral contraceptives and hormone-replacement therapy during the study.

Intrathecal chemotherapy for CNS prophylaxis is permitted; however, systemic chemotherapy for CNS prophylaxis is prohibited. CNS prophylaxis using high-dose IV methotrexate (e.g., 1 g/m^2 per cycle) is not permitted.

Prophylaxis prior to each study treatment, if applicable, is described in Section 4.3.2.

Patients who experience infusion-related symptoms may be treated symptomatically as described in Section 5.1.7.4.

Treatment of severe CRS or HLH according to published recommendations and/or institutional practice is permitted. Refer to Sections 5.1.2.1 and 5.1.2.2.

4.4.1.1 Treatment and Prophylaxis of Neutropenia

Prophylactic and therapeutic use of G-CSF (*e.g.,* filgrastim, pegfilgrastim) is allowed (see Appendix 14) in accordance with instructions provided in the package inserts, institutional practice, and/or published guidelines (Smith et al. 2015b). Growth factor support should be started when ANC is $<500/mm^3$, unless medically contraindicated.

After reviewing available data, the IMC may determine that G-CSF is required as primary prophylaxis in each cycle of therapy for patients receiving mosunetuzumab plus polatuzumab vedotin. If necessary, the IMC will communicate a recommendation regarding dose and form of G-CSF for primary prophylaxis to the study sites.

4.4.1.2 Prophylaxis for Tumor Lysis Syndrome

Tumor lysis syndrome is a known pharmacodynamic effect of anti-tumor therapy in hematologic malignancies including NHL. All patients should be well hydrated prior to the first dose of study treatment. Based on their risk for developing TLS, patients enrolled in a group or arm with mosunetuzumab will receive TLS prophylaxis with allopurinol or a suitable alternative treatment prior to the initiation of treatment (see Section 5.1.2.2). If deemed appropriate by the investigator, patients should continue to receive repeated prophylaxis and adequate hydration prior to each subsequent cycle of treatment.

4.4.1.3 Prophylaxis for Infections

Anti-infective prophylaxis for viral, fungal, bacterial or pneumocystis infections is permitted and should be instituted per institutional practice.

4.4.1.4 Pre-Planned Radiotherapy

Pre-planned radiotherapy (i.e., radiation that was planned before the first day of study treatment) to be given at the end of study treatment) may be administered to initial sites of bulky or extranodal disease according to institutional practice. If indicated, pre-planned radiotherapy should be administered within 8 weeks after the last study drug treatment and should start after the PRA with PET-CT scans are completed. Any radiotherapy should be pre-planned by the center and documented prior to the first day of study treatment and then entered in the eCRF once the patient is dosed for the first day of study treatment. All unplanned radiotherapy administered to patients will be considered as a new anti-lymphoma treatment (NALT).

4.4.1.5 COVID-19 Vaccinations

Concomitant administration of an approved non-live COVID-19 vaccine is permitted. Examples of permitted vaccines include mRNA, inactivated virus, and replication-deficient viral vector vaccines. The decision whether and when to administer

a *COVID-19* vaccine should be individualized by the investigator in consultation with the patient.

Factors to consider when making the individualized decision for patients receiving mosunetuzumab include the following:

- Risk of SARS-CoV-2 infection and potential benefit from the vaccine
- General condition of the patient and potential complications associated with SARS-CoV-2 infection
- Severity and seriousness of the underlying disease
- Epidemiology of COVID-19 in the patient's location

Prior to starting study treatment, *COVID-19* vaccines and other permitted vaccines should ideally be administered to patients before the start of immunosuppressive therapy, with the aim to complete the vaccination course at least one week prior to starting study treatment, unless a delay is clinically unacceptable.

If a *COVID-19* vaccine is administered while the patient is already receiving treatment *with mosunetuzumab, the COVID vaccine should be administered in the middle of a treatment cycle, for example one week before or after a dose of mosunetuzumab. The* administration of the vaccine should be timed to take place after completion of mosunetuzumab step-up dosing and at least one week after administration of the target dose.

CRS is a risk for mosunetuzumab that occurs most commonly during step-up dosing. Many *COVID-19* vaccines are highly immunogenic, and their risk of potentiating CRS is unknown.

4.4.1.6 Other Concomitant Medications

Necessary supportive measures for optimal medical care will be given throughout the study according to institutional standards.

Anti-emetic therapy may be instituted for any patient if clinically indicated according to institutional practice.

4.4.2 Cautionary Therapy

4.4.2.1 Medications Given with Precaution Due to Effects Related to Cytochrome P450 Enzymes

Mosunetuzumab

Given the expected pharmacology of mosunetuzumab, the transient release of cytokines (most resolved within the first 24 hours of the C1D1 dose) may suppress CYP450 enzymes and cause drug-drug interactions. Preliminary clinical data indicate that mosunetuzumab induced a transient elevation in plasma IL-6, with peak levels occurring in the majority of patients within 4–6 hours of the C1D1 dose, and returning to baseline by 24 hours. Patients may be of highest risk of a drug-drug interaction are

those receiving concomitant medications that are CYP450 substrates and have a narrow therapeutic index (Appendix 9). Such concomitant medications should be monitored for toxicity, and dose adjusted accordingly.

Polatuzumab Vedotin

In vitro data suggest that unconjugated MMAE is mainly metabolized by CYP3A4 and, to a lesser extent, by CYP2D6. Based on a validated physiological-based PK model simulation (Chen et al. 2015), strong CYP3A4 inhibitors may increase the exposure (e.g., AUC) of unconjugated MMAE by approximately 50%, while acMMAE pharmacokinetics is not affected. Concomitant medications that are strong CYP3A4 inhibitors (Appendix 9) should be considered cautionary as they may potentially lead to adverse reactions, which require close monitoring.

If a patient is taking any of the medications in the categories of strong CYP3A4 inhibitors and inducers, the investigator will assess and document the use of these medications known or suspected to fall in those categories.

A sample list of cautionary medications that fall into the categories within this section can be found in Appendix 9. The lists of medications are not necessarily comprehensive. The investigator should consult the prescribing information when determining whether a concomitant medication can be safely administered with study treatment.

Tocilizumab

CYP450 enzymes in the liver are down-regulated by infection and inflammatory stimuli, including cytokines such as IL-6. Inhibition of IL-6 signaling in patients with rheumatoid arthritis who are treated with tocilizumab may restore CYP450 activities to higher levels than those patients not treated with tocilizumab, leading to increased metabolism of drugs that are CYP450 substrates. In vitro studies showed that tocilizumab has the potential to affect expression of multiple CYP enzymes, including CYP1A2, CY2B6, CYP2C9, CYP2C19, CYP2D6, and CYP3A4. The effects of tocilizumab on CYP2C8 or transporters are unknown. In vivo studies with omeprazole (metabolized by CYP2C19 and CYP3A4) and simvastatin (metabolized by CYP3A4) showed up to a 28% and 57% decrease in exposure 1 week following a single dose of tocilizumab, respectively.

The effect of tocilizumab on CYP enzymes may be clinically relevant for CYP450 substrates with narrow therapeutic index (see Appendix 9), where the dose is individually adjusted:

- Upon initiation or discontinuation of tocilizumab in patients being treated with these types of medicinal products, therapeutic monitoring of effect (e.g., warfarin) or drug concentration (e.g., cyclosporine or theophylline) should be performed and the individual dose of the medicinal product adjusted as needed.
- Prescribers should exercise caution when tocilizumab is coadministered with CYP3A4 substrate drugs where a decrease in effectiveness is undesirable (e.g., oral contraceptives, lovastatin, atorvastatin).

- The effect of tocilizumab on CYP450 enzyme activity may persist for several weeks after stopping therapy.

4.4.2.2 Herbal Therapies

Concomitant use of herbal therapies is not recommended because their pharmacokinetics, safety profiles, and potential drug-drug interactions are generally unknown. However, herbal therapies not intended for the treatment of cancer may be used during the study at the discretion of the investigator.

4.4.3 Prohibited Therapy

Use of the following therapies is prohibited during the study:

- Administration of live vaccines (see also Section 4.4.4.1)
- Cytotoxic chemotherapy other than study treatments intended for treatment of lymphoma
- Radiotherapy for treatment of lymphoma
- Immunotherapy other than study treatments for treatment of lymphoma
- Immunosuppressive therapy (except for medications indicated per protocol, including those listed in the exclusion criteria [Section 4.1.2], corticosteroids, and tocilizumab)
- Hormone therapy for the treatment of cancer, whether approved by local regulatory authorities or investigational
  - Adjuvant endocrine therapy for non-metastatic, hormone-receptor positive breast cancer is permitted.
- Biologic or targeted agents other than study treatments intended for treatment of lymphoma
- Herbal therapies intended as treatment of lymphoma
- Any therapies intended for the treatment of lymphoma, whether approved by local regulatory authorities or investigational

Patients who require the use of any of these agents will be discontinued from study treatment. Patients who are discontinued from study treatment will be followed for safety outcomes for 90 days following the patient's last dose of study treatment or until the patient receives *next anti-lymphoma treatment*, whichever occurs first.

The above lists of medications are not necessarily comprehensive. The investigator should contact the Medical Monitor if questions arise regarding medications not listed above.

4.4.4 Additional Restrictions

4.4.4.1 Immunizations

Patients who participate in the study may not receive either primary or booster vaccination with live virus vaccines for at least 4 weeks before initiation of or at any time

during study treatment and after the last dose until B-cell recovery to the normal ranges. Killed vaccines or toxoids should be given at least 4 weeks prior to the first dose of study treatment to allow development of sufficient immunity. Investigators should review the vaccination status of potential study patients being considered for this study and follow the U.S. Centers for Disease Control and Prevention guidelines for adult vaccination with non-live vaccines intended to prevent infectious diseases before study therapy. Patients who require the use of vaccination with live virus vaccines will be discontinued from study treatment.

4.5 STUDY ASSESSMENTS

Screening and pretreatment tests and evaluations will be performed within 14 days preceding the first dose of study treatment (except pretreatment biopsy, radiographic tumor assessment, and bone marrow aspirate and biopsy (if applicable), which may be performed up to 28 days preceding the first dose of study drug, providing no anti-tumor therapy was administered in this period). Results of standard-of-care tests or examinations performed prior to obtaining informed consent and within the screening window specified above may be used; these tests do not need to be repeated for screening.

The schedules of activities to be performed during the study are provided in Appendix 1–Appendix 6. All activities must be performed and documented for each patient.

Patients will be closely monitored for safety and tolerability throughout the study. Patients should be assessed for toxicity prior to each dose; dosing will occur only if the clinical assessment and local laboratory test values are acceptable.

4.5.1 Informed Consent Forms and Screening Log

Written informed consent for participation in the study must be obtained before performing any study-related procedures (including screening evaluations). Informed Consent Forms for enrolled patients and for patients who are not subsequently enrolled will be maintained at the study site.

All screening evaluations must be completed and reviewed to confirm that patients meet all eligibility criteria before enrollment. The investigator will maintain a screening log to record details of all patients screened and to confirm eligibility or record reasons for screening failure, as applicable.

4.5.2 Medical History, Concomitant Medication, and Demographic Data

Medical history, including clinically significant diseases, surgeries, cancer history (including prior cancer therapies and procedures, WHO 2016 classification, and current Ann Arbor stage), B symptoms (i.e., weight loss, night sweats, or fever), reproductive status, smoking history, and use of alcohol and drugs of abuse, will be recorded at

baseline. In addition, all medications (e.g., prescription drugs, over-the-counter drugs, vaccines, herbal or homeopathic remedies, nutritional supplements) used by the patient within 7 days prior to initiation of study treatment will be recorded. At the time of each follow-up physical examination, an interval medical history should be obtained and any changes in medications and allergies should be recorded.

Demographic data will include age, sex, and self-reported race/ethnicity. Race/ethnicity data is collected in order to assess whether the enrolled population is reflective of the general population and to evaluate whether different treatment effects are observed among different populations.

4.5.3 Physical Examinations

A complete physical examination should include an evaluation of the head, eyes, ears, nose, and throat, and the cardiovascular, dermatologic, musculoskeletal, respiratory, gastrointestinal, genitourinary, and neurologic systems. A complete neurologic examination, which includes an evaluation of mental status, cranial nerves, muscle strength, sensation, and coordination should be performed and documented in the patient chart. Any abnormality identified at baseline should be recorded on the General Medical History and Baseline Conditions eCRF.

A targeted physical examination limited to systems associated with symptoms should be performed at specified postbaseline visits and as clinically indicated. Targeted physical examinations should be limited to systems of primary relevance (i.e., cardiovascular, respiratory, neurologic, and any system that might be associated with tumor assessment, or potential drug-related toxicity [e.g., clinical assessment for peripheral neuropathy in patients receiving polatuzumab vedotin or vincristine]; see Section 5.1).

As part of tumor assessment, targeted physical examination should also include the evaluation of the presence and degree of enlarged lymph nodes, hepatomegaly, splenomegaly, or other findings of concern for lymphoma, which will be recorded on the appropriate Tumor Assessment eCRF.

Changes from baseline abnormalities should be recorded in patient notes.
New or worsened clinically significant abnormalities should be recorded as adverse events on the Adverse Event eCRF.

4.5.4 Prognostic Indices

FLIPI and FLIPI2 clinical factors obtained at diagnosis and at enrollment will be collected for patients with FL. IPI clinical factors at diagnosis and at enrollment will be collected for patients with DLBCL or transformed FL. MIPI clinical factors obtained at diagnosis and at enrollment will be collected for patients with MCL. See Appendix 11 for description of the FLIPI, FLIPI2, IPI, and MIPI.

4.5.5 Vital Signs

Vital signs will include measurements of systolic and diastolic blood pressure, respiratory rate, pulse oximetry, pulse rate, and body temperature while the patient is in a sitting or semi-supine position. Every effort should be made to ensure that vital signs are obtained from patients in a consistent manner/position. Weight, height, and BSA will also be recorded (Appendix 1). Height and BSA are required at screening only (within 96 hours of C1D1), unless there has been > 10% change in body weight since the last BSA assessment, in which case BSA should be recalculated and documented in the eCRF.

Vital signs for patients should be obtained according to the schedule of activities in Appendix 1. Additional vital sign monitoring should be performed if clinically indicated.

During the administration of study treatment, vital signs should be assessed according to the schedule of activities in Appendix 1.

4.5.6 Tumor and Response Evaluations

All evaluable or measurable disease must be documented at screening and re-assessed at each subsequent tumor evaluation. Response will be assessed by the investigator on the basis of clinical assessments, CT scans, PET-CT scans, and bone marrow examinations (if applicable) using the Lugano 2014 criteria (Cheson et al. 2014). See Section 3.1.1, Appendix 1, and Appendix 2 for further details on response assessment schedule.

4.5.6.1 Radiographic Assessments

PET scans, in conjunction with diagnostic-quality CT scans, will be obtained in this study. PET-CT scans should include skull-base to mid-thigh. Full-body PET-CT scan should be performed when clinically appropriate.

CT scans with oral and IV contrast should include chest, abdomen, and pelvic scans; CT scans of the neck should be included if clinically indicated. CT scans for response assessment may be limited to areas of prior involvement only if required by local regulatory authorities.

In patients for whom contrast is contraindicated, (e.g., patients with contrast allergy or impaired renal clearance), CT or combined PET-CT scans without contrast are permitted so long as they permit consistent and precise measurement of target lesions during the study treatment period. For patients for whom CT is contraindicated, MRI scans may be performed instead. Details regarding imaging procedures in these cases will be provided in the Imaging Manual.

PET and diagnostic-quality CT scans are required at screening, at the interim response assessment, and at the PRA visit. During follow-up, CT scans with or without PET scans may be utilized. Before a metabolic complete response is achieved, it is recommended

that PET scans should continue in conjuction with diagnositic-quality CT scans. Additionally, if disease progression or relapse is suspected before the PRA, both PET and diagnostic-quality CT scans should be performed for tumor assessment. The Lugano 2014 criteria (Appendix 7) will be used to assess overall response to study treatment.

The same radiographic assessment modality should be used for all response evaluations to ensure consistency across different timepoints.

A full tumor assessment including radiographic assessment must be performed any time disease progression or relapse is suspected.

4.5.6.2 Bone Marrow Assessments

Bone marrow examinations are required at screening for staging purposes for patients with FL and MCL. However, if the bone marrow involvement of lymphoma is confirmed by the presence of circulating lymphoma cells, the screening bone marrow biopsy may be omitted. *Peripheral blood smear and/or flow cytometry (preferred) can be used in place of a bone marrow biopsy to confirm the presence of circulating lymphoma cells.* For patients with DLBCL, screening PET/CT scans may be utilized to assess bone marrow involvement; bone marrow examinations are not required unless clinically indicated (Cheson et al. 2014). The screening bone marrow may be obtained within 28 days before the start of study treatment. In addition, the definition of CR for CT-based response requires clearing of previously infiltrated bone marrow. Bone marrow examinations should include a biopsy for morphology and an aspirate for local hematology (local flow studies are optional).

Repeat bone marrow examinations are required in two circumstances.

- If there was bone marrow infiltration determined by bone marrow examination and/or presence of circulating lymphoma cells at screening, then a subsequent bone marrow biopsy (trephine) is required for clinical response evaluation for all patients who may have achieved a CR for CT-based response. The subsequent bone marrow biopsy should be performed within 28 days of the observed CR. In patients with a PR and continued bone marrow involvement, a subsequent bone marrow examination may be required to confirm a CR for CT-based response at a later timepoint.
- A bone marrow aspirate and biopsy should be done at the time of relapse or transformation if bone marrow involvement is suspected.
- For patients with DLBCL, PET/CT scans may be utilized to assess bone marrow involvement, and repeat bone marrow examinations are not required unless clinically indicated (Cheson et al. 2014).

Any additional (unscheduled) bone marrow examinations performed during the study will be at the discretion of the investigator.

Bone marrow biopsy and aspirates do not need to be sent to the central laboratory; however, the associated hematopathology report should be submitted when available.

4.5.7 Assessment of Transplant Eligibility Status

Assessment of transplant eligibility status by the investigator will include questions on whether patient is transplant eligible (yes/no), and if no, reasons for not transplant eligible including, for example, disease not in remission, co-morbidity precluding transplant, donor stem cells unavailable, physician decision, and patient decision. The assessment of transplant eligibility status will be performed at screening. Additional unscheduled assessment of transplant eligiility status will be at the discretion of the investigator.

4.5.8 Laboratory, Biomarker, and Other Biological Samples

Samples for the following laboratory tests will be sent to the study site's local laboratory for analysis.

- Hematology: CBC (including hemoglobin, hematocrit, RBC, WBC), platelet count, ANC, absolute lymphocyte count, and other cells
- A peripheral blood smear and/or flow cytometry (preferred) to detect malignant cells are to be performed at screening (if not done as part of standard-of-care tests) (Section 5.1.2.1).
- Coagulation: aPTT, PT, INR, and fibrinogen
  - Fibrinogen will be collected when monitoring systemic immune activation events (e.g., MAS/HLH, severe CRS).
- Quantitative Igs (IgA, IgG, and IgM)
- Serum chemistry: sodium, potassium, chloride, bicarbonate, glucose, BUN or urea, creatinine, calcium, magnesium, phosphorous, total and direct bilirubin, total protein, albumin, ALT, AST, ALP, gamma-glutamyl transferase, LDH, and uric acid
- Beta-2 microglobulin
- C-reactive protein
- Serum ferritin
- Viral serology and detection
  - Hepatitis B (HBsAg, hepatitis B surface antibody [HBsAb], and hepatitis B core antibody [HBcAb]; HBV DNA by PCR if acute or chronic HBV infection cannot be ruled out by serology results [www.cdc.gov/hepatitis/hbv/pdfs/serologicchartv8.pdf])
  - HCV antibody; HCV RNA by PCR if the patient is HCV antibody positive
  - EBV and CMV by quantitative PCR using peripheral blood samples

  Notes: Blood samples should also be collected for central laboratory assessments. If local laboratory assessments are not available for quantitative PCR detection of active EBV and CMV, local laboratory collections may be

waived only if samples are collected for central laboratory assessments of viral infections.

  - HIV serology

- Pregnancy test
  - All women of childbearing potential will have a serum pregnancy test at screening (within 7 days prior to C1D1). Urine or serum pregnancy tests will be performed at specified subsequent visits (Appendix 1). If a urine pregnancy test is positive, it must be confirmed by a serum pregnancy test.

Samples for the following laboratory tests will be sent to one or several central laboratories or to the Sponsor or designee for analysis:

- Whole blood samples for flow cytometry and PBMC isolation
- Plasma for cytokines, including but not limited to IL-6 and IFN-γ
- Serum samples will be obtained for measurement of cytokines and IL-6 PD markers in patients treated with tocilizumab.
- Plasma for circulating CD20
- Blood for assessment of minimal residual disease status (expansion cohorts only)
- Samples may only be analyzed for selected patients.
- Serum samples for measurement of mosunetuzumab concentrations using mosunetuzumab validated PK assay
- Serum samples for measurement of total antibody analyte concentrations for polatuzumab vedotin using polatuzumab vedotin validated PK assay
- Lithium heparin plasma for polatuzumab vedotin acMMAE and unconjugated MMAE using a validated PK assay
- Serum samples will be obtained for measurement of tocilizumab concentration using validated tocilizumab PK assay in patients treated with tocilizumab
- Blood samples will be collected for viral infection test for quantitative PCR detection of viral infection that may include, but is not limited to, EBV and CMV
- Serum samples for measurement of ADAs to mosunetuzumab using validated assays
- Serum samples will be obtained for measurement of ADAs to polatuzumab vedotin using validated assays
- Serum samples for measurement of ADAs to tocilizumab using validated assays
- Tumor biopsies from safely accessible tumor sites (i.e., without unacceptable risk of a major procedural complication(s) per investigator assessment). Samples collected via resection, core-needle biopsy, or excisional, incisional, punch, or forceps biopsies are preferred. Tumor tissue from fine-needle aspirates or bone metastases that have been decalcified is not acceptable. The specimen must contain adequate evaluable tumor cells ($\geq$20% for excisional biopsy and $\geq$50% for core biopsy). Sites are strongly encouraged to submit a representative formalin-

fixed, paraffin-embedded tumor specimen in a paraffin block (preferred) or at least 20 slides containing unstained, freshly cut, serial sections. Tumor biopsies are required at the following timepoints:

- Pretreatment biopsy: Biopsies performed at any time between the last dose of last prior anti-cancer therapy and the first dose of mosunetuzumab or polatuzumab vedotin or prior to control arm chemoimmunotherapy regimens are required if judged safe and practical by investigator as described immediately above. Where possible, the tumor site being biopsied should be sufficiently large and accessible to permit a total of at least two biopsies (pretreatment and on-treatment) without unacceptable risk of a major procedural complication. Patients must have at least one measurable lesion that will not be biopsied in order to preserve the ability to radiographically assess tumor response.
- Fresh pretreatment biopsy is preferred. Patients who are unable to undergo biopsy procedures may be eligible for study enrollment if archival tumor tissue samples (paraffin blocks or at least 20 unstained slides) can be sent to the Sponsor. Biopsies obtained at any time between the last dose of last prior anti-cancer therapy and the first dose of study treatment are acceptable.
- On-treatment biopsy: If possible, on-treatment biopsy should be performed on the same lesion on which the screening biopsy was done. If this is not possible, a biopsy from another safely accessible site is permitted provided that there is at least one measurable lesion that is not biopsied in order to preserve the ability to radiographically assess tumor response. *The on-treatment biopsy may be omitted if there is no lesion available to biopsy due to response to treatment or the on-treatment biopsy may cause additional safety risk(s) for the patient.* The timing of on-treatment biopsies are detailed in Appendix 1.
- Re-treatment biopsy: Patients proceeding to re-treatment following disease progression will need to complete screening assessments (Appendix 1) to re-confirm eligibility, including undergoing a repeat tumor biopsy from a safely accessible site. Patients who have no lesion amenable for biopsy at disease progression may still be considered for study drug re-treatment (Section 3.1.5).
- Additional tumor biopsies are optional and may be performed at the investigator's discretion (e.g., to confirm disease recurrence or progression or to confirm an alternate histologic diagnosis); see Section 4.5.12. Where possible, tissue from these biopsies should be provided to the Sponsor for exploratory analyses.

All biopsies, whether fresh or archival, must be accompanied by the associated pathology report.

Exploratory biomarker research in tumor tissue and blood may include, but will not be limited to, analysis of genes or gene signatures associated with tumor immunobiology, prognostic or predictive markers associated with response to mosunetuzumab and polatuzumab vedotin, markers associated with T-cell activation, localization, and

activation status of immune cells and their subsets, and may involve extraction of DNA, circulating tumor DNA or RNA, analysis of somatic mutations, and use of next-generation sequencing (NGS). Assays for exploratory analysis include, but are not limited to, IHC, immunofluorescence, and RNA sequencing. Additional exploratory biomarkers may be assessed based on evolving clinical and nonclinical data.

Screening blood and tumor tissue samples, including those collected from patients who do not enroll in the study, may be used for future research and/or development of disease-related tests or tools.

For sampling procedures, storage conditions, and shipment instructions, see the laboratory manual.

Unless the patient gives specific consent for his or her leftover samples to be stored for optional exploratory research (see Section 4.5.13), biological samples will be destroyed when the final Clinical Study Report has been completed, with the following exceptions:

- Serum samples collected for PK or immunogenicity analysis may be needed for additional immunogenicity characterization and PK and immunogenicity assay development and validation; therefore, these samples will be destroyed no later than 5 years after the final Clinical Study Report has been completed.
- Blood, plasma, serum, and fresh tumor samples and their derivatives collected for biomarker research will be destroyed no later than 5 years after the final Clinical Study Report has been completed
- For enrolled patients, remaining archival tissue blocks will be returned to the site upon request or 18 months after final closure of the study database, whichever occurs first. For patients who are not enrolled, remaining archival tissue blocks will be returned to the site no later than 3 months after eligibility determination.

When a patient withdraws from the study, samples collected prior to the date of withdrawal may still be analyzed, unless the patient specifically requests that the samples be destroyed or local laws require destruction of the samples. However, if samples have been tested prior to withdrawal, results from those tests will remain as part of the overall research data.

Data arising from sample analysis will be subject to the confidentiality standards described in Section 8.4.

4.5.9 Electrocardiograms

Single ECG recordings will be obtained at screening and at end of treatment, as outlined in the schedule of activities (see Appendix 1) and may be obtained at unscheduled timepoints as indicated.

All ECG recordings must be performed using a standard high-quality, high-fidelity digital electrocardiograph machine equipped with computer-based interval measurements.

Lead placement should be as consistent as possible. ECG recordings must be performed after the patient has been resting in a supine position for at least 10 minutes. All ECGs are to be obtained prior to other procedures scheduled at that same time (e.g., vital sign measurements, blood draws). Circumstances that may induce changes in heart rate, including environmental distractions (e.g., television, radio, conversation) should be avoided during the pre-ECG resting period and during ECG recording.

For safety monitoring purposes, the investigator must review, sign, and date all ECG tracings. Paper copies of ECG tracings will be kept as part of the patient's permanent study file at the site. Digital recordings will be stored at the site. The following should be recorded in the appropriate eCRF: heart rate, RR interval, QRS interval, PR duration, uncorrected QT interval, and QT interval corrected through use of Fridericia's formula (QTcF) based on the machine readings of the individual ECG tracings. Any morphologic waveform changes or other ECG abnormalities must be documented on the eCRF. If considered appropriate by the Sponsor, ECGs may be analyzed retrospectively at a central laboratory.

If at a particular postdose timepoint the mean QTcF is > 500 ms and/or > 60 ms longer than the baseline value, another ECG must be recorded, ideally within the next 5 minutes, and ECG monitoring should continue until QTcF has stabilized on two successive ECGs. The Medical Monitor should be notified. Standard-of-care treatment may be instituted per the discretion of the investigator. If a PK sample is not scheduled for that timepoint, an unscheduled PK sample should be obtained. A decision on study drug discontinuation should be made, as described in Section 4.6.1. The investigator should also evaluate the patient for potential concurrent risk factors (e.g., electrolyte abnormalities, co-medications known to prolong the QT interval, severe bradycardia).

4.5.10 Patient-Reported Outcomes

For Arm K, PROs will be evaluated in patients using EQ-5D-5L questionnaire at timepoints detailed in Appendix 1. This questionnaire will be used to capture a patient's assessment of his or her overall health status.

The questionnaire, translated into the local language, as appropriate, will be completed in their entirety at specified timepoints during the study. To ensure instrument validity and that data standards meet health authority requirements, questionnaire will be self-administered before the patient receives any information on disease status, prior to the performance of non-PRO assessments (except laboratory blood collections), and prior to administration of study treatment, unless otherwise specified. Study site staff will ensure that PRO questionnaire is provided to patients for completion per the schedule of activities; before patients complete the visit, the site staff will confirm completion or alternatively document any reasons for not completing the questionnaire. In the event that the patient completes the PRO questionnaire on a scheduled assessment but is not

subsequently dosed, this will be recorded as an unplanned visit, and the PRO will be administered again at the subsequent rescheduled treatment visit.

4.5.10.1 EQ-5D-5L

The EQ-5D-5L (Appendix 12) is a generic, preference-based health utility measure that assesses health status and is used to inform pharmacoeconomic evaluations.

The EQ-5D-5L consists of two parts. The first part, health state classification, contains five dimensions of health: mobility, self-care, usual activities, pain/discomfort, and anxiety/depression (Herdman et al. 2011; Janssen et al. 2013). Published weights are available that allow for the creation of a single summary score. Overall scores typically range from 0–1, with low scores representing a higher level of dysfunction. The second part is a 0–100-point visual analog scale that assesses current health status, where higher scores are reflective of better health.

4.5.11 *Cumulative Illness Rating Scale-Geriatrics (CIRS-G)*

The CIRS-G is a validated version of the CIRS in the geriatric population (Parmelee et al. 1995) that was revised to reflect common problems of the elderly/unfit and was shown to be a valid indicator of health status in this patient population. The scale provides a comprehensive review of medical problems by organ system (14 organ systems), based on a 0–4 rating, yielding a cumulative score (0=No problem; 1=Current mild problem or past significant problem; 2 =Moderate disability or morbidity/requires "first line" therapy; 3 =Severe/constant significant disability/"uncontrollable" chronic problems; 4 =Extremely Severe/immediate treatment required/end organ failure/severe impairment in function) (Salvi et al. 2008) (Appendix 16). Of note, the CIRS-G assessment will be required as a screening assessment only for patients who are ≥65 years old for Arms J, L and M only, and will be completed in their entirety by the investigator or other designated study site team member.

A key consideration for the CIRS-G assessment in this study is that the hematopoietic score should not include lymphoma or hematologic deficiencies due to lymphoma. This is in alignment with the approach taken by the Fondazione Italiana Linfomi (FIL) group, which created and validated the simplified geriatric assessment (sGA), an objective tool to evaluate elderly patient fitness. The sGA comprises several components, including the CIRS-G assessment (Merli et. al 2021). Within the CIRS-G assessment used by the FIL, hematological comorbidities were excluded from the assessment of fitness (Merli et. al 2014). Thus, GO40516 will take a similar approach and exclude any lymphoma or hematologic deficiencies due to lymphoma from the hematopoietic score, considering that all patients in the study have lymphoma at study entry. Of note, any underlying hematological deficiencies not related to lymphoma will still be captured in the hematopoietic score.

4.5.12 Optional Tumor Biopsies

Consenting patients will undergo an optional tumor biopsy at disease progression, after treatment initiation, and may undergo additional on-treatment biopsies at any other time at the investigator's discretion (if deemed clinically feasible by the investigator). Samples collected via resection, core-needle biopsy (at least three cores preferred), or excisional, incisional, punch, or forceps biopsy are preferred. Cytological or fine-needle aspiration samples are not acceptable. For sampling procedures, storage conditions, and shipment instructions, see the laboratory manual.

The Informed Consent Form will contain a separate section that addresses optional biopsies. A separate, specific signature will be required to document a patient's agreement to undergo optional biopsies. The investigator should document whether or not the patient has given consent to participate and (if applicable) the date(s) of consent, by completing the Optional Biopsy Sample Informed Consent eCRF.

Samples may be used for exploratory biomarker research as described in Section 4.5.8. Refer to Section 4.5.8 for details on sample storage, use of samples after patient withdrawal, and confidentiality standards for data.

4.5.13 Optional Samples for Research Biosample Repository

4.5.13.1 Overview of the Research Biosample Repository

The RBR is a centrally administered group of facilities used for the long-term storage of human biologic specimens, including body fluids, solid tissues, and derivatives thereof (e.g., DNA, RNA, proteins, peptides). The collection, storage, and analysis of RBR specimens will facilitate the rational design of new pharmaceutical agents and the development of diagnostic tests, which may allow for individualized drug therapy for patients in the future.

RBR specimens will be used to achieve the following objectives:

- To study the association of biomarkers with efficacy, adverse events, or disease progression
- To increase knowledge and understanding of disease biology
- To study drug response, including drug effects and the processes of drug absorption and disposition
- To develop biomarker or diagnostic assays and establish the performance characteristics of these assays

4.5.13.2 Approval by the Institutional Review Board or Ethics Committee

Collection and submission of biological samples to the RBR is contingent upon the review and approval of the exploratory research and the RBR portion of the Informed Consent Form by each site's Institutional Review Board or Ethics Committee (IRB/EC) and, if applicable, an appropriate regulatory body. If a site has not been granted

approval for RBR sampling, this section of the protocol (Section 4.5.13) will not be applicable at that site.

4.5.13.3 Sample Collection

The following samples will be stored in the RBR and used for research purposes, including, but not limited to, research on biomarkers related to mosunetuzumab and polatuzumab vedotin or diseases:

- Blood sample collected at baseline (predose C1D1)
- Leftover blood, serum, plasma, PBMCs, and tumor tissue samples (with the exception of remaining archival tissue blocks, which will be returned to sites) and any derivatives thereof (e.g., DNA, RNA, proteins, peptides)

The above samples may be sent to one or more laboratories for analysis of germline or somatic mutations via whole genome sequencing (WGS), whole exome sequencing (WES), NGS, or other genomic analysis methods.

Genomics is increasingly informing researcher's understanding of disease pathobiology. WGS and WES provide a comprehensive characterization of the genome and exome, respectively, and, along with clinical data collected in this study, may increase the opportunity for developing new therapeutic approaches. Data will be analyzed in the context of this study but will also be explored in aggregate with data from other studies. The availability of a larger dataset will assist in identification of important pathways, guiding the development of new targeted agents.

For sampling procedures, storage conditions, and shipment instructions, see the laboratory manual.

RBR specimens are to be stored no later than 15 years after the final Clinical Study Report has been completed. However, the RBR storage period will be in accordance with the IRB/EC-approved Informed Consent Form and applicable laws (e.g., health authority requirements).

4.5.13.4 Confidentiality

Specimens and associated data will be labeled with a unique patient identification number.

Patient medical information associated with RBR specimens is confidential and may be disclosed to third parties only as permitted by the Informed Consent Form (or separate authorization for use and disclosure of personal health information) signed by the patient, unless permitted or required by law.

Given the complexity and exploratory nature of the analyses of RBR specimens, data derived from these analyses will generally not be provided to study investigators or

patients unless required by law. The aggregate results of any conducted research will be available in accordance with the effective Sponsor policy on study data publication.

Data generated from RBR specimens must be available for inspection upon request by representatives of national and local health authorities, and Sponsor monitors, representatives, and collaborators, as appropriate.

Any inventions and resulting patents, improvements, and/or know-how originating from the use of the RBR data will become and remain the exclusive and unburdened property of the Sponsor, except where agreed otherwise.

4.5.13.5 Consent to Participate in the Research Biosample Repository

The Informed Consent Form will contain a separate section that addresses participation in the RBR. The investigator or authorized designee will explain to each patient the objectives, methods, and potential hazards of participation in the RBR. Patients will be told that they are free to refuse to participate and may withdraw their consent at any time and for any reason during the storage period. A separate, specific signature will be required to document a patient's agreement to provide optional RBR specimens. Patients who decline to participate will not provide a separate signature.

The investigator should document whether or not the patient has given consent to participate and (if applicable) the date(s) of consent, by completing the RBR Research Sample Informed Consent eCRF.

In the event of an RBR participant's death or loss of competence, the participant's specimens and data will continue to be used as part of the RBR research.

4.5.13.6 Withdrawal from the Research Biosample Repository

Patients who give consent to provide RBR specimens have the right to withdraw their consent at any time for any reason. After withdrawal of consent, any remaining samples will be destroyed or will no longer be linked to the patient. However, if RBR specimens have been tested prior to withdrawal of consent, results from those tests will remain as part of the overall research data. If a patient wishes to withdraw consent to the testing of his or her specimens, the investigator must inform the Medical Monitor in writing of the patient's wishes through use of the appropriate RBR Subject Withdrawal Form and, if the trial is ongoing, must enter the date of withdrawal on the RBR Research Sample Withdrawal of Informed Consent eCRF. If a patient wishes to withdraw consent to the testing of his or her RBR samples after closure of the site, the investigator must inform the Sponsor by emailing the study number and patient number to the following email address:

global.rcr-withdrawal@roche.com

A patient’s withdrawal from Study GO40516 does not, by itself, constitute withdrawal of specimens from the RBR. Likewise, a patient’s withdrawal from the RBR does not constitute withdrawal from Study GO40516.

4.5.13.7 Monitoring and Oversight

RBR specimens will be tracked in a manner consistent with Good Clinical Practice by a quality-controlled, auditable, and appropriately validated laboratory information management system, to ensure compliance with data confidentiality as well as adherence to authorized use of specimens as specified in this protocol and in the Informed Consent Form. Sponsor monitors and auditors will have direct access to appropriate parts of records relating to patient participation in the RBR for the purposes of verifying the data provided to the Sponsor. The site will permit monitoring, audits, IRB/EC review, and health authority inspections by providing direct access to source data and documents related to the RBR samples.

4.6 TREATMENT, PATIENT, STUDY, AND SITE DISCONTINUATION

4.6.1 Study Treatment Discontinuation

Patients must permanently discontinue study treatment if they experience any of the following:

- Any medical condition that the investigator or Sponsor determines may jeopardize the patient’s safety if he or she continues to receive study treatment
- Investigator or Sponsor determines it is in the best interest of the patient
- Pregnancy
- Use of an anti-cancer therapy not required per protocol
- Symptomatic deterioration attributed to disease progression
- Confirmed disease progression per investigator assessment according to Lugano 2014

– For patients treated with mosunetuzumab (Groups A, B, and C; Arms I, J, K, *and L*), study treatment may be continued or re-initiated as outlined in Sections 3.1.4 and 3.1.5.

If mosunetuzumab is discontinued, the patient should be discontinued from all study treatment. The primary reason for study treatment discontinuation should be documented on the appropriate eCRF. Patients who discontinue study treatment prematurely will not be replaced, unless enrolled in the dose-escalation cohorts and discontinue prior to completion of the DLT assessment windows for Groups A, B, and C.

Patients will return to the clinic for a treatment completion or treatment discontinuation visit 30 (± 7) days after the last dose of study drug (see Appendix 1 for additional details).

After treatment discontinuation, information on survival follow-up and new anti-*lymphoma* therapy will be collected via telephone calls, patient medical records, and/or clinic visits approximately every 3 months until death or loss to follow-up (unless the patient withdraws consent or the Sponsor terminates the study). Information on subsequent anti-*lymphoma* therapies will include systemic therapies (e.g., chemotherapy, targeted therapy, hormonal therapy), surgery (e.g., resection of metastatic disease), SCT, CAR-T cell therapy and radiation procedures (radiotherapy to a tumor lesion).
If the patient withdraws from the study, the site's staff may use a public information source (e.g., county records) to obtain information about survival status only.

4.6.2 Patient Discontinuation from Study

Patients have the right to voluntarily withdraw from the study at any time for any reason. In addition, the investigator has the right to withdraw a patient from the study at any time. Reasons for withdrawal from the study may include, but are not limited to, the following:

- Patient withdrawal of consent
- Study termination or site closure
- Patient non-compliance, defined as failure to comply with protocol requirements as determined by the investigator or Sponsor

Every effort should be made to obtain information on patients who withdraw from the study. The primary reason for withdrawal from the study should be documented on the appropriate eCRF. Patients who withdraw from the study will not be replaced unless enrolled in the dose-escalation cohorts and discontinue prior to completion of the DLT assessment windows for Groups A, B, and C.

If a patient withdraws from the study, the study staff may use a public information source (e.g., county records) to obtain information about survival status.

4.6.3 Study Discontinuation

The Sponsor has the right to terminate this study at any time. Reasons for terminating the study may include, but are not limited to, the following:

- The incidence or severity of adverse events in this or other studies indicates a potential health hazard to patients
- Patient enrollment is unsatisfactory

The Sponsor will notify the investigator if the Sponsor decides to discontinue the study.

4.6.4 Site Discontinuation

The Sponsor has the right to close a site at any time. Reasons for closing a site may include, but are not limited to, the following:

- Excessively slow recruitment
- Poor protocol adherence

- Inaccurate or incomplete data recording
- Non-compliance with the International Council for Harmonisation (ICH) guideline for Good Clinical Practice
- No study activity (i.e., all patients have completed the study and all obligations have been fulfilled)

5. ASSESSMENT OF SAFETY

5.1 SAFETY PLAN

This is the first combination study in which mosunetuzumab will be administered to humans in combination with polatuzumab vedotin. *On 3 June 2022, mosunetuzumab (Lunsumio®) as a monotherapy has been conditionally approved by the European Commission for the treatment of adult patients with R/R FL who have received at least two prior systemic therapies. Clinical development is ongoing for additional indications including aggressive NHL. On 24 May 2022, the European Commission granted the full marketing authorisation for the use of polatuzumab vedotin (Polivy®) in combination with R-CHP for the treatment of adult patients with first-line DLBCL. Polatuzumab vedotin has also been approved in combination with bendamustine and rituximab* in *many* countries, *including the European Union and the United States,* for the treatment of adult patients with R/R DLBCL. Because the combination of mosunetuzumab plus polatuzumab vedotin is not yet approved, the safety plan for this study is primarily based on clinical experience with each investigational agent, as outlined in their respective Investigator's Brochures.

Measures will be taken to ensure the safety of patients participating in this trial, including the use of stringent inclusion and exclusion criteria (Sections 4.1.1 and 4.1.2) and close monitoring, as described below. As described in Section 3.1.2, enrollment of patients for DLT evaluation purposes will be staggered such that the first 2 patients in each dose-escalation cohort will have respective C1D1 treatments administered ≥72 hours apart. Subsequent patients in each cohort will be staggered such that their C1D1 treatments are administered ≥24 hours apart.

All patients will be monitored closely for toxicity. Patients will be assessed clinically for toxicity prior to each dose using the NCI CTCAE v5.0 grading scale unless otherwise stated. CRS severity will be graded according to the ASTCT CRS Consensus Grading criteria (Appendix 8). All adverse events and serious adverse events will be recorded during the trial and for up to 90 days after the last dose of study treatment or until the initiation of another systemic anti-cancer therapy, whichever occurs first. To mitigate potential unknown risks, dosing beyond C1 will be limited to patients who do not demonstrate unacceptable toxicity or compelling evidence of disease progression (see Section 3.1.2.6). See Section 5.3 for details regarding safety reporting for this study.

Specific anticipated or potential toxicities associated with administration of mosunetuzumab and polatuzumab, and the other investigational agents, as well as the measures taken to avoid or minimize such toxicities in this trial, are described in the following sections.

5.1.1 Mosunetuzumab Administration and Hospitalization

Administration of mosunetuzumab will be performed in a clinical setting with immediate access to a critical care unit and staff who are trained to monitor for and respond to medical emergencies. Neurology consultation services should be readily available to address any neurologic adverse events that may arise as a result of mosunetuzumab treatment (Section 5.1.2.2, Neurologic Adverse Events), and nephrology consultation with acute dialysis capabilities should be readily available to address any renal toxicity that might accompany TLS (Section 5.1.2.2, Tumor Lysis Syndrome).

All patients enrolled in **Group A dose escalation** will require inpatient monitoring, including hospitalization during or following mosunetuzumab administration when given in combination with polatuzumab vedotin for the first two occasions (i.e., on C1D1 and C2D1). Hospitalization will extend through at least 72 hours after the completion of mosunetuzumab administration on C1D1 (see above, Section 4.3.2.1, and Section 3.3.13), and for a minimum of 24 hours on C2D1. Patients may be hospitalized for monitoring on C1D8 or C1D15 for 24 hours at the discretion of the Investigator in consultation with the Medical Monitor.

All patients enrolled in **Group B dose escalation** will require inpatient monitoring, including hospitalization during or following first mosunetuzumab administration given on C1D8, for a minimum of 72 hours. All patients will again be hospitalized for a minimum of 24 hours on C2D1. Patients may be hospitalized for monitoring on C1D15 for 24 hours at the discretion of the Investigator in consultation with the Medical Monitor.

All patients enrolled in **Group C dose escalation** will require inpatient monitoring, including hospitalization during or following mosunetuzumab administration when given in combination with polatuzumab vedotin for the first time (i.e., C2D1). Hospitalization will extend through at least 72 hours after the completion of mosunetuzumab administration on C2D1.

Patients enrolled in the safety run-in for **Arm K** will require inpatient monitoring, including hospitalization during or following mosunetuzumab administration when given in combination with polatuzumab vedotin for the first time (i.e., C1D1). Hospitalization will extend through at least 24 hours after the completion of mosunetuzumab administration. Patients may be hospitalized for monitoring for subsequent doses for 24 hours at the discretion of the investigator in consultation with the Medical Monitor.

Hospitalization requirements during subsequent cycles for any individual patient will be determined on the basis of the clinical course during the first cycle (or the second cycle

for Group C); patients with Grade 3 IRR, CRS, injection-site reactions, or TLS during C1 (or during C2 for Group C) may also be hospitalized through at least 72 hours after the end of the administration of the subsequent dose.

Hospitalization for administration of previously assessed doses is not required unless clinically indicated at the study investigator's discretion and in consultation with the Medical Monitor. Examples where such hospitalization may be warranted include but are not limited to prior observed Grade ≥ 2 adverse events potentially attributable to mosunetuzumab at the same or similar dose, and TLS monitoring and prophylaxis.

Based on available clinical safety data and the recommendation of the IMC, for patients who receive mosunetuzumab at a dose level that has been tested to be safe and tolerable in dose escalation (or in the safety run-in for Arm K), hospitalization is not mandatory after any dosing day. This applies to patients enrolled in expansion cohorts and to those patients who receive study re-treatment. For patients enrolled in Arm K expansion, hospitalization will not be mandatory after any dosing day if no Grade 3 CRS is observed in the safety run-in group. Instead, the investigator should actively assess the need for hospitalization, and patients should be hospitalized after mosunetuzumab administration whenever clinically indicated.

For all treatment groups, decisions to modify or discontinue the requirement for hospitalization in expansion cohorts will be made based on the recommendation of the IMC (Section 3.1.5.2) and in consultation with study investigators.

5.1.2 Risks Associated with Mosunetuzumab

On the basis of clinical data to date with mosunetuzumab, the following known and suspected risks are described below. Guidelines around the management of these risks through dose and schedule modifications are described in Section 5.1.7. Refer to the Mosunetuzumab Investigator's Brochure for complete and updated details.

5.1.2.1 Known Risks Associated with Mosunetuzumab

Cytokine Release Syndrome

The mechanism of action of mosunetuzumab is immune cell-activation against CD20-expressing cells; therefore, a spectrum of events involving IRRs, target-mediated cytokine release, and/or hypersensitivity with or without emergent ADAs, may occur. Other CD20-directed therapies and immunomodulatory therapies have been associated with IRRs, CRS, and/or hypersensitivity (Rituxan USPI; Gazyva USPI; Blincyto® USPI).

CRS has been reported in Study GO29781 and is an identified risk of mosunetuzumab. Refer to the current Mosunetuzumab Investigator's Brochure for details.

To date, CRS observed with mosunetuzumab have been mostly mild to moderate in severity, and include symptoms such as fever, headache, and myalgia, and respond to symptomatic treatment with analgesics, anti-pyretics, and antihistamines as indicated.

Severe or life-threatening presentations of IRRs and/or CRS, such as hypotension, tachycardia, dyspnea, or chest discomfort, should be treated aggressively with supportive and resuscitative measures as indicated, including the use of tocilizumab and/or high-dose corticosteroids, IV fluids, and other supportive measures per local institutional practice. Severe CRS may be associated with other clinical sequelae such as disseminated intravascular coagulation and capillary leak syndrome, or may manifest as HLH (see Section 5.1.2.2). Standard of care for severe or life-threatening CRS resulting from immune-based monoclonal antibody therapy has not been established; case reports and recommendations for CD19 CAR-T have been published (Teachey et al. 2013; Lee et al. 2014, 2019; Maude et al. 2014; Neelapu et al. 2018; also see Section 5.1.2.2 and FDA approval for two products describing risk management for CRS [Yescarta USPI; Kymriah™ USPI]).

Disease-related factors that may be associated with an increased risk of severe CRS following CAR-T–cell therapy, and therefore, potentially other T-cell engaging therapies, include (but are not limited to) lymphoma bone marrow involvement, extranodal disease, B cell lymphocytosis, and the presence of circulating peripheral malignant cells. Patients with the above disease-related factors must be discussed with the Medical Monitor; additional monitoring (i.e., more frequent measurements of vital signs) during mosunetuzumab dosing (especially first dose) should be undertaken (Section 4.5.5), and management of treatment-emergent adverse events (including CRS) must adhere to guidance in Section 5.1.7.5.

To minimize the risk and sequelae of IRR and CRS, mosunetuzumab will be administered over a minimum of 4 hours in C1 in a clinical setting as described in Section 5.1.1. Corticosteroid premedication should be administered as described in Section 4.3.2.1.Management guidelines for CRS following mosunetuzumab are summarized in Table 18, with the grading of CRS following the ASTCT CRS Consensus Grading criteria described in Appendix 8. Given the mechanism of action of mosunetuzumab, IRRs and CRS may be indistinguishable; hence, their evaluation and treatment are identical (see Section 5.1.7.5 and Table 18). Management of Grade $\geq$ 3 CRS should be immediately discussed between the treating investigator and the Medical Monitor. As noted in Table 18 even moderate presentations of CRS in patients with extensive comorbidities should be monitored closely with consideration given to ICU admission and tocilizumab administration.

Refer to Section 5.3.5.1 for adverse event reporting procedures related to CRS.
If tocilizumab is administered, refer to Appendix 1 for the schedule of activities for tocilizumab treatment of CRS. Refer to Appendix 10 for anaphylaxis management.

Neutropenia

Neutropenia is a known class effect associated with other CD20-directed therapies as well as blinatumomab (Blincyto USPI). Reversible neutropenia has been observed following mosunetuzumab-treatment in Study GO29781. Some patients developing neutropenia have received growth factor support and/or temporary treatment holds. See Section 1.2.2.1 and the Mosunetuzumab Investigator's Brochure for details.

Patients who experience Grade 3–4 neutropenia should be closely monitored with more frequent assessments as applicable; see Section 4.4.1.1 for use of growth factor support.

Tumor Lysis Syndrome

TLS is a known pharmacodynamic effect of anti-tumor therapy in hematologic malignancies including NHL. TLS has been reported with blinatumomab, CAR-T cell therapy, and other CD20 directed therapy (Blincyto USPI; Gazyva USPI; Rituxan USPI; Porter et al. 2011). The inherent risk of TLS is dependent on the malignancy being treated and individual patient characteristics (Coiffier et al. 2008). There is the theoretical risk of TLS if treatment with mosunetuzumab results in the rapid destruction of a large number of tumor cells.

The risk of TLS with mosunetuzumab in patients with NHL is predicted to be highest for those with bulky disease (defined in the context of TLS as any lesion $\geq$ 10 cm on the screening CT scan) and elevated pretreatment LDH levels, particularly in the presence of dehydration or compromised renal function. While DLBCL, transformed lymphomas, and MCLs may be at higher risk of TLS as compared with follicular, marginal, and small-cell lymphomas (Cairo et al. 2010), any risk stratification based on tumor type must be considered along with the effectiveness of therapy (Howard et al. 2011).
As mosunetuzumab has the potential for potent B-cell killing (Section 1.2), patients will receive prophylaxis for TLS based on their risk (Section 5.1.7.8).

Prior to each mosunetuzumab treatment given during C1 and C2, the patient's serum chemistry and hematology laboratory samples should be obtained and reviewed and prophylactic measures initiated according to the guidelines described below. Access to nephrologist and acute dialysis services must be available in the event of clinically significant TLS (see Appendix 15).

Specific prophylaxis recommendations for the prevention of TLS are described in Section 5.1.7.8.

Infections

Due to its anticipated mode of action resulting in profound B-cell depletion, mosunetuzumab may be associated with an increased risk of infections. Infections have been reported in patients receiving other CD20-directed therapies as well as blinatumomab (Blincyto USPI; Gazyva USPI; Rituxan USPI). Therefore,

mosunetuzumab should not be administered in the presence of active severe infections. Investigators should exercise caution when considering the use of mosunetuzumab in patients with history of recurring or chronic infections or with underlying conditions that may predispose patients to infections. Signs and symptoms of infection should result in prompt evaluation and appropriate samples for bacteriological investigation prior to starting antibiotic or other treatment.

Particular attention should be given to patients who have had significant prior immunosuppressive treatment such as high-dose chemotherapy. PML has been associated with treatment with CD20-directed therapies including rituximab and obinutuzumab. The diagnosis of PML should be considered in any patient presenting with new-onset neurologic manifestations, and consultation with a neurologist and diagnostic procedures including brain MRI and lumbar puncture should be performed as clinically indicated. Note, however, that new onset neurologic adverse events following initial doses of mosunetuzumab may be more likely due to acute effects of mosunetuzumab- (see Section 5.1.2.2, Neurologic Adverse Events), as PML associated with rituximab generally occurred following long-term exposure (Carson et al. 2009).

Hepatitis B reactivation has been reported with other CD20-directed therapies. Patients with a history of chronic hepatitis B infection or positive test results for active or chronic. HBV infection defined by HBsAg and/or positive total HBcAb and positive HBV PCR, or patients with HCV infection as assessed by PCR, will be excluded from this trial (Section 4.1.2). Refer to Table 16 for management guidelines for patients who develop detectable hepatitis B viral load while in the study.

Patients w*ho are HIV positive may be eligible to enroll in the study if specific criteria are met (Section 4.1.1).* Signs and symptoms of HIV may confound assessment of the safety profile of mosunetuzumab in combination with polatuzumab vedotin. HIV has also been associated with development of secondary HLH. *Patients with positive HIV should be monitored per local institutional standards while receiving study treatment*.

Tumor Flare

Adverse events associated with tumor inflammation/flare have been reported in Study GO29781. Consistent with the mechanism of action of mosunetuzumab, tumor flare is likely due to the influx of T cells into tumor sites following mosunetuzumab administration and may be associated with pseudoprogression (refer to Section 3.1.4). Reported tumor flare associated adverse events to date have had a short time to onset following initial mosunetuzumab administration. Tumor flare may additionally occur in patients who are re-treated with mosunetuzumab following disease progression as described in Section 3.1.5. On the basis of safety data collected to date, tumor flare has manifested as new or worsening pleural effusions. In addition, depending on tumor size and anatomic location, tumor flare may potentially result in mass effects on vital structures including airways, major blood vessels, gastrointestinal tract (risk of perforation and hemorrhage), and/or major organs. If such manifestations are temporally associated

with early mosunetuzumab dosing, the treating physician/study investigator should consider those events to be tumor flare and report as “tumor flare” or “tumor inflammation”. Patients with tumors at critical anatomic locations should be closely monitored for tumor flare, and the treating physician/study investigator should perform risk assessments and establish risk mitigation strategies prior to mosunetuzumab treatment. Refer to the Mosunetuzumab Investigator’s Brochure for complete and updated safety information.

The recognition of tumor flare may be supported by clinical presentation and temporal association. Please see below for general guidance on how to assess for a possible tumor flare event:

- *Confirm temporal association with mosunetuzumab administration. Tumor flare events tend to have an early onset (Cycles 1-2), are transient and affect organ systems in proximity to tumor involvement.*
- *When possible and clinically indicated, obtain a biopsy of the involved site. If the clinical presentation involves a new or worsening pleural or pericardial effusion or ascites, a sample of the fluid may be collected and analyzed.*
- *If a sample can be obtained, assess for immune cell infiltration (e.g., by flow cytometry or immunohistochemistry). A confirmed tumor flare event is characterized by immune cell infiltrates on biopsy.*
- *Rule out other causes of a suspected tumor flare event, including infection and disease progression.*

Please see eCRF Completion Guidelines for guidance on how to report a Tumor Flare event.

Injection-Site Reactions

Localized injection-site reactions following SC administration of the anti-CD20 monoclonal antibody rituximab have been observed (Assouline et al. 2016). Most of these were mild to moderate in severity (MabThera® Summary of Product Characteristics). As CD4^{+} and CD8^{+} Tcells (Mueller et al. 2014) as well as B cells (Egbuniwe et al. 2015) reside in the skin, localized reactions following mosunetuzumab SC administration may occur. The injection-site reactions are an identified risk of mosunetuzumab. Injection-site reactions have been obsevered in Study GO29781. The manifestation included erythemia, pain, eczema, phlebitis, pruritis, and rash on injection site. *Please refer to Section 5.1.7.7 for injection site reactions management guidelines.*

5.1.2.2 Potential Risks Associated with Mosunetuzumab

Hemophagocytic Lymphohistiocytosis

CRS with features of adult-onset secondary HLH[1] has been reported with blinatumomab as well as CAR adoptive T-cell therapy (Blincyto USPI; Teachey et al. 2013;

[1] For the purposes of the Study GO40516 protocol and study-related documents, macrophage activation syndrome (MAS) and HLH are considered to be synonymous terms.

Lee et al. 2014). A fatal case of secondary HLH, in a patient positive for Epstein-Barr virus as assessed by EBV-encoded small RNA in situ hybridization, has been reported in Study GO29781 (refer to the current Mosunetuzumab Investigator's Brochure for details).

While severe CRS and secondary HLH have overlapping presentation and symptoms, secondary HLH may be precipitated by other conditions including infections, autoimmune disease and malignancies (Ramos-Casals 2014). The prevalence of these conditions in the study patient population makes the distinction between severe CRS and secondary HLH and identification of inciting factors challenging. On the one hand, in one series, B-cell malignancies were the most common malignancy associated with secondary HLH (Rivière et al. 2014). On the other hand, active infection with EBV is one of the most common infectious causes of secondary HLH (Hashemi-Sadraei et al. 2015; Schram and Berliner 2015), and reactivation of latent EBV may occur in patients with CLL (Rath et al. 2008), which in turn may lead to HLH (Lim et al. 2014). It remains unknown whether mosunetuzumab treatment may further increase the risk of developing HLH in patients who have additional risk factors.

In the setting of T-cell engaging therapies including mosunetuzumab, CRS is much more likely compared with secondary HLH; considering the overlapping presentation of symptoms, management of these patients should be primarily focused on treatment of CRS (see Table 18). In atypical cases such as late onset CRS (past completion of stepup dosing with mosunetuzumab-) or CRS that is refractory to treatment, work up for HLH should be initiated, and all cases of suspected HLH should be reported to the Medical Monitor immediately. While there is no currently universally accepted set of criteria for diagnosing secondary or reactive HLH in the adult population, proposed criteria have been published (Henter et al. 2007, Fardet et al. 2014; Hejblum et al. 2014, McClain and Eckstein 2019 see Section 5.1.7.6).

Neurological Adverse Events

Encephalopathy has been observed in in the setting of CRS and/or elevation in liver function tests (LFTs) following mosunetuzumab treatment (refer to the Mosunetuzumab Investigator's Brochure for details).

Neurologic toxicity has also been reported in patients treated with blinatumomab and CD19 CAR T-cell therapy (Blincyto USPI; Maude et al. 2014; Kochenderfer et al. 2015). Reported symptoms in patients treated with blinatumomab or CD19 CAR T-cell therapy have included headache, confusion, aphasia, encephalopathy, tremor, seizure, and other neurologic events. The etiology of toxicity in these settings is uncertain and may not be responsive to cytokine directed therapy such as tocilizumab, but has generally improved with treatment discontinuations and corticosteroids (Blincyto USPI; Viardot et al. 2010; Kochenderfer et al. 2015). In patients with B-cell acute lymphoblastic leukemia treated with blinatumomab, neurologic toxicities were observed in approximately 50% of patients; Grade ≥ 3 neurologic toxicity was observed in

approximately 15% of patients. The majority of neurologic adverse events resolved following interruption of blinatumomab, with some patients requiring treatment discontinuation (Blincyto USPI).

Neurological adverse events will be monitored closely during the trial. All patients will be required to undergo a baseline complete neurologic examination prior to the first mosunetuzumab administration; the examination should include an evaluation of mental status, cranial nerves, motor strength, sensation, and coordination. Results of the neurologic examination should be documented in the patient's chart. Patients with a history of neurologic disease may be excluded from this trial (see Section 4.1.2).

Patients should be routinely assessed for any signs or symptoms of neurological adverse events as part of the on-treatment clinical examination (see Section 4.5.3). If new or worsening neurological adverse events is suspected, the patient should be referred to a neurologist for further evaluation of potential drug-related neurotoxicity. Corticosteroids should be considered to treat suspected neurological adverse events. Imaging studies (e.g., diffusion-weighted MRI) should be performed if clinically indicated (see Table 21).

The investigator should instruct patients to refrain from driving or engaging in hazardous occupations or activities if the patients develop specific adverse events while on mosunetuzumab:

- For patients who develop a neurologic adverse event that may affect driving (see Appendix 13) and for patients who develop CRS, HLH, or Grade 3–4 LFT elevation, the investigator should advise patients to refrain from driving or engaging in hazardous occupations or activities until the event is resolved.
- Patients who develop tremor, dizziness, insomnia, or a Grade ≥3 neurologic adverse event should be assessed by neurologic examination to determine if the adverse event may impair the ability of the patient to drive or engage in hazardous occupations or activities. For patients assessed to be at increased risk, the investigator should advise the patient to refrain from driving or engaging in hazardous occupations or activities until the event is resolved.

Thrombocytopenia

Thrombocytopenia is associated with other CD20-directed therapies as well as blinatumomab (Blincyto USPI). Reversible thrombocytopenia has been observed following mosunetuzumab treatment in Study GO29781. Refer to the Mosunetuzumab Investigator's Brochure for details.

Patients should be closely monitored for thrombocytopenia; regular laboratory tests should be performed until the event resolves. Transfusion of blood products (e.g., platelet transfusion) according to institutional practice is at the discretion of the treating physician. Use of all concomitant therapies, which could possibly worsen

thrombocytopenia-related events, such as platelet inhibitors and anticoagulants, should also be taken into consideration.

Elevated Liver Enzymes and Hepatotoxicity

Transient Grade 3 AST elevation in the setting of Grade 2 CRS as well as Grade 3 hepatic encephalopathy/Grade 4 elevation in LFTs have been observed following mosunetuzumab treatment (refer to the Mosunetuzumab Investigator's Brochure for details).

Elevated liver enzymes have been reported with blinatumomab (Blincyto USPI), usually but not exclusively, in the setting of CRS. Grade ≥ 3 liver enzyme elevations occurred in approximately 6% of patients outside the setting of CRS. Nearly all liver enzyme elevations resolved either with blinatumomab treatment interruption or while treatment continued. Some patients with resolved liver enzyme elevations were successfully rechallenged, suggesting a first-dose effect rather than direct toxicity (Blincyto Drug Approval Package).

Patients who do not meet eligibility criteria for LFTs at screening will be excluded from this trial (Section 4.1.2). The LFTs will be assessed regularly during study and should be managed according to guidelines in Table 22. Patients with right upper-quadrant abdominal pain and/or unexplained nausea or vomiting should have LFTs performed immediately and reviewed before administration of the next dose of study drug.

For patients with elevated LFTs, concurrent medication, viral hepatitis, and toxic or neoplastic etiologies should be considered and addressed, as appropriate.

Immunogenicity (Anti-Drug Antibodies)

As with any recombinant antibody, mosunetuzumab may elicit an immune response, and patients may develop antibodies against the molecule. Patients will be closely monitored for any potential immune response to mosunetuzumab in the first-line treatment setting, which may have an impact on the benefit-risk profile of the agent. Therefore, a risk-based strategy (Rosenberg and Worobec 2004a, 2004b, 2005; Koren et al. 2008) will be utilized to detect and characterize ADA responses to mosunetuzumab. Out of 53 evaluable postbaseline patients tested in Phase Ia study of mosunetuzumab (Study GO29781), none of the patients was confirmed positive for anti-mosunetuzumab antibodies. Because mosunetuzumab is a B cell-depleting agent and has demonstrated low immunogenicity rates in the Phase I study, the frequency of ADA sampling times for mosunetuzumab will be reduced in this study compared with Study GO29781.

5.1.3 Risks Associated with Polatuzumab Vedotin

On the basis of clinical data to date, the following known and suspected risks are described below. Guidelines around the management of these risks through dose and

schedule modifications are described in Section 5.1.7. Refer to the Polatuzumab Vedotin Investigator's Brochure for complete and updated details.

5.1.3.1 Known Risks Associated with Polatuzumab Vedotin

Based on clinical experience with polatuzumab vedotin in patients treated in the current Phase I and Phase II studies, myelosuppression and peripheral neuropathy are identified risks of polatuzumab vedotin.

Myelosuppression: consolidation of neutropenia (including febrile neutropenia, thrombocytopenia, and anemia)

Neutropenia, neutropenia-associated events, thrombocytopenia, and anemia, including serious and severe cases, have been reported in patients receiving polatuzumab vedotin. Adequate hematologic function should be confirmed before initiation of study treatment. Patients receiving study treatment will be regularly monitored for evidence of marrow toxicity with complete blood counts. Study treatment for hematologic toxicities may be delayed or modified for hematologic toxicities as described in Table 16.

The use of G-CSF for neutropenia is described in Section 4.4.1.1.

Transfusion support for anemia and thrombocytopenia is permitted at the discretion of the investigator.

Peripheral Neuropathy

Patients receiving polatuzumab vedotin may develop peripheral neuropathy, including peripheral sensory and/or motor neuropathy. These events have generally been reversible to varying degrees as much as available, but it is not known if full reversibility can be expected or predicted. Patients in clinical trials with polatuzumab vedotin should be monitored for symptoms of neuropathy, including hypoesthesia, hyperesthesia, paresthesia, dysesthesia, discomfort, a burning sensation, weakness, gait disturbance, loss of balance, orthostatic hypotension, syncope, or neuropathic pain. Patients experiencing new or worsening peripheral neuropathy may require a dose delay, change in dose, or discontinuation of treatment and should be managed according to the protocol.

Study treatment dose and schedule modifications for peripheral neuropathy are described in Section 5.1.7.

Infections

Patients receiving polatuzumab vedotin may be at a higher risk of developing infections. Serious infections, including opportunistic infections, such as pneumonia (including *P*neumocystis jirovecii and other fungal pneumonia), bacteremia, sepsis, herpes infection, and cytomegalovirus infection have been reported in patients treated with polatuzumab vedotin. Several risk factors in the patient population under study influence patients' vulnerability to a higher risk of infections, particularly serious and opportunistic infections. These risk factors include predisposition of the indication

disease to infections, elderly population, comorbidity, and impaired capacity of bone marrow recovery with multiple lines of prior anti-cancer therapies. Infections have been reported in Phase I and II clinical studies with polatuzumab vedotin, including a case of PML in the setting of combined treatment with CD20-directed therapy.

In addition, neutropenia is a known risk for polatuzumab vedotin. Granulocytopenia is a major predisposing factor to infections in patients with B-cell lymphoma. The reported incidence of infection in chemotherapy courses for B-cell lymphoma associated with $<$ 500 granulocytes/µL was higher than those with $\geq$500 granulocytes/µL. Neutropenia events should be monitored closely and any signs of infection should be treated as appropriate (Taplitz et al. 2018).

Infusion-Related Events

Infusion related reactions have been reported in patients receiving polatuzumab vedotin. Commonly experienced events include nausea, vomiting, chills, fever, pruritus, hypotension, flushing, and other symptoms. In the majority of the patients, the events were Grade 1–2. Premedication and monitoring procedures for polatuzumab vedotin administration are outlined in Section 4.3.2.2.

Gastrointestinal Toxicity (Diarrhea, Nausea, Vomiting, Constipation, Anorexia)

Diarrhea, nausea, vomiting, constipation, and abdominal pain are reported frequently, with diarrhea and nausea being the most common ($\geq$20%) treatment-emergent adverse events in Phase I and II clinical studies with polatuzumab vedotin. Diarrhea has been responsible for study drug modification and discontinuation. Most cases were low grade, with more serious cases being confounded by polypharmacy, comorbidities, or disease under study.

Refer to the Polatuzumab Vedotin Investigator Brochure for additional information.

5.1.3.2 Potential Risks Associated with Polatuzumab Vedotin

Tumor Lysis Syndrome

There is a potential risk of TLS if treatment with polatuzumab vedotin results in the rapid destruction of a large number of tumor cells.

TLS prophylaxis for treatment groups or arms including mosunetuzumab will follow the prophylaxis measures as described in Section 5.1.2.2.

Patients who are considered to have a high tumor burden (e.g., lymphocyte count $\geq 25 \times 10^9$/L or bulky lymphadenopathy) and who are considered to be at risk for TLS by the investigator will receive TLS prophylaxis (e.g., allopurinol $\geq$300 mg/day by mouth or a suitable alternative treatment such as rasburicase starting 48–72 hours before study treatment) and must be well hydrated before the initiation of study treatment at C1D1. These patients should continue to receive repeated prophylaxis and adequate hydration, as deemed appropriate by the investigator.

For patients with evidence of TLS, all study treatment should be suspended and the patient should be treated as clinically indicated. Following the complete resolution of TLS complications, treatment may be resumed at the full dose at the next scheduled infusion in conjunction with prophylactic therapy.

Immunogenicity (Anti-Drug Antibodies)

As with any recombinant antibody, polatuzumab vedotin may elicit an immune response, and patients may develop antibodies against the molecule. Out of 481 evaluable postbaseline patients in the Phase Ib and II studies of polatuzumab vedotin (Studies GO29365, GO27834, GO29044, and JO29138), only 10 (2.1%) patients tested positive for ADAs that were treatment-induced for polatuzumab vedotin. No conclusions could be drawn concerning the potential impact of ADAs on pharmacokinetics, efficacy, or safety due to a limited, very low number of ADA-positive patients for polatuzumab vedotin.

Patients will be closely monitored for any potential immune response to polatuzumab vedotin in the R/R setting, which may have an impact on the benefit-risk profile of the agent. Therefore, a risk-based strategy (Rosenberg and Worobec 2004a, 2004b, 2005; Koren et al. 2008) will be utilized to detect and characterize ADA responses to polatuzumab vedotin. Because polatuzumab vedotin is a B-cell–depleting agent and has demonstrated low immunogenicity rates in the Phase I and II study, the frequency of ADA sampling times will be reduced in this study compared with previous studies.

Reproductive Toxicity

Adverse effects on human reproduction and fertility are anticipated with the administration of polatuzumab vedotin, given the mechanism of action of MMAE. Standard exclusion criteria are used to ensure that patients of childbearing potential (male or female) are using adequate contraceptive methods.

Hyperglycemia

Hyperglycemia has been observed in patients treated with polatuzumab vedotin as well as with other ADCs that use the same valine-citrulline–MMAE platform. Hyperglycemia has been reversible upon holding or discontinuing treatment of the ADCs and/or initiation or adjustment of anti-hyperglycemic medications.

Hepatotoxicity

Hepatotoxicity has been observed in patients treated with polatuzumab vedotin in both the Phase I and II trials. Although the relationship between hepatotoxicity and polatuzumab vedotin has not been definitively determined, transient, dose-related increases in hepatic enzymes were noted in nonclinical rat studies. No hepatotoxicity was noted following administration of the surrogate ADC in cynomolgus monkeys.

Transient elevations of transaminases have been reported in patients receiving polatuzumab vedotin and have ranged in intensity from Grade 1–2. These have been reversible with and without dose modification. Two patients treated with polatuzumab vedotin have experienced serious adverse events, including fatty infiltration of the liver (steatosis) in one patient and hepatic cytolysis in the other. Both were deemed “related” to polatuzumab vedotin and both patients fully recovered following discontinuation of the polatuzumab vedotin.

Carcinogenicity

Polatuzumab vedotin may have carcinogenic potential given the mechanism of action of MMAE, the cytotoxic component of polatuzumab vedotin. Myelodysplatic syndrome and other second malignancies have been reported in Phase I and II clinical studies with polatuzumab vedotin. The majority of these patients had received multiple prior lines of anti-cancer therapy, and this was considered as a significant contributory factor.

For polatuzumab vedotin dose delay, modification, and discontinuation instructions, see Table 16.

5.1.4 Risks of Overlapping Toxicities with Mosunetuzumab and Polatuzumab Vedotin

Potential overlapping toxicities between mosunetuzumab and polatuzumab vedotin include thrombocytopenia, neutropenia and infections, TLS, and hepatotoxicity. As such, when these toxicities occur, attribution to a particular agent may be difficult.

As this is the first study combining mosunetuzumab with polatuzumab vedotin, the actual risk of these toxicities is unknown. Treatment hold or discontinuation decisions should in general apply to both agents in response to these toxicities. Refer to Section 5.1.7 for details.

5.1.5 Risks Associated with Rituximab

See the current Rituximab Investigator’s Brochure for full information.

For rituximab dose delay, modification, and discontinuation instructions, see Section 5.1.7.

5.1.6 Risks of Overlapping Toxicities with Rituximab and Polatuzumab Vedotin

Potential overlapping toxicities between rituximab and polatuzumab vedotin include infections, infusion related reactions, neutropenia and TLS. See the current Polatuzumab Vedotin Investigator’s Brochure for additional information for rituximab and polatuzumab combination use. For dose delay, modification, and discontinuation instructions, see Section 5.1.7.

5.1.7 Management of Patients Who Experience Adverse Events

5.1.7.1 Dose Delays and Dose Modifications

Patients should be assessed clinically for toxicity before each dose using NCI CTCAE v5.0 unless otherwise stated. These guidelines pertain to dose delays and modifications based on physical examination findings, observed toxicities, and laboratory results obtained within 72 hours before study treatment administration. Dosing will occur only if a patient's clinical assessment and laboratory test values are acceptable. The determination of all dose and schedule modifications will be made on the basis of the investigator's assessment of ongoing clinical benefit with continuing study treatment in consultation with the Medical Monitor required for mosunetuzumab and polatuzumab vedotin only.

Guidelines for dose delays and modifications of polatuzumab vedotin are shown in Table 15, and Table 16, respectively. Dose delays and dose modifications due to adverse events not specified in Table 16 should proceed on the basis of the principle of maintaining the dose intensity of each treatment regimen.

For patients receiving polatuzumab vedotin, the dose of polatuzumab vedotin may be reduced stepwise to a maximum of one level for management of drug-related toxicities as outlined in Table 15 per the guidelines outlined in Table 16. If further dose reduction is indicated after one dose reduction, the patient must discontinue polatuzumab vedotin but may continue treatment with the remaining study drugs at the investigator's discretion in consultation with the Medical Monitor.

Dose reductions of mosunetuzumab are allowed, but only following dose holds as described in Table 16, Table 18, Table 21 and Table 22. The dose of mosunetuzumab may be reduced to a lower dose level that has previously been cleared during dose escalation (Section 3.1.2). In the event that a lower dose (DL_3 test dose) of mosunetuzumab has not been tested, a dose reduction of 25% is recommended.

No dose modifications for rituximab (375 mg/m^2) are allowed.

Guidance for treatment interruptions for adverse events is provided in Table 16 and Section 5.1.7.2. In general, if one study drug is delayed, the administration of the other study drugs should be delayed for the same time frame (i.e., all study drugs should be delayed for the same time frame so that they are all given together beginning on D1 of the same cycle). Exceptions may occur to maintain the schedule of mosunetuzumab step-up (see Section 5.1.7.2).

Table 15 Steps of Dose Reduction for Polatuzumab Vedotin

Dose Level	Polatuzumab Vedotin
Starting dose	1.8 mg/kg per cycle
First dose reduction	1.4 mg/kg per cycle
Second dose reduction	Discontinue drug

Table 16 Guidelines for Dose Delay, Modification or Discontinuation of Mosunetuzumab [b], Polatuzumab Vedotin and Rituximab

Event(s)	Dose Delay or Modification
Grade 3 or 4 neutropenia with or without infection or fever [a]<br>First occurrence	Delay study treatment for a maximum of 14 days.<br>Growth factors (e.g., G-CSF) for neutropenia are permitted (in addition to primary prophylaxis per Section 4.4.1.1).<br>Patients in the DLT assessment period are not permitted to start the next scheduled cycle of mosunetuzumab until ANC is ≥ 1000/mm3 (see Section 3.1.2.1.2).<br>Dose of mosunetuzumab, polatuzumab vedotin, and rituximab should not be modified for this reason.<br>For patients receiving mosunetuzumab, see Section 5.1.7.1. Consider holding mosunetuzumab for persistent Grade 4 neutropenia (see Section 5.1.2.2, Neutropenia and Thrombocytopenia).
Recurrent Grade 3 or 4 neutropenia	Delay study treatment for a maximum of 14 days.<br>Growth factors (e.g., G-CSF) for neutropenia are permitted (in addition to primary prophylaxis per Section 4.4.1.1).<br>Dose of mosunetuzumab, polatuzumab vedotin, and rituximab should not be modified for this reason.<br>For patients receiving mosunetuzumab, see Section 5.1.7.1. Consider holding mosunetuzumab for persistent Grade 4 neutropenia (see Section 5.1.2.2, Neutropenia and Thrombocytopenia).<br>If Grade 3–4 neutropenia persists despite growth factor support, in the absence of fever, patient may continue study treatment at the investigator's discretion.
Grade 3 or 4 thrombocytopenia<br>First occurrence	Delay study treatment for a maximum of 14 days.<br>If platelet count recovers to ≥ 75,000/μL ≤ Day 7 of the scheduled date of the next cycle, administer full dose of study treatment.<br>Dose of mosunetuzumab polatuzumab vedotin, and rituximab should not be modified for this reason.<br>For patients receiving mosunetuzumab, see Section 5.1.7.1. Consider holding mosunetuzumab for persistent Grade 4 thrombocytopenia (see Section 5.1.2.2, Neutropenia and Thrombocytopenia).

Table 16 Guidelines for Dose Delay, Modification or Discontinuation of Mosunetuzumab [b], Polatuzumab Vedotin and Rituximab (cont.)

Event(s)	Dose Delay or Modification
Recurrent Grade 3 or 4 thrombocytopenia	Delay all study treatment for a maximum of 14 days.<br>If platelet count recovers to ≥75,000/μL ≤Day 7 after the scheduled date of the next cycle, administer full dose of all study drugs.<br>Dose of mosunetuzumab, polatuzumab vedotin, and rituximab should not be modified for this reason.<br>For patients receiving mosunetuzumab, see Section 5.1.7.1. Consider holding mosunetuzumab for persistent Grade 4 thrombocytopenia (see Section 5.1.2.2, Neutropenia and Thrombocytopenia).
Grade 1 neuropathy	No study treatment modification is recommended for Grade 1 sensory or motor peripheral neuropathy.
Grade 2 or 3 peripheral neuropathy (including peripheral sensory or motor neuropathy)	Delay all study treatment.<br>If recovered to Grade ≤1 within ≤14 days of the scheduled date of the next cycle:<br>If the dose of polatuzumab vedotin is 1.8 mg/kg, then reduce polatuzumab vedotin to 1.4 mg/kg (permanent dose reduction). Mosunetuzumab *or rituximab* may be administered at the recommended dose.<br>If there was a prior dose reduction of polatuzumab vedotin to 1.4 mg/kg for Grade 2 or 3 peripheral neuropathy, polatuzumab vedotin must be permanently discontinued. Mosunetuzumab or rituximab may be administered at the recommended dose.<br>If not recovered to Grade ≤1 until >14 days or after the scheduled date for the next cycle, polatuzumab vedotin must be permanently discontinued. Mosunetuzumab *or rituximab* may be administered at the recommended dose.
Grade 4 neuropathy (including peripheral sensory or motor neuropathy)	Discontinue polatuzumab vedotin treatment permanently.<br>Patients should be evaluated regarding the continuation of mosunetuzumab or rituximab on the basis of their benefit–risk.

Table 16 Guidelines for Dose Delay, Modification or Discontinuation of Mosunetuzumab [b], Polatuzumab Vedotin and Rituximab (cont.)

Event(s)	Dose Delay or Modification
Bilirubin > 3.0 mg/dL	*Delay all treatment until resolution to ≤ 1.5 mg/dL within ≤ 14 days. Evaluate for causality.*<br>Dose delay should be avoided if hyperbilirubinemia is not related to hepatic injury (i.e., hemolysis or Gilbert disease). In these cases, dose delay considerations should be guided by direct bilirubin levels.<br>Consider withholding mosunetuzumab (see Section 5.1.2.2, Elevated Liver Enzymes and Hepatotoxicity).
Grade 3 or 4 constipation or ileus	Withold polatuzumab vedotin until improvement to Grade ≤ 2. Mosunetuzumab may be continued or delayed at the discretion of the investigator.<br>Consider reducing polatuzumab vedotin to the next dose level (see in Table 15) after improvement to Grade ≤ 2.
Grade 3 or 4 TLS	Withhold all study treatment. The patient's next dose may be delayed for up to 14 days.<br>Following complete resolution TLS, study treatment may be re-administered at the full dose during next scheduled infusion, in conjunction with prophylactic therapy.<br>For mosunetuzumab-specific TLS guidance, see Section 5.1.2.2, Tumor Lysis Syndrome.
Grade 3 IRR, second episode	Discontinue mosunetuzumab, polatuzumab vedotin or rituximab permanently.<br>If IRR is attributed to polatuzumab vedotin, discontinue polatuzumab vedotin and continue mosunetuzumab or rituximab.<br>If IRR is attributed to mosunetuzumab, discontinue both mosunetuzumab and polatuzumab vedotin, and follow Table 18.<br>If IRR is attributed to rituximab, discontinue rituximab and continue polatuzumab vedotin.

Table 16 Guidelines for Dose Delay, Modification or Discontinuation of Mosunetuzumab [b], Polatuzumab Vedotin and Rituximab (cont.)

Event(s)	Dose Delay or Modification
Grade 3 or 4 non-hematologic toxicity not specifically described above (excluding alopecia, nausea, and vomiting)	Consider delaying all study treatment for a maximum of 14 days.<br>Subsequent recurrence: Based on the nature of the toxicity, decrease polatuzumab vedotin to a lower dose as described in Table 15). There are no dose reductions for mosunetuzumab.<br>Second and subsequent recurrence: Based on the nature of the toxicity and if the event is not clinically manageable and resolving within 14 days of the date of the next scheduled cycle, consider discontinuation of suspect study treatment permanently.
Hepatitis B reactivation (as noted by new detectable HBV-DNA levels)	HBV-DNA levels between WHO-recommended range of 29–100 IU/mL: Re-test within 2 weeks. If still positive, hold all study treatment and treat patient with an appropriate nucleoside analogue. Immediately refer patient to a gastroenterologist or hepatologist.<br>HBV-DNA levels at WHO-recommended cutoff of > 100 IU/mL: Hold all study treatment and treat the patient with an appropriate nucleoside analogue. Immediately refer patient to a gastroenterologist or hepatologist.<br>Rising HBV-DNA viral load (exceeding 100 IU/mL) while on an appropriate anti-viral therapy: Discontinue all study treatment immediately.

G-CSF = granulocyte colony-stimulating factor; HBV = hepatitis B virus; IRR = infusion-related reaction; LVSD = left ventricular systolic dysfunction; TLS = tumor lysis syndrome.

[a] All based on laboratory test results obtained within 72 hours before infusion of Day 1 of that cycle.

[b-] *For patients receiving mosunetuzumab via SC injection, please refer to Section 5.1.7.2 for additional details on repeat step up dosing in the event of a dose delay.*

5.1.7.2 Treatment Interruption

If scheduled dosing coincides with a holiday that precludes dosing, dosing should commence on the nearest following date, with subsequent dosing continuing on a 21 or 28-day schedule as applicable.

Study treatment may be temporarily suspended in patients who experience toxicity considered to be related to study drug (see Table 16). In the event that a patient has a toxicity in C1 necessitating mosunetuzumab interruption for > 7 days, the Medical Monitor should be notified and the patient may be required to repeat mosunetuzumab at the highest dose previously tolerated prior to resuming the planned treatment schedule.

- With the exception of withholding polatuzumab vedotin for neuropathy per Table 16, study drugs withheld for > 14 days because of toxicity considered to be related to study drug should be discontinued, unless resumption of treatment is decided following investigator discussion with the Medical Monitor. Study treatment may continue in the event one or more of the study drugs are discontinued as long as the investigator, in consultation with the Medical Monitor, believes that the patient is continuing to derive clinical benefit from remaining study treatments.

Study treatment may also be temporarily suspended for reasons other than toxicity (e.g., surgical procedures) after discussion with Medical Monitor. The investigator *may consult the* Medical Monitor *to* determine the acceptable length of treatment interruption. Specific guidelines around dosage modifications and interruption for patients who are receiving study treatment and experience toxicities are detailed in Table 16.

Additional guidelines for treatment interruption for specific treatment arms are listed below:

- To maintain the step-up schedule, mosunetuzumab DL_1, DL_2, and first DL_3 test dose should not be held for uncomplicated neutropenia without associated fever or infection or for thrombocytopenia without associated bleeding. Any modification of mosunetuzumab dose schedule should be discussed with the Medical Monitor. For patients on a Group B schedule, if C2D1 polatuzumab vedotin dose is delayed, the mosunetuzumab DL_3 dose may still be administered on C2D1 so that there is no delay of step-up. Polatuzumab vedotin should then be administered during C2, and C3 should start 21 (±2) days after the administration of C2 polatuzumab vedotin.
- If the administration of polatuzumab vedotin is delayed, mosunetuzumab or rituximab should be delayed for the same time frame so that they are given together on D1 of the same cycle.
- *For patients receiving mosunetuzumab via IV administration, if dose delay results in a treatment-free interval of 6 weeks or longer (≥6 weeks), repeat the mosunetuzumab C1 step-up dosing (along with the same C1 step-up dosing assessments; Appendix 1) prior to resuming the planned cycle treatment schedule. If dose delay results in a treatment-free interval of <6 weeks, administer*

mosunetuzumab with the planned cycle treatment schedule; no repeat mosunetuzumab step-up is needed.

> *In the event that a patient has a toxicity in Cycle 1 necessitating mosunetuzumab interruption for >7 days, the Medical Monitor should be notified and the patient may be required to repeat mosunetuzumab at the highest dose previously tolerated prior to resuming the planned treatment schedule.*

- *For patients receiving mosunetuzumab via SC injection, if dose delay results in a treatment-free interval of 6 weeks or longer (≥6 weeks), step-up dosing of mosunetuzumab is required with 5 mg administered on Day 1 of the first cycle after the dose delay, followed by the next planned dose on Day 8 (along with the same schedule of assessments for those dosing days; refer to Appendix 1). Corticosteroid prophylaxis should be administered on both days to mitigate CRS risks. If dose delay results in a treatment-free interval of <6 weeks, administer mosunetuzumab with the planned cycle treatment schedule; no repeat mosunetuzumab step up is needed.*

  > *In the event that a patient has a toxicity necessitating mosunetuzumab interruption for >7 days prior to Day 8 of Cycle 1 dose, the patient is required to repeat the 5 mg dose prior to resuming the planned Cycle 1 treatment schedule (7 days after the administration of the 5 mg dose). Corticosteroid prophylaxis should be administered on both days to mitigate CRS risks. For treatment interruptions after C1D8 or C1D15, treatment should be continued without repeating step-up dosing, unless the treatment-free interval is longer than 6 weeks (see above).*

- In general, patients who experience a Grade 4 non-laboratory abnormality study treatment adverse event should discontinue all study treatment and may not be re-treated. Exceptions may be warranted after discussion with the Medical Monitor taking into consideration the benefit-risk ratio for a given individual patient and/or taking into consideration ad-hoc and patient-specific risk mitigations. For guidance on further treatment in patients who experience Grade 4 CRS, refer to Section 5.1.7.5 and Table 17. Another exception to this relates to TLS. Because TLS represents a pharmacodynamic effect of study treatment that may result in clinical benefit, patients who experience Grade 4 TLS may be considered to continue in the study. In order to be considered for subsequent study treatment, all toxicities and laboratory abnormalities related to TLS should be resolved within 2 weeks. Patients must be hospitalized for TLS prophylaxis and monitoring with the next study treatment dose (see Section 5.1.1 and Section 5.1.2.2, Tumor Lysis Syndrome). For other Grade 4 non-laboratory abnormality adverse events, the investigator may consult with the Medical Monitor for continuation of study treatment.

5.1.7.3 Schedule Modifications

For patients in the Phase II portion of the study with DLBCL, FL, or MCL who have received C1 treatment may have the dosing schedule changed to a 28-day cycle if it is considered by the investigator that changing a patient’s dosing regimen from 21–28-day

cycles would provide sufficient time for recovery from a transient and reversible toxicity (e.g., cytopenia without requiring repeated treatment delays). Modifications of this type to the dosing schedule must be made in consultation with the Medical Monitor.

5.1.7.4 Infusion-Related Reactions and Anaphylaxis

Medications, including epinephrine for SC injections, corticosteroids, diphenhydramine hydrochloride for IV injection, and resuscitation equipment should be available for immediate use. Guidelines for management of infusion-related symptoms for polatuzumab vedotin or rituximab are provided in Table 17 and for mosunetuzumab in Table 18. Also**,** refer to see Table 16 for dose modifications of study treatment regimen.

In the event of a life-threatening IRR (which may include pulmonary or cardiac events) or IgE-mediated anaphylactic reaction, mosunetuzumab, polatuzumab vedotin, and rituximab should be discontinued and no additional drug should be administered. Patients who experience any of these reactions should receive aggressive symptomatic treatment and will be discontinued from study treatment. See Appendix 10 for recommended management of anaphylaxis.

Table 17 Management of Infusion-Related Symptoms for Polatuzumab Vedotin or Rituximab

Infusion-Related Symptoms	Guidance
Grade 1–2	• Slow or hold infusion.<br>• Give supportive treatment. [a]<br>• Upon symptom resolution, may resume infusion-rate escalation at the investigator's discretion.<br>• Note: For Grade 2 wheezing or urticara, patient must be premedicated for any subsequent doses. If symptoms recur, stop the infusion immediately and permanently discontinue study drug.
Grade 3	• Discontinue infusion.<br>• Give supportive treatment. [a]<br>• Upon symptom resolution, may resume infusion-rate escalation, at investigator's discretion. [b]<br>• Note: If the same adverse event recurs with same severity, treatment must be permanently discontinued.<br>• Note: For Grade 3 hypotension or fever, patient must be premedicated before re-treatment. If symptoms recur, then study drug must be permanently discontinued.<br>• Note: If patient has Grade 3 wheezing, bronchospasm, or generalized urticaria at first occurrence, permanently discontinue study drug.
Grade 4	• Discontinue infusion immediately, treat symptoms aggressively, and permanently discontinue study drug.

NCI CTCAE v5.0 = National Cancer Institute Common Terminology Criteria for Adverse Events, Version 5.0.

Refer to the NCI CTCAE v5.0 for the grading of symptoms. Management of IgE-mediated allergic reactions should be as directed in the text following this table.

[a] Supportive treatment: Patients should be treated with acetaminophen/paracetamol and an antihistamine such as diphenhydramine if they have not been received in the previous 4 hours. IV saline may be indicated. For bronchospasm, urticaria, or dyspnea, patients may require antihistamines, oxygen, corticosteroids (e.g., 100 mg IV prednisolone or equivalent), and/or bronchodilators. Patients with hypotension who require vasopressor support must be permanently discontinued from study drug.

[b] Infusion rate escalation after re-initiation: Upon complete resolution of symptoms, the infusion may be resumed at 50% of the rate achieved prior to interruption. In the absence of infusion-related symptoms, the rate of infusion may be escalated in increments of 50 mg/hr every 30 minutes.

5.1.7.5 Cytokine Release Syndrome

Guidelines for CRS apply for patients receiving mosunetuzumab. Given the mechanism of action of mosunetuzumab, CRS and IRRs during or after infusion of mosunetuzumab may be clinically indistinguishable from each other, and their recommended treatment is the same. CRS resolution is defined as the patient no longer requiring vasopressor support or oxygen supplementation.

Table 18 Management of Cytokine Release Syndrome for Patients Receiving Mosunetuzumab (IV and SC)

CRS Grade [a]	Action with Current Mosunetuzumab IV	Supportive Care	Anti-IL6/Corticosteroid Therapy	Action for Next Mosunetuzumab Dose
**Grade 1**<br>Fever ≥ 38ºC	• Slow infusion to ≤ 50% or interrupt infusion until symptoms resolve; re-start at same rate.<br>• If symptoms recur with rechallenge, interrupt study treatment, do not resume, and manage per Grade 2.	• Symptomatic management of constitutional symptoms and organ toxicities.<br>• Consider empiric broad-spectrum antibiotics.<br>• Consider G-CSF if neutropenic.<br>• Maintenance IV fluids for hydration.<br>• Consider hospitalization until symptoms completely resolve.	• For prolonged CRS (> 2 days) in patients with significant symptoms and/or comorbidities (per investigator discretion, e.g., impaired cardiovascular function, reduced pulmonary reserve), consider tocilizumab and corticosteroids as per Grade 2.	• Administer premedications for next dose per Section 4.3.2.1.<br>• For IV mosunetuzumab, consider 50% (or lower) rate of infusion for next step-up dose in C1 or 50% rate of infusion if next dose is same dose level (beyond C1).<br>• Consider enhanced premedications for next dose.<br>• Consider hospitalization for next dose.
**Grade 2**<br>Fever ≥ 38ºC with hypotension not requiring vasopressors and/or hypoxia requiring low-flow oxygen [b] by nasal cannula or blow-by	• Hold further study treatment until symptoms resolved; consider re-starting infusion at 50% rate.<br>• If symptoms recur with rechallenge at decreased infusion rate, interrupt study treatment, do not resume, and manage per Grade 3.	• Symptomatic management of constitutional symptoms and organ toxicities.<br>• Consider ICU admission for hemodynamic monitoring.	• Consider tocilizumab. [c]<br>• For persistent refractory hypotension after 1–2 doses of anti-IL6 therapy, consider dexamethasone 10 mg IV every 6 hours (or equivalent).	• May receive the next dose of mosunetuzumab if symptoms resolve to Grade ≤ 1 for 3 consecutive days.<br>• Consider enhanced premedications for next dose.<br>• For IV mosunetuzumab, consider 50% (or lower) rate of infusion for next step-up dose in C1 or 50% rate of infusion if next dose is same dose level (beyond C1).

Table 18 Management of Cytokine Release Syndrome for Patients Receiving Mosunetuzumab (IV and SC) (cont.)

CRS Grade [a]	Action with Current Mosunetuzumab IV	Supportive Care	Anti-IL6/Corticosteroid Therapy	Action for Next Mosunetuzumab Dose
**Grade 2 (cont.)**<br>Fever ≥ 38ºC with hypotension not requiring vasopressors and/or hypoxia requiring low-flow oxygen [b] by nasal cannula or blow-by		For hypotension: IV fluid bolus as needed; for persistent refractory hypotension (e.g., after two fluid boluses and anti-IL6 therapy), start vasopressors and manage per Grade 3.<br>Rule out other inflammatory conditions which can mimic severe CRS (e.g., infections/ sepsis).<br>Consider empiric broad-spectrum antibiotics.<br>If no improvement within 24 hours, initiate work up and assess for signs and symptoms of MAS/HLH as described in Section 5.1.7.5.	Manage per Grade 3 if no improvement within 24 hours after starting tocilizumab.	Consider hospitalization for next dose.

Table 18 Management of Cytokine Release Syndrome for Patients Receiving Mosunetuzumab (IV and SC) (cont.)

CRS Grade [a]	Action with Current Mosunetuzumab IV	Supportive Care	Anti-IL6/Corticosteroid Therapy	Action for Next Mosunetuzumab Dose
**Grade 3**<br>Fever ≥ 38ºC with hypotension requiring a vasopressor (with or without vasopressin) and/or hypoxia requiring high flow oxygen by nasal cannula, face mask, non-rebreather mask, or Venturi mask	Stop infusion, do not resume.	Symptomatic management of organ toxicities, admit to ICU for hemodynamic monitoring.<br>For hypotension: IV fluid bolus and vasopressors as needed.<br>Rule out other inflammatory conditions which can mimic severe CRS (e.g., infections/ sepsis).<br>Consider empiric broad-spectrum antibiotics.<br>If no improvement within 24 hours, initiate work up and assess for signs and symptoms of MAS/HLH as described in Section 5.1.7.5.	Administer tocilizumab[c]<br>Dexamethasone 10 mg IV every 6 hours (or equivalent). If refractory, manage as per Grade 4. [c]<br>Manage per Grade 4 if no improvement within 18–24 hours after second dose of tocilizumab.	• May receive the next dose of mosunetuzumab if CRS event was responsive to treatment (i.e., clinical improvement within 8–12 hours following tocilizumab/corticosteroids administration) and symptoms resolve to Grade ≤ 1 for 3 consecutive days:<br>Enhanced premedications for next dose<br>For mosunetuzumab IV, decrease to 50% (or lower) rate of infusion for next step-up dose in C1, or 50% rate of infusion if next dose is same dose level (beyond C1)<br>Hospitalization for next dose<br>For mosunetuzumab IV, the next dose should be reduced to the next lower dose level that has been previously cleared during dose escalation. [g]

Table 18 Management of Cytokine Release Syndrome for Patients Receiving Mosunetuzumab (IV and SC) (cont.)

CRS Grade [a]	Action with Current Mosunetuzumab IV	Supportive Care	Anti-IL6/Corticosteroid Therapy	Action for Next Mosunetuzumab Dose
**Grade 3 (cont.)**<br>Fever ≥ 38ºC with hypotension requiring a vasopressor (with or without vasopressin) and/or hypoxia requiring high flow oxygen by nasal cannula, face mask, non-rebreather mask, or Venturi mask				If the reduced IV dose is tolerated with no signs/symptoms of Grade 3 or higher CRS, the patient may return to the next higher dose that has been previously cleared during dose escalation.<br>• For mosunetuzumab SC, if Grade 3 occurs after the C1D1 5 mg injection, the next dose should be 5mg. If the next dose is tolerated without Grade 3 or higher CRS, the subsequent dose may be increased to 45 mg.<br>• For mosunetuzumab SC, if Grade 3 occurs after a 45 mg injection, the next dose should be 20mg. If the next dose is tolerated without Grade 3 or higher CRS, the subsequent dose may be re-escalated to 45 mg.<br>• If Grade 3 CRS recurs with subsequent doses, permanently discontinue mosunetuzumab. [h]

Table 18 Management of Cytokine Release Syndrome for Patients Receiving Mosunetuzumab (IV and SC) (cont.)

CRS Grade [a]	Action with Current Mosunetuzumab IV	Supportive Care	Anti-IL6/Corticosteroid Therapy	Action for Next Mosunetuzumab Dose
**Grade 4**<br>Fever ≥ 38ºC with hypotension requiring multiple vasopressors (excluding vasopressin) and/or hypoxia requiring oxygen by positive pressure (e.g., CPAP, BiPAP, intubation and mechanical ventilation)	• Stop infusion, do not resume.	• ICU admission and hemodynamic monitoring.<br>• Mechanical ventilation as needed.<br>• IV fluids and vasopressors as needed.<br>• Symptomatic management of organ toxicities.<br>• Rule out other inflammatory conditions which can mimic severe CRS (e.g., Infections/ sepsis)<br>• Consider empiric broad-spectrum antibiotics.<br>• If no improvement within 24 hours, initiate work up and assess for signs and symptoms of MAS/HLH as described in Section 5.1.7.5.	• Administer tocilizumab. [c]<br>• For patients refractory to tocilizumab, consider siltuximab, anakinra, and emapalumab, based on discretion of the investigator; management should be discussed with the Medical Monitor. [d]<br>• Dexamethasone 10 mg IV every 6 hours (or equivalent).<br>• If refractory, consider methylprednisolone 1000 mg/day IV. [e, f]	• Permanently discontinue mosunetuzumab. [h]

Table 18 Management of Cytokine Release Syndrome for Patients Receiving Mosunetuzumab (IV and SC) (cont.)

ASTCT = American Society for Transplantation and Cellular Therapy; BiPAP = bilevel positive airway pressure; CPAP = continuous positive airway pressure; CRS = cytokine release syndrome; C = cycle; D = day; G-CSF = granulocyte colony stimulating factor; HLH = hemophagocytic lymphohistiocytosis; MAS = macrophage activation syndrome.

[a] CRS grading per ASTCT (Lee et al. 2019). Fever is defined as temperature ≥ 38ºC not attributable to any other cause. In patients who have CRS and then receive antipyretic or anticytokine therapy such as tocilizumab or steroids, fever is no longer required to grade subsequent CRS severity. CRS grade is determined by the more severe event: hypotension or hypoxia not attributable to any other cause.

[b] Low-flow nasal cannula is defined as oxygen delivered at ≤ 6 L/minute. Low flow also includes blow-by oxygen delivery. High-flow nasal cannula is defined as oxygen delivered at > 6 L/minute.

[c] Tocilizumab should be administered at a dose of 8 mg/kg IV (8 mg/kg for participants at a weight of ≥ 30 kg only; 12 mg/kg for participants at a weight of < 30 kg; doses exceeding 800 mg per infusion are not recommended); repeat every 8 hours as necessary (up to a maximum of 4 doses). Refer to Appendix 5 for Schedule of Activities for tocilizumab treatment of CRS.

[d] Riegler et al. 2019.

[e] Antifungal prophylaxis should be strongly considered in patients receiving steroids for treatment of CRS.

[f] For example, methylprednisolone IV 1000 mg/day for 3 days, followed by rapid taper at 250 mg every 12 hours for 2 days, 125 mg every 13 hours for 2 days, and 60 mg every 12 hours for 2 days.

[g] If Grade 3 CRS occurs following mosunetuzumab IV administration at Cycle 1 Day 1 or Cycle 1 Day 8, repeat the same step-up dose for the next infusion.

[h] Resumption of mosunetuzumab may be considered in patients who are deriving benefit and have fully recovered from the adverse event. Patients can be re-challenged with mosunetuzumab only at the investigator's discretion if below are confirmed:

Individual risk-benefit assessment by principal investigator/treating physician favors continued treatment;

The patient has recovered from previous toxicities and has sufficient organ function/reserve to receive subsequent doses;

The patient has been adequately consented for risks associated with continued treatment and decides to receive subsequent doses;

The above risk-benefit assessment and evaluation of patients are discussed with the Sponsor;

Subsequent doses are well planned with precautionary measures, including dose reduction, slow infusion rate at 50% or lower, mandatory hospitalizations, and enhanced premedications.

5.1.7.6 Hemophagocytic Lymphohistiocytosis

The supportive management of HLH is generally similar to that of CRS (see Section 5.1.7.5). Specific diagnostic, monitoring, and management guidelines for HLH are described below.

Patients with suspected HLH should be diagnosed according to published criteria by McClain and Eckstein (2019). A patient should be classified as having HLH if five of the following eight criteria are met:

- Fever $\geq$38.5°C
- Splenomegaly
- Peripheral blood cytopenia consisting of at least two of the following:
- Hemoglobin $<$90 g/L (9 g/dL) ($<$100 g/L [10 g/dL] for infants $<$4 weeks old)
- Platelet count $<100 \times 109$/L (100,000/μL)
- ANC $<1.0 \times 109$/L (1000/μL)
- Fasting triglycerides $>$2.992 mmol/L (265 mg/dL) and/or fibrinogen $<$1.5 g/L (150 mg/dL)
- Hemophagocytosis in bone marrow, spleen, lymph node, or liver
- Low or absent natural killer cell activity
- Ferritin $>$ 500 mg/L (500 ng/mL)
- Soluble interleukin 2 (IL-2) receptor (soluble CD25) elevated $\geq$2 standard deviations above age-adjusted laboratory-specific norms

In all cases of suspected HLH, the Medical Monitor should be immediately notified. Patients should be hospitalized with the following diagnostic and monitoring measures initiated:

- Frequent (e.g., every 4 hours) vital signs and physical examination including evaluation for splenomegaly
- Serial (at least daily) monitoring of serum chemistries, complete blood counts, LFTs, LDH, ferritin, PT/PTT, fibrinogen, D-dimer and triglycerides
- Consideration of bone marrow and/or lymph node biopsy to assess for hemophagocytosis and active infection, including assessment of EBV protein localization in T/B/NK cells
- Complete infectious disease workup including:
  - Blood cultures (bacterial and fungal)
  - Urine cultures and urinalysis
  - Radiographic assessments (e.g., chest X-ray or CT scan)
  - Assessment for active viral infections, including, but not limited to, EBV and CMV
- If available, assessment for soluble CD25 and assessment of NK cell function

- DNA for exploratory genetic testing of mutations potentially associated with HLH, (e.g., PRF1, MUNC13-4, STXBP2 should be considered [Zhang et al. 2011])

Patients with suspected HLH should be treated according to the guidelines in Table 19. In the case of confirmed HLH, permanently discontinue study treatment.

Table 19 Management Guidelines for Suspected and Confirmed Hemophagocytic Lymphohistiocytosis

Event	Management
Suspected HLH	Withhold study treatment and contact Medical Monitor.<br>Consider patient referral to hematologist.<br>Initiate supportive care, including intensive care monitoring if indicated per institutional guidelines.<br>Consider treatment for HLH with appropriate therapy
Confirmed HLH	Permanently discontinue study treatment and contact the Medical Monitor.<br>Refer patient to a hematologist<br>Institute appropriate supportive care, including intensive care monitoring, if indicated per the institutional guidelines<br>Treat with appropriate HLH therapy according to institutional standards or published references (Schram and Berliner 2015; Vallurupalli and Berliner 2019)

HLH = hemophagocytic lymphohistiocytosis.

5.1.7.7 Injection-Site Reactions after SC Administration of Mosunetuzumab

Refer to Section 5.3.5.2 for adverse event reporting procedures related to injection-site reactions. Patients who experience localized injection-site reactions following SC administration of mosunetuzumab should be managed according to the guidelines detailed in Table 20. *Should an individual patient at any time develop unacceptable localized injection-site reactions following SC administration of mosunetuzumab, conversion to IV mosunetuzumab administration may be considered following discussion with the Medical Monitor.*

Table 20 Management Guidelines for Injection-Site Reactions

Grade	Management
Grade 1	• Consider treatment with topical steroids.<br>• Continue mosunetuzumab in subsequent cycles.
Grade 2	• Notify Medical Monitor.<br>• Initiate treatment with topical steroids.<br>• If progressive after 24 hours, consider prednisone or equivalent 10–30 mg/day.<br>• Continue mosunetuzumab in subsequent cycles after improvement to Grade ≤ 1.

Table 20 Management Guidelines for Injection-Site Reactions (cont.)

Grade	Management
Grade 3	• Notify Medical Monitor.<br>• Withhold mosunetuzumab.<br>• Initiate prednisone 1 mg/kg/day or equivalent.<br>• Consult dermatology.<br>• Taper steroids after improvement to Grade ≤ 1.<br>• Continue mosunetuzumab in subsequent cycles after improvement to Grade ≤ 1.
Grade 4	• Notify Medical Monitor.<br>• Management as for Grade 3.<br>• Permanently discontinue SC mosunetuzumab.<br>• Consider continuing study treatment with mosunetuzumab IV.

5.1.7.8 Tumor Lysis Syndrome

Treatment for laboratory and or clinical presentations of TLS will follow institutional practice. Prophylaxis of TLS should follow local institutional practice; however, for patients receiving mosunetuzumab, the following measures must be implemented.

All patients should receive prophylaxis for TLS prior to each mosunetuzumab administration during Cycles 1 and 2. Prophylaxis guidelines include the following:

- Hydration, consisting of a fluid intake of approximately 2–3 L/day starting 24–48 hours prior to the first dose of mosunetuzumab
  - If a patient is hospitalized for the administration of study treatment, IV hydration at a rate of 150–200 mL/hr should begin at the conclusion of mosunetuzumab administration and continue for at least 24 hours thereafter.
  - If a patient receives study treatment in the outpatient setting, fluid intake should be maintained at 2–3 L/day for at least 24 hours after mosunetuzumab administration.
  - Modification of fluid rate should be considered for individuals with specific medical needs.
- Administration of an agent to reduce uric acid *should be considered*:
  - Allopurinol (e.g., 300 mg/day orally beginning 72 hours prior to dose and continuing for 3–7 days afterward) should be administered for those patients judged to be of low or intermediate risk of developing TLS, per investigator's discretion.
  - For patients with elevated uric acid levels prior to mosunetuzumab treatment or considered to be at high risk for TLS: Rasburicase (e.g., 0.2 mg/kg IV over 30 minutes prior to first dose mosunetuzumab and daily for up to 5 days thereafter) should be administered, unless contraindicated (Elitek® USPI).

  - Treatment with allopurinol/rasburicase should continue as specified above, or if laboratory evidence of TLS is observed until normalization of serum uric acid or other laboratory parameters.
  - If treatment with allopurinol or rasburicase is contraindicated or is otherwise inappropriate in the view of the investigator, the Medical Monitor should be contacted for further guidance.
- Laboratory monitoring for tumor lysis is described in the schedule of activities (serum chemistry, creatinine, and LDH; see Appendix 1 and Appendix 15).
- Note that uric acid measurement in the presence of rasburicase administration requires special handling (Elitek USPI).
- Telemetry should be considered for patients at high risk for TLS; therefore, patients should be considered for inpatient monitoring following any mosunetuzumab administration given in the first 2 cycles, in addition to required hospitalization described in Section 5.1.1.

Due to the risk of TLS following mosunetuzumab and polatuzumab vedotin administration, patients must have creatinine clearance ≥50 mL/min to participate in this trial (Section 4.1.1). Laboratory results should be reviewed and electrolyte values should not demonstrate any clinically significant abnormalities prior to the administration of mosunetuzumab in C1 and beyond, otherwise the patient should receive additional prophylactic treatment and hydration prior to the initiation of dosing. Laboratory abnormalities suggestive of TLS should prompt immediate action by the treating clinicians, and TLS should be treated aggressively per institutional practice.

Patients at high risk for TLS should continue to receive prophylaxis with allopurinol or rasburicase and adequate hydration with each subsequent dose of mosunetuzumab until the patient is no longer considered to be at risk for TLS. Patients who develop either clinical or laboratory TLS during C1 (or C2 for Group C) should be considered for hospitalization (i.e., through at least 72 hours mosunetuzumab after study treatment administration) during subsequent cycles for optimum hydration and monitoring; such cases should be discussed with the Medical Monitor.

5.1.7.9 Neurological Adverse Events

Guidelines for neurological adverse events apply for patients receiving mosunetuzumab (Table 21). For patients developing peripheral neuropathy, refer to Table 16.
For patients receiving both polatuzumab vedotin and mosunetuzumab who develop Grade ≥2 peripheral sensory neuropathy and/or peripheral motor neuropathy, consideration for holding or discontinuing mosunetuzumab in addition to action taken with polatuzumab vedotin should be made and discussed with the Medical Monitor.

Table 21 Management Guidelines for Neurological Adverse Events for Patients Receiving Mosunetuzumab

Event	Grade	Management
Seizure	Grade 1–2	• Withhold further study treatment, provide supportive care.<br>• Consider treatment with corticosteroids.<br>• Consider consultation with a neurologist; consider brain MRI (with diffusion-weighted imaging), lumbar puncture, EEG.<br>• Study treatment may be resumed after consultation with the Medical Monitor if no recurrent seizure for at least 3 days and with confirmation of baseline neurologic examination. [a]<br>• Consider dose reduction of mosunetuzumab when resuming.
	Grade 3–4	• Permanently discontinue study treatment.<br>• Consider treatment with corticosteroids.<br>• Obtain neurology consultation.
Neurologic events not otherwise specified	Grade 1	• Notify Medical Monitor.<br>• Consider withholding study treatment during evaluation.
	Grade 2	• Notify Medical Monitor.<br>• Withhold mosunetuzumab and evaluate etiology. Consider imaging as appropriate.<br>• Consider treatment with corticosteroids.<br>• Consider neurology consultation.<br>• Study treatment may be resumed when symptoms have returned to baseline $\geq$3 consecutive days without the need for medical management and with confirmation of baseline neurologic examination. [a]
	Grade 3	• Notify Medical Monitor.<br>• Withhold mosunetuzumab and evaluate etiology. Consider imaging as appropriate.<br>• Consider treatment with corticosteroids.<br>• Obtain neurology consultation.<br>• Consider discontinuation mosunetuzumab if symptoms persist $>$ 7 days. [a]<br>• Mosunetuzumab may be resumed when symptoms have returned to baseline $\geq$3 consecutive days without the need for medical management and with baseline neurologic examination.<br>• Permanently discontinue study treatment for recurrent Grade 3 event.

Table 21 Management Guidelines for Neurological Adverse Events for Patients Receiving Mosunetuzumab (cont.)

Event	Grade	Management
Neurologic events not otherwise specified	Grade 4	• Notify Medical Monitor.<br>• Permanently discontinue study treatment.<br>• Obtain neurology consultation.

MRI = magnetic resonance imaging.

[a] The overall benefit–risk of continued treatment with mosunetuzumab should be assessed by the study investigator in consultation with the Medical Monitor.

5.1.7.10 Elevated Liver Enzymes and Hepatotoxicity

Guidelines for hepatotoxicity in Table 22 apply for patients receiving mosunetuzumab.

For patients with isolated elevated bilirubin, refer to Table 16.

For patients receiving both polatuzumab vedotin and mosunetuzumab who develop isolated elevated bilirubin, consideration for withholding or discontinuing mosunetuzumab in addition to action taken with polatuzumab vedotin should be made and discussed with the Medical Monitor. Similarly, for patients developing elevated liver enzymes with or without elevated bilirubin, considerations for withholding or discontinuing polatuzumab vedotin should be made and discussed with the Medical Monitor.

Transient Grade 3 AST and ALT elevations have been observed with mosunetuzumab in the setting of CRS and have resolved with supportive treatment. HLH (Section 5.1.2.2) may present as acute liver failure (Lin et al. 2016; Jagtap et al. 2017). In instances where no alternative etiology (e.g. viral, neoplastic) is identified, an immune mediated cause should be considered and evaluated.

Table 22 Management Guidelines for Liver Function Test Abnormalities and Hepatic Events for Patients Receiving Mosunetuzumab

LFT Abnormality	Management
Grade 1 AST or ALT elevation	Continue mosunetuzumab.<br>Notify Medical Monitor and monitor LFTs (including AST, ALT, and bilirubin) weekly.
Grade 2 AST or ALT elevation	**All events:**<br>Withhold mosunetuzumab.<br>Monitor LFTs at least weekly and as clinically indicated until values resolve to normal or baseline.<br>Resume mosunetuzumab when resolved to Grade ≤ 1 or baseline.<br>Consider hepatology consultation.<br>**Events > 5 days' duration:**<br>Obtain hepatology consultation; evaluate etiology.
Grade 3 AST or ALT elevation	**All events:**<br>Withhold mosunetuzumab<br>Monitor LFTs every 24–48 hours until decreasing, and then follow weekly.<br>Obtain hepatology consultation; consider liver biopsy to assess hepatic injury. [a]<br>Resume mosunetuzumab when resolved to Grade ≤ 1 or baseline.

Table 22 Management Guidelines for Liver Function Test Abnormalities and Hepatic Events for Patients Receiving Mosunetuzumab (cont.)

LFT Abnormality	Management
Grade 3 AST or ALT elevation	**Events > 5 days' duration:**<br>Resume mosunetuzumab when resolved to Grade ≤ 1 or baseline[a]
Grade 4 AST or ALT elevation	Permanently discontinue mosunetuzumab. [b]<br>Follow management guidelines as described for Grade 3 events.

CRS = cytokine release syndrome; LFT = liver function test; HLH = hemophagocytic lymphohistiocytosis.

[a] Immune-related event should be considered when concurrent clinical and laboratory manifestations of CRS (Section 5.1.2.1) HLH (Section 5.1.2.2) are present, or in instances where no alternative etiology (e.g. viral, neoplastic) can account for observed LFT abnormalities.

[b] Resumption of mosunetuzumab may be considered in patients who are deriving benefit and who have fully recovered from the immune-related event. Patients may resume dosing with mosunetuzumab only after discussion with the investigator and the Medical Monitor.

5.1.7.11 Neutropenia and Thrombocytopenia in Patients Receiving Mosunetuzumab

Treatment delays for polatuzumab for neutropenia are described in Table 16.

For patients receiving both polatuzumab vedotin and mosunetuzumab who develop persistent Grade 4 neutropenia or thrombocytopenia, consideration for withholding or discontinuing mosunetuzumab in addition to action taken with polatuzumab vedotin should be made and discussed with the Medical Monitor. Mosunetuzumab step doses should not be held for uncomplicated neutropenia without associated fever or for thrombocytopenia without associated bleeding in C1. Any modification of mosunetuzumab dose schedule should be discussed with the Medical Monitor. See also Section 5.1.7.2.

5.1.8 Internal Monitoring Committee

Because this is the first trial to combine mosunetuzumab with polatuzumab vedotin, an IMC will be utilized during the study to make recommendations regarding study conduct on the basis of trial safety data to ensure enhanced patient safety while receiving study treatment.

The IMC will be established to monitor patient safety throughout the study and make decisions regarding dose escalations based on the plan described in Section 3.1.2.

The IMC will make recommendations about whether to continue mandatory hospitalizations with specific study treatment regimens and about the use of G-CSF and other supportive care measures. The IMC will also make a recommendation about whether to continue into the expansion phase of the study after completion of the dose-finding phase, the Arm K safety run-in, the first 10 patients who have completed at

least one cycle in Arm L, or at any interim safety analysis of Arms I, J, and K. The IMC will also make a recommendation about the mosunetuzumab dose and schedule in combination with polatuzumab vedotin to be used in the expansion phases for Arms I and J.

The IMC will consist of an IMC Medical Monitor Chair who is not associated with the project, and representatives from Clinical Science, Safety Science, and Biostatistics who are all external to the study team.

In addition to the ongoing assessment of the incidence and nature of toxicity, adverse events (particularly Grades ≥3), serious adverse events, deaths, and laboratory abnormalities by the investigator and the Medical Monitor, the IMC will review all necessary cumulative data at regular intervals during the study. At the time of each review, the IMC may make recommendations regarding study conduct, including but not limited to, the following: performing additional safety analyses, amending the study protocol, holding patient enrollment pending further safety evaluations, enrolling additional patients at a specific dose level and schedule to obtain additional safety data, holding/discontinuing study treatment, making decisions to modify or discontinue the requirement for hospitalization with study treatment, or terminating the study. Decisions will be made in consideration of the totality of the available data. Ad hoc meetings may be called in addition to scheduled meetings, as necessary, to provide recommendations on management of any new safety issues. Specific operational details such as the committee's composition, frequency and timing of meetings, and members' roles and responsibilities will be detailed in an IMC Charter.

5.2 SAFETY PARAMETERS AND DEFINITIONS

Safety assessments will consist of monitoring and recording adverse events, including serious adverse events and adverse events of special interest, performing protocol-specified safety laboratory assessments, measuring protocol-specified vital signs, and conducting other protocol-specified tests that are deemed critical to the safety evaluation of the study.

Certain types of events require immediate reporting to the Sponsor, as outlined in Section 5.4.

5.2.1 Adverse Events

According to the ICH guideline for Good Clinical Practice, an adverse event is any untoward medical occurrence in a clinical investigation subject administered a pharmaceutical product, regardless of causal attribution. An adverse event can therefore be any of the following:

- Any unfavorable and unintended sign (including an abnormal laboratory finding), symptom, or disease temporally associated with the use of a medicinal product, whether or not considered related to the medicinal product

- Any new disease or exacerbation of an existing disease (a worsening in the character, frequency, or severity of a known condition) (see Sections 5.3.5.11 and 5.3.5.12 for more information)
- Recurrence of an intermittent medical condition (e.g., headache) not present at baseline
- Any deterioration in a laboratory value or other clinical test (e.g., ECG, X-ray) that is associated with symptoms or leads to a change in study treatment or concomitant treatment or discontinuation from study drug
- Adverse events that are related to a protocol-mandated intervention, including those that occur prior to assignment of study treatment (e.g., screening invasive procedures such as biopsies)

5.2.2 Serious Adverse Events (Immediately Reportable to the Sponsor)

A serious adverse event is any adverse event that meets any of the following criteria:

- Is fatal (i.e., the adverse event actually causes or leads to death)
- Is life threatening (i.e., the adverse event, in the view of the investigator, places the patient at immediate risk of death)
  - This does not include any adverse event that, had it occurred in a more severe form or was allowed to continue, might have caused death.
- Requires or prolongs inpatient hospitalization (see Section 5.3.5.13)
- Results in persistent or significant disability/incapacity (i.e., the adverse event results in substantial disruption of the patient's ability to conduct normal life functions)
- Is a congenital anomaly/birth defect in a neonate/infant born to a mother exposed to study drug
- Is a significant medical event in the investigator's judgment (e.g., may jeopardize the patient or may require medical/surgical intervention to prevent one of the outcomes listed above)

The terms "severe" and "serious" are not synonymous. Severity refers to the intensity of an adverse event (e.g., rated as mild, moderate, or severe, or according to National Cancer Institute Common Terminology Criteria for Adverse Events [NCI CTCAE]; see Section 5.3.3); the event itself may be of relatively minor medical significance (such as severe headache without any further findings).

Severity and seriousness need to be independently assessed for each adverse event recorded on the eCRF.

Serious adverse events are required to be reported by the investigator to the Sponsor immediately (i.e., no more than 24 hours after learning of the event; see Section 5.4.2 for reporting instructions).

5.2.3 Adverse Events of Special Interest (Immediately Reportable to the Sponsor)

Adverse events of special interest are required to be reported by the investigator to the Sponsor immediately (i.e., no more than 24 hours after learning of the event; see Section 5.4.2 for reporting instructions). Adverse events of special interest for this study are as follows:

- Cases of potential drug-induced liver injury that include an elevated ALT or AST in combination with either an elevated bilirubin or clinical jaundice, as defined by Hy's Law (see Section 5.3.5.9)
- Suspected transmission of an infectious agent by the study drug, as defined below
  - Any organism, virus, or infectious particle (e.g., prion protein transmitting transmissible spongiform encephalopathy), pathogenic or non-pathogenic, is considered an infectious agent. A transmission of an infectious agent may be suspected from clinical symptoms or laboratory findings that indicate an infection in a patient exposed to a medicinal product. This term applies only when a contamination of the study drug is suspected.
- Adverse events of special interest specific for mosunetuzumab:
  - Grade $\geq$2 CRS
  - Grade $\geq$2 neurologic adverse event
  - Any suspected MAS/HLH
  - Grade $\geq$2 injection-site reactions
  - TLS (minimum Grade 3 by definition)
  - Febrile neutropenia (minimum Grade 3 by definition)
  - Any grade disseminated intravascular coagulation (minimum Grade 2 by definition)
  - Grade $\geq$2 AST, ALT, or total bilirubin elevation

- Grade ≥2 tumor flare, e.g. manifestation of signs/symptoms associated with increase in size of known nodal or extranodal lesions by clinical or radiographic assessment, new onset or worsening of pre-existing pleural effusions; refer to Section 5.1.2.2 and the Mosunetuzumab Investigator's Brochure for additional details
- Any grade pneumonitis or interstitial lung disease (excluding pneumonia of infectious eitology)

- Adverse events of special interest specific for polatuzumab vedotin:
  - TLS any grade (irrespective of causality)
  - Second malignancies
  - Grade ≥3 peripheral neuropathy
  - Progressive multifocal leukoencephalopathy
  - Systemic hypersensitivity reactions/anaphylactic and anaphylactoid reactions

5.2.4 Dose-Limiting Toxicities (Immediately Reportable to the Sponsor)

During the DLT assessment window, adverse events identified as DLTs, as defined in Section 3.1.2.1, are required to be reported by the investigator to the Sponsor immediately (i.e., no more than 24 hours after learning of the event; see Section 5.4.2 for reporting instructions).

5.3 METHODS AND TIMING FOR CAPTURING AND ASSESSING SAFETY PARAMETERS

The investigator is responsible for ensuring that all adverse events (see Section 5.2.1 for definition) are recorded on the Adverse Event eCRF and reported to the Sponsor in accordance with instructions provided in this section and in Sections 5.4–5.6.

For each adverse event recorded on the Adverse Event eCRF, the investigator will make an assessment of seriousness (see Section 5.2.2 for seriousness criteria), severity (see Section 5.3.3), and causality (see Section 5.3.4).

5.3.1 Adverse Event Reporting Period

Investigators will seek information on adverse events at each patient contact.
All adverse events, whether reported by the patient or noted by study personnel, will be recorded in the patient's medical record and on the Adverse Event eCRF.

After informed consent has been obtained but prior to initiation of study drug, only serious adverse events caused by a protocol-mandated intervention (e.g., invasive procedures such as biopsies, discontinuation of medications) should be reported (see Section 5.4.2 for instructions for reporting serious adverse events).

After initiation of study drug, all adverse events will be reported until 90 days after the last dose of study drug or the initiation of *next anti-lymphoma treatment*, whichever is earlier.

Instructions for reporting adverse events that occur after the adverse event reporting period are provided in Section 5.6.

5.3.2 Eliciting Adverse Event Information

A consistent methodology of non-directive questioning should be adopted for eliciting adverse event information at all patient evaluation timepoints. Examples of non-directive questions include the following:

"How have you felt since your last clinic visit?"

"Have you had any new or changed health problems since you were last here?"

5.3.3 Assessment of Severity of Adverse Events

The adverse event severity grading scale for the NCI CTCAE (v5.0) will be used for assessing adverse event severity, except grading of CRS will follow the ASTCT CRS Consensus Grading criteria described in Appendix 8. Table 23 will be used for assessing severity for adverse events that are not specifically listed in the NCI CTCAE.

Table 23 Adverse Event Severity Grading Scale for Events Not Specifically Listed in NCI CTCAE

Grade	Severity
1	Mild; asymptomatic or mild symptoms; clinical or diagnostic observations only; or intervention not indicated
2	Moderate; minimal, local, or non-invasive intervention indicated; or limiting age-appropriate instrumental activities of daily living [a]
3	Severe or medically significant, but not immediately life-threatening; hospitalization or prolongation of hospitalization indicated; disabling; or limiting self-care activities of daily living [b, c]
4	Life-threatening consequences or urgent intervention indicated [d]
5	Death related to adverse event [d]

NCI CTCAE = National Cancer Institute Common Terminology Criteria for Adverse Events.

Note: Based on the most recent version of NCI CTCAE (v5.0), which can be found at: http://ctep.cancer.gov/protocolDevelopment/electronic_applications/ctc.htm

[a] Instrumental activities of daily living refer to preparing meals, shopping for groceries or clothes, using the telephone, managing money, etc.

[b] Examples of self-care activities of daily living include bathing, dressing and undressing, feeding oneself, using the toilet, and taking medications, as performed by patients who are not bedridden.

[c] If an event is assessed as a "significant medical event," it must be reported as a serious adverse event (see Section 5.4.2 for reporting instructions), per the definition of serious adverse event in Section 5.2.2.

[d] Grade 4 and 5 events must be reported as serious adverse events (see Section 5.4.2 for reporting instructions), per the definition of serious adverse event in Section 5.2.2.

5.3.4 Assessment of Causality of Adverse Events

Investigators should use their knowledge of the patient, the circumstances surrounding the event, and an evaluation of any potential alternative causes to determine whether an adverse event is considered to be related to the study drug, indicating "yes" or "no" accordingly. The following guidance should be taken into consideration (see also Table 24):

- Temporal relationship of event onset to the initiation of study drug
- Course of the event, with special consideration of the effects of dose reduction, discontinuation of study drug, or reintroduction of study drug (as applicable)
- Known association of the event with the study drug or with similar treatments
- Known association of the event with the disease under study
- Presence of risk factors in the patient or use of concomitant medications known to increase the occurrence of the event
- Presence of non-treatment-related factors that are known to be associated with the occurrence of the event

Table 24 Causal Attribution Guidance

Is the adverse event suspected to be caused by the study drug on the basis of facts, evidence, science-based rationales, and clinical judgment?	
YES	There is a plausible temporal relationship between the onset of the adverse event and administration of the study drug, and the adverse event cannot be readily explained by the patient's clinical state, intercurrent illness, or concomitant therapies; and/or the adverse event follows a known pattern of response to the study drug; and/or the adverse event abates or resolves upon discontinuation of the study drug or dose reduction and, if applicable, reappears upon re-challenge.
NO	An adverse event will be considered related, unless it fulfills the criteria specified below. Evidence exists that the adverse event has an etiology other than the study drug (e.g., preexisting medical condition, underlying disease, intercurrent illness, or concomitant medication); and/or the adverse event has no plausible temporal relationship to administration of the study drug (e.g., cancer diagnosed 2 days after first dose of study drug).

For patients receiving combination therapy, causality will be assessed individually for each protocol-mandated therapy.

5.3.5 Procedures for Recording Adverse Events

Investigators should use correct medical terminology/concepts when recording adverse events on the Adverse Event eCRF. Avoid colloquialisms and abbreviations.

Only one adverse event term should be recorded in the event field on the Adverse Event eCRF.

5.3.5.1 Infusion-Related Reactions/Cytokine Release Syndrome and Anaphylaxis Attributed to Mosunetuzumab

Given the mechanism of action of mosunetuzumab, IRRs and CRS may be indistinguishable from one another (Section 5.1.2.1). As patients are receiving corticosteroid premedication with mosunetuzumab, fever response may be blunted. Therefore, all adverse events consistent with a diagnosis of infusion-related event or CRS that are attributed to mosunetuzumab, and associated with fever, hypotension or hypoxia not attributable to any other cause, will be recorded singularly as CRS. Adverse events of CRS are graded using the ASTCT CRS Consensus Grading criteria (Appendix 8).

The one exception to this reporting guidance is if a clinical presentation suggests an immediate, acute hypersensitivity (e.g., generalized hives, mucosal edema, with or without wheezing and hypotension), a diagnosis of "allergic reaction" or "anaphylaxis" should be used.

For adverse events with a diagnosis of "cytokine release syndrome" or "infusion-related reaction", associated signs and symptoms should be recorded on the dedicated eCRF

for CRS events. Each CRS event should be recorded separately on the Adverse Event eCRF and should be graded according to ASTCT CRS Consensus Grading, including events without fever, but manifesting with hypotension or hypoxia. The associated signs and symptoms, including those relating to blood pressure and hypoxia, should be graded according to NCI-CTCAE v5.0 and recorded on the dedicated eCRF for CRS/IRR events, as well as the management of these symptoms, for example, pressor and oxygen use. All other laboratory abnormalities (e.g., LFTs, cytopenias) and end-organ adverse events (e.g., neurologic toxicities) should be reported separately in the adverse events eCRF. Organ toxicities that may occur concurrently with CRS (e.g., neurologic signs and symptoms, or liver-associated enzyme abnormalities) should be reported separately on the Adverse Event eCRF. Adverse events attributable to mosunetuzumab and consistent with a diagnosis of IRR or CRS but not associated with fever, hypotension or hypoxia, should be reported separately on the Adverse Event eCRF.

Ambiguous terms such as “systemic reaction” should be avoided.

In addition to documentations on the Adverse Event eCRF, non-serious Grade ≥2 CRS events should be reported as an adverse event of special interest (see Section 5.2.3).

5.3.5.2 Injection-Site Reactions

Localized injection-site reactions following SC mosunetuzumab administration should be captured as a diagnosis of "injection site reactions." Associated signs and symptoms will be recorded separately on the dedicated Injection-Site Reaction eCRF. In addition, Grade ≥2 injection-site reaction should be reported as a non-serious adverse event of special interest (see Section 5.2.3).

5.3.5.3 Infusion-Related Reactions Attributed to Polatuzumab Vedotin or Rituximab

Adverse events that occur during or within 24 hours after study drug administration and are judged to be related to polatuzumab vedotin or rituximab infusion should be captured as a diagnosis "infusion-related reaction," "hypersensitivity reaction," or "anaphylactic reaction" on the Adverse Event eCRF. If possible, avoid ambiguous terms such as "systemic reaction." Associated signs and symptoms should be recorded on the dedicated eCRF.

5.3.5.4 Diagnosis versus Signs and Symptoms

For adverse events other than IRRs and CRS reactions (see Sections 5.3.5.1 and 5.3.5.3), a diagnosis (if known) should be recorded on the Adverse Event eCRF rather than individual signs and symptoms (e.g., record only liver failure or hepatitis rather than jaundice, asterixis, and elevated transaminases). However, if a constellation of signs and/or symptoms cannot be medically characterized as a single diagnosis or syndrome at the time of reporting, each individual event should be recorded on the Adverse Event eCRF. If a diagnosis is subsequently established, all previously reported adverse events

based on signs and symptoms should be nullified and replaced by one adverse event report based on the single diagnosis, with a starting date that corresponds to the starting date of the first symptom of the eventual diagnosis.

5.3.5.5 Adverse Events That Are Secondary to Other Events

In general, adverse events that are secondary to other events (e.g., cascade events or clinical sequelae) should be identified by their primary cause, with the exception of severe or serious secondary events. A medically significant secondary adverse event that is separated in time from the initiating event should be recorded as an independent event on the Adverse Event eCRF. For example:

- If vomiting results in mild dehydration with no additional treatment in a healthy adult, only vomiting should be reported on the eCRF.
- If vomiting results in severe dehydration, both events should be reported separately on the eCRF.
- If a severe gastrointestinal hemorrhage leads to renal failure, both events should be reported separately on the eCRF.
- If dizziness leads to a fall and consequent fracture, all three events should be reported separately on the eCRF.
- If neutropenia is accompanied by an infection, both events should be reported separately on the eCRF.

All adverse events should be recorded separately on the Adverse Event eCRF if it is unclear as to whether the events are associated.

5.3.5.6 Persistent or Recurrent Adverse Events

A persistent adverse event is one that extends continuously, without resolution, between patient evaluation timepoints. Such events should only be recorded once on the Adverse Event eCRF. The initial severity (intensity or grade) of the event will be recorded at the time the event is first reported. If a persistent adverse event becomes more severe, the most extreme severity should also be recorded on the Adverse Event eCRF. Details regarding any increases or decreases in severity will be captured on the Adverse Event Intensity or Grade Changes eCRF. If the event becomes serious, it should be reported to the Sponsor immediately (i.e., no more than 24 hours after learning that the event became serious; see Section 5.4.2 for reporting instructions). The Adverse Event eCRF should be updated by changing the event from "non-serious" to "serious," providing the date that the event became serious, and completing all data fields related to serious adverse events.

A recurrent adverse event is one that resolves between patient evaluation timepoints and subsequently recurs. Each recurrence of an adverse event should be recorded as a separate event on the Adverse Event eCRF.

5.3.5.7 Abnormal Laboratory Values

Not every laboratory abnormality qualifies as an adverse event. A laboratory test result must be reported as an adverse event if it meets any of the following criteria:

- Is accompanied by clinical symptoms
- Results in a change in study treatment (e.g., dosage modification, treatment interruption, or treatment discontinuation)
- Results in a medical intervention (e.g., potassium supplementation for hypokalemia) or a change in concomitant therapy
- Is clinically significant in the investigator’s judgment

    Note: For oncology trials, certain abnormal values may not qualify as adverse events.

It is the investigator’s responsibility to review all laboratory findings. Medical and scientific judgment should be exercised in deciding whether an isolated laboratory abnormality should be classified as an adverse event.

If a clinically significant laboratory abnormality is a sign of a disease or syndrome (e.g., alkaline phosphatase and bilirubin 5 × ULN associated with cholestasis), only the diagnosis (i.e., cholestasis) should be recorded on the Adverse Event eCRF.

If a clinically significant laboratory abnormality is not a sign of a disease or syndrome, the abnormality itself should be recorded on the Adverse Event eCRF, along with a descriptor indicating whether the test result is above or below the normal range (e.g., “elevated potassium,” as opposed to “abnormal potassium”). If the laboratory abnormality can be characterized by a precise clinical term per standard definitions, the clinical term should be recorded as the adverse event. For example, an elevated serum potassium level of 7.0 mEq/L should be recorded as “hyperkalemia.”

Observations of the same clinically significant laboratory abnormality from visit to visit should only be recorded once on the Adverse Event eCRF (see Section 5.3.5.6 for details on recording persistent adverse events).

5.3.5.8 Abnormal Vital Sign Values

Not every vital sign abnormality qualifies as an adverse event. A vital sign result must be reported as an adverse event if it meets any of the following criteria:

- Is accompanied by clinical symptoms
- Results in a change in study treatment (e.g., dosage modification, treatment interruption, or treatment discontinuation)
- Results in a medical intervention or a change in concomitant therapy
- Is clinically significant in the investigator’s judgment

It is the investigator's responsibility to review all vital sign findings. Medical and scientific judgment should be exercised in deciding whether an isolated vital sign abnormality should be classified as an adverse event.

If a clinically significant vital sign abnormality is a sign of a disease or syndrome (e.g., high blood pressure), only the diagnosis (i.e., hypertension) should be recorded on the Adverse Event eCRF.

Observations of the same clinically significant vital sign abnormality from visit to visit should only be recorded once on the Adverse Event eCRF (see Section 5.3.5.6 for details on recording persistent adverse events).

5.3.5.9 Abnormal Liver Function Tests

The finding of an elevated ALT or AST ($>3\times$ baseline value) in combination with either an elevated total bilirubin ($>2\times$ ULN) or clinical jaundice in the absence of cholestasis or other causes of hyperbilirubinemia is considered to be an indicator of severe liver injury (as defined by Hy's Law). Therefore, investigators must report as an adverse event the occurrence of either of the following:

- Treatment-emergent ALT or AST $>3\times$ baseline value in combination with total bilirubin $>2\times$ ULN (of which $\geq 35\%$ is direct bilirubin)
- Treatment-emergent ALT or AST $>3\times$ baseline value in combination with clinical jaundice

The most appropriate diagnosis or (if a diagnosis cannot be established) the abnormal laboratory values should be recorded on the Adverse Event eCRF (see Section 5.3.5.7) and reported to the Sponsor immediately (i.e., no more than 24 hours after learning of the event), either as a serious adverse event or an adverse event of special interest (see Section 5.4.2).

5.3.5.10 Deaths

Deaths that occur during the protocol-specified adverse event reporting period (see Section 5.3.1) that are attributed by the investigator solely to progression of DLBCL, FL, or MCL should be recorded on the Death Attributed to Progressive Disease eCRF. All other deaths that occur during the adverse event reporting period, regardless of relationship to study drug, must be recorded on the Adverse Event eCRF and immediately reported to the Sponsor (see Section 5.4.2).

Death should be considered an outcome and not a distinct event. The event or condition that caused or contributed to the fatal outcome should be recorded as the single medical concept on the Adverse Event eCRF. Generally, only one such event should be reported. If the cause of death is unknown and cannot be ascertained at the time of reporting, **"unexplained death"** should be recorded on the Adverse Event eCRF. If the cause of death later becomes available (e.g., after autopsy), "unexplained death" should be replaced by the established cause of death. The term **"sudden death"** should not

be used unless combined with the presumed cause of death (e.g., “sudden cardiac death”).

Deaths that occur after the adverse event reporting period should be reported as described in Section 5.6.

5.3.5.11 Preexisting Medical Conditions

A preexisting medical condition is one that is present at the screening visit for this study. Such conditions should be recorded on the General Medical History and Baseline Conditions eCRF.

A preexisting medical condition should be recorded as an adverse event only if the frequency, severity, or character of the condition worsens during the study. When recording such events on the Adverse Event eCRF, it is important to convey the concept that the preexisting condition has changed by including applicable descriptors (e.g., “more frequent headaches”).

5.3.5.12 Lack of Efficacy or Worsening of DLBCL, FL, or MCL

Events that are clearly consistent with the expected pattern of progression of the underlying disease should not be recorded as adverse events. These data will be captured as efficacy assessment data only. In most cases, the expected pattern of progression will be based on Lugano 2014 criteria. In rare cases, the determination of clinical progression will be based on symptomatic deterioration. However, every effort should be made to document progression through use of objective criteria. If there is any uncertainty as to whether an event is due to disease progression, it should be reported as an adverse event.

5.3.5.13 Hospitalization or Prolonged Hospitalization

Any adverse event that results in hospitalization (i.e., inpatient admission to a hospital) or prolonged hospitalization should be documented and reported as a serious adverse event (per the definition of serious adverse event in Section 5.2.2), except as outlined below.

An event that leads to hospitalization under the following circumstances should not be reported as an adverse event or a serious adverse event:

- Hospitalization for respite care
- Planned hospitalization required by the protocol
- Optional hospitalization for monitoring as described by the protocol (Section 5.1.1).
- Hospitalization for a preexisting condition, provided that all of the following criteria are met:
  - The hospitalization was planned prior to the study or was scheduled during the study when elective surgery became necessary because of the expected normal progression of the disease

- The patient has not experienced an adverse event

- Hospitalization due solely to progression of the underlying cancer

An event that leads to hospitalization under the following circumstances is not considered to be a serious adverse event, but should be reported as an adverse event instead:

- Hospitalization for an adverse event that would ordinarily have been treated in an outpatient setting had an outpatient clinic been available

5.3.5.14 Patient-Reported Outcome Data

Adverse event reports will not be derived from PRO data by the Sponsor and safety analyses will not be performed using PRO data. Sites are not expected to review the PRO data for adverse events.

5.4 IMMEDIATE REPORTING REQUIREMENTS FROM INVESTIGATOR TO SPONSOR

Certain events require immediate reporting to allow the Sponsor to take appropriate measures to address potential new risks in a clinical trial. The investigator must report such events to the Sponsor immediately; under no circumstances should reporting take place more than 24 hours after the investigator learns of the event. The following is a list of events that the investigator must report to the Sponsor within 24 hours after learning of the event, regardless of relationship to study drug:

- Serious adverse events (defined in Section 5.2.2; see Section 5.4.2 for details on reporting requirements)
- Adverse events of special interest (defined in Section 5.2.3; see Section 5.4.2 for details on reporting requirements)
- Pregnancies (see Section 5.4.3 for details on reporting requirements)
- Accidental overdoses or medication errors (see Section 5.4.4 for details on reporting requirements)

The investigator must report new significant follow-up information for these events to the Sponsor immediately (i.e., no more than 24 hours after becoming aware of the information). New significant information includes the following:

- New signs or symptoms or a change in the diagnosis
- Significant new diagnostic test results
- Change in causality based on new information
- Change in the event's outcome, including recovery
- Additional narrative information on the clinical course of the event

Investigators must also comply with local requirements for reporting serious adverse events to the local health authority and IRB/EC.

5.4.1 Emergency Medical Contacts

Medical Monitor Contact Information for All Sites

Medical Monitor: [redacted] *Pharm.D*. (Primary)

Telephone No.: [redacted]

Medical Monitor (Back up) [redacted]*, M.D.*

Mobile Telephone No.: [redacted]

Roche Medical Responsible: [redacted], Ph.D. (Secondary)

Mobile Telephone No.: [redacted]

To ensure the safety of study patients, an Emergency Medical Call Center Help Desk will access the Roche Medical Emergency List, escalate emergency medical calls, provide medical translation service (if necessary), connect the investigator with a Roche Medical Responsible (listed above and/or on the Roche Medical Emergency List), and track all calls. The Emergency Medical Call Center Help Desk will be available 24 hours per day, 7 days per week. Toll-free numbers for the Help Desk, as well as Medical Monitor and Medical Responsible contact information, will be distributed to all investigators.

5.4.2 Reporting Requirements for Serious Adverse Events and Adverse Events of Special Interest

5.4.2.1 Events That Occur prior to Study Drug Initiation

After informed consent has been obtained but prior to initiation of study drug, only serious adverse events caused by a protocol-mandated intervention should be reported. The paper Clinical Trial Adverse Event/*Special Situations Form* provided to investigators should be completed and submitted to the Sponsor or its designee immediately (i.e., no more than 24 hours after learning of the event), either by faxing or by scanning and emailing the form using the fax number or email address provided to investigators.

5.4.2.2 Events That Occur after Study Drug Initiation

After initiation of study drug, serious adverse events and adverse events of special interest will be reported until 90 days after the last dose of study drug. DLTs will be reported during the DLT assessment window. Investigators should record all case details that can be gathered immediately (i.e., within 24 hours after learning of the event) on the Adverse Event eCRF and submit the report via the electronic data capture (EDC) system. A report will be generated and sent to Roche Safety Risk Management by the EDC system.

In the event that the EDC system is unavailable, the paper Clinical Trial Adverse Event/*Special Situations Form* provided to investigators should be completed and submitted to the Sponsor or its designee immediately (i.e., no more than 24 hours after learning of the event), either by faxing or by scanning and emailing the form using the

fax number or email address provided to investigators. Once the EDC system is available, all information will need to be entered and submitted via the EDC system.

Instructions for reporting serious adverse events that occur > 90 days after the last dose of study treatment are provided in Section 5.6.

5.4.3 Reporting Requirements for Pregnancies

5.4.3.1 Pregnancies in Female Patients

Female patients of childbearing potential will be instructed through the Informed Consent Form to immediately inform the investigator if they become pregnant during the study or within 3 months after the final dose of mosunetuzumab, 9 months after the final dose of polatuzumab vedotin, 12 months after the final dose of rituximab, and 3 months after the final dose of tocilizumab, as applicable. The investigator should report the pregnancy on the paper Clinical Trial Pregnancy Reporting Form and submit the form to the Sponsor or its designee immediately (i.e., no more than 24 hours after learning of the pregnancy), either by faxing or by scanning and emailing the form using the fax number or email address provided to investigators. Pregnancy should not be recorded on the Adverse Event eCRF. The investigator should discontinue study drug and counsel the patient, discussing the risks of the pregnancy and the possible effects on the fetus. Monitoring of the patient should continue until conclusion of the pregnancy. Any serious adverse events associated with the pregnancy (e.g., an event in the fetus, an event in the mother during or after the pregnancy, or a congenital anomaly/birth defect in the child) should be reported on the Adverse Event eCRF. In addition, the investigator will submit a Clinical Trial Pregnancy Reporting Form with additional information on the pregnant partner and the course and outcome of the pregnancy as it becomes available.

5.4.3.2 Pregnancies in Female Partners of Male Patients

Male patients will be instructed through the Informed Consent Form to immediately inform the investigator if their partner becomes pregnant during the study or 6 months after the final dose of polatuzumab vedotin, 3 months after the final dose of rituximab, or 60 days after the final dose of tocilizumab, as applicable. *The investigator should report the pregnancy on the* paper Clinical Trial Pregnancy Reporting Form *and submit the form* to the Sponsor or its designee immediately (i.e., no more than 24 hours after learning of the pregnancy), either by faxing or by scanning and emailing the form using the fax number or email address provided to investigators. Attempts should be made to collect and report details of the course and outcome of any pregnancy in the partner of a male patient exposed to study drug. When permitted by the site, the pregnant partner would need to sign an Authorization for Use and Disclosure of Pregnancy Health Information to allow for follow-up on her pregnancy. If the authorization has been signed, the investigator should submit a Clinical Trial Pregnancy Reporting Form *with additional* information on the *pregnant partner and the* course and outcome of the pregnancy *as it* becomes available. An investigator who is contacted by the male patient or his pregnant partner may provide information on the risks of the pregnancy

and the possible effects on the fetus, to support an informed decision in cooperation with the treating physician and/or obstetrician.

5.4.3.3 Abortions

Any spontaneous abortion should be classified as a serious adverse event (as the Sponsor considers abortions to be medically significant), recorded on the Adverse Event eCRF, and reported to the Sponsor immediately (i.e., no more than 24 hours after learning of the event; see Section 5.4.2).

If a therapeutic or elective abortion was performed because of an underlying maternal or embryofetal toxicity, the toxicity should be classified as a serious adverse event, recorded on the Adverse Event eCRF, and reported to the Sponsor immediately (i.e., no more than 24 hours after learning of the event; see Section 5.4.2). A therapeutic or elective abortion performed for reasons other than an underlying maternal or embryofetal toxicity is not considered an adverse event.

All abortions should be reported as pregnancy outcomes on the paper Clinical Trial Pregnancy Reporting Form.

5.4.3.4 Congenital Anomalies/Birth Defects

Any congenital anomaly/birth defect in a child born to a female patient exposed to study drug or the female partner of a male patient exposed to study drug should be classified as a serious adverse event, recorded on the Adverse Event eCRF, and reported to the Sponsor immediately (i.e., no more than 24 hours after learning of the event; see Section 5.4.2).

5.4.4 Reporting Requirements for Cases of Accidental Overdose or Medication Error

Accidental overdose and medication error (hereafter collectively referred to as “special situations”), are defined as follows:

- Accidental overdose: accidental administration of a drug in a quantity that is higher than the assigned dose
- Medication error: accidental deviation in the administration of a drug
  - In some cases, a medication error may be intercepted prior to administration of the drug.

Special situations are not in themselves adverse events but may result in adverse events. All special situations associated with mosunetuzumab, polatuzumab vedotin, rituximab, and tocilizumab, regardless of whether they result in an adverse event, should be recorded on the Adverse Event eCRF and reported to the Sponsor immediately

(i.e., no more than 24 hours after learning of the event). Special situations should be recorded as described below:

- Accidental overdose: Enter the drug name and “accidental overdose” as the event term. Check the “Accidental overdose” and “Medication error” boxes.
- Medication error that does not qualify as an overdose: Enter the name of the drug administered and a description of the error (e.g., wrong dose administered, wrong dosing schedule, incorrect route of administration, wrong drug, expired drug administered) as the event term. Check the “Medication error” box.
- Medication error that qualifies as an overdose: Enter the drug name and “accidental overdose” as the event term. Check the “Accidental overdose” and “Medication error” boxes. Enter a description of the error in the additional case details.
- Intercepted medication error: Enter the drug name and “intercepted medication error” as the event term. Check the “Medication error” box. Enter a description of the error in the additional case details.

Each adverse event associated with a special situation should be recorded separately on the Adverse Event eCRF. If the associated adverse event fulfills seriousness criteria or qualifies as an adverse event of special interest, the event should be reported to the Sponsor immediately (i.e., no more than 24 hours after learning of the event; see Section 5.4.2). Adverse events associated with special situations should be recorded as described below for each situation:

- Accidental overdose: Enter the adverse event term. Check the “Accidental overdose” and “Medication error” boxes.
- Medication error that does not qualify as an overdose: Enter the adverse event term. Check the “Medication error” box.
- Medication error that qualifies as an overdose: Enter the adverse event term. Check the “Accidental overdose” and “Medication error” boxes.

As an example, an accidental overdose that resulted in a headache would require the completion of two Adverse Event eCRF pages, one to report the accidental overdose and one to report the headache. The “Accidental overdose” and “Medication error” boxes would need to be checked on both eCRF pages.

5.5 FOLLOW-UP OF PATIENTS AFTER ADVERSE EVENTS

5.5.1 Investigator Follow-Up

The investigator should follow each adverse event until the event has resolved to baseline grade or better, the event is assessed as stable by the investigator, the patient is lost to follow-up, or the patient withdraws consent. Every effort should be made to follow all serious adverse events considered to be related to study drug or trial-related procedures until a final outcome can be reported.

During the study period, resolution of adverse events (with dates) should be documented on the Adverse Event eCRF and in the patient's medical record to facilitate source data verification.

All pregnancies reported during the study should be followed until pregnancy outcome.

5.5.2 Sponsor Follow-Up

For serious adverse events, adverse events of special interest, and pregnancies, the Sponsor or a designee may follow up by telephone, fax, email, and/or a monitoring visit to obtain additional case details and outcome information (e.g., from hospital discharge summaries, consultant reports, autopsy reports) in order to perform an independent medical assessment of the reported case.

5.6 ADVERSE EVENTS THAT OCCUR AFTER THE ADVERSE EVENT REPORTING PERIOD

The Sponsor should be notified if the investigator becomes aware of any serious adverse event that occurs after the end of the adverse event reporting period (defined as 90 days after the last dose of study drug or the initiation of *next anti-lymphoma treatment*, whichever is earlier), if the event is believed to be related to prior study drug treatment. These events should be reported through use of the Adverse Event eCRF. However, if the EDC system is not available, the investigator should report these events directly to the Sponsor or its designee, either by faxing or by scanning and emailing the paper Clinical Trial Adverse Event/*Special Situations Form* using the fax number or email address provided to investigators.

5.7 EXPEDITED REPORTING TO HEALTH AUTHORITIES, INVESTIGATORS, INSTITUTIONAL REVIEW BOARDS, AND ETHICS COMMITTEES

The Sponsor will promptly evaluate all serious adverse events and adverse events of special interest against cumulative product experience to identify and expeditiously communicate possible new safety findings to investigators, IRBs, ECs, and applicable health authorities based on applicable legislation.

To determine reporting requirements for single adverse event cases, the Sponsor will assess the expectedness of these events using the following reference documents:

- Mosunetuzumab Investigator's Brochure
- Polatuzumab Vedotin Investigator's Brochure
- Rituximab Investigator's Brochure
- Tocilizumab Investigator's Brochure

The Sponsor will compare the severity of each event and the cumulative event frequency reported for the study with the severity and frequency reported in the applicable reference document.

Reporting requirements will also be based on the investigator's assessment of causality and seriousness, with allowance for upgrading by the Sponsor as needed.

6. STATISTICAL CONSIDERATIONS AND ANALYSIS PLAN

6.1 DETERMINATION OF SAMPLE SIZE

The sample size for the Phase Ib dose-finding portion of the study is based on the dose-escalation rules described in Section 3.1.2. The planned enrollment for the dose-finding phase is approximately 9–42 patients. After the RP2D has been determined for mosunetuzumab combined with polatuzumab vedotin, a total of approximately [redacted] patients will be enrolled in the Phase II single-arm expansion portion of the study (100 patients in the R/R DLBCL cohort, [redacted] patients in the R/R FL cohort, and [redacted] patients in the R/R MCL cohort). Approximately [redacted] patients with R/R DLBCL will also be enrolled in the Phase II randomized portion of the study.

The primary efficacy endpoint for the Phase II expansion is best ORR as determined by PET-CT and/or CT scan as assessed by the IRC (Arms I, J, K, L and M). The primary analysis will be estimation of best ORR in patients treated with mosunetuzumab in combination with polatuzumab vedotin in the R/R DLBCL cohort (Arms J and L), in patients treated with mosunetuzumab in combination with polatuzumab vedotin in the R/R FL cohort (Arm I), in patients treated with mosunetuzumab in combination with polatuzumab vedotin in the R/R MCL cohort (Arm K), and in patients treated with rituximab in combination with polatuzumab vedotin in the R/R DLBCL cohort (Arm M).

With [redacted], [redacted], or [redacted] patients in a treatment arm, the 95% exact Clopper-Pearson CIs (Clopper and Pearson 1934) for estimation of the true ORR would have a margin of error not exceeding ±16.7%, ±11.6%, or ±10.3% respectively. Table 25 shows Clopper-Pearson exact 95% CIs corresponding to observed ORR ranging from 30%–80% based on sample sizes of [redacted], [redacted] and [redacted]

For the R/R DLBCL/trFL/FL3b expansion cohort of Arm J, with observed ORR of 65%, a sample size of 100 patients will result in 95% CI of (55%, 74%; i.e., a true ORR of 42% is ruled out). Additionally, the planned sample size of 100 patients will provide more extensive safety data and 99% power to detect a difference in ORR, with a two-sided significance level of 5%.

For the R/R DLBCL randomized Phase II portion Arms L and M, the margin of error for 95% exact Clopper-Pearson CIs for estimation of the true ORR would not exceed ±16.7% *and ±11.6%*, with [redacted] *and* [redacted] patients in each arm*, respectively*.

Table 25 Clopper-Pearson Exact 95% Confidence Intervals for Assumed Observed ORR based on Sample Size of [redacted], [redacted], and [redacted] Patients

Observed ORR	No. of Patients with ORR (95% CI for rate)		
	n = [redacted]	n = [redacted]	n = [redacted]
80%	[redacted]		
75%			
70%			
65%			
60%			
55%			
50%			
45%			
40%			
35%			
30%			

ORR = overall response rate.

With respect to assessment of safety, point estimates will be presented. Table 26 provides probabilities of seeing at least one adverse event among [redacted] patients for true adverse event frequencies ranging from 1% to 20%. For example, with [redacted] patients in a treatment arm, there is at least an 87% chance of observing at least one adverse event with true incidence of ≥ 5%.

Table 26 Probability of Safety-Signal Detection based on Sample Size of [redacted], [redacted], and [redacted] Patients

True Underlying Probability of an adverse event	Probability of Observing at Least 1 adverse event in [redacted] Patients	Probability of Observing at Least 1 adverse event in [redacted] Patients	Probability of Observing at Least 1 adverse event in [redacted] Patients
1%	33%	55%	63%
5%	87%	98%	99%
10%	99%	> 99%	> 99%
15%	> 99%	> 99%	> 99%
20%	> 99%	> 99%	> 99%

6.2 SUMMARIES OF CONDUCT OF STUDY

Enrollment, major protocol violations (including violations of inclusion/exclusion criteria), and study drug administration will be summarized by treatment arm and dose level. Patient disposition will be summarized by treatment arm and dose level and will include whether treatment was completed or discontinued early and the reason for early treatment termination.

6.3 SUMMARIES OF TREATMENT GROUP COMPARABILITY

Demographic and baseline characteristics, such as age, sex, race/ethnicity, duration of malignancy, and baseline ECOG PS will be summarized by using means, standard deviations, medians, and ranges for continuous variables and proportions for categorical variables. All summaries will be presented overall and by treatment arm and dose level.

6.4 EFFICACY ANALYSES

For Arms I, J, K, L and M, the population for the efficacy analyses will consist of all treated patients, with patients grouped according to their *received treatment*.

The efficacy analyses in the Phase Ib dose-finding portion of the study are exploratory. Descriptive summary statistics will be provided for all efficacy endpoints.

6.4.1 Primary Efficacy Endpoint

The primary efficacy endpoint for Arms I, J, K, L and M is best ORR, defined as the percentage of patients with CR or PR at any time based on PET-CT and/or CT scan and as determined by the IRC using the Lugano 2014 criteria (Cheson et al. 2014) specified in Appendix 7. Patients with missing or no response assessments will be classified as non-responders. The best ORRs between Arms L and M will be compared approximately 6 months after the last patient is enrolled in the randomized Phase II portion. *A best ORR delta of ≥10% between Arm L over Arm M would demonstrate clinical benefits of mosunetuzumab in combination with polatuzumab vedotin over rituximab in combination with polatuzumab vedotin.*

Comparison with respect to ORR between the treated patient population and historical controls will be tested for Arm J (R/R DLBCL/trFL/FL3b) and Arm K (R/R MCL).
The control ORR is assumed to be 42% for Arm J and 30% for Arm K (see *Table 1 and* Table 3 for historical controls).

The following hypothesis will be tested at a one-sided 0.025 level of significance using an exact binomial test in Arm J cohort:

Ho: ORR $=$42% versus Ha: ORR $>$42%.

The following hypothesis will be tested at a one-sided 0.025 level of significance using an exact binomial test in Arm K cohort:

Ho: ORR = 30% versus Ha: ORR > 30%.

The ORR will be estimated and the corresponding Clopper-Pearson exact 95% CI will be constructed for each treatment arm.

6.4.2 Secondary Efficacy Endpoints

Secondary efficacy endpoints of response rates using the Lugano 2014 criteria (Cheson et al. 2014, Appendix 7) are described below. Patients with missing or no response assessments will be classified as non-responders. For each of these endpoints of response rates, the point estimate and the corresponding Clopper-Pearson exact 95% CI will be consutrcted for each treatment arm.

- CR rate at PRA as measured by PET-CT scan and as determined by the investigator and by the IRC
- ORR, defined as the percentage of patients with PR or CR at PRA as measured by PET-CT scan and as determined by the IRC and by the investigator
- Best ORR, defined as the percentage of patients with any PR or CR while in the study as measured by PET-CT and/or CT only and as determined by the investigator
- Best CR rate, defined as the percentage of patients with any CR while in the study as measured by PET-CT and/or CT only and as determined by the IRC and by the investigator

DOR, defined as the time from the first occurrence of a documented objective response (CR or PR) to the time of disease progression, relapse, or death from any cause, whichever occurs first. For patients who do not experience death or disease progression, DOR will be censored at the date of last evaluable tumor assessment. DOR will be assessed per IRC and per investigator, using the Lugano 2014 criteria (Cheson et al. 2014; see Appendix 7). Analyses of DOR will include only patients with objective responses (CR or PR) at any time in the study. The Kaplan-Meier method (Kaplan and Meier 1958) will be used to estimate the distribution of DOR and median DOR (if analytically possible) for each treatment arm, with 95% CI for the median DOR constructed using the Brookmeyer-Crowley method (Brookmeyer and Crowley 1982). DOR will also be summarized for the subgroups of patients whose best objective response is PR and patients whose best objective response is CR.

PFS, defined as the time from first study treatment to the first occurrence of disease progression, relapse, or death from any cause, whichever occurs first. For patients who do not experience disease progression, relapse or death, PFS will be censored at the date of last evaluable tumor assessment. For patients who do not have a post-baseline evaluable tumor assessment, PFS will be censored at D1. The PFS will be assessed

per IRC and per investigator, using the Lugano 2014 criteria (Cheson et al. 2014; see Appendix 7. The Kaplan-Meier method (Kaplan and Meier 1958) will be used to estimate the distribution of PFS, median (if analytically possible), and 6-month and 1-year PFS for each treatment arm. The Brookmeyer-Crowley method (Brookmeyer and Crowley 1982) will be used to construct the 95% CI for the median PFS. The Greenwood's formula will be used to provide standard errors and the corresponding 95% CIs for 6-month PFS and 1-year PFS.

EFS, defined as the time from first study treatment to the first occurrence of any treatment failure including disease progression, relapse, initiation of NALT, or death. For patients who do not experience the specified event (disease progression/relapse, death, start of an NALT), EFS will be censored at the date of last evaluable tumor assessment. For patients who do not have a post-baseline evaluable tumor assessment or documentation of NALT, EFS will be censored at D1. EFS will be assessed per IRC and per investigator, using the Lugano 2014 criteria (Cheson et al. 2014; see Appendix 7). Analyses of EFS will be identical to those outlined previously for PFS.

OS, defined as the time from first study treatment to the date of death from any cause. Patients who have not died will be censored at the last date known to be alive. Analyses of OS will be identical to those outlined previously for PFS.

6.4.3 <u>Exploratory Efficacy Endpoints</u>

Proportion of patients who undergo SCT after achieving a response while in the study will be analyzed for Arms I, J, K, L, M and M-crossover. Analyses may be conducted on different subpopulations based on transplant eligibility status at baseline.

For the exploratory PRO endpoint for the Arm K expansion phase, descriptive summary statistics and change from baseline will be calculated at each assessment for all scales of the EQ-5D-5L.

CIRS-G assessment endpoints for Arms J, L, and M

Univariate and multivariate analyses of prognostic factors for OS and PFS, will be conducted with the safety-evaluable population in patients who are ≥65 years old, using log-rank test or chi-squared test. One of the prognostic factors to be considered is CIRS-G. The HR for the prognostic factors will be estimated using a stratified Cox proportional hazards model. The 95% CI for the hazard ratio will be provided.

6.5 SAFETY ANALYSES

All safety analyses will be based on the safety-evaluable population (i.e., patients who received any study treatment), according to the actual treatment received. Safety will be assessed through summaries of adverse events, changes in laboratory test results, changes in ECGs, changes in ADAs, and changes in vital signs. All collected adverse event data will be listed by phase of the study, assigned treatment arm and dose level,

and patient number. All adverse events occurring on or after first study treatment will be summarized by mapped terms, appropriate thesaurus levels, and toxicity grade per NCI CTCAE v5.0 (or, for CRS events, per ASTCT consensus grading criteria). In addition, all serious adverse events, including deaths, will be listed separately and summarized. DLTs and adverse events leading to treatment discontinuation will also be separately listed. Selected laboratory data will be listed, with values outside of normal ranges identified.

6.6 PHARMACOKINETIC ANALYSES

Individual and mean serum concentration of mosunetuzumab and polatuzumab vedotin versus time data will be tabulated and plotted by dose level. The C_{max} and C_{min}, of mosunetuzumab and polatuzumab vedotin will be summarized. Additional PK parameters will be calculated including area AUC, CL, and V_{ss}, as appropriate for data collected. Estimates for these parameters will be tabulated and summarized.

Predose rituximab and obinutuzumab concentrations will be summarized for patients who received prior rituximab or obinutuzumab treatments.

Additional PK analyses will be conducted as appropriate.

6.7 IMMUNOGENICITY ANALYSES

Validated screening, titering, and confirmatory assays will be employed to assess ADAs before, during, and after treatment with mosunetuzumab and polatuzumab vedotin (see Appendix 3 and Appendix 4).

The immunogenicity analysis population will consist of all patients with at least one ADA assessment. Patients are considered to be negative for ADAs if they are ADA negative at all timepoints. Patients are considered to be treatment unaffected if they are ADA positive at baseline but do not have any postbaseline samples with a titer that is at least 4-fold greater than the titer of the baseline sample. Patients are considered to have treatment-induced ADA responses if they are ADA negative or missing data at baseline and then develop an ADA response following study drug administration. Patients are considered to have treatment-enhanced ADA responses if they are ADA positive at baseline and the titer of one or more postbaseline samples is at least 4-fold greater (i.e., at least 0.60 titer unit) than the titer of the baseline sample.

The relationship between ADA status and safety, efficacy, PK, and biomarker endpoints may also be assessed as appropriate and reported in a descriptive manner via subgroup analyses, as appropriate.

6.8 BIOMARKER ANALYSES

Exploratory analyses of biomarkers related to tumor and disease biology as well as the mechanisms of action of polatuzumab vedotin and mosunetuzumab will be conducted. The association between candidate biomarkers and ORR and other measures of

efficacy and safety, with treatment and independent of treatment, will be explored to assess potential predictive and prognostic value, respectively. The effects of baseline prognostic characteristics, including NHL subtypes and mutation profiles on efficacy, will be evaluated using univariate and/or multivariate statistical methods such as Cox regression and logistic regression.

Exploratory PD analyses may include assessments of cytokines, T-cell activation and proliferation, NK cells, B cells as well as other assessments of biomarkers in both tumor tissue and blood when available.

6.9 INTERIM ANALYSES

6.9.1 <u>Planned Interim Safety Analyses</u>

The IMC will review all cumulative safety data by cohort and by treatment arm on a periodic basis during the Phase II expansion portion of the study, occurring when approximately [redacted] and [redacted] total patients in the Phase II single-arm expansion cohorts have each received at least 2 cycles of treatment, or more frequently as indicated or requested by the Medical Monitor. The IMC may make recommendations regarding study conduct, including but not limited to, the following: performing additional safety analyses, amending the study protocol, holding patient enrollment pending further safety evaluations, holding/discontinuing study treatment, making decisions to modify or discontinue the requirement for hospitalization with study treatment, or terminating the study. Any outcomes of these safety reviews that affect study conduct will be communicated in a timely manner to the investigators for notification of the IRB/EC. A detailed plan will be included in the IMC Charter.

6.9.2 <u>Interim Futility Analysis</u>

An interim futility analysis will be conducted in Arm K. If the interim analysis, which will be conducted at least 3 months after approximately [redacted] patients have been dosed *with at least one dose of study treatment*, demonstrates a posterior probability of $<$30% that the true investigator-assessed ORR is $>$30% (e.g., observing no more than [redacted] responders in [redacted] patients), enrollment in Arm K may be stopped.

An interim futility analysis will be conducted in Arms L and M. If the interim analysis, which will be conducted at least 3 months after approximately [redacted] patients in Arm M have been dosed with at least one dose of study treatment, demonstrates a posterior probability of >85% that the true investigator-assessed ORR in Arm M is >42% (e.g., observing at least [redacted] [[redacted]%] responders in [redacted] patients), enrollment in Arms L and M may expand to [redacted] patients total ([redacted] patients in each arm). Otherwise, if the interim analysis demonstrates a posterior probability of ≤85% that the true investigator-assessed ORR in Arm M is >42% (e.g., observing no more than [redacted] [[redacted]%] responders in [redacted] patients), enrollment in Arms L and M may be stopped at [redacted] patients total ([redacted] patients in each arm).

6.9.3 Optional Interim Analyses

Given the hypothesis-generating nature of this study, the Sponsor may choose to conduct interim efficacy analyses. The decision to conduct an optional interim analysis and the timing of the analysis will be documented in the Sponsor's trial master file prior to the conduct of the interim analysis. The interim analysis will be performed and interpreted by Sponsor study team personnel.

7. DATA COLLECTION AND MANAGEMENT

7.1 DATA QUALITY ASSURANCE

The Sponsor will be responsible for data management of this study, including quality checking of the data. Data entered manually will be collected via EDC through use of eCRFs. Sites will be responsible for data entry into the EDC system. In the event of discrepant data, the Sponsor will request data clarification from the sites, which the sites will resolve electronically in the EDC system.

The Sponsor will produce an EDC Study Specification document that describes the quality checking to be performed on the data. Central laboratory data will be sent directly to the Sponsor, using the Sponsor's standard procedures to handle and process the electronic transfer of these data.

The eCRFs and correction documentation will be maintained in the EDC system's audit trail. System backups for data stored by the Sponsor and records retention for the study data will be consistent with the Sponsor's standard procedures.

The PRO data will be collected on paper questionnaires. The data from the questionnaires will be entered into the EDC system by site staff. In the event due to Covid-19 restrictions patients are not able to come to the clinic for their scheduled assessment, sites can call patients, and, using phone scripts, read questions verbatim to patients while capturing their responses on the paper questionnaire the patients would have completed upon coming to the clinic. Source documentation sufficient to pass an audit should be obtained, which includes information that the questionnaires were administered via phone because of COVID-19.

7.2 ELECTRONIC CASE REPORT FORMS

The eCRFs are to be completed through use of a Sponsor-designated EDC system. Sites will receive training and have access to a manual for appropriate eCRF completion. eCRFs will be submitted electronically to the Sponsor and should be handled in accordance with instructions from the Sponsor.

All eCRFs should be completed by designated, trained site staff. The eCRFs should be reviewed and electronically signed and dated by the investigator or a designee.

At the end of the study, the investigator will receive patient data for his or her site in a readable format on a compact disc that must be kept with the study records. Acknowledgement of receipt of the compact disc is required.

7.3 PATIENT-REPORTED OUTCOME DATA

PROs will be collected in patients enrolled in NHL expansion cohorts. PRO data will be collected on paper questionnaires. The data from the questionnaires will be entered into the EDC system by site staff. In the event due to Covid-19 restrictions patients are not able to come to the clinic for their scheduled assessment, sites can call patients, and, using phone scripts, read questions verbatim to patients while capturing their responses on the paper questionnaire the patients would have completed upon coming to the clinic. Source documentation sufficient to pass an audit should be obtained, which includes information that the questionnaires were administered via phone because of Covid-19.

7.4 SOURCE DATA DOCUMENTATION

Study monitors will perform ongoing source data verification and review to confirm that critical protocol data (i.e., source data) entered into the eCRFs by authorized site personnel are accurate, complete, and verifiable from source documents.

Source documents (paper or electronic) are those in which patient data are recorded and documented for the first time. They include, but are not limited to, hospital records, clinical and office charts, laboratory notes, memoranda, PROs, evaluation checklists, pharmacy dispensing records, recorded data from automated instruments, copies of transcriptions that are certified after verification as being accurate and complete, microfiche, photographic negatives, microfilm or magnetic media, X-rays, patient files, and records kept at pharmacies, laboratories, and medico-technical departments involved in a clinical trial.

Before study initiation, the types of source documents that are to be generated will be clearly defined in the Trial Monitoring Plan. This includes any protocol data to be entered directly into the eCRFs (i.e., no prior written or electronic record of the data) and considered source data.

Source documents that are required to verify the validity and completeness of data entered into the eCRFs must not be obliterated or destroyed and must be retained per the policy for retention of records described in Section 7.6.

To facilitate source data verification and review, the investigators and institutions must provide the Sponsor direct access to applicable source documents and reports for trial-related monitoring, Sponsor audits, and IRB/EC review. The study site must also allow inspection by applicable health authorities.

7.5 USE OF COMPUTERIZED SYSTEMS

When clinical observations are entered directly into a study site's computerized medical record system (i.e., in lieu of original hardcopy records), the electronic record can serve as the source document if the system has been validated in accordance with health authority requirements pertaining to computerized systems used in clinical research. An acceptable computerized data collection system allows preservation of the original entry of data. If original data are modified, the system should maintain a viewable audit trail that shows the original data as well as the reason for the change, name of the person making the change, and date of the change.

7.6 RETENTION OF RECORDS

Records and documents pertaining to the conduct of this study and the distribution of IMP, including eCRFs, electronic or paper PRO data (if applicable), Informed Consent Forms, laboratory test results, and medication inventory records, must be retained by the Principal Investigator for 15 years after completion or discontinuation of the study or for the length of time required by relevant national or local health authorities, whichever is longer. After that period of time, the documents may be destroyed, subject to local regulations.

No records may be disposed of without the written approval of the Sponsor. Written notification should be provided to the Sponsor prior to transferring any records to another party or moving them to another location.

Roche will retain study data for 25 years after the final Clinical Study Report has been completed or for the length of time required by relevant national or local health authorities, whichever is longer.

8. ETHICAL CONSIDERATIONS

8.1 COMPLIANCE WITH LAWS AND REGULATIONS

This study will be conducted in full conformance with the ICH E6 guideline for Good Clinical Practice and the principles of the Declaration of Helsinki, or the applicable laws and regulations of the country in which the research is conducted, whichever affords the greater protection to the individual. The study will comply with the requirements of the ICH E2A guideline (Clinical Safety Data Management: Definitions and Standards for Expedited Reporting). Studies conducted in the United States or under a U.S. Investigational New Drug (IND) Application will comply with U.S. FDA regulations and applicable local, state, and federal laws. Studies conducted in the European Union or European Economic Area will comply with the E.U. Clinical Trial Directive (2001/20/EC) and applicable local, regional, and national laws.

8.2 INFORMED CONSENT

The Sponsor's sample Informed Consent Form (and ancillary sample Informed Consent Forms such as a Child's Informed Assent Form or Mobile Nursing Informed Consent

Form, if applicable) will be provided to each site. If applicable, it will be provided in a certified translation of the local language. The Sponsor or its designee must review and approve any proposed deviations from the Sponsor's sample Informed Consent Forms or any alternate consent forms proposed by the site (collectively, the "Consent Forms") before IRB/EC submission. The final IRB/EC–approved Consent Forms must be provided to the Sponsor for health authority submission purposes according to local requirements.

If applicable, the Informed Consent Form will contain separate sections for any optional procedures. The investigator or authorized designee will explain to each patient the objectives, methods, and potential risks associated with each optional procedure. Patients will be told that they are free to refuse to participate and may withdraw their consent at any time for any reason. A separate, specific signature will be required to document a patient's agreement to participate in optional procedures. Patients who decline to participate will not provide a separate signature.

The Consent Forms must be signed and dated by the patient or the patient's legally authorized representative before his or her participation in the study. The case history or clinical records for each patient shall document the informed consent process and that written informed consent was obtained prior to participation in the study.

The Consent Forms should be revised whenever there are changes to study procedures or when new information becomes available that may affect the willingness of the patient to participate. The final revised IRB/EC-approved Consent Forms must be provided to the Sponsor for health authority submission purposes.

Patients must be re-consented to the most current version of the Consent Forms (or to a significant new information/findings addendum in accordance with applicable laws and IRB/EC policy) during their participation in the study. For any updated or revised Consent Forms, the case history or clinical records for each patient shall document the informed consent process and that written informed consent was obtained using the updated/revised Consent Forms for continued participation in the study.

A copy of each signed Consent Form must be provided to the patient or the patient's legally authorized representative. All signed and dated Consent Forms must remain in each patient's study file or in the site file and must be available for verification by study monitors at any time.

For sites in the United States, each Consent Form may also include patient authorization to allow use and disclosure of personal health information in compliance with the U.S. Health Insurance Portability and Accountability Act (HIPAA) of 1996. If the site utilizes a separate Authorization Form for patient authorization for use and disclosure of personal health information under the HIPAA regulations, the review, approval,

and other processes outlined above apply except that IRB review and approval may not be required per study site policies.

8.3 INSTITUTIONAL REVIEW BOARD OR ETHICS COMMITTEE

This protocol, the Informed Consent Forms, any information to be given to the patient, and relevant supporting information must be submitted to the IRB/EC by the Principal Investigator and reviewed and approved by the IRB/EC before the study is initiated. In addition, any patient recruitment materials must be approved by the IRB/EC.

The Principal Investigator is responsible for providing written summaries of the status of the study to the IRB/EC annually or more frequently in accordance with the requirements, policies, and procedures established by the IRB/EC. Investigators are also responsible for promptly informing the IRB/EC of any protocol amendments (see Section 9.6).

In addition to the requirements for reporting all adverse events to the Sponsor, investigators must comply with requirements for reporting serious adverse events to the local health authority and IRB/EC. Investigators may receive written IND safety reports or other safety-related communications from the Sponsor. Investigators are responsible for ensuring that such reports are reviewed and processed in accordance with health authority requirements and the policies and procedures established by their IRB/EC, and archived in the site's study file.

8.4 CONFIDENTIALITY

The Sponsor maintains confidentiality standards by coding each patient enrolled in the study through assignment of a unique patient identification number. This means that patient names are not included in data sets that are transmitted to any Sponsor location.

Patient medical information obtained by this study is confidential and may be disclosed to third parties only as permitted by the Informed Consent Form (or separate authorization for use and disclosure of personal health information) signed by the patient, unless permitted or required by law.

Medical information may be given to a patient's personal physician or other appropriate medical personnel responsible for the patient's welfare, for treatment purposes.

Given the complexity and exploratory nature of exploratory biomarker analyses, data derived from these analyses will generally not be provided to study investigators or patients unless required by law. The aggregate results of any conducted research will be available in accordance with the effective Roche policy on study data publication (see Section 9.5).

Data generated by this study must be available for inspection upon request by representatives of national and local health authorities, Sponsor monitors, representatives, and collaborators, and the IRB/EC for each study site, as appropriate.

8.5 FINANCIAL DISCLOSURE

Investigators will provide the Sponsor with sufficient, accurate financial information in accordance with local regulations to allow the Sponsor to submit complete and accurate financial certification or disclosure statements to the appropriate health authorities. Investigators are responsible for providing information on financial interests during the course of the study and for 1 year after completion of the study (see definition of end of study in Section 3.2).

9. STUDY DOCUMENTATION, MONITORING, AND ADMINISTRATION

9.1 STUDY DOCUMENTATION

The investigator must maintain adequate and accurate records to enable the conduct of the study to be fully documented, including, but not limited to, the protocol, protocol amendments, Informed Consent Forms, and documentation of IRB/EC and governmental approval. In addition, at the end of the study, the investigator will receive the patient data, including an audit trail containing a complete record of all changes to data.

9.2 PROTOCOL DEVIATIONS

The investigator should document and explain any protocol deviations. The investigator should promptly report any deviations that might have an impact on patient safety and data integrity to the Sponsor and to the IRB/EC in accordance with established IRB/EC policies and procedures. The Sponsor will review all protocol deviations and assess whether any represent a serious breach of Good Clinical Practice guidelines and require reporting to health authorities. As per the Sponsor’s standard operating procedures, prospective requests to deviate from the protocol, including requests to waive protocol eligibility criteria, are not allowed.

9.3 SITE INSPECTIONS

Site visits will be conducted by the Sponsor or an authorized representative for inspection of study data, patients’ medical records, and eCRFs. The investigator will permit national and local health authorities; Sponsor monitors, representatives, and collaborators; and the IRBs/ECs to inspect facilities and records relevant to this study.

9.4 ADMINISTRATIVE STRUCTURE

This trial will be sponsored and managed by F. Hoffmann-La Roche Ltd. The Sponsor will provide clinical operations management, data management, and medical monitoring.

Approximately 40 sites globally will participate to enroll approximately 229-262 patients. Enrollment will occur through an IxRS.

Central facilities will be used for certain study assessments throughout the study (e.g., specified laboratory tests, biomarker and PK analyses), as specified in Section 4.5. Accredited local laboratories will be used for routine monitoring; local laboratory ranges will be collected.

An IMC will be employed to monitor and evaluate patient safety throughout the study. Tumor response and progression will be evaluated by an IRC and the investigator.

9.5 PUBLICATION OF DATA AND PROTECTION OF TRADE SECRETS

Regardless of the outcome of a trial, the Sponsor is dedicated to openly providing information on the trial to healthcare professionals and to the public, at scientific congresses, in clinical trial registries, and in peer-reviewed journals. The Sponsor will comply with all requirements for publication of study results. Study data may be shared with others who are not participating in this study (see Section 8.4 for details), and redacted Clinical Study Reports and other summary reports will be made available upon request. For more information, refer to the Roche Global Policy on Sharing of Clinical Study information at the following web site:

https://www.roche.com/innovation/process/clinical-trials/data-sharing/

The results of this study may be published or presented at scientific congresses. For all clinical trials in patients involving an IMP for which a marketing authorization application has been filed or approved in any country, the Sponsor aims to submit a journal manuscript reporting primary clinical trial results within 6 months after the availability of the respective Clinical Study Report. In addition, for all clinical trials in patients involving an IMP for which a marketing authorization application has been filed or approved in any country, the Sponsor aims to publish results from analyses of additional endpoints and exploratory data that are clinically meaningful and statistically sound.

The investigator must agree to submit all manuscripts or abstracts to the Sponsor prior to submission for publication or presentation. This allows the Sponsor to protect proprietary information and to provide comments based on information from other studies that may not yet be available to the investigator.

In accordance with standard editorial and ethical practice, the Sponsor will generally support publication of multicenter trials only in their entirety and not as individual center data. In this case, a coordinating investigator will be designated by mutual agreement.

Authorship will be determined by mutual agreement and in line with International Committee of Medical Journal Editors authorship requirements. Any formal publication of the study in which contribution of Sponsor personnel exceeded that of conventional

monitoring will be considered as a joint publication by the investigator and the appropriate Sponsor personnel.

Any inventions and resulting patents, improvements, and/or know-how originating from the use of data from this study will become and remain the exclusive and unburdened property of the Sponsor, except where agreed otherwise.

9.6 PROTOCOL AMENDMENTS

Any protocol amendments will be prepared by the Sponsor. Protocol amendments will be submitted to the IRB/EC and to regulatory authorities in accordance with local regulatory requirements.

Approval must be obtained from the IRB/EC and regulatory authorities (as locally required) before implementation of any changes, except for changes necessary to eliminate an immediate hazard to patients or changes that involve logistical or administrative aspects only (e.g., change in Medical Monitor or contact information).

10. REFERENCES

Aaronson NK, Ahmedzai S, Bergman B, et al. The European Organization for Research and Treatment of Cancer QLQ-C30: a quality-of-life instrument for use in international clinical trials in oncology. J Natl Cancer Inst 1993;85:365−76.

Abramson JS, Palomba ML, Gordon LI, et al. Lisocabtagene maraleucel for patients with relapsed or refractory large B-cell lymphomas (TRANSCEND NHL 001): a multicentre seamless design study. Lancet 2020;396:839−52.

Agresti A. Categorical data analysis. 2nd edition. New York:Wiley, 2002.

Aliqopa™ (copanlisib) U.S. Package Insert, Bayer HealthCare Pharmaceuticals Inc. 2017 [updated September 2017; cited 31 October 2017]. Available from: http://labeling.bayerhealthcare.com/html/products/pi/Aliqopa_PI.pdf.

Armitage JO, Weisenburger DD. New approach to classifying non-Hodgkin's lymphomas: clinical features of the major histologic subtypes. Non-Hodgkin's Lymphoma Classification Project. J Clin Oncol 1998;16:2780–95.

Assouline S, Buccheri V, Delmer A, et al. Pharmacokinetics, safety, and efficacy of subcutaneous versus intravenous rituximab plus chemotherapy as treatment for chronic lymphocytic leukaemia (SAWYER): a phase 1b, open-label, randomised controlled non-inferiority trial. Lancet Haematol 2016;3:128–38.

Atwell S, Ridgway JB, Wells JA, et al. Stable heterodimers from remodeling the domain interface of a homodimer using a phage display library. J Mol Biol 1997;270:26–35.

Bargou R, Leo E, Zugmaier G, et al. Tumor regression in cancer patients by very low doses of a T-cell-engaging antibody. Science 2008;321:974–7.

Barrans S, Crouch S, Smith A, et al. Rearrangement of MYC is associated with poor prognosis in patients with diffuse large B-cell lymphoma treated in the era of rituximab. J Clin Oncol 2010;28:3360–5.

Blincyto® (blinatumomab) FDA Drug Approval Package, Amgen Inc. 2014 [updated November 2017; cited 16 April 2018]. Available from: https://www.accessdata.fda.gov/scripts/cder/daf/index.cfm?event=BasicSearch.process.

Blincyto® (blinatumomab) U.S. Package Insert, Amgen Inc. 2014 [updated March 2018; cited 16 April 2018]. Available from: http://pi.amgen.com/~/media/amgen/repositorysites/pi-amgen-com/blincyto/blincyto_pi_hcp_english.ashx.

Brice P, Bastion Y, Lepage E, et al. Comparison in low-tumor-burden follicular lymphomas between an initial no-treatment policy, prednimustine, or interferon-alfa: a randomized study from the Groupe d'Etude des Lymphomes Folliculaires. J Clin Oncol 1997;15:1110.

Brookmeyer R, Crowley J. A confidence interval for the median survival time. Biometrics1982;38:29–41.

Brownstein CM, Adriaens L, Bannerji R, et al. First-in-human study assessing safety and tolerability of REGN1979, a novel CD20 × CD3 bispecific antibody, in patients with CD20+ B-cell malignancies previously treated with anti-CD20 therapy. J Clin Oncol 2015;33(15 Suppl):TPS3089.

Caimi PF, Ai W, Alderuccio JP, et al. Loncastuximab tesirine in relapsed or refractory diffuse large B-cell lymphoma (LOTIS-2): a multicentre, open-label, single-arm, phase 2 trial. Lancet Oncol 2021;22:790–800.

Cairo MS, Coiffier B, Reiter A, et al. on behalf of the TLS Expert Panel. Recommendations for the evaluation of risk and prophylaxis of tumour lysis syndrome (TLS) in adults and children with malignant diseases: an expert TLS panel consensus. Br J Haematol 2010;149:578–86.

Carson KR, Evens AM, Richey EA, et al. Progressive multifocal leukoencephalopathy after rituximab therapy in HIV-negative patients: a report of 57 cases from the Research on Adverse Drug Events and Reports project. Blood 2009;113:4834–40.

Carter GC, Liepa AM, Zimmermann AH, et al. Validation of the Functional Assessment of Cancer Therapy–Lymphoma (FACT-LYM) in patients with relapsed/refractory mantle cell lymphoma. Blood 2008;112:2376.

Cheah CY, Chihara D, Romaguera JE, et al. Patients with mantle cell lymphoma failing ibrutinib are unlikely to respond to salvage chemotherapy and have poor outcomes. Ann Oncol 2015;26:175–79.

Chen DS, Mellman I. Oncology meets immunology: the cancer-immunity cycle. Immunity 2013;39:1–10.

Chen F, Teachey DT, Pequignot E, et al. Measuring IL-6 and sIL-6R in serum from patients treated with tocilizumab and/or siltuximab following CAR T cell therapy. J Immunol Methods 2016;434:1–8.

Chen Y, Samineni D, Mukadam S, et al. Physiologically based pharmacokinetic modeling as a tool to predict drug interactions for antibody-drug conjugates. Clin Pharmacokinet 2015;54:81–93.

Cheson BD, Ansell S, Schwartz L, et al. Refinement of the Lugano Classification lymphoma response criteria in the era of immunomodulatory therapy. Blood 2016;128:2489–96.

Cheson BD, Chua N, Mayer J, et al. Overall survival benefit in patients with rituximab-refractory indolent non-Hodgkin lymphoma who received obinutuzumab plus bendamustine induction and obinutuzumab maintenance in the GADOLIN study. J Clin Oncol 2018:JCO2017763656. doi: 10.1200/JCO.2017.76.3656. [Epub ahead of print].

Cheson BD, Fisher IR, Barrington SF, et al. Recommendations for initial evaluation, staging and response assessment of Hodgkin and non-Hodgkin lymphoma: the Lugano Classification. J Clin Oncol 2014;32:1–9.

Cheson BD, Horning SJ, Coiffier B, et al. Report of an international workshop to standardize response criteria for non-Hodgkin's lymphomas. NCI Sponsored International Working Group. J Clin Oncol 1999;17:1244.

Cheson BD, Pfistner B, Juweid ME, et al. Revised response criteria for malignant lymphoma. J Clin Oncol 2007;25:579–86.

Clopper C; Pearson E S. The use of confidence or fiducial limits illustrated in the case of the binomial. Biometrika 1934;26:404–13.

Coiffier B, Altman A, Pui C, et al. Guidelines for the management of pediatric and adult tumor lysis syndrome: an evidence–based review. J Clin Oncol 2008;26:2767–78.

Coiffier B, Lepage E, Brière J, et al. CHOP chemotherapy plus rituximab compared with CHOP alone in elderly patients with diffuse large B-cell lymphoma. N Engl J Med 2002;346:235–42.

Coiffier B, Thieblemont C, Van Der Neste E, et al. Long-term outcome of patients in the LNH-98.5 Trial, the first randomized study comparing rituximab-CHOP to standard CHOP chemotherapy in DLBCL patients: a study by the Groupe d'Etudes des Lymphomes de l'Adulte. Blood 2010;116:2040–5.

Dang NH, Ogura M, Castaigne S, et al. Randomized, phase 3 trial of inotuzumab ozogamicin plus rituximab versus chemotherapy plus rituximab for relapsed/refractory aggressive B-cell non-Hodgkin lymphoma. Br J Haematol 2017. doi: 10.1111/bjh.14820. [Epub ahead of print].

Davila ML, Riviere I, Wang X, et al. Efficacy and toxicity management of 19-28z CAR T cell therapy in B cell acute lymphoblastic leukemia. Sci Transl Med 2014;6:224ra25.

Doessegger L, Banholzer ML. Clinical development methodology for infusion-related reactions with monoclonal antibodies. Clin Transl Immunology 2015;4:e39.

Dornan D, Bennett F, Chen Y, et al. Therapeutic potential of an anti-CD79b antibody-drug conjugate, anti-CD79b-vc-MMAE, for the treatment of non-Hodgkin lymphoma. Blood 2009;114:2721–9.

Doronina SO, Toki BE, Torgov MY, et al. Development of potent monoclonal antibody auristatin conjugates for cancer therapy. Nat Biotechnol 2003;21:778–84.

Dreyling M, Santoro A, Mollica L, et al. Phosphatidylinositol 3-kinase inhibition by copanlisib in relapsed or refractory indolent lymphoma. J Clin Oncol 2017;35:3898–3905.

Dunleavy K, Fanale M, LaCasce A, et al. Preliminary report of a multicenter prospective phase II study of DA-EPOCH-R in MYC-rearranged aggressive B-cell lymphoma. Blood 2014;124:395.

Edelman GM, Cunningham BA, Gall WE, et al. The covalent structure of an entire gammaG immunoglobulin molecule. Proc Natl Acad Sci USA 1969;63:78–85.

Egbuniwe IU, Karagiannis SN, Nestle FO, et al. Revisiting the role of B cells in skin immune surveillance. Trends Immunol 2015;36:102–11.

Elitek® (rasburicase) U.S. Package Insert, sanofi-aventis U.S. LLC. 2002 [updated September 2017; cited 16 April 2018]. Available from: http://products.sanofi.us/elitek/elitek.html.

Fardet L, Galicier L, Lambotte O, et al. Development and validation of the Hscore, a score for the diagnosis of reactive hemophagocytic syndrome. Arthritis Rheumatol 2014;66:2613–20.

Feugier P, Van Hoof A, Sebban C, et al. Long-term results of the R-CHOP study in the treatment of elderly patients with diffuse large B-cell lymphoma: a study by the Groupe d'Etude des Lymphomes de l'Adulte. J Clin Oncol 2005;23:4117–26.

Flinn IW, Miller CB, Ardeshna KM, et al. DYNAMO: A Phase II Study of Duvelisib (IPI-145) in Patients With Refractory Indolent Non-Hodgkin Lymphoma. J Clin Oncol 2019;37:912–22.

Fowler NH, Samaniego F, Jurczak W, et al. Umbralisib, a Dual PI3Kδ/CK1ε Inhibitor in Patients With Relapsed or Refractory Indolent Lymphoma. J Clin Oncol 2021;39:1609–18.

Friedberg JW. Relapsed/refractory diffuse large B-cell lymphoma. Hematology Am Soc Hematol Educ Program 2011;2011:498–505.

Gazyva® (obinutuzumab) U.S. Package Insert, Genentech Inc. 2013 [updated November 2017; cited 16 April 2018]. Available from: http://www.gene.com/download/pdf/gazyva_prescribing.pdf.

Gisselbrecht C, Glass B, Mounier N, et al. Salvage regimens with autologous transplantation for relapsed large B-cell lymphoma in the rituximab era. J Clin Oncol 2010;28:4184–90.

Goebeler ME, Knop S, Viardot A, et al. Bispecific T-cell engager (BiTE) antibody construct blinatumomab for the treatment of patients with relapsed/refractory non-Hodgkin lymphoma: final result from a phase I study. J Clin Oncol 2016;34:1104–11.

Gopal AK, Kahl BS, de Vos S, et al. PI3Kδ inhibition by idelalisib in patients with relapsed indolent lymphoma. N Engl J Med 2014;370:1008–18.

Grupp SA, Kalos M, Barrett D, et al. Chimeric antigen receptor–modified T cells for acute lymphoid leukemia. New Engl J Med 2013;368:1509–18.

Hashemi-Sadraei N, Vejpongsa P, Baljevic M, et al. Epstein-Barr virus-related hemophagocytic lymphohistiocytosis: hematologic emergency in the critical care setting. Case Rep Hematol 2015;2015:491567.

Hejblum G, Lambotte O, Galicier L, et al. A web-based delphi study for eliciting helpful criteria in the positive diagnosis of hemophagocytic syndrome in adult patients. PloS One. 2014;9:e94024.

Henter JI, Horne AC, Arico M, et al. HLH-2004: diagnostic and therapeutic guidelines for hemophagocytic lymphohistiocytosis. Pediatr Blood Cancer 2007;48:124–31.

Herdman M, Gudex C, Lloyd A, et al. Development and preliminary testing of the new five-level version of EQ-5D (EQ-5D-5L). Qual Life Res 2011;20:1727–36.

Hiddemann W, Kneba M, Dreyling M, et al. Frontline therapy with rituximab added to the combination of cyclophosphamide, doxorubicin, vincristine, and prednisone (CHOP) significantly improves the outcome for patients with advanced-stage follicular lymphoma compared with therapy with CHOP alone: results of a prospective randomized study of the German Low-Grade Lymphoma Study Group. Blood 2005;106:3725–32.

Hlubocky FJ, Webster K, Cashy J, et al. The development and validation of a measure of health-related quality of life for non-Hodgkin’s lymphoma: the Functional Assessment of Cancer Therapy—Lymphoma (FACT-Lym). Lymphoma 2013;2013:1–9.

Howard SC, Jones DP, Pui CH. The tumor lysis syndrome. New Engl J Med 2011;364:1844–54.

Huang HQ, Brady MF, Cella D, et al. Validation and reduction of FACT/GOG-Ntx subscale for platinum/paclitaxel-induced neurologic symptoms: a gynecologic oncology group study. Int J Gynecol Cancer 2007;17:387–93.

Jacobson C, Chavez JC, Sehgal AR, et al. 700 Primary Analysis of Zuma-5: A Phase 2 Study of Axicabtagene Ciloleucel (Axi-Cel) in Patients with Relapsed/Refractory (R/R) Indolent Non-Hodgkin Lymphoma (iNHL) [abstract]. 62nd ASH Annual Meeting and Exposition 2020.

Jagtap N, Sharma M, Rajesh G, et al. Hemophagocytic lymphohistiocytosis masquerading as acute liver failure: a single center experience. J Clin Exp Hepatol 2017;7:184–9.

Janssen MF, Pickard AS, Golicki D, et al. Measurement properties of the EQ-5D-5L compared to the EQ-5D-3L across eight patient groups: a multi-country study. Qual Life Res 2013;22:1717–27.

Jerkeman M, Kaasa S, Hjermstad M, et al. Health-related quality of life and its potential prognostic implications in patients with aggressive lymphoma: a Nordic Lymphoma Group Trial. Med Oncol 2001;18:85–94.

Kabat EA, Wu TT, Perry HM, et al. Sequences of proteins of immunological interest. 5th ed. Bethesda, Maryland: Public Health Service, National Institutes of Health, 1991.

Kalakonda N, Maerevoet M, Cavallo F, et al. Selinexor in patients with relapsed or refractory diffuse large B-cell lymphoma (SADAL): a single-arm, multinational, multicentre, open-label, phase 2 trial. Lancet Haematol 2020;7:e511-22.

Kaplan EL, Meier P. Nonparametric estimation from incomplete observations. J Am Stat Assoc 1958;53:457–81.

Kochenderfer JN, Dudley ME, Feldman SA, et al. B-cell depletion and remissions of malignancy along with cytokine-associated toxicity in a clinical trial of anti-CD19 chimeric-antigen-receptor–transduced T cells. Blood 2012;119:2709–20.

Kochenderfer JN, Dudley ME, Kassim SH, et al. Chemotherapy-refractory diffuse large B-cell lymphoma and indolent B-cell malignancies can be effectively treated with autologous T cells expressing an anti-CD19 chimeric antigen receptor. J Clin Oncol 2015;33:540–9.

Koren E, Smith HW, Shores E, et al. Recommendations on risk-based strategies for detection and characterization of antibodies against biotechnology products. J Immunol Methods 2008;333:1–9.

Kymriah™ (tisagenlecleucel) U.S. Package Insert, Novartis Pharmaceuticals Corporation. 2017 [updated August 2017; cited 4 April 2018]. Available from: https://www.pharma.us.novartis.com/sites/www.pharma.us.novartis.com/files/kymriah.pdf.

La Rosee P. Treatment of hemophagocytic lymphohistiocytosis in adults. Hematology Am Soc Hematol Educ Program 2015;2015:190–6.

Lee DW, Gardner R, Porter DL, et al. Current concepts in the diagnosis and management of cytokine release syndrome. Blood 2014;124:188–95.

Lee DW, Santomasso BD, Locke FL et al. ASTCT consensus grading for cytokine release syndrome and neurologic toxicity associated with immune effector cells. Biol Blood Marrow Transplant 2019;25:625–38.

Lenz G, Wright G, Dave SS, et al. Stromal gene signature in large B-cell lymphomas. N Engl J Med 2008;359:2313–23.

Leonard JP, Jung SH, Johnson J, et al. Randomized trial of lenalidomide alone versus lenalidomide plus rituximab in patients with recurrent follicular lymphoma: CALGB 50401 (Alliance). J Clin Oncol 2015;33:3635–40.

Lim MY, Fedoriw Y, Ramanayake H, et al. Epstein-Barr virus reactivation and hemophagocytic lymphohistiocytosis in a patient with chronic lymphocytic leukemia. Leuk Lymphoma 2014;55:2938–41.

Lin S, Li Y, Long J, et al. Acute liver failure caused by hemophagocytic lymphohistiocytosis in adults: a case report and review of the literature. Medicine (Baltimore). 2016;95:e5431.

Lossos IS, Gascoyne RD. Transformation of follicular lymphoma. Best Pract Res Clin Haematol 2011;24:147–63.

Marcus R, Davies A, Ando K, et al. Obinutuzumab for the first-line treatment of follicular lymphoma. N Engl J Med 2017;377:1331–44.

Martin P, Maddocks K, Leonard JP, et al. Postibrutinib outcomes in patients with mantle cell lymphoma. Blood 2016;127:1559–63.

Maude SL, Frey N, Shaw PA, et al. Chimeric antigen receptor T cells for sustained remissions in leukemia. New Engl J Med 2014;371:1507–17.

McClain KL, Eckstein O. Clinical features and diagnosis of hemophagocytic lymphohistiocytosis.Up to Date [resource on the Internet]. [updated October 2019; cited: 16 October 2019]. Available from: https://www.uptodate.com/contents/clinical-features-and-diagnosisof-hemophagocytic-lymphohistiocytosis.

McCulloch R, Visco C, Eyre TA, et al. Efficacy of R-BAC in relapsed, refractory mantle cell lymphoma post BTK inhibitor therapy. Br J Haematol 2020;189:684–88.

Merli F, Luminari S, Tucci A, et al. Simplified geriatric assessment in older patients with diffuse large B-cell lymphoma: The prospective elderly project of the fondazione italiana linfomi. J. Clin. Oncol 2021;39(11):1214–22.

Merli F, Luminari S, Rossi G, et al. Outcome of frail elderly patients with diffuse large B-cell lymphoma prospectively identified by Comprehensive Geriatric Assessment: results from a study of the Fondazione Italiana Linfomi. Leuk Lymphoma 2014;55(1):38-43.

Miyoshi H, Arakawa F, Sato K, et al. Comparison of CD20 expression in B-cell lymphoma between newly diagnosed, untreated cases and those after rituximab treatment. Cancer Sci 2012;103:1567–73.

Morschhauser F, Flinn IW, Advani R, et al. Polatuzumab vedotin or pinatuzumab vedotin plus rituximab in patients with relapsed or refractory non-Hodgkin lymphoma: final results from a phase 2 randomised study (ROMULUS). Lancet Haematol 2019;6:e254–65.

Morschhauser F, Tilly H, Chaidos A, et al. Tazemetostat for patients with relapsed or refractory follicular lymphoma: an open-label, single-arm, multicentre, phase 2 trial. Lancet Oncol 2020;11:1433–42.

Mounier N, El Gnaoui T, Tilly H, et al. Rituximab plus gemcitabine and oxaliplatin in patients with refractory/relapsed diffuse large B-cell lymphoma who are not candidates for high-dose therapy. A phase II Lymphoma Study Association trial. Haematologica 2013;98:1726–31.

Mueller SN, Zaid A, Carbone FR. Tissue-resident T cells: dynamic players in skin immunity. Front Immunol. 2014;5:332.

Nagorsen D, Kufer P, Baeuerle PA, et al. Blinatumomab: a historical perspective. Pharmacol Ther 2012;136:334–42.

National Institutes of Health. Recombinant DNA Advisory Committee (RAC). Cytokine release syndrome after T cell immunotherapy meeting [videocast on the Internet]. 10 June 2015 [cited 30 January 2017]. Available from: https://videocast.nih.gov/summary.asp?Live=16420&bhcp=1.

[NCCN] National Comprehensive Cancer Network. NCCN clinical practice guidelines in oncology (NCCN Guidelines): B-cell lymphomas, version 2 [resource on the Internet]. 2018 [cited 3 March 2018]. Available from: https://www.nccn.org/professionals/physician_gls/recently_updated.aspx.

Neelapu SS, Locke FL, Bartlett NL, et al. Kte-C19 (anti-CD19 CAR T cells) induces complete remissions in patients with refractory diffuse large B-cell lymphoma (DLBCL): results from the pivotal phase 2 Zuma-1. Blood 2016;128:LBA-6.

Neelapu SS, Tummala S, Kebriaei P, et al. Chimeric antigen receptor T-cell therapy–assessment and management of toxicities. Nat Rev Clin Oncol 2018;15:47–62.

ODAC FDA Briefing Document PI3K Inhibitors Hematologic Malignancies [Cited April 21, 2022]. Available from: https://www.fda.gov/media/157762/download.

Olejniczak SH, Stewart CC, Donohue K, et al. A quantitative exploration of surface antigen expression in common B-cell malignancies using flow cytometry. Immunol Invest 2006;35:93–114.

Osoba D, Rodrigues G, Myles J, et al. Interpreting the significance of changes in health-related quality-of-life scores. J Clin Oncol 1998;16:139–44.

Palanca-Wessels MCA, Czuczman M, Salles G, et al. Safety and activity of the anti-CD79B antibody–drug conjugate polatuzumab vedotin in relapsed or refractory B-cell non-Hodgkin lymphoma and chronic lymphocytic leukaemia: a phase 1 study. Lancet Oncology 2015;16:704–15.

Panelli MC, White R, Foster M, et al. Forecasting the cytokine storm following systemic interleukin (IL)-2 administration. J Transl Med 2004;2:17.

Parmelee PA, Thuras PD, Katz IR, et al. Validation of the Cumulative Illness Rating Scale in a geriatric residential population. J Am Geriatr Soc. 1995;43(2):130–7.

Pettengell R, Coiffier B, Narayanan G, et al. Pixantrone dimaleate versus other chemotherapeutic agents as a single-agent salvage treatment in patients with relapsed or refractory aggressive non-Hodgekin lymphoma: a phase 3, multicenter, open-label, randomised trial. Lancet Oncol 2012;13:696–706.

Pettengell R, Donatti C, Hoskin P, et al. The impact of follicular lymphoma on health-related quality of life. Ann Oncol 2008;19:570–6.

Pfeifer M, Zheng B, Erdmann T, et al. Anti-CD22 and anti-CD79B antibody drug conjugates are active in different molecular diffuse large B-cell lymphoma subtypes. Leukemia. 2015;29:1578–86.

Pfreundschuh M, Kuhnt E, Trümper L, et al. CHOP-like chemotherapy with or without rituximab in young patients with good-prognosis diffuse large-B-cell lymphoma: 6-year results of an open-label randomised study of the MabThera International Trial (MinT) Group. Lancet Oncol 2011;12:1013–22.

Phillips T., Brunvand M., Chen A., et al. Polatuzumab vedotin combined with obinutuzumab for patients with relapsed or refractory non-Hodgkin lymphoma: preliminary safety and clinical activity of a phase Ib/II study. Blood 2016;128:622.

Polson AG, Calemine Fenaux J, Chan P, et al. Antibody drug conjugates for the treatment of non-Hodgkin's lymphoma: target and linker drug selection. Cancer Res 2009;69:2358–64.

Polson AG, Yu SF, Elkins K, et al. Antibody-drug conjugates targeted to CD79 for the treatment of non-Hodgkin lymphoma. Blood 2007;110:616–23.

Porter DL, Hwang WT, Frey NV, et al. Chimeric antigen receptor T cells persist and induce sustained remissions in relapsed refractory chronic lymphocytic leukemia. Sci Transl Med 2015;7:303ra139.

Porter DL, Levine BL, Kalos M, et al. Chimeric antigen receptor-modified T cells in chronic lymphoid leukemia. N Engl J Med 2011;365:725–33.

Ramos-Casals M, Brito-Zerón P, López-Guillermo A, et al. Adult haemophagocytic syndrome. Lancet 2014;383:1503–16.

Rath J, Geisler C, Christiansen CB, et al. Epstein-Barr virus reactivation is a potentially severe complication in chronic lymphocytic leukemia patients with poor prognostic biological markers and fludarabine refractory disease. Haematologica 2008;93:1424–6.

Riegler LL, Jones GP, Lee DW. Current approaches in the grading and management of cytokine release syndrome after chimeric antigen receptor T-cell therapy. Ther Clin Risk Manag. 2019;15:323–35.

Rituxan® (rituximab) U.S. Package Insert, Genetech Inc. 1997 [updated April 2018; cited 16 April 2018]. Available from: http://www.gene.com/download/pdf/rituxan_prescribing.pdf.

Rivière S, Galicier L, Coppo P, et al. Reactive hemophagocytic syndrome in adults: a retrospective analysis of 162 patients. Am J Med 2014;127:1118–25.

Rosenberg AS, Worobec AS. A risk-based approach to immunogenicity concerns of therapeutic protein products: Part III: effects of manufacturing changes in

immunogenicity and the utility of animal immunogenicity studies. Biopharm International 2005;18:32–36.

Rosenberg AS, Worobec AS. A risk-based approach to immunogenicity concerns of therapeutic protein products: Part I: considering consequences of the immune response to a protein. Biopharm International 2004a;17:22–26.

Rosenberg AS, Worobec AS. A risk-based approach to immunogenicity concerns of therapeutic protein products: Part II: considering host-specific and product-specific factors impacting immunogenicity. Biopharm International 2004b;17:34–42.

Salles G, Duell J, González Barca E, et al. Tafasitamab plus lenalidomide in relapsed or refractory diffuse large B-cell lymphoma (L-MIND): a multicentre, prospective, single-arm, phase 2 study. Lancet Oncol 2020;21:978–88.

Salles GA, Trotman J, Lill M, et al. Pseudo-progression among patients with follicular lymphoma treated with ibrutinib in the phase 2 DAWN study [abstract]. Blood 2016;128:2980.

Salvi F, Miller MD, Grilli A, at al. A manual of guidelines to score the modified cumulative illness rating scale and its validation in acute hospitalized elderly patients. J Am Geriatr Soc. 2008;56(10):1926–31.

Savage KJ, Johnson NA, Ben-Neriah S, et al. MYC gene rearrangements are associated with a poor prognosis in diffuse large B-cell lymphoma patients treated with R-CHOP chemotherapy. Blood 2009;114:3533–7.

Schram AM, Berliner N. How I treat hemophagocytic lymphohistiocytosis in the adult patient. Blood 2015;125:2908–14.

Schuster SJ, Bishop MR, Tam CS, et al. Tisagenlecleucel in Adult Relapsed or Refractory Diffuse Large B-Cell Lymphoma. N Engl J Med 2019;380:45–56.

Sehn LH, Chua N, Mayer J, et al. Obinutuzumab plus bendamustine versus bendamustine monotherapy in patients with rituximab-refractory indolent non-Hodgkin lymphoma (GADOLIN): a randomised, controlled, open-label, multicentre, phase 3 trial. Lancet Oncol 2016;17:1081–93.

Sehn LH, Herrera AF, Flowers CR. et al. Polatuzumab Vedotin in Relapsed or Refractory Diffuse Large B-Cell Lymphoma. Journal of Clinical Oncology 2020 38:2, 155-165.

Smith EJ, Olson K, Haber LJ, et al. A novel, native-format bispecific antibody triggering T-cell killing of B-cells is robustly active in mouse tumor models and cynomolgus monkeys. Sci Rep 2015a;5:17943.

Smith TJ, Bohlke K, Lyman GH, et al. Recommendations for the use of WBC growth factors: American Society of Clinical Oncology Clinical Practice Guideline Update. J Clin Oncol 2015b;33:3199–212.

Spiess C, Merchant M, Huang A, et al. Bispecific antibodies with natural architecture produced by co-culture of bacteria expressing two distinct half-antibodies. Nat Biotechnol 2013:31:753–8.

Sweetenham JW. Diffuse large B-cell lymphoma: risk stratification and management of relapsed disease. Hematology Am Soc Hematol Educ Program 2005;252–9.

Taplitz RA, Kennedy EB, Bow EJ, et al. Outpatient management of fever and neutropenia in adults treated for malignancy: American Society of Clinical Oncology and Infectious Diseases Society of America Clinical Practice Guideline Update. J Clin Oncol 2018 20 February:JCO2017776211. doi: 10.1200/JCO.2017.77.6211. [Epub ahead of print].

Teachey DT, Rheingold SR, Maude SL, et al. Cytokine release syndrome after blinatumomab treatment related to abnormal macrophage activation and ameliorated with cytokine-directed therapy. Blood 2013:121:5154–7.

Tilly H, Morschhauser F, Sehn L, et al. The POLARIX Study: Polatuzumab vedotin with rituximab, cyclophosphamide, doxorubicin, and prednisone (pola-R-CHP) versus rituximab, cyclophosphamide, doxorubicin, vincristine and prednisone (R–CHOP) therapy in patients with previously untreated diffuse large B-cell lymphoma. Blood 2021;138:2;LBA-1.

Thall PF, Simon R. Practical Bayesian guidelines for phase IIB clinical trials. Biometrics. 1994 Jun;50:337–49.

Tholstrup D, Brown Pde N, Jurlander J, et al. Quality of life in patients with diffuse large B-cell lymphoma treated with dose-dense chemotherapy is only affected temporarily. Leuk Lymphoma 2011;52:400–8.

Topp MS, Kufer P, Gokbuget N, et al. Targeted therapy with the T-cell-engaging antibody blinatumomab of chemotherapy-refractory minimal residual disease in B-lineage acute lymphoblastic leukemia patients results in high response rate and prolonged leukemia-free survival. J Clin Oncol 2011;29:2493–8.

Trotman J, Fournier M, Lamy T, et al. Positron emission tomography-computed tomography (PET-CT) after induction therapy is highly predictive of patient outcome in follicular lymphoma: analysis of PET-CT in a subset of PRIMA trial participants. J Clin Oncol 2011;19:3194–200.

Vallurupalli M and Berliner N. Emapalumab for the treatment of relapsed/refractory hemophagocytic lymphohistiocytosis. Blood 2019;134(21):1783-1786.

van Oers MH, Klasa R, Marcus RE, et al. Rituximab maintenance improves clinical outcome of relapsed/resistant follicular non-Hodgkin lymphoma in patients both with and without rituximab during induction: results of a prospective randomized phase 3 intergroup trial. Blood 2006;108:3295–301.

van Oers MH, Van Glabbeke M, Giurgea L, et al. Rituximab maintenance treatment of relapsed/resistant follicular non-Hodgkin's lymphoma: long-term outcome of the

EORTC 20981 phase III randomized intergroup study. J Clin Oncol 2010;28:2853–8.

Varghese B, Menon J, Rodriguez L, et al. A novel CD20 × CD3 bispecific fully human antibody induces potent anti-tumor effects against B cell lymphoma in mice. Blood 2014;124:4501.

Viardot A, Goebeler ME, Hess G, et al. Phase 2 study of the bispecific T-cell engager (BiTE) antibody blinatumomab in relapsed/refractory diffuse large B-cell lymphoma. Blood 2016;127:1410–6.

Viardot A, Goebeler ME, Hess G, et al. Treatment of relapsed/refractory diffuse large B-cell lymphoma with the bispecific t-cell engager (BiTE®) antibody construct blinatumomab: primary analysis results from an open-label, phase 2 study. Blood 2014;124:4460.

Viardot A, Goebeler ME, Noppeney R, et al. Blinatumomab monotherapy shows efficacy in patients with relapsed diffuse large B-cell lymphoma. Blood 2011;118:1637.

Viardot A, Goebeler ME, Scheele J, et al. Treatment of patients with non-Hodgkin lymphoma (NHL) with CD19/CD3 bispecific antibody blinatumomab (MT103): double-step dose increase to continuous infusion of 60 μg/m^2/d is tolerable and highly effective. Blood 2010;116:2880.

Vidal L, Gafter-Gvili A, Gurion R, et al. Bendamustine for patients with indolent B cell lymphoid malignancies including chronic lymphocytic leukaemia. Cochrane Database Syst Rev 2012;CD009045.

Wang M, Munoz J, Goy A, et al. KTE-X19 CAR T-Cell Therapy in Relapsed or Refractory Mantle-Cell Lymphoma. N Engl J Med 2020;382:1331–42.

Wang M, Schuster SJ, Phillips T, et al. Observational study of lenalidomide in patients with mantle cell lymphoma who relapsed/progressed after or were refractory/intolerant to ibrutinib (MCL-004). J Hematol Oncol 2017;10:171

Wolchok JD, Hoos A, O'Day S, et al. Guidelines for the evaluation of immune therapy activity in solid tumors: immune-related response criteria. Clin Can Res 2009;15:7412–20.

Xu-Monette Z, Wu L, Visco C, et al. Mutational profile and prognostic significance of TP53 in diffuse large B-cell lymphoma patients treated with R-CHOP: report from an International DLBCL Rituximab-CHOP Consortium Program Study. Blood 2012;120:3986–96.

Yescarta® (axicabtagene ciloleucel) U.S. Package Insert, Kite Pharma, Inc. 2017 [cited 30 October 2017]. Available from: https://www.yescarta.com/wp-content/uploads/yescarta-pi.pdf.

Young KH, Leroy K, Moller MB, et al. Structural profiles of TP53 gene mutations predict clinical outcome in diffuse large B-cell lymphoma: an international collaborative study. Blood 2008;112:3088–98.

Zhang K, Jordan MB, Marsh RA, et al. Hypomorphic mutations in PRF1, MUNC13-4, and STXBP2 are associated with adult-onset familial HLH. Blood 2011;118:5794–8.

Zhou Z, Sehn LH, Rademaker A, et al. An enhanced International Prognostic Index (NCCN-IPI) for patients with diffuse large B-cell lymphoma treated in the rituximab era. Blood 2014;123:837–42.

Zydelig® (idelalisib) U.S. Package Insert, Gilead Sciences, Inc. 2014 [updated January 2018; cited 16 April 2018]. Available from: http://www.gilead.com/~/media/Files/pdfs/medicines/oncology/zydelig/zydelig_pi.pdf.

Appendix 1 Schedule of Activities

The tables below are applicable to all patients in the Phase Ib (dose finding) and Phase II (expansion and *randomized*) portions of the study. For post-treatment follow-up/relapse assessments, see Appendix 2. For all pharmacokinetic, immunogenicity, and biomarker blood samples, see Appendix 3, (Groups A, B, and C; Arms I and J), and Appendix 4 (Arms K and L), and Appendix 5 (Arm M). For assessments related to treatment with tocilizumab (if applicable), see Appendix 6.

Screening, Cycles 1–3, and Interim Response															
	Screening [a] (– 14 days)	Cycle 1 [b]								Cycle 2 [b, c]				Cycle 3 [c]	
		D1	D2	D3	D4	D8 [ff]	D9	D11	D15 [ff]	D1	D2	D8 [ff]	D15 [ff]	D1	D2
Informed consent [d]	x														
Demographic data	x														
General medical history and baseline conditions	x														
CIRS-G [e] (for patients ≥65 years old for Arms J, L and M only)	*x*														
FLIPI, FLIPI2 (for FL), IPI (for DLBCL), and MIPI (for MCL)	x														
ECOG PS	x	x								x				x	
Assessment of transplant eligbility status [f]	x														
B symptoms [g]	x														
Concomitant medications [h]	x	x	x	x		x			x	x	x	x	x	x	x
Adverse events [i]	x	x	x	x		x			x	x	x	x	x	x	x
Vital signs [j]	x	x				x			x	x		x	x	x	
Height, BSA, and weight [k]	x	x								x				x	
Complete physical and neurologic examination [l]	x														

Screening, Cycles 1–3, and Interim Response (cont.)															
	Screening [a] (– 14 days)	Cycle 1 [b]								Cycle 2 [b, c]				Cycle 3 [c]	
		D1	D2	D3	D4	D8 [ff]	D9	D11	D15 [ff]	D1	D2	D8 [ff]	D15 [ff]	D1	D2
Targeted physical examination [m]		x				x			x	x		x	x	x	
Single 12-lead ECG [n]	x														
PET-CT [o]	x														
Bone marrow aspirate and biopsy [p]	x														
Tumor biopsy for biomarkers [q]	x								See footnote [q]						
Blood sample for RBR (optional) [r]		x													
EQ-5D-5L (Arm K) [s]		x								x					
**Study Drug Administration**															
Group A															
Polatuzumab vedotin		x								x				x	
Mosunetuzumab		x				x			x	x				x	
Group B															
Polatuzumab vedotin		x								x				x	
Mosunetuzumab						x			x	x				x	
Group C															
Polatuzumab vedotin										x				x	
Mosunetuzumab		x				x			x	x				x	

Screening, Cycles 1–3, and Interim Response (cont.)															
	Screening [a] (– 14 days)	Cycle 1 [b]								Cycle 2 [b, c]				Cycle 3 [c]	
		D1	D2	D3	D4	D8 [ff]	D9	D11	D15 [ff]	D1	D2	D8 [ff]	D15 [ff]	D1	D2
Arms I, J, and K															
Polatuzumab vedotin		Follow schedule from either Group A, B, or C (selected from dose-finding phase).													
Mosunetuzumab															
Arm L															
Polatuzumab vedotin		x								x				x	
Mosunetuzumab		x				x			x	x				x	
Arm M															
Polatuzumab vedotin		x								x				x	
Rituximab		x								x				x	
**Local Labs**															
Peripheral blood smear and/or flow cytometry [ee]	x														
HBV, HCV, and HIV [t]	x														
Blood for EBV and CMV titer by PCR [u]	x									x					
Hematology [v]	x	x				x			x	x		x	x	x	
Chemistry (serum) [w]	x	x				x			x	x		x	x	x	
Beta-2 microglobulin	x														
C-reactive protein and serum ferritin	x	x				x			x	x		x	x	x	

Appendix 1 Schedule of Activities (cont.)

Screening, Cycles 1–3, and Interim Response (cont.)															
	Screening [a] (–14 days)	Cycle 1 [b]								Cycle 2 [b, c]				Cycle 3 [c]	
		D1	D2	D3	D4	D8 [ff]	D9	D11	D15 [ff]	D1	D2	D8 [ff]	D15 [ff]	D1	D2
Coagulation (aPTT, PT, INR)	x	x				x			x	x		x	x		
Pregnancy test [x]	x									x				x	
Total IgA, IgG, IgM	x	Every 6 months (collected at closest corresponding visit)													
**Central Labs**															
Blood for viral infection test by quantitative PCR [y]	x									x					
Blood biomarker sample		See Appendix 3 and Appendix 4													
Serum PK sample		See Appendix 3 and Appendix 4													
Serum ADA samples		See Appendix 3 and Appendix 4													

Cycles 4–9 and Beyond through Study Drug Completion/Early Discontinuation								
	Cycles 4–6 [c]		Interim Response (between C4D15 and C4D21)	Cycle 7 [c]	Cycle 8 [c]	Primary Response [z]	Cycle 9 and beyond [aa]	Study Drug Completion/Early Discontinuation [bb]
	D1	D2		D1	D1		D1	
ECOG PS	x		x	x	x	x	x	x
B symptoms [g]						x		
Concomitant medications [h]	x	x	x	x	x	x	x	x
Adverse events [i]	x	x	x	x	x	x	x	x
Vital signs [j]	x		x	x	x	x	x	x
Height, BSA, and weight [k]	x			x	x	x	x	x

Cycles 4–9 and Beyond through Study Drug Completion/Early Discontinuation (cont.)								
	Cycles 4–6 [c]		Interim Response (between C4D15 and C4D21)	Cycle 7 [c]	Cycle 8 [c]	Primary Response [z]	Cycle 9 and beyond [aa]	Study Drug Completion/Early Discontinuation [bb]
	D1	D2		D1	D1		D1	
Targeted physical examination [m]	x		x	x	x	x	x	x
Single 12-lead ECG [n]								x
PET-CT [o]			x			x		
PET-CT or CT only [o]							At 9 months, 12 months, then every 6 months	
Bone marrow aspirate and biopsy [p]						x		
Tumor biopsy for biomarkers [q]								x [q]
EQ-5D-5L (Arm K) [s]	x [cc]				x		Every 3 months	x
Cycles 4–9 and Beyond through Study Drug Completion/Early Discontinuation (cont.)								
Survival and new anti-cancer therapy follow-up [dd]								Every 3 months from completion/ discontinuation of treatment

Cycles 4–9 and Beyond through Study Drug Completion/Early Discontinuation (cont.)								
	Cycles 4–6 [c]		Interim Response (between C4D15 and C4D21)	Cycle 7 [c]	Cycle 8 [c]	Primary Response [z]	Cycle 9 and beyond [aa]	Study Drug Completion/Early Discontinuation [bb]
	D1	D2		D1	D1		D1	
**Study Drug Administration**								
Group A								
Polatuzumab vedotin	x							
Mosunetuzumab	x			x	x		x [aa]	
Group B								
Polatuzumab vedotin	x							
Mosunetuzumab	x			x	x		x [aa]	
Group C								
Polatuzumab vedotin	x			x				
Mosunetuzumab	x			x	x		x [aa]	
Arms I, J, and K								

Cycles 4–9 and Beyond through Study Drug Completion/Early Discontinuation (cont.)								
	Cycles 4–6 [c]		Interim Response (between C4D15 and C4D21)	Cycle 7 [c]	Cycle 8 [c]	Primary Response [y]	Cycle 9 and beyond [aa]	Study Drug Completion/Early Discontinuation [bb]
	D1	D2		D1	D1		D1	
Polatuzumab vedotin	Follow schedule from either Group A, B, or C (selected from dose-finding phase).		Follow schedule from either Group A, B, or C (selected from dose-finding phase).				Follow schedule from either Group A, B, or C (selected from dose-finding phase).	
Mosunetuzumab								
Arm L								
Polatuzumab vedotin	x							
Mosunetuzumab	x			x	x			
Arm M								
Polatuzumab vedotin	x							
Rituximab	x			x	x			

Cycles 4–9 and Beyond through Study Drug Completion/Early Discontinuation (cont.)								
	Cycles 4–6 [c]		Interim Response (between C4D15 and C4D21)	Cycle 7 [c]	Cycle 8 [c]	Primary Response [z]	Cycle 9 and beyond [aa]	Study Drug Completion/Early Discontinuation [bb]
	D1	D2		D1	D1		D1	
**Local Labs**								
Hematology [v]	x			x	x	x	x	x
Chemistry (serum) [w]	x			x	x	x	x	x
C-reactive protein and serum ferritin	x			x	x	x	x	x
Pregnancy test [x]	x			x	x		x	x
Total IgA, IgG, IgM	Every 6 months (collected at closest corresponding visit)			Every 6 months (collected at closest corresponding visit)			Every 6 months (collected at closest corresponding visit)	
**Central Labs**								
Blood for viral infection test by quantitative PCR [y]	See footnote "y"							
Blood biomarker sample	See Appendix 3 and Appendix 4							
Serum PK sample	See Appendix 3 and Appendix 4							
Serum ADA sample	See Appendix 3 and Appendix 4							

ADA = anti-drug antibody; BSA = body surface area; C = cycle; *CIRS-G = Cumulative Illness Rating Scale-Geriatric;* CMV = cytomegalovirus; CR = complete response; CRS = cytokine release syndrome; CRu = complete response, unconfirmed; CT = computed tomography (scan); D = day; DLBCL = diffuse large B-cell lymphoma; EBV = Epstein-Barr virus; ECOG PS = Eastern Cooperative Oncology Group Performance Status; eCRF = electronic Case Report Form; EQ-5D-5L = EuroQol 5-Dimension, 5-Level (questionnaire); FL = follicular lymphoma; FLIPI = Follicular Lymphoma International Prognostic Index; GGT = gamma-glutamyl transferase; HBcAb = hepatitis B core antibody; HBsAb = hepatitis B surface antibody; HBsAg = hepatitis B surface antigen; HBV = hepatitis B virus; HCV = hepatitis C virus; IMC = Internal Monitoring Committee; IPI = International Prognostic Index; IRR = infusion-related reaction; LDH = lactate dehydrogenase; mo = month(s); PCR = polymerase chain reaction; PET = positron emission tomography (scan); PET-CT = positron emission tomography–computed tomography (scan); PK = pharmacokinetic; PR = partial response; PRO = patient reported outcome; RBR = Research Biosample Repository; wk = week.

Assessments are to be taken prior to study drug infusion/injection, unless otherwise specified. Pre-infusion laboratory samples should be drawn 0-36 hours prior to study treatment infusion/injection.
For mosunetuzumab re-treatment as single agent or in combination with polatuzumab vedotin: If the time between last dose of initial treatment and first dose of re-treatment including step dose is < 6 weeks, skip assessments in Cycle 1 and proceed with Cycle 2 assessments. Mosunetuzumab will be administered for up to a total of 17 cycles; polatuzumab vedotin (if applicable) will be administered for up to 6 cycles.
Screening assessments for re-treatment should follow the same schedule as the initial screening assessments. Screening assessments for Arm M-crossover should also follow the same schedule as the initial screening assessments, except for a repeat screening tumor biopsy requirement.
For any treatment-free intervals ≥6 weeks during study treatment, repeat step-up dosing of mosunetuzumab is required for the first cycle given after the dose delay. The same local assessments for step-up dosing as in Cycle 1 should be repeated. If a dose delay of >7 days occurs during Cycle 1, please refer to Section 5.1.7.2 for specific instructions for patients receiving mosunetuzumab IV or SC.

[a] Screening and pretreatment tests and evaluations will be performed within 14 days preceding the first dose of study treatment (except pretreatment biopsy, radiographic tumor assessment, and bone marrow aspirate and biopsy (if applicable), which may be performed up to 28 days preceding the first dose of study drug, providing no anti-tumor therapy was administered in this period). In addition, a serum pregnancy test should be performed within 7 days preceding the first dose of study treatment. Results of standard-of-care tests or examinations performed prior to obtaining informed consent and within the screening window specified above may be used; these tests do not need to be repeated for screening.

[b] Patients enrolled in Group A dose escalation will be hospitalized for at least 72 hours after the completion of mosunetuzumab administration on C1D1 and for a minimum of 24 hours on C2D1. Patients enrolled in Group B dose escalation will be hospitalized for at least 72 hours after the completion of mosunetuzumab administration on C1D8 and for a minimum of 24 hours on C2D1. Patients enrolled in Group C dose escalation will be hospitalized for at least 72 hours after the completion of mosunetuzumab administration on C2D1. Patients enrolled in the Arm K safety run-in will be hospitalized for at least 24 hours after the completion of mosunetuzumab administration on the first dosing day (i.e., C1D1) in which mosunetuzumab is given in combination with polatuzumab vedotin.

[c] For Group B, as well as Arms I, J, K*, and L* when using Group B dosing schedule: In Cycle 3 and beyond, study drug infusions/injections

should occur on Day 1 of each 21-day cycle but may be given up to ± 2 days from scheduled date (with a minimum of 19 days between doses) for logistic/scheduling reasons. Other study visits starting in Cycle 3 should occur within ± 2 days from the scheduled date, unless otherwise noted. For Groups A, C, *and Arms L and M,* as well as Arms I, J, and K when using Group A or C dosing schedule: For Cycle 2, study drug infusion/injection should occur on Day 1 of the cycle but may be given up to ± 1 day from the scheduled date (with a minimum of 6 days after C1D15 dosing). For Cycle 3 and beyond, study drug infusions/injections should occur on Day 1 of each 21-day cycle but may be given up to ± 2 days from scheduled date (with a minimum of 19 days between doses) for logistic/scheduling reasons. Other study visits starting in Cycle 2 should occur within ± 2 days from the scheduled date, unless otherwise noted.

[d] Informed consent must be documented before any study-specific screening procedure is performed.

[e] The CIRS-G assessment should be collected at screening for all patients ≥ 65 years old and for Arms J, L and M only (see Section 4.5.11).

[f] Transplant eligibility assessment, as determined by investigator (see Section 4.5.7).

[g] Unexplained weight loss > 10% over the previous 6 months, fever (> 38°C/100.4°F), and/or drenching night sweats.

[h] Medication (e.g., prescription drugs, over-the-counter drugs, vaccines, herbal or homeopathic remedies, nutritional supplements) used by a patient in addition to protocol-mandated treatment from 7 days prior to initiation of study drug until *90 days after the last dose of study treatment or start of new anti-lymphoma therapy, whichever is earlier*.

[i] After informed consent has been obtained but prior to initiation of study drug, only serious adverse events caused by a protocol-mandated intervention should be reported. After initiation of study drug, all adverse events will be reported until 90 days after the last dose of study treatment or the initiation of *next anti-lymphoma treatment*, whichever is earlier. After this period, the Sponsor should be notified if the investigator becomes aware of any serious adverse event (if believed to be related to prior study drug treatment) that occurs after the end of the adverse event reporting period (see Section 5.6). The investigator should follow each adverse event until the event has resolved to baseline grade or better, the event is assessed as stable by the investigator, the patient is lost to follow-up, or the patient withdraws consent. Every effort should be made to follow all serious adverse events considered to be related to study drug or trial-related procedures until a final outcome can be reported.

j For all groups/arms: Includes systolic and diastolic blood pressure, respiratory rate, pulse oximetry, pulse rate, and body temperature while the patient is in a sitting or semi-supine position. Record abnormalities observed at baseline on the General Medical History and Baseline Conditions eCRF. At subsequent visits, record new or worsened clinically significant abnormalities on the Adverse Event eCRF.

<u>For mosunetuzumab infusions</u>:

- Hospitalized patients for monitoring: Check vital signs pre-infusion, every 30 (±10) minutes during the infusion, at the end of the infusion, and then every 60 (±10) minutes until 6 hours after the end of infusion. Thereafter, vital signs should be checked every 4 hours until discharge.
- All other Cycle 1 and 2 mosunetuzumab infusions (or the subsequent cycle if CRS occurred after the last mosunetuzumab infusion): Check vital signs pre-infusion, every 30 (±10) minutes during the infusion, at the end of the infusion, and 2 hours after infusion.
- For subsequent mosunetuzumab IV infusions in the absence of CRS after last infusion: Check vital signs pre-infusion and every 60 (±15) minutes during the infusion and for 2 hours after the end of infusion. For patients who experienced CRS after the last mosunetuzumab IV, vital signs should be assessed pre-infusion, every 30 (±10) minutes during the infusion, and for 2 hours after the end of infusion

<u>For mosunetuzumab SC injections:</u>

- Hospitalized patients for monitoring: Check vital signs pre-injection (within 30 minutes) and every 15 (±10) minutes for one hour following the mosunetuzumab injection. After this 1-hour period is completed, vital signs should be recorded every 30 (± 10) minutes for 4 hours post-injection. Thereafter, vital signs should be monitored every 4 hours until hospital or clinic discharge.
- All other Cycle 1 and 2 mosunetuzumab SC injections (or the subsequent cycle if CRS occurred after last mosunetuzumab injection): Check vital signs pre-injection (within 30 minutes) and 30 (±15) minutes after injection.

For subsequent mosunetuzumab injections in the absence of CRS after the last injection: check vital signs pre-injection (within 30 minutes). *Observe patients for at least 15 minutes. Check vital signs once during observation period.*

<u>For polatuzumab vedotin infusions:</u>

- Vital signs should be assessed before the start of the infusion, every 15 (±5) minutes during the infusion, at the end of the infusion, and every 30 (±10) minutes for 90 minutes (Cycle 1 or if an infusion-related reaction occurred with the last polatuzumab vedotin) or for 30 (±10) minutes (subsequent cycles in the absence of an infusion-related reaction with the last polatuzumab vedotin infusion) after the end of infusion.

For <u>rituximab</u> infusions:

- *During the administration of rituximab in Cycle 1, vital signs are to be obtained before infusion of rituximab then after the start of the infusion, approximately every 15 (±5) minutes for 90 minutes, and then every 30 (±10) minutes until 1 hour after the end of the infusion. During administration of rituximab in subsequent cycles, vital signs are to be recorded before infusion of rituximab, then after the start of infusion, and approximately every 30 (±10) minutes until 1 hour after the end of infusion.*

[k] Height and BSA are required at screening only, within 96 hours of C1D1, unless there has been a > 10% change in body weight since the last BSA assessment, in which case BSA should be recalculated and documented in the eCRF.

[l] Complete physical examination includes an evaluation of the head, eyes, ears, nose, and throat, and the cardiovascular, dermatologic, musculoskeletal, respiratory, gastrointestinal, genitourinary, and neurologic systems. A complete neurologic examination, which includes an evaluation of mental status, cranial nerves, muscle strength, sensation, and coordination should be performed and documented in the patient chart. Record abnormalities observed at baseline on the General Medical History and Baseline Conditions eCRF. At subsequent visits, record new or worsened clinically significant abnormalities on the Adverse Event eCRF.

[m] Targeted physical examinations should be limited to systems of primary relevance (i.e., cardiovascular, respiratory, neurologic, and any system that might be associated with tumor assessment, or potential drug-related toxicity [e.g., clinical assessment for peripheral neuropathy in patients receiving polatuzumab vedotin]). Record new or worsened clinically significant abnormalities on the Adverse Event eCRF. For pre-infusion timepoints, targeted physical examinations may be performed within 96 hours preceding study treatment administration unless otherwise specified.

[n] See Section 4.5.9 for details. Single ECG recordings will be obtained at screening and at end of treatment. ECGs should also be performed when clinically indicated in any patient with evidence of, or suspicion for, clinically significant signs or symptoms of cardiac dysfunction. Obtain post-screening ECGs as close as possible to scheduled serum and plasma PK samples (see Appendix 3 and Appendix 4). If a PK sample is not scheduled for that timepoint, an unscheduled PK sample should be obtained.

[o] See Section 4.5.6 for details. Assess response using image-based evaluation, using standard Lugano 2014 criteria (see Appendix 7). PET and diagnostic-quality CT scans are required at screening, at the interim response assessment, and at the PRA visit. Perform CT scan with or without PET during follow-up at 9 months (±1 month) after C1D1, 12 months (±1 month) after C1D1, and then every 6 months (±1 month) until disease progression or study discontinuation, whichever is earlier. Before a metabolic complete response is achieved, it is recommended that PET scans should continue in conjunction with diagnostic-quality CT scans. A full tumor assessment including radiographic assessment must be performed any time disease progression or relapse is suspected. If disease progression or relapse is suspected before the PRA, both PET and diagnostic-quality CT scans should be performed for tumor assessment. Scans should be performed according to the guidelines in the imaging manual provided to all sites.

[p] Bone marrow examinations are required at screening for staging purposes for patients with FL and MCL. However, if the bone marrow involvement of lymphoma is confirmed by the presence of circulating lymphoma cells, the screening bone marrow biopsy may be omitted. Bone marrow examinations should include a biopsy for morphology and an aspirate for local hematology (flow studies are optional). Repeat bone marrow examinations are required to confirm a CR for CT-based response if there was bone marrow infiltration at screening, or if bone marrow involvement is suspected for disease relapse or transformation. Additional (unscheduled) bone marrow examinations may be performed at the discretion of the investigator. The associated hematopathology report should be submitted when available. For patients with DLBCL, PET/CT scans may be utilized to assess bone marrow involvement; bone marrow examinations are not required unless clinically indicated.

[q] Pretreatment, on-treatment, and re-treatment tumor tissue biopsies are mandatory. See Section 4.5.8 for details.
Pretreatment biopsy: Fresh pretreatment biopsy is preferred but archival tissue obtained after the last treatment is acceptable (see Section 4.5.8).
On-treatment biopsy: Obtain on-treatment biopsy between C1D16 and C2D8. *The on-treatment biopsy may be omitted if there is no lesion available to biopsy due to response to treatment or the on-treatment biopsy may cause additional safety risk(s) for the patient.* For expansion cohorts, an MRD blood sample is required when an on-treatment tumor biopsy is collected (see Appendix 3 and Appendix 4 for MRD blood collection).
Re-treatment biopsy: Patients proceeding to re-treatment following disease progression will need to complete screening assessments (Appendix 1) to re-confirm eligibility, including undergoing a repeat tumor biopsy from a safely accessible site (see Section 4.5.8). Patients who have no lesion amenable for biopsy at disease progression may still be considered for study drug re-treatment following a discussion between the study investigator and the Medical Monitor.
Optional tumor biopsies: Additional tumor biopsies are optional and may be performed at the investigator's discretion (e.g., to confirm disease recurrence or progression or to confirm an alternate histologic diagnosis); see Section 4.5.12 . For expansion cohorts, an MRD blood sample is required when optional tumor biopsies are collected (Appendix 3 and Appendix 4).
For all biopsies: All biopsies, whether fresh or archival, must be accompanied by the associated pathology report. Tumor tissue samples should consist of representative tumor specimens in paraffin blocks (preferred) or at least 20 unstained slides.

[r] Not applicable for a site that has not been granted approval for RBR sampling. Performed only for patients at participating sites who have provided written informed consent to participate. Obtain prior to study treatment.

[s] PROs will be evaluated in patients enrolled in Arm K using the EQ-5D-5L questionnaire. The questionnaire will be self-administered before the patient receives any information on disease status, prior to the performance of non-PRO assessments (except laboratory blood collections), and prior to the administration of study treatment, unless otherwise specified.

[t] HBsAg, HBsAb, HBcAb, HCV antibody, and HIV antibody serology are required. Patients whose hepatitis B serology results cannot rule out acute or chronic HBV infection must be negative for HBV by PCR to be eligible for study participation. Patients who are positive for HCV antibody must be negative for HCV by PCR to be eligible for study participation. *Patient who are positive for HIV may be eligible provided they are stable on anti-retroviral therapy, have a CD4 count ≥200/μL, and have an undetectable viral load.*

[u] Quantitative PCR for detection of active EBV and CMV should be performed at screening, C2D1, and when clinically indicated on a peripheral blood sample per local lab requirements. Blood samples should also be collected for central laboratory assessments at the same timepoints (see footnote "*y*" for sample types required for *central* lab assessments). If local laboratory assessments are not available for quantitative PCR detection of active EBV and CMV, local laboratory collections may be waived only if samples are collected for central laboratory assessments of viral infections. If EBV or CMV DNA levels are detected (positive), contact the Medical Monitor for additional recommendations, and repeat quantitative PCR monitoring weekly until DNA levels decrease, and then continue to monitor by quantitative PCR at every cycle until two consecutive negative (undetectable) results.

[v] Hematology includes CBC (including hemoglobin, hematocrit, RBC, WBC), platelet count, ANC, absolute lymphocyte count, and other cells.

[w] Chemistry panel (serum) includes sodium, potassium, chloride, bicarbonate, glucose, BUN or urea, creatinine, calcium, magnesium, phosphorous, total and direct bilirubin, total protein, albumin, ALT, AST, ALP, GGT, LDH, and uric acid.

[x] All women of childbearing potential will have a serum pregnancy test at screening. Serum pregnancy should be performed within 7 days preceding the first dose of study treatment. Urine or serum pregnancy tests will be performed at specified subsequent visits. If a urine pregnancy test is positive, it must be confirmed by a serum pregnancy test.

[y] For quantitative PCR detection of viral infection, which may include, but is not limited to, EBV and CMV. At screening, C2D1 pre-dose and at other timepoints when clinically indicated, blood samples should be sent for central laboratory assessments, in addition to local laboratory assessments (see footnote "u").

[z] Primary response assessment should be conducted at the end of Cycle 8 (C8D21 ± 1 week) prior to C9D1 for Groups A and C (and Arms I, J, K, L, and M if following the Group A or C dosing schedule); at the end of Cycle 9 (C9D21 ± 1 week) prior to C10D1 for Group B (and Arms I, J, K, L, and M if following Group B dosing schedule).

[aa] Group B includes one scheduled dose of mosunetuzumab on C9D1. Patients in Groups A and C and Arms I and J who are eligible for extended treatment with mosunetuzumab (see Section 3.1.5) may receive a total of 17 cycles of treatment with mosunetuzumab. All patients in Arm K will receive a total of 17 cycles of treatment with mosunetuzumab. Extended treatment beyond 8 cycles is not applicable to Arms L and M.

[bb] Patients who complete the treatment period will return to the clinic for a treatment completion visit within 30 (±7) days after the last dose of study drug. Patients who discontinue study drug prematurely will return to the clinic for a treatment discontinuation visit within 30 (±7) days after the last dose of study drug. The visit at which response assessment shows progressive disease may be used as the treatment discontinuation visit.

[cc] For C4D1 and C6D1.

[dd] When completed/discontinued from treatment, patients should be followed for survival follow-up and new anti-*lymphoma* therapy via telephone calls, patient medical records, and/or clinic visits approximately every 3 months until death (unless the patient withdraws consent or the Sponsor terminates the study). If the patient withdraws from the study, the site's staff may use a public information source (e.g., county records) to obtain information about survival status only.

[ee] Flow cytometry is the preferred method over peripheral blood smear. *Peripheral blood smear and/or flow cytometry can be used in place of a bone marrow biopsy to confirm the presence of circulating lymphoma cells, as indicated in footnote "p".* If malignant cells are detected, the results must be discussed with the Medical Monitor.

[ff] D8 and D15 assessments are not required for Arm M.

Appendix 2
Schedule for Post-Treatment Follow-Up

Assessments/Procedures	Post-Treatment Follow-Up [a]
EQ-5D-5L (Arm K) [b]	Every 3 months × 2 then every 6 months
Targeted physical examination [c]	Every 3 months
Vital signs (blood pressure, pulse rate, and body temperature)	Every 3 months
ECOG PS	Every 3 months
Tumor assessments [d]	At 9 months, 12 months, then every 6 months
Total IgA, IgG, IgM	Every 6 months
Hematology [e]	Every 3 months × 2 then every 6 months
Serum chemistry [f]	Every 3 months × 2 then every 6 months
Bone marrow biopsy and aspirate [g]	As needed to confirm relapse
Tumor biopsy for biomarkers (optional) [h]	At disease progression
Blood biomarker sample	See Appendix 3 and Appendix 4
Serum/plasma PK sample and serum ADA sample for mosunetuzumab and polatuzumab vedotin	Once, ≥ 90 days after last study drug administration (see Appendix 3 and Appendix 4)

ADA = anti-drug antibody; C = cycle; CT = computed tomography (scan); ECOG PS = Eastern Cooperative Oncology Group Performance Status; eCRF = electronic Case Report Form; EQ-5D-5L = EuroQol 5-Dimension, 5-Level (questionnaire); GGT = gamma-glutamyl transferase; LDH = lactate dehydrogenase; NK = natural killer (cell); PET = positron emission tomography (scan); PK = pharmacokinetic.

[a] Schedule corresponds to visit timepoints only for patients who complete or discontinue the study treatment, but remain on the study without disease progression. Continue to follow patients on this schedule timed from the study drug completion/early discontinuation visit. The first two visits should occur within ± 7 days from the scheduled date, while subsequent visits should occur within ± 14 days from the scheduled date. Other assessments/procedures can be performed at an earlier timepoint to align with the tumor assessment visit. Perform assessments until disease relapse/progression (assessments should be performed for the last visit when disease relapse/progression occurs), start of new anti-cancer therapy, or withdrawal from study participation, whichever occurs first.

[b] Patients should complete the questionnaire prior to any other study assessments (except laboratory blood collections).

[c] Targeted physical examinations should be limited to systems of primary relevance (i.e., cardiovascular, respiratory, neurologic, and any system that might be associated with tumor assessment [e.g., lymph nodes, liver, and spleen and those systems associated with symptoms], or potential drug related toxicity [e.g., clinical assessment for peripheral neuropathy in patients receiving polatuzumab vedotin]). Changes from baseline abnormalities should be recorded in patient notes. New or worsened clinically significant abnormalities should be recorded as adverse events on the Adverse Event eCRF.

[d] Assess response using image-based evaluation using standard Lugano 2014 criteria (see Appendix 7). Perform CT scan with or without PET during follow up at 9 months (±1 month) after C1D1, 12 months (±1 month) after C1D1, and then every 6 months (±1 month) until disease progression or study discontinuation, whichever is earlier. Before a metabolic complete response is achieved, it is recommended that PET scans should continue in conjunction with diagnostic-quality CT scans. A full tumour assessment including radiographic assessment using CT scan with or without PET must be performed any time disease progression or relapse is suspected during follow up. Scans should be performed according to the guidelines in the imaging manual provided to all sites.

[e] Hematology includes CBC (including hemoglobin, hematocrit, RBC, WBC), platelet count, ANC, absolute lymphocyte count, and other cells.

[f] Chemistry panel (serum) includes sodium, potassium, chloride, bicarbonate, glucose, BUN or urea, creatinine, calcium, magnesium, phosphorous, total and direct bilirubin, total protein, albumin, ALT, AST, ALP, GGT, LDH, and uric acid.

[g] Bone marrow examination (biopsy and aspirate for morphology) is required only if there is a clinical suspicion of disease recurrence in the bone marrow. Unsuccessful attempts at marrow aspiration will not be considered a protocol violation. For patients with DLBCL, PET/CT scans may be utilized to assess bone marrow involvement; bone marrow examinations are not required unless clinically indicated.

[h] Optional tumor biopsies may be performed at disease progression and at the investigator's discretion (e.g., to confirm disease recurrence or progression or to confirm an alternate histologic diagnosis). Tumor tissue samples should consist of representative tumor specimens in paraffin blocks (preferred) or at least 20 unstained slides. For expansion cohorts, an MRD blood sample is required when optional tumor biopsies are collected (Appendix 3 and Appendix 4)

Appendix 3
Schedule of Pharmacokinetic, Immunogenicity, and Biomarker Blood Samples: Groups A, B, and C (Dose-Finding Phase) and Arms I and J (Combination Expansion Phase)

Visit	Timepoint	PK/ADA Sample Type	Biomarker Sample Type [f]
Cycle 1,<br>Day 1	≤8 hours prior to the first study drug infusion	Group A and Arms I and J, if Group A regimen is used:<br>Mosunetuzumab PK (serum)<br>Mosunetuzumab ADA (serum)<br>Pola PK (plasma) for acMMAE and unconjugated MMAE [a]<br>Pola PK (serum) for total antibody<br>Pola ADA (serum)<br>Rituximab PK (serum) [b]<br>Obinutuzumab PK (serum) [b]	Groups A, B, and C and Arms I and J:<br>Blood for flow cytometry<br>Blood for TBNK<br>Plasma for cytokines<br>MRD sample<br>Blood for PBMC
		Group B and Arms I and J, if Group B regimen is used:<br>Mosunetuzumab PK (serum)<br>Mosunetuzumab ADA (serum)<br>Pola PK (plasma) for acMMAE and unconjugated MMAE [a]<br>Pola PK (serum) for total antibody<br>Pola ADA (serum)<br>Rituximab PK (serum) [b]<br>Obinutuzumab PK (serum) [b]	–
		Group C and Arms I and J, if Group C regimen is used:<br>Mosunetuzumab PK (serum)<br>Mosunetuzumab ADA (serum)<br>Rituximab PK (serum) [b]<br>Obinutuzumab PK (serum) [b]	–
	0–30 minutes after end of infusion of the respective agent	Group A and Arms I and J, if Group A regimen is used:<br>Mosunetuzumab PK (serum)<br>Pola PK (plasma) for acMMAE and unconjugated MMAE [a]<br>Group B and Arms I and J, if Group B regimen is used:<br>Pola PK (plasma) for acMMAE and unconjugated MMAE [a]<br>Group C and Arms I and J, if Group C regimen is used:<br>Mosunetuzumab PK (serum)	Group A and Arms I and J, if Group A regimen is used (collect at 0–30 minutes after end of Pola infusion):<br>Blood for flow cytometry<br>Blood for TBNK<br>Plasma for cytokines<br>Blood for PBMC isolation[i]

Visit	Timepoint	PK/ADA Sample Type	Biomarker Sample Type [f]
Cycle 1, Day 1 (cont.)	2 hours (±30 minutes) after end of infusion of mosunetuzumab	Groups A and C and Arms I and J, if Group A or C regimen is used:<br>Mosunetuzumab PK (serum)	Groups A, B, and C and Arms I and J:<br>Blood for flow cytometry<br>Blood for TBNK<br>Plasma for cytokines<br>Blood for PBMC isolation
Cycle 1, Day 2	24 (±4) hours after end of infusion of the respective agent on Cycle 1 Day 1	Group A:<br>Mosunetuzumab PK (serum)<br>Pola PK (plasma) for acMMAE and unconjugated MMAE [a]<br>Group B:<br>Pola PK (plasma) for acMMAE and unconjugated MMAE [a]<br>Group C:<br>Mosunetuzumab PK (serum)	–
Cycle 1, Day 4	72 (±4) hours after end of mosunetuzumab infusion on Cycle 1 Day 1	Group A only:<br>Mosunetuzumab PK (serum)	–
Cycle 1, Days 8 and 15	≤8 hours prior to the infusion of mosunetuzumab	Group A and Arms I and J, if Group A regimen is used:<br>Mosunetuzumab PK (serum)<br>Pola PK (plasma) for acMMAE and unconjugated MMAE [a]<br>Group B and Arms I and J, if Group B regimen is used:<br>Mosunetuzumab PK (serum)<br>Pola PK (plasma) for acMMAE and unconjugated MMAE [a]<br>Group C and Arms I and J, if Group C regimen is used:<br>Mosunetuzumab PK (serum)	Groups A, B, and C and Arms I and J:<br>Plasma for cytokines<br>MRD sample [f]<br>Blood for PBMC isolation<br>Blood for flow cytometry
	0–30 minutes after end of infusion of mosunetuzumab	Groups A, B, and C and Arms I and J, (regimens A, B, or C):<br>Mosunetuzumab PK (serum)	–

Visit	Timepoint	PK/ADA Sample Type	Biomarker Sample Type [f]
Cycle 1, Days 8 and 15	2 hours (±30 minutes) after end of infusion of mosunetuzumab	Groups A, B, and C and Arms I and J (regimens A, B, or C):<br>• Mosunetuzumab PK (serum)	Groups A, B, and C and Arms I and J:<br>Plasma for cytokines<br>Blood for flow cytometry
Cycle 1, Day 9	24 (±4) hours after end of mosunetuzumab infusion on Cycle 1, Day 8	Group B only:<br>Mosunetuzumab PK (serum)	–
Cycle 1, Day 11	72 (±4) hours after end of mosunetuzumab infusion on Cycle 1, Day 8	Group B only:<br>Mosunetuzumab PK (serum)	–
Cycle 2, Day 1	≤8 hours prior to the first study drug infusion	Groups A, B, and C and Arms I and J (regimens A, B, or C):<br>Mosunetuzumab PK (serum)<br>Mosunetuzumab ADA (serum)<br>Pola PK (plasma) for acMMAE and unconjugated MMAE [a]<br>Pola PK (serum) for total antibody<br>Pola ADA (serum)<br>Rituximab PK (serum) [b]<br>Obinutuzumab PK (serum) [b]	Groups A, B, and C and Arms I and J:<br>Blood for flow cytometry<br>Blood for TBNK<br>Plasma for cytokines<br>Blood for PBMC isolation<br>MRD sample [f]
	0–30 minutes after end of infusion of the respective agent	Groups A, B, and C and Arms I and J (regimens A, B, or C):<br>Mosunetuzumab PK (serum)<br>Pola PK (plasma) for acMMAE and unconjugated MMAE [a]	–
	2 hours (± 30 minutes) after end of mosunetuzumab infusion	Groups A, B, and C and Arms I and J (regimens A, B, or C):<br>Mosunetuzumab PK (serum)<br>Pola PK (plasma) for acMMAE and unconjugated MMAE [a]	Groups A, B, and C and Arms I and J:<br>Blood for flow cytometry<br>Blood for TBNK<br>Plasma for cytokines

Appendix 3 Schedule of Pharmacokinetic, Immunogenicity, and Biomarker Blood Samples: Groups A, B, and C (Dose-Finding Phase) and Arms I and J (Combination Expansion Phase) (cont.)

Visit	Timepoint	PK/ADA Sample Type	Biomarker Sample Type [f]
Cycle 3, Day 1	≤8 hours prior to the first study drug infusion	Group A, B, and C and Arms I and J (regimens A, B, or C):<br>Mosunetuzumab PK (serum)<br>Mosunetuzumab ADA (serum)<br>Pola PK (plasma) for acMMAE and unconjugated MMAE [a]<br>Pola PK (serum) for total antibody<br>Pola ADA (serum)	Groups A, B, and C and Arms I and J:<br>Blood for TBNK<br>MRD sample[f]
	0–30 minutes after end of infusion of the respective agent	Groups A, B, and C and Arms I and J, (regimens A, B, or C):<br>Mosunetuzumab PK (serum)<br>Pola PK (plasma) for acMMAE and unconjugated MMAE [a]	–
	2 hours (±30 minutes) after end of mosunetuzumab infusion	–	Groups A, B, and C and Arms I and J:<br>Blood for TBNK
Cycle 4, Day 1	≤8 hours prior to the first study drug infusion	Group A, B, and C and Arms I and J (regimens A, B, or C):<br>Mosunetuzumab PK (serum)<br>Pola PK (plasma) for acMMAE and unconjugated MMAE [a]	Groups A, B, and C and Arms I and J:<br>MRD sample [f]
	0–30 minutes after end of infusion of the respective agent	Groups A, B, and C and Arms I and J (regimens A, B, or C):<br>Mosunetuzumab PK (serum)<br>Pola PK (plasma) for acMMAE and unconjugated MMAE [a]	–
Cycle 5, Day 1	≤8 hours prior to the first study drug infusion	Groups A, B, and C and Arms I and J (regimens A, B, or C):<br>Mosunetuzumab PK (serum)<br>Pola PK (plasma) for acMMAE and unconjugated MMAE [a]	Groups A, B, and C and Arms I and J:<br>Blood for TBNK<br>Arms I and J:<br>Blood for PBMC isolation<br>Blood for flow cytometry
	0–30 minutes after end of infusion of the respective agent	Groups A, B, and C and Arms I and J (regimens A, B, or C):<br>Mosunetuzumab PK (serum)<br>Pola PK (plasma) for acMMAE and unconjugated MMAE [a]	–

Appendix 3 Schedule of Pharmacokinetic, Immunogenicity, and Biomarker Blood Samples: Groups A, B, and C (Dose-Finding Phase) and Arms I and J (Combination Expansion Phase) (cont.)

Visit	Timepoint	PK/ADA Sample Type	Biomarker Sample Type [f]
Cycle 5, Day 1	2 hours (± 30 minutes) after end of mosunetuzumab infusion	–	–
Cycle 8, Day 1	≤ 8 hours prior to mosunetuzumab infusion	Groups A, B, and C and Arms I and J (regimens A, B, or C):<br>Mosunetuzumab PK (serum)<br>Mosunetuzumab ADA (serum)<br>Pola PK (serum) for total antibody<br>Pola ADA (serum)	Arms I and J:<br>MRD sample [f]
	0–30 minutes after end of mosunetuzumab infusion	Groups A, B, and C and Arms I and J (regimens A, B, or C):<br>Mosunetuzumab PK (serum)	–
Cycles 9 and Beyond, Day 1 [e]	≤ 8 hours prior to the mosunetuzumab infusion	Groups A, B, and C and Arms I and J (regimens A, B, or C):<br>For Cycles 9 and 16 only:<br>Mosunetuzumab PK (serum)<br>For Cycle 16 only:<br>Mosunetuzumab ADA (serum)	Arms I and J:<br>Blood for TBNK (Cycles 9 and 11 only)<br>Blood for PBMC isolation (Cycle 9 only)
	2 hours (± 30 minutes) after end of mosunetuzumab infusion	–	–
Study Drug Completion/ Early Discontinuation [d]	NA	Groups A, B, and C and Arms I and J (regimens A, B, or C):<br>Mosunetuzumab PK (serum)<br>Mosunetuzumab ADA (serum)<br>Pola PK (plasma) for acMMAE and unconjugated MMAE [a]<br>Pola PK (serum) for total antibody<br>Mosunetuzumab ADA (serum)<br>Pola ADA (serum)	Arms I and J:<br>MRD sample [f]<br>Blood for PBMC isolation

Appendix 3 Schedule of Pharmacokinetic, Immunogenicity, and Biomarker Blood Samples: Groups A, B, and C (Dose-Finding Phase) and Arms I and J (Combination Expansion Phase) (cont.)

Visit	Timepoint	PK/ADA Sample Type	Biomarker Sample Type [f]
Follow-Up	NA	Groups A, B, and C and Arms I and J (regimens A, B, or C):<br>Collect the following samples at ≥90 days after the last study treatment:<br>Mosunetuzumab PK (serum)<br>Mosunetuzumab ADA (serum)<br>Pola PK (serum) for total antibody<br>Pola ADA (serum)	Arms I and J:<br>MRD sample [c, f]<br>Blood for PBMC isolation [c]<br>Blood for TBNK [c]

acMMAE = antibody-conjugated monomethyl auristatin E; ADA = anti-drug antibody; MMAE = monomethyl auristatin E; MRD = minimal residual disease; NA = not applicable; PBMC = peripheral blood mononuclear cells; PK = pharmacokinetic; Pola = polatuzumab vedotin; TBNK = T, B, and natural killer cells.

Note: Timepoints are relative to the end of mosunetuzumab infusion for each cycle, unless otherwise noted.

[a] The plasma sample will be split into two samples for analyses of acMMAE and unconjugated MMAE concentrations.

[b] Predose serum rituximab and/or obinutuzumab PK in Cycle 1 and Cycle 2 is required for patients who have received prior treatment with rituximab and/or obinutuzumab.

[c] Collect every 3 months for 2 years or until disease progression or treatment with a new anti-cancer therapy, whichever occurs sooner.

[d] Samples should be collected when **all** study drugs have been completed or discontinued.

[e] For patients who receive Cycles 9–17 only.

[f] For expansion cohorts, an MRD blood sample is also required when an on-treatment tumor biopsy (taken between C1D16 and C2D8) or an optional tumor biopsy is collected (see Appendix 1 and Appendix 2).

[g] Central laboratory assessments at 24 hours (±4 hours) post Day 1 of Cycle 1 should only be collected if the patient is hospitalized.

Appendix 4
Schedule of Pharmacokinetic, Immunogenicity, and Biomarker Blood Samples: Arms K and L (Mosun*etuzumab* SC Combination Expansion Phase)

Visit	Timepoint	PK/ADA Sample Type [h]	Biomarker Sample Type [e, h]
Cycle 1, Day 1	≤8hours prior to first study drug administration	Mosunetuzumab PK (serum)<br>Mosunetuzumab ADA (serum)<br>Rituximab PK (serum) [a]<br>Obinutuzumab PK (serum) [a]<br>Polatuzumab vedotin PK for acMMAE and unconjugated MMAE (plasma) [g]<br>Polatuzumab vedotin PK for total antibody (serum)<br>Polatuzumab vedotin ADA (serum)	Blood for flow cytometry<br>Blood for TBNK<br>Plasma for cytokines<br>Blood for PBMC isolation<br>MRD sample [e]
	0–30 minutes after end of infusion (EOI) of the polatuzumab vedotin	Polatuzumab vedotin PK for acMMAE and unconjugated MMAE (plasma) [g]	Blood for flow cytometry<br>Blood for TBNK<br>Plasma for cytokines
	2 h (±30 min) EOI of polatuzumab vedotin	Polatuzumab vedotin PK for acMMAE and unconjugated MMAE (plasma) [g]	
Cycle 1, Day 2	24 hours (±4 hours) after end of mosunetuzumab injection [f]	Mosunetuzumab PK (serum)	Plasma for cytokines<br>Blood for flow cytometry<br>Blood for TBNK
Cycle 1, Days 8 and 15	≤8 hours prior to mosunetuzumab administration	Mosunetuzumab PK (serum)	Blood for TBNK<br>Plasma for cytokines<br>Blood for PBMC isolation<br>MRD sample [e]<br>Blood for flow cytometry

Visit	Timepoint	PK/ADA Sample Type [h]	Biomarker Sample Type [e, h]
Cycle 2, 3, 4, 5, Day 1	≤8 hours prior to first study drug administration	Mosunetuzumab PK (serum)<br>Mosunetuzumab ADA (serum) (Cycle 2 and 4 only)<br>Polatuzumab vedotin PK for acMMAE and unconjugated MMAE (plasma) [g]<br>Polatuzumab vedotin PK for total antibody (serum) (Cycles 2 and 4 only)<br>Polatuzumab vedotin ADA (serum) (Cycles 2 and 4 only)<br>Rituximab PK (serum) (Cycle 2 only) [a]<br>Obinutuzumab PK (serum) (Cycle 2 only) [a]	Blood for flow cytometry (Cycles 2, 3 and 5 only)<br>Blood for TBNK (Cycles 2, 3, and 5 only)<br>Plasma for cytokines (Cycles 2 and 5 only)<br>Blood for PBMC isolation (Cycles 2 and 5 only)<br>MRD sample (Cycles 2, 3, and 4 only) [e]
	0–30 minutes after EOI of the polatuzumab vedotin	Polatuzumab vedotin PK for acMMAE and unconjugated MMAE (plasma) [g]	–
Cycle 8, Day 1	≤8 hours prior to injection of mosunetuzumab	Mosunetuzumab PK (serum)<br>Mosunetuzumab ADA (serum)<br>Polatuzumab vedotin PK for total antibody (serum)<br>Polatuzumab vedotin ADA (serum)	MRD sample [e]
Cycles 9 and beyond, Day 1 *(Arm K only)* [d]	≤8 hours prior to injection of mosunetuzumab	Mosunetuzumab PK (serum; Cycles 9 and 16 only)<br>Mosunetuzumab ADA (serum; Cycles 16 only)	Blood for TBNK (Cycles 9 and 11 only, Arm K only)<br>Blood for PBMC isolation (Cycle 9 only, Arm K only)

Visit	Timepoint	PK/ADA Sample Type [h]	Biomarker Sample Type [e, h]
Study drug completion/ Early discontinuation [c]	NA	Mosunetuzumab PK (serum)<br>Mosunetuzumab ADA (serum)<br>Polatuzumab vedotin PK for acMMAE and unconjugated MMAE (plasma) [g]<br>Polatuzumab vedotin PK for total antibody (serum)<br>Polatuzumab vedotin ADA (serum)	MRD sample [e]<br>Blood for PBMC isolation
Follow-up	NA	Collect the following samples at ≥ 90 days after the final dose of study treatment:<br>Mosunetuzumab PK (serum)<br>Mosunetuzumab ADA (serum)<br>Polatuzumab vedotin PK for total antibody (serum)<br>Polatuzumab vedotin ADA (serum)	MRD sample [b, e]<br>Blood for PBMC isolation [b]<br>Blood for TBNK [b]

ADA = anti-drug antibody; MRD = minimal residual disease; NA = not applicable; PBMC = peripheral blood mononuclear cells; PK = pharmacokinetic; TBNK = T, B, and natural killer cells.

[a] Predose serum rituximab and/or obinutuzumab pharmacokinetics in Cycles 1 and 2 is required for patients who have received prior treatment with rituximab and/or obinutuzumab.

[b] Collect every 3 months for 2 years or until disease progression or treatment with a new anti-cancer therapy, whichever occurs sooner.

[c] Samples should be collected when all study drugs have been completed or discontinued.

[d] For patients who receive Cycles 9–17 only (Arm K).

[e] An MRD blood sample is also required when an on-treatment tumor biopsy (taken between Day 16 of Cycle 1 and Day 8 of Cycle 2) or an optional tumor biopsy is collected (refer to Appendix 1 and Appendix 2).

[f] Central laboratory assessments at 24 hours (±4 h) post Day 1 of Cycle 1 should only be collected if the patient is hospitalized or if clinically indicated at the discretion of the investigator in consultation with the Medical Monitor.

[g] The plasma sample will be split into two samples for analyses of acMMAE and unconjugated MMAE concentrations.

[h] Plasma for cytokine and serum for mosunetuzumab pharmacokinetics should also be collected if the patient is hospitalized for CRS.

Appendix 5
Schedule of Pharmacokinetic, Immunogenicity, and Biomarker Blood Samples: Arm M (Rituximab-Polatuzumab Vedotin Control; Expansion Phase)

Visit	Timepoint	PK/ADA Sample Type	Biomarker Sample Type [b]
Cycle 1, Day 1	≤8 hours prior to the first infusion	Polatuzumab vedotin PK (serum) for total antibody<br>Polatuzumab vedotin ADA (serum)	Blood for flow cytometry<br>Blood for TBNK<br>Plasma for cytokines<br>Blood for PBMC isolation<br>MRD sample
	0–30 minutes after end of infusion of rituximab	–	Blood for flow cytometry<br>Blood for TBNK<br>Plasma for cytokines<br>Blood for PBMC isolation
	2 hours (±30 minutes) after end of last drug administered	Polatuzumab vedotin PK for acMMAE and unconjugated MMAE (plasma)	Blood for flow cytometry
Cycle 2, Day 1	≤8 hours prior to the first infusion	Polatuzumab vedotin PK (serum) for total antibody<br>Polatuzumab vedotin ADA (serum)<br>Polatuzumab vedotin PK for acMMAE and unconjugated MMAE (plasma)	Blood for flow cytometry<br>Blood for TBNK<br>Plasma for cytokines<br>Blood for PBMC isolation<br>MRD sample[b]
	2 hours (±30 minutes) after end of last drug administered	–	Blood for flow cytometry<br>Blood for TBNK<br>Plasma for cytokines
Cycle 3, Day 1	≤8 hours prior to the first infusion	–	Blood for TBNK<br>MRD sample[b]
	2 hours (±30 minutes) after end of last drug administered	–	–
Cycle 4, Day 1	≤8 hours prior to the first infusion	–	MRD sample[b]

Appendix 5 Schedule of Pharmacokinetic, Immunogenicity, and Biomarker Blood Samples: Arm M (Rituximab-Polatuzumab Vedotin Control; Expansion Phase) (cont.)

Visit	Timepoint	PK/ADA Sample Type	Biomarker Sample Type [b]
Cycle 5, Day 1	≤8 hours prior to the first infusion	Polatuzumab vedotin PK (serum) for total antibody<br>Polatuzumab vedotin ADA (serum)Polatuzumab vedotin PK for acMMAE and unconjugated MMAE (plasma)	Blood for TBNK<br>Blood for flow cytometry
	2 hours (±30 minutes) after end of last drug administered	Polatuzumab vedotin PK for acMMAE and unconjugated MMAE (plasma)	–
Cycle 8, Day 1	≤8 hours prior to the first infusion<br>≤8 hours prior to the first infusion	–	Blood for PBMC isolation
Study Drug Completion / Early Discontinuation [d]	–	Polatuzumab vedotin PK (plasma) for acMMAE and unconjugated MMAE [c]<br>Polatuzumab vedotin PK (serum) for total antibody<br>Polatuzumab vedotin ADA (serum)	MRD sample [b]<br>Blood for PBMC isolation
Follow-Up	NA	Collect the following samples at ≥90 days after the last dose of study treatment:<br>Polatuzumab vedotin PK (serum) for total antibody<br>Polatuzumab vedotin ADA (serum)	MRD sample [b, e]<br>Blood for PBMC isolation [e]<br>Blood for TBNK [e]

acMMAE = antibody-conjugated monomethyl auristatin E; ADA = anti-drug antibody; MMAE = monomethyl auristatin E; MRD = minimal residual disease; NA = not applicable; PBMC = peripheral blood mononuclear cells; PK = pharmacokinetic; Pola = polatuzumab vedotin; TBNK = T, B, and natural killer cells.

[a] Predose serum obinutuzumab PK is required for patients who have received prior treatment with obinutuzumab.

[b] For expansion cohorts, an MRD blood sample is required when an on-treatment tumor biopsy (taken between C1D16 and C2D8) or an optional tumor biopsy is collected (see Appendix 3 and Appendix 4).

[c] The plasma sample will be split into two samples for analyses of antibody-conjugated MMAE and unconjugated MMAE concentrations.

[d] Samples should be collected when all study drugs have been completed or discontinued.

[e] Collect every 3 months for 2 years or until disease progression or treatment with a new anti-cancer therapy, whichever occurs sooner.

Appendix 6
Schedule for Tocilizumab Treatment of Cytokine Release Syndrome

Assessment/Procedure [a]	Pre-TCZ Tx (within 24 hours)	TCZ IV Admin.	Post-TCZ Treatment [b, c]					
			6 hours	1 day	2 days	3 days	8 days	8 weeks
TCZ administration (8 mg/kg for patients ≥ 30 kg; 12mg/kg for patients < 30 kg; doses exceeding 800 mg per infusion are not recommended)		x						
Vital signs [d]	x [e]		Measure at least every 6 hours until resolution to baseline, then every 12 hours until end of hospitalization [e]					
Pressor documentation [f]	x [e]		Record at least every 6 hours until pressors are discontinued [e]					
FiO_2	x [e]		Record at least every 6 hours until patient on room air [e]					
Pulse oximetry, resting	x [e]		Measure at least every 6 hours until resolution to baseline, and then every 12 hours until end of hospitalization [e]					
**Local Laboratory Assessments**								
Hematology	x		x	x	x	x	x	
Liver function tests (AST, ALT, total bilirubin)	x		x	x	x	x	x	
Serum chemistry and creatinine [g]	x		x	x	x	x	x	
CRP, LDH, and serum ferritin	x		x	x	x	x	x	
Coagulation (aPTT, PT/INR, fibrinogen)	x		x	x	x	x	x	
Infection workup [h]	x							
**Central Laboratory Assessments**								
Serum cytokines	x	x	x	x	x	x	x	

Assessment/Procedure [a]	Pre-TCZ Tx (within 24 hours)	TCZ IV Admin.	Post-TCZ Treatment [b, c]					
			6 hours	1 day	2 days	3 days	6 hours	8 weeks
Serum IL-6 PD markers [i]	x	x [j]	x	x	x	x	x	
Serum TCZ pharmacokinetics	x	x [j]	x	x	x	x	x	x
Serum TCZ ADA	x							x

ADA = anti-drug antibodies; Admin. = administration; CRP = C-reactive protein; EBV = Epstein-Barr virus; eCRF = electronic Case Report Form; IL-6 = interleukin 6; LDH = lactate dehydrogenase; PD = pharmacodynamic; TCZ = tocilizumab; Tx = treatment.

Note: Record abnormalities or worsened clinically significant abnormalities on the Adverse Event eCRF.

[a] An assessment/procedure may be waived if a patient is hospitalized at a facility that does not have the capacity to perform study the assessment. Hospitalization should not be prolonged to perform study assessments in this schedule of assessments.

[b] If the TCZ dose is repeated, follow schedule following the *last* TCZ dose.

[c] For post-TCZ treatment timepoints: 6 hours (± 30 minutes), 1 day (24 ± 4 hours), 2 days (48 ± 4 hours), 3 days (72 ± 4 hours), 8 days (192 ± 48 hours), and 8 weeks (56 days ± 48 hours) after completion of TCZ infusion.

[d] Includes respiratory rate, pulse rate, and systolic and diastolic blood pressure while the patient is in a seated or supine position, and temperature.

[e] The maximum and minimum values for any 24-hour period should be recorded in the clinical database.

[f] Document vasopressor type and dose in the concomitant medication eCRF.

[g] Includes sodium, potassium, chloride, bicarbonate, glucose, and BUN.

[h] Includes assessment for bacterial, fungal, and viral infections: cultures, serologies and molecular diagnostic tests. Assessment of pretreatment and on-treatment EBV status should be conducted, including enumeration of EBV viral load in PBMC and plasma, and evaluation of EBV encoded ribonucleotides (EBER) or EBV nuclear antigen (EBNA) with T/B/NK cell markers.

[i] Includes IL-6, soluble IL-6R, sgp130.

[j] Blood draws for serum TCZ PK and serumIL-6 PD markers will be performed within 15 minutes post-end of TCZ infusion and will be drawn from the arm that was not used to administer TCZ.

Appendix 7
Lugano Response Criteria for Malignant Lymphoma
(Cheson et al. 2014)

TARGET AND NON-TARGET LESIONS

Up to six of the largest target nodes, nodal masses, or other lymphomatous lesions that are measurable in two diameters should be identified from different body regions representative of the patient's overall disease burden and include mediastinal and retroperitoneal disease, if involved. At baseline, a measurable node must be greater than 15 mm in longest diameter (LDi). Measurable extranodal disease may be included in the six representative, measured lesions. At baseline, measurable extranodal lesions should be greater than 10 mm LDi.

All other lesions (including nodal, extranodal, and assessable disease) should be followed as non-measured disease as non-target lesions (e.g. cutaneous, GI, bone, spleen, liver, kidneys, pleural or pericardial effusions, ascites, bone, bone marrow).

SPLIT LESIONS AND CONFLUENT LESIONS

Lesions may split or may become confluent over time. In the case of split lesions, the individual product of the perpendicular diameters (PPDs) of the nodes should be summed together to represent the PPD of the split lesion; this PPD is added to the sum of the PPDs of the remaining lesions to measure response. If subsequent growth of any or all of these discrete nodes occurs, the nadir of each individual node is used to determine progression. In the case of confluent lesions, the PPD of the confluent mass should be compared with the sum of the PPDs of the individual nodes, with more than 50% increase in PPD of the confluent mass compared with the sum of individual nodes necessary to indicate progressive disease. The LDi and smallest diameter (SDi) are no longer needed to determine progression.

Response and Site	PET-CT–Based Response	CT-Based Response
Complete	Complete metabolic response	Complete radiologic response (all of the following)
Lymph nodes and extralymphatic sites	Score 1, 2, or 3 [a] with or without a residual mass on 5PS [b]<br>It is recognized that in Waldeyer's ring or extranodal sites with high physiologic uptake or with activation within spleen or marrow (e.g., with chemotherapy or myeloid colony-stimulating factors), uptake may be greater than normal mediastinum and/or liver. In this circumstance, complete metabolic response may be inferred if uptake at sites of initial involvement is no greater than surrounding normal tissue even if the tissue has high physiologic uptake.	Target nodes/nodal masses must regress to ≤ 1.5 cm in LDi<br>No extralymphatic sites of disease
Non-measured lesion	Not applicable	Absent
Organ enlargement	Not applicable	Regress to normal
New lesions	None	None
Bone marrow	No evidence of FDG-avid disease in marrow	Normal by morphology; if indeterminate, IHC negative
Partial	Partial metabolic response	Partial remission (all of the following)
Lymph nodes and extralymphatic sites	Score 4 or 5 [b] with reduced uptake compared with baseline and residual mass(es) of any size	≥ 50% decrease in SPD of up to 6 target measurable nodes and extranodal sites
	At interim, these findings suggest responding disease	When a lesion is too small to measure on CT, assign 5 mm × 5 mm as the default value
	At end of treatment, these findings indicate residual disease	When no longer visible, 0 × 0 mm<br>For a node > 5 mm × 5 mm, but smaller than normal, use actual measurement for calculation

Appendix 7 Lugano Response Criteria for Malignant Lymphoma (Cheson et al. 2014) (cont.)

Response and Site	PET-CT–Based Response	CT-Based Response
Non-measured lesion	Not applicable	Absent/normal, regressed, but no increase
Organ enlargement	Not applicable	Spleen must have regressed by > 50% in length beyond normal
New lesions	None	None
Bone marrow	Residual uptake higher than uptake in normal marrow but reduced compared with baseline (diffuse uptake compatible with reactive changes from chemotherapy allowed). If there are persistent focal changes in the marrow in the context of a nodal response, consideration should be given to further evaluation with MRI or biopsy or an interval scan.	Not applicable
No response or stable disease	No metabolic response	Stable disease
Target nodes/nodal masses, extranodal lesions	Score 4 or 5 [b] with no significant change in FDG uptake from baseline at interim or end of treatment	< 50% decrease from baseline in SPD of up to 6 dominant, measurable nodes and extranodal sites; no criteria for progressive disease are met
Non-measured lesion	Not applicable	No increase consistent with progression
Organ enlargement	Not applicable	No increase consistent with progression
New lesions	None	None
Bone marrow	No change from baseline	Not applicable
Progressive disease	Progressive metabolic disease	Progressive disease requires at least 1 of the following
Individual target nodes/nodal masses	Score 4 or 5 [b] with an increase in intensity of uptake from baseline and/or	PPD progression:

Response and Site	PET-CT–Based Response	CT-Based Response
Extranodal lesions	New FDG-avid foci consistent with lymphoma at interim or end-of-treatment assessment	An individual node/lesion must be abnormal with:<br>LDi > 1.5 cm and<br>Increase by > 50% from PPD nadir and<br>An increase in LDi or SDi from nadir<br>0.5 cm for lesions ≤ 2 cm<br>1.0 cm for lesions > 2 cm<br>In the setting of splenomegaly (> 13 cm), the splenic length must increase by > 50% of the extent of its prior increase beyond baseline (e.g., a 15-cm spleen must increase to > 16 cm). If no prior splenomegaly, must increase by at least 2 cm from baseline.<br>New or recurrent splenomegaly
Non-measured lesions	None	New or clear progression of pre-existing non-measured lesions
New lesions	New FDG-avid foci consistent with lymphoma rather than another etiology (e.g., infection, inflammation); if uncertain regarding etiology of new lesions, biopsy or interval scan may be considered	Regrowth of previously resolved lesions<br>A new node > 1.5 cm in any axis<br>A new extranodal site > 1.0 cm in any axis; if < 1.0 cm in any axis, its presence must be unequivocal and must be attributable to lymphoma<br>Assessable disease of any size unequivocally attributable to lymphoma
Bone marrow	New or recurrent FDG-avid foci	New or recurrent involvement

5PS = 5-point scale; CCT = computed tomography; FDG = fluorodeoxyglucose; IHC = immunohistochemistry; LDi = longest transverse diameter of a lesion; MRI = magnetic resonance imaging; PET = positron emission tomography; PPD = cross product of the LDi and perpendicular diameter; SDi = shortest axis perpendicular to the LDi; SPD = sum of the product of the perpendicular diameters for multiple lesions.

---

[a] A score of 3 in many patients indicates a good prognosis with standard treatment, especially if at the time of an interim scan. However, in trials involving PET where de-escalation is investigated, it may be preferable to consider a score of 3 as inadequate response (to avoid undertreatment). Measured dominant lesions: Up to six of the largest dominant nodes, nodal masses, and extranodal lesions selected to be clearly measurable in two diameters. Nodes should preferably be from disparate regions of the body and should include, where applicable, mediastinal and retroperitoneal areas. Non-nodal lesions include those in solid organs (e.g., liver, spleen, kidneys, lungs), gastrointestinal involvement, cutaneous lesions, or those noted on palpation. Non-measured lesions: Any disease not selected as measured; dominant disease and truly assessable disease should be considered not measured. These sites include any nodes, nodal masses, and extranodal sites not selected as dominant or measurable or that do not meet the requirements for measurability but are still considered abnormal, as well as truly assessable disease, which is any site of suspected disease that would be difficult to follow quantitatively with measurement, including pleural effusions, ascites, bone lesions, leptomeningeal disease, abdominal masses, and other lesions that cannot be confirmed and followed by imaging. In Waldeyer's ring or in extranodal sites (e.g., GI tract, liver, bone marrow), FDG uptake may be greater than in the mediastinum with complete metabolic response, but should be no higher than surrounding normal physiologic uptake (e.g., with marrow activation as a result of chemotherapy or myeloid growth factors).

[b] PET 5PS: 1 = no uptake above background; 2 = uptake $\leq$ mediastinum; 3 = uptake $>$ mediastinum but $\leq$ liver; 4 = uptake moderately $>$ liver; 5 = uptake markedly higher than liver and/or new lesions; X = new areas of uptake unlikely to be related to lymphoma.

Appendix 8
ASTCT Cytokine Release Syndrome Consensus Grading

CRS Parameter	Grade 1	Grade 2	Grade 3	Grade 4
Fever [a]	Temperature ≥ 38°C	Temperature ≥ 38°C	Temperature ≥ 38°C	Temperature ≥ 38°C
			with	
Hypotension	None	Not requiring vasopressors	Requiring a vasopressor with or without vasopressin	Requiring multiple vasopressors (excluding vasopressin)
			and/or [b]	
Hypoxia	None	Requiring low-glow nasal cannula [c] or blow-by	Requiring high-flow nasal cannula, facemask, nonrebreather mask or Venturi mask	Requiring positive pressure (e.g., CPAP, BiPAP, intubation and mechanical ventilation)

ASTCT = American Society for Transplantation and Cellular Therapy; BiPAP = bilevel positive airway pressure; CPAP = continuous positive airway pressure; CRS = cytokine release syndrome; CTCAE = Common Terminology Criteria for Adverse Events.

Grade 5 CRS is defined as death due to CRS in which another cause is not the principle factor leading to this outcome.

Organ toxicities associated with CRS may be graded according to CTCAE v5.0 but they do not influence CRS grading.

[a] Fever is defined as a temperature ≥ 38ºC not attributable to any other cause. In patients who have CRS then receive antipyretic or anticytokine therapy such as tocilizumab or steroids, fever is no longer required to grade subsequent CRS severity. In this case, CRS grading is determined by hypotension and/or hypoxia.

[b] CRS grade is determined by the more severe event, hypotension or hypoxia not attributable to any other cause. For example, a patient with temperature of 39.5 ºC, hypotension requiring 1 vasopressor, and hypoxia requiring low-flow nasal cannula is classified as Grade 3 CRS.

[c] Low-flow nasal cannula is defined as oxygen delivered at ≤ 6 L/minute. Low flow also includes blow-by oxygen delivery, sometimes used in pediatrics. High-flow nasal cannula is defined as oxygen delivered at > 6 L/minute.

Appendix 9
Examples of Sensitive In Vivo CYP Substrates and CYP Substrates with Narrow Therapeutic Range

CYP Enzymes [a]	Sensitive Substrates [b]	Substrates with Narrow Therapeutic Range [c]
CYP1A2	Alosetron, caffeine, duloxetine, melatonin, ramelteon, tacrine, tizanidine	Theophylline, tizanidine
CYP2B6 [d]	Bupropion, efavirenz	
CYP2C8	Repaglinide [e]	Paclitaxel
CYP2C9	Celecoxib	Warfarin, phenytoin
CYP2C19	Lansoprazole, omeprazole, S-mephenytoin	S-mephenytoin
CYP3A [f]	Alfentanil, aprepitant, budesonide, buspirone, conivaptan, darifenacin, darunavir, dasatinib, dronedarone, eletriptan, eplerenone, everolimus, felodipine, indinavir, fluticasone, lopinavir, lovastatin, lurasidone, maraviroc, midazolam, nisoldipine, quetiapine, saquinavir, sildenafil, simvastatin, sirolimus, tolvaptan, tipranavir, triazolam, vardenafil	Alfentanil, astemizole [g], cisapride [g], cyclosporine, dihydroergotamine, ergotamine, fentanyl, pimozide, quinidine, sirolimus, tacrolimus, terfenadine [g]
CYP2D6	Atomoxetine, desipramine, dextromethorphan, metoprolol, nebivolol, perphenazine, tolterodine, venlafaxine	Thioridazine

AUC = area under the concentration–time curve; CYP = cytochrome P450 enzymes.

[a] Note that this is not an exhaustive list. For an updated list, see the following link: https://www.fda.gov/Drugs/DevelopmentApprovalProcess/DevelopmentResources/DrugInteractionsLabeling/ucm080499.htm.

[b] Sensitive CYP substrates refer to drugs whose plasma AUC values have been shown to increase 5-fold or higher when co-administered with a known CYP inhibitor.

[c] CYP substrates with narrow therapeutic range refers to drugs whose exposure-response relationship indicates that small increases in their exposure levels by the concomitant use of CYP inhibitors may lead to serious safety concerns (e.g., torsades de pointes).

[d] The AUC of these substrates were not increased by 5-fold or more with a CYP2B6 inhibitor, but they represent the most sensitive substrates studied with available inhibitors evaluated to date.

[e] Repaglinide is also a substrate for OATP1B1, and it is only suitable as a CYP2C8 substrate if the inhibition of OATP1B1 by the investigational drug has been ruled out.

[f] Because a number of CYP3A substrates (e.g., darunavir, maraviroc) are also substrates of P-gp, the observed increase in exposure could be due to inhibition of both CYP3A and P-gp.

[g] Withdrawn from the U.S. market because of safety reason.

SAMPLE LIST OF CAUTIONARY MEDICATIONS

(A) INHIBITORS

	Strong Inhibitors	Moderate Inhibitors	Weak Inhibitors
CYP3A	Boceprevir, clarithromycin, cobicistat, conivaptan, danoprevir/ritonavir, elvitegravir/ritonavir, idelalisib,[a] indinavir, itraconazole, ketoconazole, mibefradil, lopinavir/ritonavir, nefazodone, nelfinavir, paritaprevir/ritonavir combinations, ritonavir, posaconazole, saquinavir, telaprevir, telithromycin, tipranavir/ritonavir, troleandomycin, voriconazole	Amprenavir, aprepitant, atazanavir, cimetidine, ciprofloxacin, clotrimazole, crizotinib,[a] cyclosporine,[a] darunavir/ritonavir, diltiazem, dronedarone, erythromycin, fluconazole, fluvoxamine, fosamprenavir, imatinib, [a] isavuconazole, tofisopam, verapamil	Chlorzoxazone, cilostazol, fosaprepitant, istradefylline, ivacaftor, lomitapide, ranitidine, ranolazine, tacrolimus, ticagrelor

[a] These are the anticancer agents; contact medical monitor before use.

(B) INDUCERS

	Strong Inducers	Moderate Inducers	Weak Inducers
CYP3A	Avasimibe, carbamazepine, enzalutamide, mitotane, phenytoin, rifampin, St. John's wort	Bosentan, efavirenz, etravirine, modafinil, nafcillin	Armodafinil, rufinamide

SOURCE:

U.S. Food and Drug Administration. Drug Development and Drug Interactions: Table of Substrates, Inhibitors and Inducers [resource on the Internet]. 2017 [cited 17 April 2018]. Available from: https://www.fda.gov/Drugs/DevelopmentApprovalProcess/DevelopmentResources/DrugInteractionsLabeling/ucm093664.htm.

Appendix 10
Recommended Anaphylaxis Management

The following equipment is needed in the event of a suspected anaphylactic reaction during study drug administration:

- Appropriate monitors (electrocardiogram, blood pressure, pulse oximetry)
- Oxygen and masks for oxygen delivery
- Airway management devices per standard of care
- Epinephrine for intravenous, intramuscular, and/or endotracheal administration in accordance with institutional guidelines
- Salbutamol (or albuterol or equivalent)
- Antihistamines (H1 and H2 blockers)
- Corticosteroids
- IV infusion solutions, tubing, catheters, and tape

The following are the procedures to follow in the event of a suspected anaphylactic reaction during study drug administration:

- Stop the study drug administration.
- Call for additional assistance.
- Maintain an adequate airway.
- Provide oxygen.
- Ensure that appropriate monitoring is in place, with continuous electrocardiogram and pulse oximetry monitoring, if possible.
- Administer epinephrine first, followed by antihistamines, albuterol, or other medications as required by patient status and directed by the physician in charge.
- Continue to observe the patient and document observations.

Appendix 11
Follicular Lymphoma International Prognostic Index, International Prognostic Index, and Mantle Cell Lymphoma International Prognostic Index

Follicular Lymphoma International Prognostic Index (FLIPI)	
Risk Factors	
Ann-Arbor Stage III or IV	
Age $>$ 60 years	
Serum LDH $>1\times$ ULN	
Anemia (hemoglobin $<$ 120 g/L)	
Involved nodal areas $>$ 4	
FLIPI Risk Group	Number of FLIPI Risk Factors
Low	0 or 1
Intermediate	2
High	3–5

FDG-PET = fluorodeoxyglucose–positron emission tomography (scan); FLIPI = Follicular Lymphoma International Prognostic Index; LDH = lactate dehydrogenase; ULN = upper limit of normal.

Note: The results of FDG-PET should not be taken into account for calculation of FLIPI as this prognostic score was established without FDG-PET.

Adapted from: Solal-Celigny P, Roy P, Colombat P, et al. Follicular lymphoma international prognostic index. Blood 2004;104:1258–64.

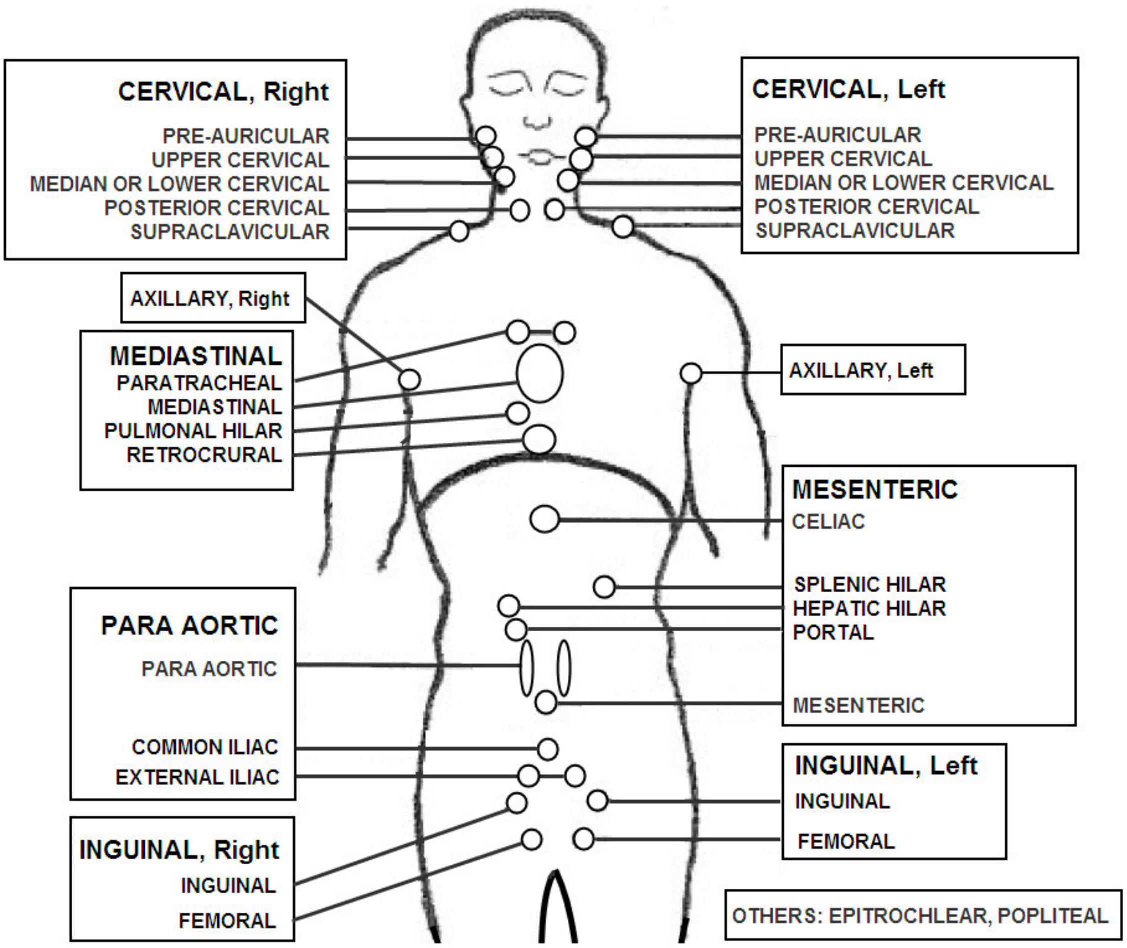

Reference modified from: Solal-Celigny P, Roy P, Colombat P, et al. Follicular lymphoma international prognostic index. Blood 2004;104:1258–64.

Follicular Lymphoma International Prognostic Index (FLIPI) 2	
Risk Factors	
Bone marrow involvement	
Age > 60 years	
β_2 microglobulin > 1 × ULN	
Anemia (hemoglobin < 120 g/L)	
Longest diameter of largest involved node > 6 cm	
FLIPI2 Risk Group	Number of FLIPI2 Risk Factors
Low	0
Intermediate	1 or 2
High	3–5

FDG-PET = fluorodeoxyglucose–positron emission tomography (scan); FLIPI2 = Follicular Lymphoma International Prognostic Index 2; LDH = lactate dehydrogenase; ULN = upper limit of normal.

Note: The results of FDG-PET should not be taken into account for calculation of FLIPI2 as this prognostic score was established without FDG-PET.

Adapted from: Federico M, Bellei M, Marcheselli L, et al. Follicular Lymphoma International Prognostic Index 2: a new prognostic index for follicular lymphoma developed by the International Follicular Lymphoma Prognostic Factor Project. J Clin Oncol 2009;27:4555–62.

Appendix 11 Follicular Lymphoma International Prognostic Index, International Prognostic Index, and Mantle Cell Lymphoma International Prognostic Index (cont.)

International Prognostic Index (IPI)	
Risk Factors	
Ann-Arbor Stage III or IV	
Age $>$ 60 years	
Serum LDH $> 1 \times$ ULN	
ECOG Performance Status ≥ 2	
Extranodal involvement ≥ 2	
IPI Risk Group	Number of IPI Risk Factors
Low	0 or 1
Low-intermediate	2
High-intermediate	3
High	4 or 5

ECOG = Eastern Cooperative Oncology Group; FDG-PET = fluorodeoxyglucose–positron emission tomography (scan); IPI = International Prognostic Index; LDH = lactate dehydrogenase; ULN upper limit of normal.

Note: The results of FDG-PET should not be taken into account for calculation of IPI as this prognostic score was established without FDG-PET.

Adapted from: Shipp MA, Harrington DP, Anderson JR, et al. A predictive model for aggressive Non-Hodgkin's Lymphoma. N Engl J Med 1993;329:987–94.

Appendix 11 Follicular Lymphoma International Prognostic Index, International Prognostic Index, and Mantle Cell Lymphoma International Prognostic Index (cont.)

Mantle Cell Lymphoma International Prognostic Index (MIPI)				
Points	Age, y	ECOG	LDH/ULN	WBC, 10^9/L
0	< 50	0–1	< 0.67	< 6.7
1	50-59	—	0.67-0.99	6.7–9.99
2	60-69	2–4	1.0-1.49	10.0–14.99
3	≥ 70	—	≥ 1.5	≥ 15.0
MIPI Risk Group	Total MIPI Points			
Low	0–3			
Intermediate	4–5			
High	6 or greater			

ECOG = Eastern Cooperative Oncology Group; LDH = lactate dehydrogenase; ULN = upper limit of normal; WBC = white blood cell.

Note: When Ki-67 is available, combined biologic MIPI score (CBS) can classify the risk group. The CBS is calculated as 0.03535 × age (years) + 0.6978 (if ECOG > 1) + 1.367 × log10 (LDH/ULN) + 0.9393 × log10 (WBC count) 0.02142 × Ki-67 (%). Low risk is defined by CBS < 5.7; intermediate risk, 5.7 ≤ CBS < 6.5; and high risk, CBS ≥ 6.5.

Adapted from: Hoster E, Dreyling M, Klapper W, et al. A new prognostic index (MIPI) for patients with advanced-stage mantle cell lymphoma. Blood 2008;111:558–65.

Appendix 12
EuroQol 5-Dimension, 5-Level (EQ-5D-5L) Questionnaire

<u>Do not reproduce or distribute</u>. *The Sponsor will provide sites with all instruments to be completed in this study.*

EQ-5D-5L

Sample

Health Questionnaire

English version for the UK

UK (English) © 2009 EuroQol Group EQ-5D™ is a trade mark of the EuroQol Group

Under each heading, please tick the ONE box that best describes your health TODAY.

MOBILITY

I have no problems in walking about ❑
I have slight problems in walking about ❑
I have moderate problems in walking about ❑
I have severe problems in walking about ❑
I am unable to walk about ❑

SELF-CARE

I have no problems washing or dressing myself ❑
I have slight problems washing or dressing myself ❑
I have moderate problems washing or dressing myself ❑
I have severe problems washing or dressing myself ❑
I am unable to wash or dress myself ❑

USUAL ACTIVITIES *(e.g. work, study, housework, family or leisure activities)*

I have no problems doing my usual activities ❑
I have slight problems doing my usual activities ❑
I have moderate problems doing my usual activities ❑
I have severe problems doing my usual activities ❑
I am unable to do my usual activities ❑

PAIN / DISCOMFORT

I have no pain or discomfort ❑
I have slight pain or discomfort ❑
I have moderate pain or discomfort ❑
I have severe pain or discomfort ❑
I have extreme pain or discomfort ❑

ANXIETY / DEPRESSION

I am not anxious or depressed ❑
I am slightly anxious or depressed ❑
I am moderately anxious or depressed ❑
I am severely anxious or depressed ❑
I am extremely anxious or depressed ❑

Sample

- We would like to know how good or bad your health is TODAY.
- This scale is numbered from 0 to 100.
- 100 means the best health you can imagine.
  0 means the worst health you can imagine.
- Mark an X on the scale to indicate how your health is TODAY.
- Now, please write the number you marked on the scale in the box below.

YOUR HEALTH TODAY =

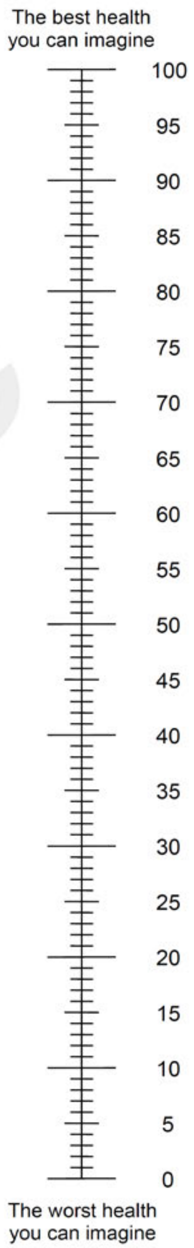

Sample

Appendix 13
Neurologic Adverse Events that May Affect Driving

Patients should be advised by the study investigator of potential neurologic toxicity, which may include seizures and alterations of consciousness.

Neurologic adverse events with the potential to impact cognition or consciousness that may affect driving (driving-impacting cognition or consciousness neurologic events [DI-CCNAE]) include, but are not limited to: amnesia, aphasia, confusional state, delirium, depressed level of consciousness, disturbance in attention, encephalopathy, hallucination, hepatic encephalopathy, insomnia, memory impairment, seizure, visual hallucination, and vertigo.

Neurologic adverse events with the potential to impact cognition or consciousness (cognition or consciousness neurologic events [CCNAE]) may include, but are not limited to: dizziness, insomnia, postural dizziness, and tremor. Patients with CCNAEs or Grade $\geq$3 neurologic adverse events should be assessed by neurologic examination to evaluate risk of impairment for driving or engaging in hazardous occupations or activities. When necessary, consult the Medical Monitor and obtain neurology consultation for evaluation of neurologic events that have the potential to impact cognition or consciousness.

Appendix 14
Recommendations for the Use of White Blood Cell Growth Factors

PRIMARY PROPHYLACTIC G-CSF ADMINISTRATION (FIRST AND SUBSEQUENT-CYCLE USE)

Primary prophylaxis with growth-colony stimulating factor (G-CSF) is recommended if any of the following clinical factors are present:

- Age $\geq$65 years
- Advanced disease
- Previous chemotherapy or radiation therapy
- Preexisting neutropenia or bone marrow involvement with tumor
- Infection
- Open wounds or recent surgery
- Poor performance status or poor nutritional status
- Poor renal function
- Liver dysfunction, most notably elevated bilirubin
- Cardiovascular disease
- Multiple comorbid conditions

SECONDARY PROPHYLACTIC G-CSF ADMINISTRATION

Prophylactic G-CSF administration is recommended for patients who fulfill each of the following circumstances:

- Experienced a neutropenic complication from a prior cycle of study treatment for which primary prophylactic G-CSF was not received; and
- The intent is to avoid dose reduction or treatment delay, which may compromise disease-free or overall survival or treatment outcome.

THERAPEUTIC USE OF G-CSF

Administration G-CSF should be considered for the following patients:

- Patients with febrile neutropenia who are at high risk for infection-associated complications; or
- Patients who have prognostic factors that are predictive of poor clinical outcome, e.g., prolonged ($>$10 days) and profound ($<$100/mm^3) neutropenia, age $>$65 years, uncontrolled primary disease, pneumonia, hypotension and multi-organ dysfunction (sepsis), invasive fungal infection, being hospitalized at the time of fever development.

REFERENCES

Smith TJ, Bohlke K, Lyman GH, et al. Recommendations for the use of WBC growth factors: American Society of Clinical Oncology Clinical Practice Guideline Update. J Clin Oncol 2015b;33:3199−212.

Appendix 15
Definitions of Laboratory and Clinical Tumor Lysis Syndrome

Table 1. Definitions of Laboratory and Clinical Tumor Lysis Syndrome.*

Metabolic Abnormality	Criteria for Classification of Laboratory Tumor Lysis Syndrome	Criteria for Classification of Clinical Tumor Lysis Syndrome
Hyperuricemia	Uric acid >8.0 mg/dl (475.8 μmol/liter) in adults or above the upper limit of the normal range for age in children	
Hyperphosphatemia	Phosphorus >4.5 mg/dl (1.5 mmol/liter) in adults or >6.5 mg/dl (2.1 mmol/liter) in children	
Hyperkalemia	Potassium >6.0 mmol/liter	Cardiac dysrhythmia or sudden death probably or definitely caused by hyperkalemia
Hypocalcemia	Corrected calcium <7.0 mg/dl (1.75 mmol/liter) or ionized calcium <4.5 mg/dl (1.12 mmol/liter)†	Cardiac dysrhythmia, sudden death, seizure, neuromuscular irritability (tetany, paresthesias, muscle twitching, carpopedal spasm, Trousseau's sign, Chvostek's sign, laryngospasm, or bronchospasm), hypotension, or heart failure probably or definitely caused by hypocalcemia
Acute kidney injury‡	Not applicable	Increase in the serum creatinine level of 0.3 mg/dl (26.5 μmol/liter) (or a single value >1.5 times the upper limit of the age-appropriate normal range if no baseline creatinine measurement is available) or the presence of oliguria, defined as an average urine output of <0.5 ml/kg/hr for 6 hr

* In laboratory tumor lysis syndrome, two or more metabolic abnormalities must be present during the same 24-hour period within 3 days before the start of therapy or up to 7 days afterward. Clinical tumor lysis syndrome requires the presence of laboratory tumor lysis syndrome plus an increased creatinine level, seizures, cardiac dysrhythmia, or death.

† The corrected calcium level in milligrams per deciliter = measured calcium level in milligrams per deciliter + 0.8 × (4 − albumin in grams per deciliter).

‡ Acute kidney injury is defined as an increase in the creatinine level of at least 0.3 mg per deciliter (26.5 μmol per liter) or a period of oliguria lasting 6 hours or more. By definition, if acute kidney injury is present, the patient has clinical tumor lysis syndrome. Data about acute kidney injury are from Levin et al.[11]

Note: Tumor lysis syndrome should be graded according to National Cancer Institute Common Terminology Criteria for Adverse Events, Version 4.0.

Adapted from: Howard SC, Jones DP, Pui CH. The tumor lysis syndrome. N Engl J Med 2011;364:1844–54.

Appendix 16
Cumulative Illness Rating Scale-Geriatric (CIRS-G)

Organ System	Score	Rating Strategy
Heart	0	No problem
	+1	MI >5 years ago, occasional angina treated with meds as needed
	+2	CHF compensated with meds, daily antianginal meds, left ventricular hypertrophy, atrial fibrillation, bundle branch block, daily antiarrhythmic drugs
	+3	MI ≤5 years ago, abnormal stress test, or past PTCA or CABG
	+4	Marked activity restriction secondary to cardiac status (i.e., unstable angina or intractable CHF)
Vascular	0	No problem
	+1	Hypertension compensated with salt restriction and weight loss, cholesterol >200 mg/dL
	+2	Daily antihypertensive meds, one symptom of atherosclerotic disease (angina, claudication, bruit, amaurosis fugax, absent pedal pulses), aortic aneurysm <4 cm
	+3	≥2 symptoms of atherosclerosis
	+4	Previous vascular surgery, aortic aneurysm ≥4 cm
Hematopoietic *(hematologic deficiencies due to lymphoma should not be included in the score, see Section 4.5.11)*	0	No problem *(or if the hematologic deficiencies are due to lymphoma)*
	+1	Hemoglobin: females 10-12 g/dL, males 12-14 g/dL, anemia of chronic disease
	+2	Hemoglobin: females 8 to <10 g/dL, males 10 to <12 g/dL, anemia secondary to iron/vitamin B-12/folate deficiency or chronic renal failure, total WBC 2,000–4,000
	+3	Hemoglobin: females <8 g/dL, males <10 g/dL, total WBC <2,000
	~~+4~~	~~Any leukemia or lymphoma~~ *(This score should not be included due to lymphoma as underlying disease in this study).*
Respiratory	0	No problem
	+1	Recurrent episodes of acute bronchitis, current treated asthma with inhalers as needed, cigarette smoker 10–20 pack years
	+2	X-ray evidence of COPD, requires daily theophylline or inhalers, treated for pneumonia two or more times in the past 5 years, smoked 21–40 pack years
	+3	Limited ambulation secondary to limited respiratory capacity, requires oral steroids for lung disease, smoked >40 pack years
	+4	Requires supplemental oxygen, ≥1 episode of respiratory failure requiring assisted ventilation, any lung cancer

Eyes, ears, nose, throat, and larynx	0	No problem
	+1	Corrected vision 20/40, chronic sinusitis, mild hearing loss
	+2	Corrected vision 20/60 or reads newsprint with difficulty, requires hearing aid, chronic sinonasal complaints requiring medication, requires medication for vertigo
	+3	Partially blind (requires an escort to venture out), unable to read newsprint, conversational hearing still impaired with hearing aid
	+4	Functional blindness, functional deafness, laryngectomy, requires surgical intervention for vertigo
Upper GI	0	No problem
	+1	Hiatal hernia, heartburn complaints treated with as-needed meds
	+2	Needs daily H_2 blocker or antacid, documented gastric or duodenal ulcer within 5 years
	+3	Active ulcer, guaiac positive stools, any swallowing disorder or dysphagia
	+4	Gastric cancer, history of perforated ulcer, melena or hematochezia from upper GI source
Lower GI	0	No problem
	+1	Constipation managed with meds as needed, active hemorrhoids, status post hernia repair
	+2	Requires daily bulk laxatives or stool softeners, diverticulosis, untreated hernia
	+3	Bowel impaction in the past year, daily use of stimulant laxatives or enemas
	+4	Hematochezia from lower GI source, currently impacted, diverticulitis flare up, status post bowel obstruction, bowel carcinoma
Liver, pancreas, and biliary	0	No problem
	+1	History of hepatitis >5 years ago, cholecystectomy
	+2	Mildly elevated LFTs (≤150% of normal), hepatitis within 5 years, cholelithiasis, daily or heavy alcohol use within 5 years
	+3	Elevated bilirubin (total >2 mg/dL), marked elevation of LFTs (>150% of normal), requires supplemental pancreatic enzymes for digestion
	+4	Clinical or lab evidence of biliary obstruction, any biliary tree carcinoma, cholecystitis, pancreatitis, active hepatitis+

Renal	0	No problem
	+1	Kidney stone passage within the past 10 years or asymptomatic kidney stone, pyelonephritis within 5 years
	+2	Serum creatinine 1.5-3.0 mg/dL without diuretic or antihypertensive medication
	+3	Serum creatinine >3.0 mg/dL OR serum creatinine >1.5 mg/dL on diuretic, antihypertensive, or bicarbonate therapy, current pyelonephritis
	+4	Requires dialysis, renal carcinoma
Genitourinary	0	No problem
	+1	Stress incontinence, hysterectomy, BPH without urinary symptoms
	+2	Abnormal pap smear, frequent UTIs (≥3 in past year), urinary incontinence (non-stress) in females, BPH with hesitancy or frequency, current UTI, any urinary diversion procedure, status post TURP
	+3	Prostate cancer in situ (i.e., found incidentally during TURP), vaginal bleeding, cervical carcinoma in situ, hematuria, status post urosepsis in past year
	+4	Acute urinary retention, any GU carcinoma except as above
Musculoskeletal and skin	0	No problem
	+1	Uses meds as needed for arthritis or has mildly limited ADLs from joint pathology, excised non-melanoma skin cancers, skin infections requiring antibiotics within a year
	+2	Daily antiarthritic medications or use of assistive devices or moderate limitation in ADLs, daily meds for chronic skin conditions, melanoma without metastasis
	+3	Severely impaired ADLs secondary to arthritis, requires steroids for arthritic condition, vertebral compression fractures from osteoporosis
	+4	Wheelchair bound, severe joint deformity or severely impaired usage, osteomyelitis, any bone or muscle carcinoma, metastatic melanoma
Neurologic	0	No problem
	+1	Frequent headaches requiring meds as needed without interference with daily activities, history of TIA phenomena (at least one)
	+2	Requires daily meds for chronic headaches or headaches that regularly interfere with daily activities, status post CVA without significant residual, mild neurodegenerative disease (Parkinson's, MS, ALS, etc.)
	+3	Status post CVA with mild residual dysfunction, any CNS neurosurgical procedure, moderate neurodegenerative disease
	+4	Status post CVA with residual functional hemiparesis or aphasia, severe neurodegenerative disease

Endocrine and breast	0	No problem
	+1	Diabetes mellitus compensated with diet, obesity (BMI > 30), requires thyroid hormone replacement
	+2	Diabetes mellitus requiring insulin or oral agents, fibrocystic breast disease
	+3	Any electrolyte disturbance requiring hospital treatment, morbid obesity (BMI > 45)
	+4	Brittle or poorly controlled diabetes mellitus or diabetic coma in the past year, requires adrenal hormone replacement, adrenal, thyroid, or breast carcinoma
Psychiatric illness (Original study references DSM III-R)	0	No problem
	+1	Minor psychiatric condition or history thereof: specifically, previous outpatient mental health treatment during a crisis, outpatient treatment for depression > 10 years ago, current use of minor tranquilizers for episodic anxiety (occasional usage), mild early dementia
	+2	A history of major depression (by DSM) within the past 10 years (treated or untreated), mild dementia, any previous psychiatric hospitalization, any psychotic episode substance abuse history > 10 years ago
	+3	Currently meets DSM criteria for major depression or two or more episodes of major depression in the past 10 years, moderate dementia, current usage of daily anti-anxiety medication, currently meets DSM criteria for substance abuse or dependency, requires daily antipsychotic medication
	+4	Current mental illness requiring psychiatric hospitalization, institutionalization, or intensive outpatient management (e.g. patients with severe or suicidal depression, acute psychosis or psychotic decompensation, severe agitation from dementia, severe substance abuse, etc.), severe dementia

ADLs = activities of daily living; ALS = amyotrophic lateral sclerosis; BMI = body mass index; BPH = benign prostatic hyperplasia; CABG = coronary artery bypass grafting; CHF = congestive heart failure; COPD = chronic obstructive pulmonary disease; CVA = cerebrovascular accident; DSM = Diagnostic and Statistical Manual of Mental Disorders; DSM III-R = DSM, Third Edition, Revised; GI = gastrointestinal; GU = genitourinary; LFTs = liver function tests; MI = myocardial infarction; MS = multiple sclerosis; PTCA = *percutaneous* transluminal coronary angioplasty; TIA = transient ischemic attack; TURP = transurethral resection of the prostate; UTI = urinary tract infection.

REFERENCES

Parmelee PA, Thuras PD, Katz IR, et al. Validation of the Cumulative Illness Rating Scale in a geriatric residential population. J Am Geriatr Soc 1995;43(2):130–7.

Merli F, Luminari S, Tucci A, et al. Simplified geriatric assessment in older patients with diffuse large B-cell lymphoma: The prospective elderly project of the fondazione italiana linfomi. J. Clin. Oncol 2021;39(11):1214–22.

Merli F, Luminari S, et al. Outcome of frail elderly patients with diffuse large B-cell lymphoma prospectively identified by Comprehensive Geriatric Assessment: results from a study of the Fondazione Italiana Linfomi. Leuk Lymphoma 2014;55(1):38-43.

Signature Page for Published - Protocol - GO40516 - LUNSUMIO - v8 - Global/Core
System identifier: RIM-CLIN-450853

Approval Task	[redacted]<br>Company Signatory<br>14-Sep-2022 05:48:33 GMT+0000
