# Supplementary material for: Mosunetuzumab with polatuzumab vedotin in relapsed or refractory aggressive large B cell lymphoma: a phase 1b/2 trial
Source: Nat Med. 2023 Dec 10;30(1):229–39. doi: 10.1038/s41591-023-02726-5 (PMC10803244; doi:10.1038/s41591-023-02726-5)
Supplement: Supplementary file 2 — Reporting Summary [file 41591_2023_2726_MOESM2_ESM.pdf]

nature portfolio

Corresponding author(s): L. Elizabeth Budde

Last updated by author(s): Nov 16, 2023

Reporting Summary

Nature Portfolio wishes to improve the reproducibility of the work that we publish. This form provides structure for consistency and transparency in reporting. For further information on Nature Portfolio policies, see our Editorial Policies and the Editorial Policy Checklist.

Statistics

For all statistical analyses, confirm that the following items are present in the figure legend, table legend, main text, or Methods section.

n/a	Confirmed	
☐	☒	The exact sample size (*n*) for each experimental group/condition, given as a discrete number and unit of measurement
☐	☒	A statement on whether measurements were taken from distinct samples or whether the same sample was measured repeatedly
☐	☒	The statistical test(s) used AND whether they are one- or two-sided<br>*Only common tests should be described solely by name; describe more complex techniques in the Methods section.*
☐	☒	A description of all covariates tested
☐	☒	A description of any assumptions or corrections, such as tests of normality and adjustment for multiple comparisons
☐	☒	A full description of the statistical parameters including central tendency (e.g. means) or other basic estimates (e.g. regression coefficient) AND variation (e.g. standard deviation) or associated estimates of uncertainty (e.g. confidence intervals)
☐	☒	For null hypothesis testing, the test statistic (e.g. *F*, *t*, *r*) with confidence intervals, effect sizes, degrees of freedom and *P* value noted<br>*Give P values as exact values whenever suitable.*
☒	☐	For Bayesian analysis, information on the choice of priors and Markov chain Monte Carlo settings
☐	☒	For hierarchical and complex designs, identification of the appropriate level for tests and full reporting of outcomes
☐	☒	Estimates of effect sizes (e.g. Cohen's *d*, Pearson's *r*), indicating how they were calculated

Our web collection on statistics for biologists contains articles on many of the points above.

Software and code

Policy information about availability of computer code

Data collection	No software was used for data collection.
Data analysis	FACSDiva software (BD Biosciences, version 6.1.3, FCS Express (DeNovo, Version 4, Clinical Edition), and SAS version 9.4

For manuscripts utilizing custom algorithms or software that are central to the research but not yet described in published literature, software must be made available to editors and reviewers. We strongly encourage code deposition in a community repository (e.g. GitHub). See the Nature Portfolio guidelines for submitting code & software for further information.

Data

Policy information about availability of data

All manuscripts must include a data availability statement. This statement should provide the following information, where applicable:

- Accession codes, unique identifiers, or web links for publicly available datasets
- A description of any restrictions on data availability
- For clinical datasets or third party data, please ensure that the statement adheres to our policy

The sponsor was involved in the design and conduct of the study, collection, management, analysis, and interpretation of the data. All authors had full access to the data in the study.

Qualified researchers may request access to individual patient-level data through the clinical study data request platform (https://vivli.org/). Further details on

Roche's criteria for eligible studies are available at https://vivli.org/members/ourmembers/. For further details on Roche's Global Policy on the Sharing of Clinical Information and how to request access to related clinical study documents, see https://www.roche.com/ research_and_development/ who_we_are_how_we_work/clinical_trials/our_commitment_to_data_sharing.htm.

Research involving human participants, their data, or biological material

Policy information about studies with human participants or human data. See also policy information about sex, gender (identity/presentation), and sexual orientation and race, ethnicity and racism.

Reporting on sex and gender	GO40516 collected sex data as reported by the medical treatment team and the patient. Sex was not considered in the study design as large B cell lymphoma is a disease that affects both sexes. However, sex based response to treatment were analyzed and provided in forest plots.
Reporting on race, ethnicity, or other socially relevant groupings	The study reported recruitment and response outcomes based on ethnicity (Hispanic or Latino; Not Hispanic or Latino; Not stated or unknown) and race (American Indian or Alaska Native; Asian; Black or African American; White; Unknown). Ethnicity and race were self reported. Subgroup analysis based on race and ethnicity was not possible because of small subgroup sizes.
Population characteristics	Provided in Table 1 in the manuscript.
Recruitment	This trial was open label with no blinding. Patients were recruited in 15 sites across two countries (US and Canada) among patients of the site, or among patients referred from other hospitals from September 2018 to February 2022. Sites were either at academic or community hospitals. The phase I portion of the clinical trial was enrolled based on slot availability, the study had established sites for the study that would screen patients at those locations to determine eligibility for the trial. The phase II portion was an open enrollment to the active participating sites. To participate in this study and before any non-routine baseline or screening evaluation, investigators at each study site ensured that each patient was fully informed of the study and had signed a written informed consent. The patient's eligibility was evaluated during the screening period prior to enrollment. No bias emerging from recruitment is expected. Patients were enrolled irrespective of gender, which was self-reported by the patient. Randomization was not performed as we report results from the single-arm dose expansion cohort.
Ethics oversight	The trial was conducted in accordance with the Declaration of Helsinki, International Conference on Harmonization Guidelines for Good Clinical Practice, and applicable laws and regulations. The study was approved by institutional review boards or ethics committees at each center (WCG Clinical, Inc.; NYU School of Medicine-Office of Science and Research Institutional Review Board; University of Miami- Human Subject Research Office; Wayne State University-IRB Administration Office; Advarra; Lifespan-Research Protection, Office-Office Research; Quebec-Integrated Health and Social Services, University Network for West-Central Montreal; University of Saskatchewan- Biomedical Research Ethics Board, Royal University Hospital; PennState Health Milton S. Hershey Medical Center, Institutional Review Board Human Subjects Protection Office.

Note that full information on the approval of the study protocol must also be provided in the manuscript.

Field-specific reporting

Please select the one below that is the best fit for your research. If you are not sure, read the appropriate sections before making your selection.

☒ Life sciences ☐ Behavioural & social sciences ☐ Ecological, evolutionary & environmental sciences

For a reference copy of the document with all sections, see nature.com/documents/nr-reporting-summary-flat.pdf

Life sciences study design

All studies must disclose on these points even when the disclosure is negative.

Sample size	The sample size of the dose-escalation cohort was based on dose-escalation rules, which was a modified 3+3 design; a minimum of three patients were initially enrolled in each cohort to evaluate DLTs. If none of the first three DLT-evaluable patients experienced a DLT, then enrollment of the next cohort could proceed. If a patient experienced a DLT, then the cohort was expanded to six patients to be evaluated for additional DLTs. For the phase 2 dose-expansion cohort, a sample size of 100 patients was calculated to provide 99% power to detect a difference in ORR, with a two-sided significance level of 5%. Please see section 6 of the protocol for more information on sample size.
Data exclusions	Three patients with histologically confirmed grade 1-3a follicular lymphoma were excluded from the efficacy analysis.
Replication	Not applicable for this study.
Randomization	Not applicable for this study as we report results from the single-arm cohort of the clinical trial.
Blinding	Not applicable for this study as we report an open-label clinical trial with no blinding.

Reporting for specific materials, systems and methods

We require information from authors about some types of materials, experimental systems and methods used in many studies. Here, indicate whether each material, system or method listed is relevant to your study. If you are not sure if a list item applies to your research, read the appropriate section before selecting a response.

Materials & experimental systems

n/a	Involved in the study
☒	☐ Antibodies
☒	☐ Eukaryotic cell lines
☒	☐ Palaeontology and archaeology
☒	☐ Animals and other organisms
☐	☒ Clinical data
☒	☐ Dual use research of concern
☒	☐ Plants

Methods

n/a	Involved in the study
☒	☐ ChIP-seq
☐	☒ Flow cytometry
☒	☐ MRI-based neuroimaging

Clinical data

Policy information about clinical studies

All manuscripts should comply with the ICMJE guidelines for publication of clinical research and a completed CONSORT checklist must be included with all submissions.

Clinical trial registration

NCT03671018

Study protocol

Redacted protocol provided with submission

Data collection

Between September 25, 2018, and February 14, 2022, 120 patients were enrolled from 15 sites across two countries. Sites were either at academic or community hospitals.

Outcomes

The primary objectives in the dose-escalation cohort were to evaluate safety and tolerability, and to determine any dose-limiting toxicities (DLTs), the maximum tolerated dose, and the RP2D of mosunetuzumab in combination with polatuzumab vedotin 1.8 mg/kg. The secondary objectives were: anti-tumor activity, determined by measuring the CR rate at the time of PRA based on PET–CT; best objective response rate (ORR; CR or PR at any time) on study, based on PET and/or CT scan; and duration of response (DoR), defined as the time from the first occurrence of a documented ORR to PD or relapse, or death from any cause, whichever occurred first. Response was determined by the investigator (INV) using Lugano 2014 criteria.

The primary efficacy endpoint in the dose-expansion cohort was best ORR based on PET–CT and/or CT scan by Independent Review Committee (IRC) using Lugano 2014 criteria. Secondary efficacy endpoints were also assessed using Lugano 2014 criteria and included: best ORR on study based on PET–CT and/or CT scan determined by INV; best CR rate and CR rate at PRA based on PET–CT and/or CT scan determined by INV and IRC; DoR determined by INV and IRC; progression-free survival (PFS), defined as the time from first study treatment to the first occurrence of PD or relapse, or death from any cause, whichever occurred first, determined by INV and IRC; and overall survival, defined as the time from first study treatment to death from any cause. AEs were reported using NCI CTCAE version 5.0. CRS events were graded according to ASTCT criteria

As this was a single-arm dose escalation and expansion trial, surrogate efficacy endpoints were determined for the primary and secondary efficacy objectives. These were assessed by using the Lugano 2014 criteria, in which tumor responses were determined based on PET-CT and/or CT-only scans. For safety, the investigators would assess adverse events based on the NCI-CTCAE Version 5 grading criteria. For the dose-expansion cohort, the primary endpoint of best ORR based on PET–CT and/or CT scan by Independent Review Committee (IRC) using Lugano 2014 criteria was prespecified to compare against historical control ORR of 42%. All secondary endpoints were prespecified in the Protocol. No hierarchical testing was performed.

Plants

Seed stocks

Report on the source of all seed stocks or other plant material used. If applicable, state the seed stock centre and catalogue number. If plant specimens were collected from the field, describe the collection location, date and sampling procedures.

Novel plant genotypes

Describe the methods by which all novel plant genotypes were produced. This includes those generated by transgenic approaches, gene editing, chemical/radiation-based mutagenesis and hybridization. For transgenic lines, describe the transformation method, the number of independent lines analyzed and the generation upon which experiments were performed. For gene-edited lines, describe the editor used, the endogenous sequence targeted for editing, the targeting guide RNA sequence (if applicable) and how the editor was applied.

Authentication

Describe any authentication procedures for each seed stock used or novel genotype generated. Describe any experiments used to assess the effect of a mutation and, where applicable, how potential secondary effects (e.g. second site T-DNA insertions, mosiacism, off-target gene editing) were examined.

Flow Cytometry

Plots

Confirm that:

☒ The axis labels state the marker and fluorochrome used (e.g. CD4-FITC).

☒ The axis scales are clearly visible. Include numbers along axes only for bottom left plot of group (a 'group' is an analysis of identical markers).

☒ All plots are contour plots with outliers or pseudocolor plots.

☒ A numerical value for number of cells or percentage (with statistics) is provided.

Methodology

Sample preparation	Whole blood samples were collected in BD Vacutainer tubes containing sodium heparin.<br><br>For cell labeling, samples were labeled with antibodies for 30 minutes in the dark, at ambient temperature. Red blood cells (RBC) were then lysed, utilizing Biolegend RBC Lysis Buffer for 15 minutes at ambient temperature. After centrifugation samples were washed with BD Stain Buffer and resuspended in 1% formalin fixative. Assay panel tubes were stored at 4 °C and acquired within four hours of preparation.
Instrument	Samples were acquired on BD FACSCanto II flow cytometers (BD Biosciences, designated at ILS-Dublin Canto D, s/n V33896201828), using FACSDiva software (BD Biosciences, version (6.1.3).
Software	FACSDiva software (BD Biosciences, version 6.1.3) and FCS Express (DeNovo, Version 4 Clinical Edition)
Cell population abundance	No cell sorting was done for these on-study samples.
Gating strategy	Antibodies used in the panel were:<br>Mouse anti-human HLA-DR PerCP-Cy5.5 (clone G46-6, BD Biosciences, Catalog #560652)<br>Mouse anti-human CD69 PE-Cy7 (clone FN50, BD Biosciences, Catalog #557745)<br>Mouse anti-human CD4 BV510 (clone SK3, BD Biosciences, Catalog #562970)<br>Mouse anti-human CD8 APC-H7 (clone SK-1, BD Biosciences, Catalog #561423)<br><br>Global MFI target ranges for Sphero Ultra Rainbow Calibration Beads Lot #AJ01 were used for this validation study to standardize instrument fluorescence output according to ICON's Cellular Immunology Manual LB310-SOP5 between January 2018 and February 2018.<br><br>Compensation was determined prior to acquisition of samples using the automated compensation function in FACSDiva Assessed was a negative control of unlabeled BD CompBeads and positive controls of anti-mouse IgG κ BD CompBead. For sample acquisition, the FSC threshold and PMT settings for FSC and SSC were adjusted as needed using a single tube. All samples were collected using a medium flow rate, using a stop gate of 100, 000 CD4+ or CD8+ T cells, or for a maximum of 270 seconds.

☒ Tick this box to confirm that a figure exemplifying the gating strategy is provided in the Supplementary Information.
